# Convergent Synthesis of Dihydrobenzofurans via Urea Ligand-Enabled Heteroannulation of 2-Bromophenols with 1,3-Dienes

**DOI:** 10.1021/acs.orglett.2c02301

**Published:** 2022-07-29

**Authors:** Kaitlyn E. Houghtling, Amanda M. Canfield, Shauna M. Paradine

**Affiliations:** Department of Chemistry, University of Rochester, Rochester, New York 14627, United States

## Abstract

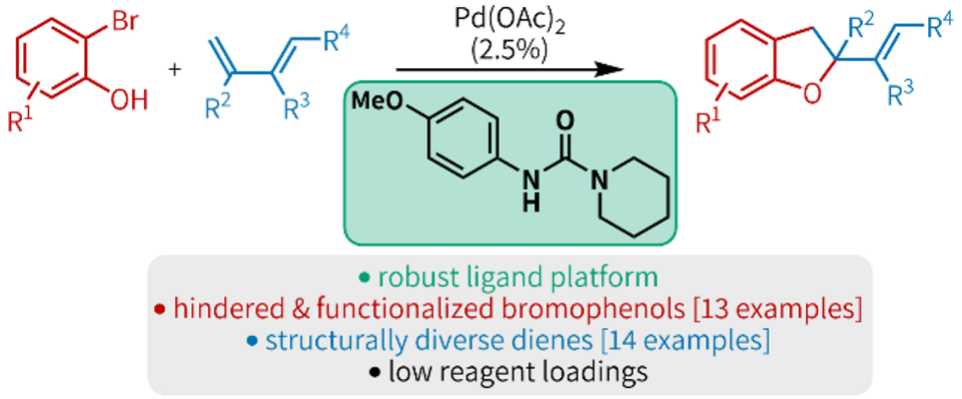

We disclose a palladium and urea ligand-mediated heteroannulation of 2-bromophenols and 1,3-dienes. This method addresses synthetic challenges present in the palladium-catalyzed heteroannulation of bifunctional reagents and olefins by engaging a diverse scope of coupling partners under a unified set of reaction conditions. Our recently developed urea ligand platform outperforms phosphine ligands to generate the dihydrobenzofuran motif in a convergent manner.

Dihydrobenzofuran (DHB) and other furan derivatives are prevalent core scaffolds in natural products^1−3^ and as therapeutics,^4,5^ organic materials,^6,7^ and agrochemicals.^8^ There are a number of modern approaches for the preparation of DHBs^9^ (Figure 1a), including phenol alkylation,^10^ intramolecular carbene insertion,^11^ benzofuran reduction,^10^ and ring contraction.^12^ Although these methods are well established, most are intramolecular and require reagents that are prepared with significant synthetic overhead, factors that make it arduous to build representative small molecule DHB libraries.

**Figure 1:**
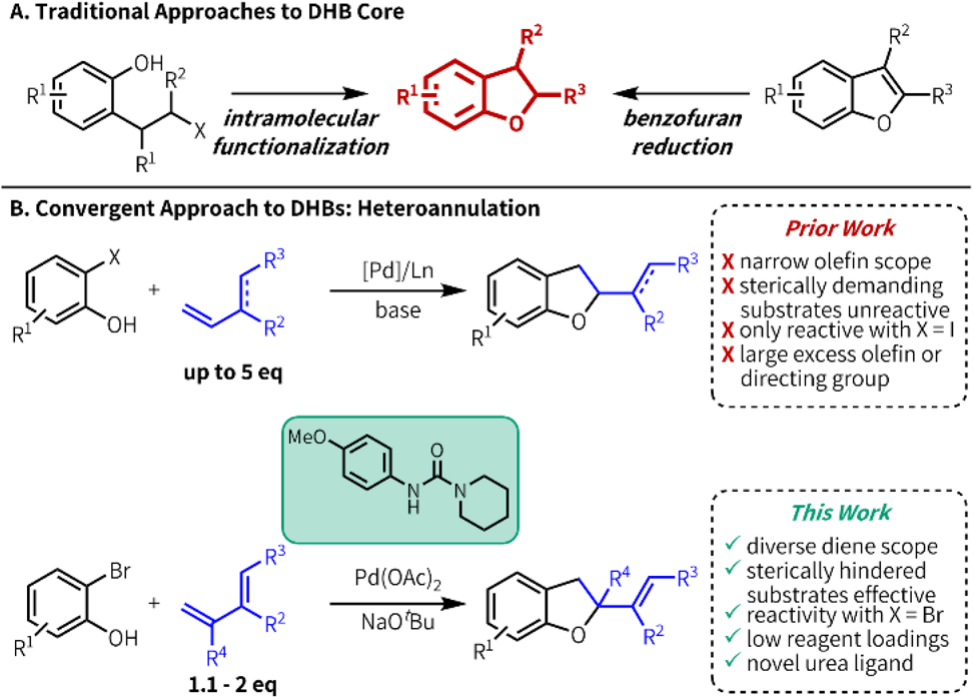
(A) Traditional approaches to access DHB cores. (B) A convergent approach to DHBs: Pd-catalyzed heteroannulation reactions of olefins and bifunctional reagents.

An attractive alternative approach to the synthesis of DHBs is through a multicomponent annulation where both C–C and C–O bonds are formed in one step (Figure 1b). This convergent strategy allows for greater variation in reagents, making it more amenable to the preparation of diverse libraries of DHB-containing compounds. One method demonstrating these qualities is palladium-mediated coupling of functionalized phenols with olefins.^13,14^ Pioneering studies have made important advances to establish the viability of this transformation, but the synthetic utility remains limited. Existing methods typically have a narrow substrate scope with respect to one or both coupling partners–activated alkenes or linear 1,3-dienes are usually required^13d−13f^ – and are intolerant of steric encumbrance in either. Reaction conditions can be substrate dependent, and large excess of the olefin (3–5 equiv.) is common in the absence of a directing group or tethering of the nucleophile to the olefin.^13d,13g,13h^ Herein, we report a urea ligand-enabled heteroannulation reaction under palladium catalysis that provides access to structurally and functionally diverse DHB products.

Very recently, our group has advanced urea-derived ligands as an alternative ligand platform for palladium catalysis.^15^ We found that urea ligands, which are small and kinetically labile, are effective for the palladium-catalyzed heteroannulation of *N*-tosyl-bromoanilines and 1,3-dienes. In addition to the potential to uncover complementary reactivity and selectivity to traditional ligands for palladium, these ligands possess practical features that make them attractive: they are readily prepared from widely available and inexpensive amine precursors, and are bench stable and robust to a variety of conditions. Given these features and our success in heteroannulation reactions forming indolines, we sought to extend our urea-enabled methodology to the use of bromophenols as bifunctional reagents. By using trisubstituted urea ligands, we can now access the analogous DHB core. Our urea-enabled method engages a diverse scope of bromophenols and dienes under a unified set of reaction conditions. This method is a convergent approach for generating a representative library of functionalized DHBs.

In our initial studies, we examined the effect of various ligands on the desired heteroannulation of 2-bromophenol **1a** and diene **2a** (Figure 2a). Without any ligand, the reaction afforded the desired product **3aa** in 49% yield; reactivity in the absence of exogenous ligand was poorer for more challenging branched dienes.^16^ Although we did not observe inhibition by phosphines,^17^ there was generally no discernible ligand effect across a range of phosphines. The lack of any effect is unexpected–we confirmed via ^31^P NMR that strong phosphine binding occurs under conditions relevant to catalysis–and elucidating the nature of this phenomenon will be the subject of future study.^16,18^ Product yield noticeably improved when urea was used; with **4a** as ligand, **3aa** was isolated in 68% yield. With this result in hand, we explored the effect that substituting the urea has on reactivity. Although we had previously found monosubstituted ureas to be optimal, in this case trisubstituted urea **4d** performed comparably to **4b** (57% vs 54%); disubstituted urea **4c** showed no ligand effect, and tetrasubstituted urea **4g** inhibited the reaction. Introducing a *para*-OMe group to the *N*-aryl substituent, as in **4e**, further improved product yield (65%); electron-withdrawing groups did not affect reactivity (**4f**) and no clear electronic trend was observed. Further investigation of substituent effects revealed that piperidine is the most effective group; other cyclic and acyclic amines perform worse (**4h**–**k**). Ultimately, **4e** maintained good reactivity across a wider range of substrates than any other phosphine or urea ligand investigated, including **4a** (see Figure 4 [**3aj**]).^16^ A 2:1 ligand/palladium ratio was optimal; product yield dropped significantly with 1:1 **4e**/Pd(OAc)_2_.^16^

**Figure 2:**
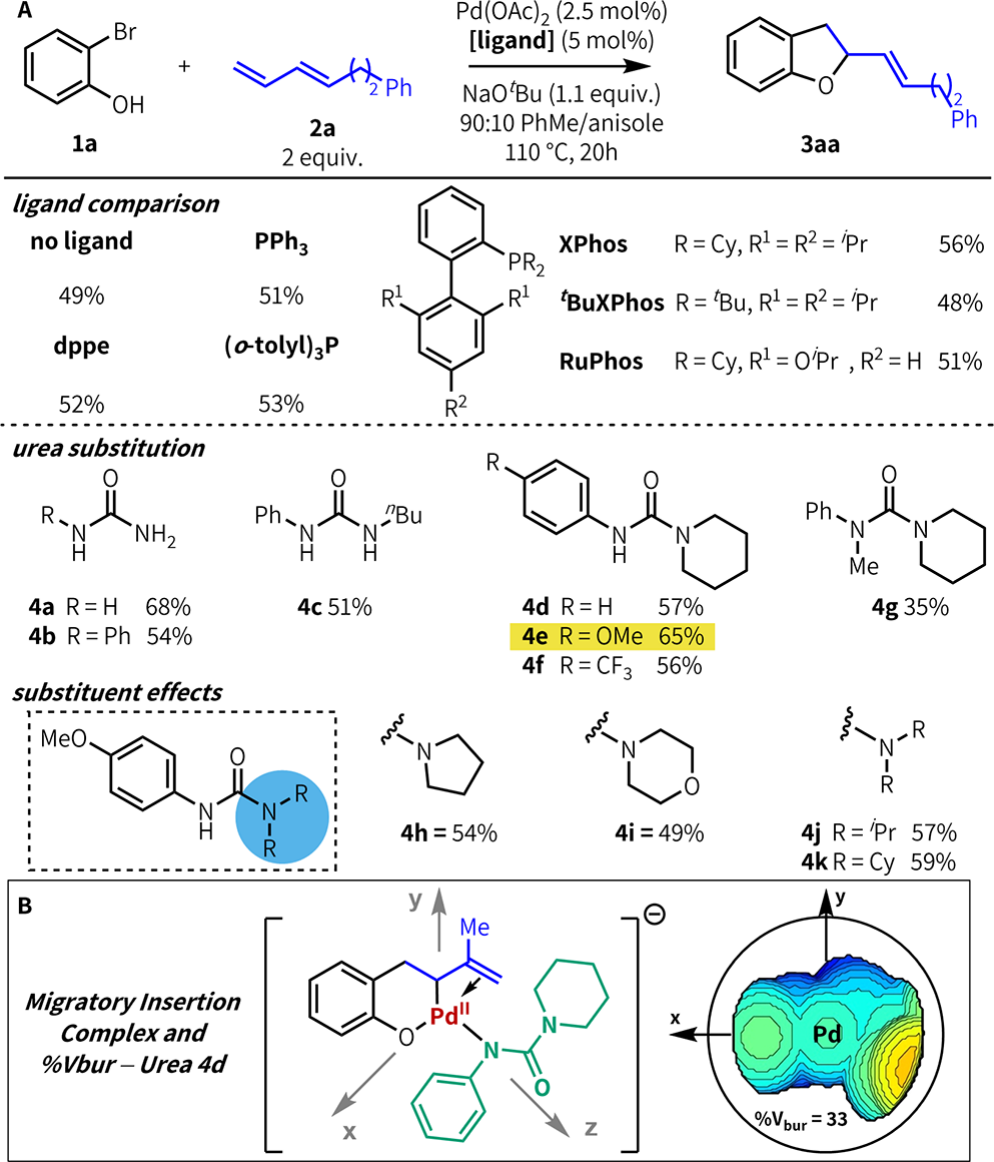
(A) Ligand structure–reactivity relationships in Pd-catalyzed heteroannulation of **1a** and diene **2a**. (B) Steric profile of trisubstituted urea **4d** (*V*_bur_ = buried volume).

To better understand the binding properties of trisubstituted ureas, we conducted DFT studies on model complexes. To discern the preferred urea binding mode, we modeled a PdCl_2_ (ureate) complex using urea **4d.**(16,19) As with monosubstituted ureas, coordination through N is significantly favored over O-coordination (−11.4 kcal/mol).^15,19e^ We then compared the buried volume (%*V*_bur_) of trisubstituted ureas with monosubstituted ureas. These *V*_bur_ calculations were performed on a post-migratory insertion Pd complex using bromophenol, isoprene, and one bound urea ligand (Figure 2b). Although **4d** (Figure 2b) is considerably larger than **4b** in any conformation (%V_bur_ = 33 vs 17),^15,17^ it is concentrated in one region of the complex rather than equally distributed as seen in phosphine ligand complexes.^16,19d^ This steric profile leaves much of the active site open and thus minimizes repulsive interactions between the ligand and other groups on the metal. These calculated properties are consistent with our previous findings.^15^

We next explored the bromophenol scope (Figure 3). Under our optimized conditions, diene **2b** and bromophenol **1a** coupled to afford **3ab** in 74% yield (0.5 mmol) and 64% yield at gram scale. Substrates bearing alkyl substitution were effective regardless of the substituent position, even when adjacent to either the bromide or phenol (**3bb**, **3eb**). Substitution adjacent to the oxygen ring is common in DHB-based natural bioactive molecules,^1,2^ and previous methods have not tolerated these substitution patterns.^13d,13e^ No clear electronic trend was observed for substitution para to the bromide (**3fb–gb**). When para to the phenol, electron-donating (**3hb**) and weakly withdrawing substituents (**3jb**) were compatible, but no product was observed with substrates bearing strongly withdrawing substituents such as CF_3_ (**3ib**). Bromophenols bearing halogen substituents such as fluorine afforded product in good yields (**3kb**); likewise, esters are well tolerated (**3mb**, 65%). Nitrogen functionality is attractive but is a challenging substrate in this type of reaction because of the propensity for nitrogen to competitively coordinate palladium. We observed modest reactivity with substrates containing tertiary amines (**3lb**, 34%), and good reactivity with a pyridinol-based substrate (**3nb**, 65%). Ketones, aldehydes, amides, nitrile, and nitro groups are not well tolerated; this is due to electron-withdrawing effects in some cases and poor solubility in others.^16^

**Figure 3:**
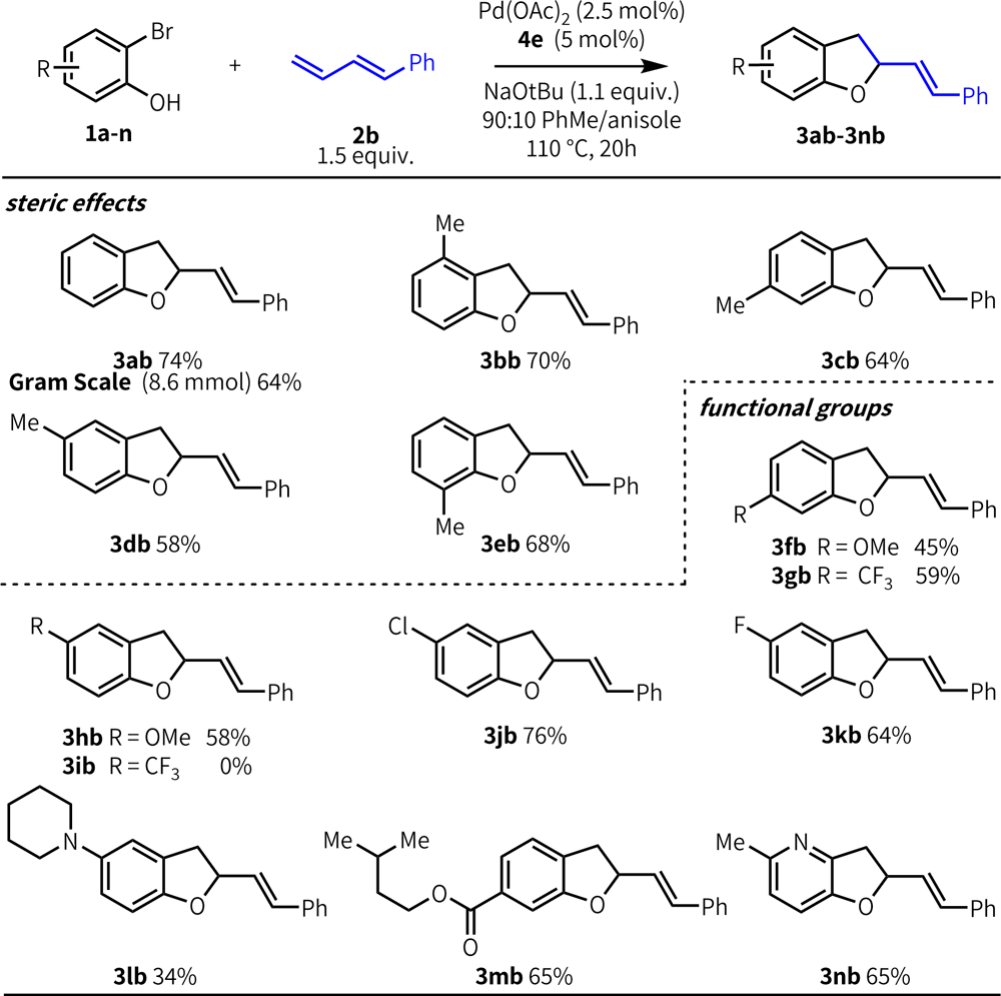
Bromophenol scope. Yields and product ratios correspond to isolated products, and are an average of three runs at 0.5 mmol scale and two runs at gram scale. Conditions: **1** (0.5 mmol), **2b** (0.75 mmol), Pd(OAc)_2_ (2.5 mol %), **4e** (5 mol %), NaO^*t*^Bu (0.55 mmol), 90:10 PhMe/anisole (0.25M), 110 °C, 24 h.

Our heteroannulation also engages structurally and functionally diverse dienes (Figure 4), with only a slight excess of diene required (1.5 equiv. vs 3–5 equiv.).^13d,13f^ Linear conjugated dienes bearing both electron-rich and electron-poor aryl substitution give products **3ac–d** in 72% and 70% yield, respectively. Unprotected and benzoate-protected primary alcohols were compatible with this methodology (**3ae–af**). Dienes with sensitive functionality such as a furan ring reacted smoothly, affording product in 64% yield (**3ah**). Various heterocycles including thiophene and phthalimide are also effective (**3ag, 3ai**). Additionally, branched dienes (**2j**–**l**), including those with sensitive functional groups (**2k**, **2l**) were good coupling partners in our methodology. Although a single product was observed with linear dienes, branched dienes gave a mixture of regioisomers with good selectivity (**3/3′** ∼ 85:15). In contrast to linear dienes, a singular phosphine ligand enhanced reactivity with myrcene (**3aj**), but it provided no advantage over urea **4e**. Sterically encumbered dienes effectively engaged in the reaction, affording **3am** and **3an** in good yield. The inclusion of the 1,2-disubstituted diene in **3am** allows for generation of a fully substituted carbon at the 2-position of the dihydrofuran ring.

**Figure 4:**
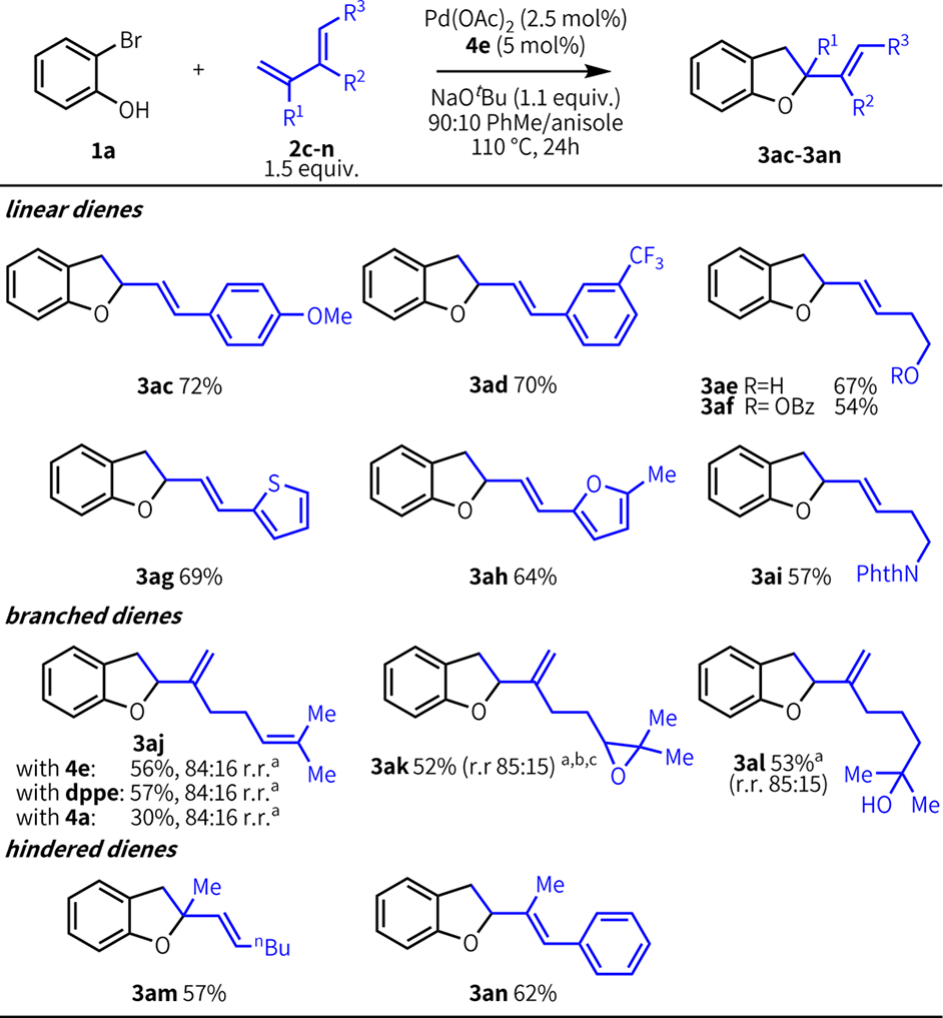
Diene scope. Yields and product ratios correspond to isolated products and are an average of three runs. Conditions: **1a** (0.5 mmol), **2** (0.75 mmol), Pd(OAc)_2_ (2.5 mol %), **4e** (5 mol %), NaO^*t*^Bu (0.55 mmol), 90:10 PhMe/anisole (0.25 M), 110 °C, 24 h. Legend: (a) 2.0 equiv. of diene used. (b) Reaction ran for 48 h. (c) Inseparable mixture of diastereomers.

We have shown that urea-enabled, palladium-catalyzed heteroannulation can be applied to the synthesis of functionalized DHBs. In contrast to existing methods, our method engages structurally diverse dienes under a unified set of reaction conditions, with broad functional group tolerance. Moreover, this chemistry can be performed with low reagent loadings and is robust to ambient conditions, making this an attractive approach for the synthesis of these core structures. Current efforts in our lab are focused on better understanding the impact that nucleophile identity has on ligand requirements for these reactions, as well as continuing to expand this methodology for the preparation of diverse heterocyclic scaffolds.

## Supporting Information Available

Additional experimental details, experimental procedures, computational studies, compound characterization, NMR spectra for all new compounds (PDF)
