# Supplementary material for: Convergent Synthesis of Dihydrobenzofurans via Urea Ligand-Enabled Heteroannulation of 2-Bromophenols with 1,3-Dienes
Source: Org Lett. 2022 Jul 29;24(31):5787–90. doi: 10.1021/acs.orglett.2c02301 (PMC9380016; doi:10.1021/acs.orglett.2c02301)
Supplement: Supplementary file 1 — ol2c02301_si_001.pdf [file ol2c02301_si_001.pdf]

Convergent Synthesis of Dihydrobenzofurans Via Urea Ligand-enabled Heteroannulation of 2-Bromophenols with 1,3-Dienes

Kaitlyn E. Houghtling, Amanda M. Canfield, Shauna M. Paradine*

Department of Chemistry, University of Rochester, 414 Hutchison Hall, 120 Trustee Road, Rochester, NY 14627 (USA).

*Corresponding Author: sparadin@ur.rochester.edu

Supporting Information

Contents

General Remarks 2
Methods 2
Materials and Reagents 2
Instrumentation 2
Abbreviations Used 3
Summary of Bromophenols Used 3
Preparation of Bromophenol Reagents 4
Summary of 1,3-Dienes Used 7
Preparation of 1,3-Dienes 8
Preparation of Urea Ligands 10
Ligand Studies 15
XPHOS Palladium Binding and Oxidative Addition Derivative Studies 22
Ligand Screen with Myrcene as More Challenging Olefin 26
Reaction Condition Optimization 29
Reaction Tolerance 35
Gram Scale Reaction 36
Bromophenol Scope 37
1,3-Diene Scope 46
Poorly Reactive Substrates 55

Computational studies 55
References Cited 61
NMR Spectra of New Compounds 63

General Remarks

Methods

All reactions were carried out under a nitrogen atmosphere in flame–dried glassware with magnetic stir bar unless otherwise specified. Stainless steel gas-tight syringes were used to transfer air- and moisture-sensitive liquids. Reactions were monitored by thin–layer chromatography (TLC) on pre–coated silica gel 60 F254 glass–supported plates from EMD, and visualized under UV light (254 nm) or with p–anisaldehyde followed by heating. Flash chromatography was performed using SiliaFlash P60 (230–400 mesh, SiliCycle). Reported product yields were determined based on material isolated after column purification. Room temperature (rt) for the laboratory is 20°C.

Materials and Reagents

Reagents were used as obtained from commercial suppliers without further purification. Tetrahydrofuran (THF), diethyl ether (Et_2O), methylene chloride (DCM), were purchased from Fisher and dispensed using the Pure Process Technology solvent purification system. ACS grade hexanes, toluene (PhMe), ethyl acetate, DCM, pentane and diethyl ether were used for column chromatography. Thin-layer chromatography (TLC) was performed on pre-coated silica gel 60 F254 glass-supported plates from EMD, and visualization was performed with a UV lamp followed by staining with p-anisaldehyde followed by heating. Silica gel chromatography purifications were carried out using SiliCycle Silica SiliaFlash P60 230-400 mesh.

Instrumentation

Proton nuclear magnetic resonance (1H NMR) and proton-decoupled carbon nuclear magnetic resonance (13C, 1H NMR) spectra were recorded on a Bruker DPX–400 instrument (operating at 400 MHz for 1H, 100 MHz for 13C) or a Bruker DPX–500 instrument (operating at 500 MHz for 1H, 125 MHz for 13C) at room temperature. Proton resonances are referenced to residual protium in the NMR solvent. Carbon resonances are referenced to the carbon resonances of the NMR solvent. Data are represented as follows: chemical shift, multiplicity (br = broad, s = singlet, d = doublet, dd = doublet of doublets, t = triplet, q = quartet, m = multiplet, app = apparent), coupling constants (J) in Hertz (Hz), integration. Mass S3 spectral (MS) data were obtained on a Thermo Fisher Q Exactive Plus spectrometer (University of Rochester Medical Center Mass Spectrometry Resource Laboratory). Elemental analysis samples were weighed using a PerkinElmer Model AD6000 Autobalance, and their compositions were determined with a PerkinElmer 2400 Series II Analyzer at the Analysis Facility at the University of Rochester, funded by NSF CHE-0650456. Gas chromatography was performed using a Shimadzu GC-2030 instrument with Shimadzu AOC 20i plus autosampler. All samples were injected from a

diethyl ether sample at 40°C ramped to 275°C. 275°C was held for 5 min and total sample run time was 22 min.

Abbreviations Used

aq. = aqueous, DCM = dichloromethane, DMF = N,N-dimethylformamide, DMSO = dimethylsulfoxide, equiv. = equivalents, h = hours, min = minutes, r.r. = regioisomeric ratio, rt = room temperature (20°C), sat = saturated, THF = tetrahydrofuran, TBS = tertbutyldimethylsilyl. PheMe = toluene

Summary of Bromophenols Used

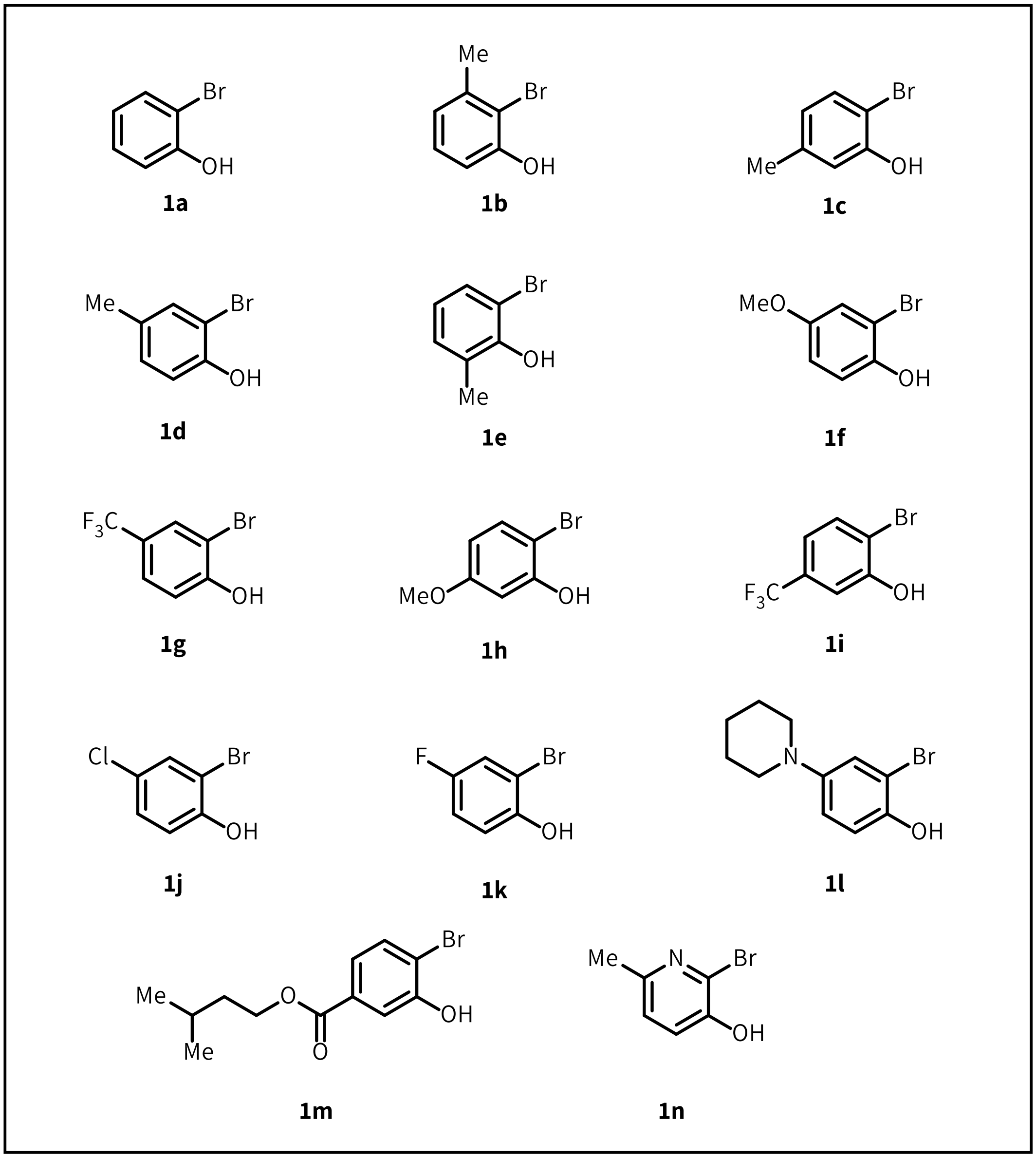

All bromophenols listed above as well as starting materials for compounds **1l** and **1m** were purchased from Oakwood Chemical and Combi-Blocks.

Preparation of Bromophenol Reagents

Preparation of 2-bromo-4-(piperidin-1-yl) phenol

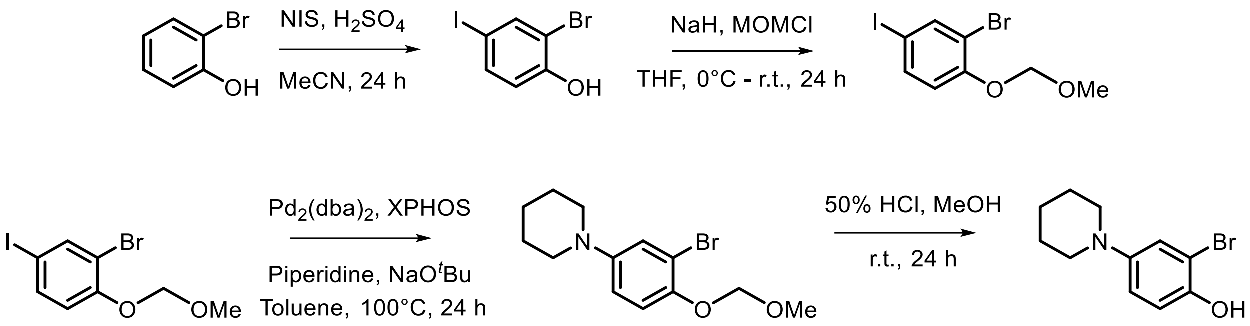

2-bromo-4-iodophenol

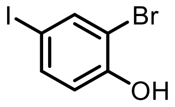

Prepared according to modified literature procedure.[1] 2-bromophenol (3.20 mL, 4.80 g, 27.7 mmol, 1.0 equiv.) was dissolved in MeCN (100 mL, 0.28 M) and H_2SO_4 (1.63 mL, 9.20 mmol, 0.33 equiv.) was added and allowed to stir at rt for 5 min. NIS (6.83 g, 28.4 mmol, 1.0 equiv.) was added portion-wise over 30 min at rt. The reaction was then allowed to run at rt overnight. Sodium thiosulfate (30 mL) was added to quench the reaction and the resulting mixture was extracted with EtOAc (3 x 50 mL). The resulting organic layer was washed with brine (50 mL), dried with $MgSO_4$, and concentrated in vacuo. The orange crude solid was purified via column chromatography with hexanes → 30% EtOAc/70% hexanes, affording the desired product as a white crystalline solid (7.66 g, 25.6 mmol, 93%).

^{1}H NMR (500 MHz, $CDCl_3$) δ 7.75 (d, *J* = 2.1 Hz, 1H), 7.49 (dd, *J* = 8.6, 2.0 Hz, 1H), 6.78 (d, *J* = 8.5 Hz, 1H), 5.58 (b, 1H). Spectral data agree with that reported in literature. [1]

2-bromo-4-iodo-1-(methoxymethoxy)benzene

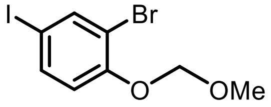

Prepared according to modified literature procedure.[2] 2-bromo-4-iodophenol (5.00 g, 16.7 mmol, 1.0 equiv.) was dissolved in THF (250 mL, 0.07 M) and cooled to 0°C. NaH (442 mg, 18.4 mmol, 1.1 equiv.) was slowly added to the solution and allowed to stir at 0°C for 1 hour. Chloromethyl methyl ether (1.40 mL, 18.4 mmol, 1.1 equiv.) was added dropwise and the reaction was allowed to come to rt and stir overnight. The reaction was quenched with ammonium chloride (75 mL) and diluted with EtOAc (100 mL). The resulting organic layer was washed with H_2O (2 x 75 mL), brine (75 mL), and dried with $MgSO_4$ before concentrating in vacuo. The resulting oil was purified via silica plug with 30% EtOAc/70% hexanes to afford a cloudy oil as the pure product in quantitative yield.

^{1}H NMR (500 MHz, $CDCl_3$) δ 7.79 (d, *J* = 2.2 Hz, 1H), 7.44 (dd, *J* = 8.7, 2.2 Hz, 1H), 6.83 (d, *J* = 8.7 Hz, 1H), 5.15 (s, 2H), 3.43 (s, 3H). Spectra data agree with that reported in literature.[3]

^{13}C NMR (126 MHz, $CDCl_3$) δ 153.6, 140.9, 137.2, 117.7, 114.0, 94.9, 84.5, 56.4.

Anal. Calcd for $C_8H_8BrIO_2$: C, 28.02; H, 2.35. Found: C, 27.88; H, 2.37.

1-(3-bromo-4-(methoxymethoxy)phenyl)piperidine

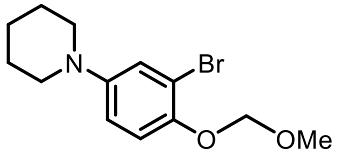

Prepared according to modified literature procedure.[4] A 2 dram vial was flame dried and purged with argon before being charged with $Pd_2(dba)_2$ (33.0 mg, 0.036 mmol, 20.0 mol%), XPhos (34.0 mg, 0.071 mmol, 40.0 mol%) and anhydrous PhMe (1.00 mL, 1.75 M). The metal/ligand mixture was allowed to stir for 20 min. 2-bromo-4-iodo-1-(methoxymethoxy)benzene (132 mg, 1.75 mmol, 1.0 equiv.), piperidine (0.17 mL, 1.75 mmol, 1.0 equiv.) and NaO*t*Bu (104 mg, 1.08 mmol, 1.1 equiv.) were added and the reaction was stirred at 100°C in a silicone oil bath for 24 h. The reaction mixture was poured over a celite plug to quench and filtered with EtOAc before being concentrated in vacuo. The resulting brown oil was purified via column chromatography with hexanes → 15% EtOAc/85% hexanes to afford the desired product as an amber oil (284 mg, 0.945 mmol, 54%).

^{1}H NMR (400 MHz, $CDCl_3$) δ 7.12 (d, *J* = 2.9 Hz, 1H), 7.04 (d, *J* = 9.0 Hz, 1H), 6.82 (dd, *J* = 9.0, 2.9 Hz, 1H), 5.15 (s, 2H), 3.52 (s, 3H), 3.08 – 3.01 (m, 4H), 1.70 (m, 4H), 1.55 (m, 2H).

^{13}C NMR (126 MHz, $CDCl_3$) δ 149.5, 147.7, 122.4, 118.5, 117.8, 114.5, 96.8, 57.1, 52.1 (2C), 26.4 (2C), 24.8.

HRMS (ESI) m/z calculated for $C_{13}H_{18}BrNO_2$ $[M+H]^+$: 300.0588, found 300.0593.

2-bromo-4-(piperidin-1-yl) phenol (1l)

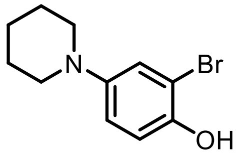

Prepared according to modified literature procedure.[5] 1-(3-bromo-4-(methoxymethoxy)phenyl)piperidine (0.600 g, 2.34 mmol, 1.0 equiv.) was added to MeOH (5.00 mL, 0.47 M) and 50% HCl (2.00 mL) was added slowly to the solution. The reaction was stirred vigorously for 24 h at rt. The reaction was diluted with H_2O (3.00 mL) and saturated $NaHCO_3$ was added slowly until pH 8 was achieved. The solution was extracted with EtOAc (3 x 5 mL), dried with $MgSO_4$, and concentrated in vacuo. The resulting brown oil was purified via column chromatography with hexanes → 25% EtOAc/75% hexanes to afford the desired product as an off-white solid in quantitative yield.

^{1}H NMR (400 MHz, $CDCl_3$) δ 7.04 (d, J = 2.8 Hz, 1H), 6.90 - 6.82 (m, 2H), 5.33 (s, 1H), 3.04 – 2.96 (m, 4H), 1.70 (m, 4H), 1.59 – 1.48 (m, 2H).

^{13}C NMR (126 MHz, $CDCl_3$) δ 147.8, 146.3, 122.9, 119.4, 116.5, 110.6, 52.4 (2C), 26.2 (2C), 24.3.

HRMS (ESI) m/z calculated for $C_{11}H_{14}BrNO$ $[M+H]^+$: 256.0337, found 256.0330.

Synthesis of bromophenol 1m

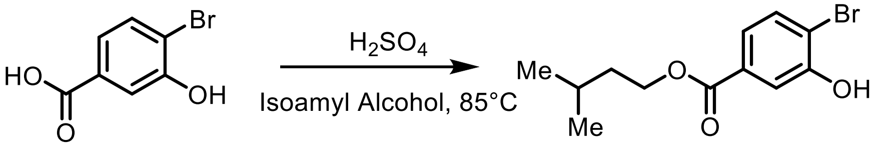

Isopentyl 4-bromo-3-hydroxybenzoate (1m)

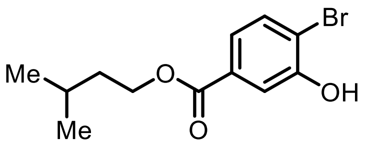

Prepared according to modified literature procedure.[6] 4-bromo-3-hydroxybenzoic acid (1.00 g, 4.61 mmol, 1.0 equiv.) was dissolved in isoamyl alcohol (15.0 mL, 138 mmol, 0.30 M). H_2SO_4 (0.200 mL, 1.11 mmol, 0.24 equiv.) was added and allowed to stir at 85°C in a silicone oil bath overnight. Upon completion, the reaction was then diluted with H_2O (10 mL) and EtOAc (30 mL), extracted with H_2O (3 X 15 mL), washed with brine (15 mL), and dried with $MgSO_4$. After concentration in vacuo, the resulting crude oil was purified via SiO_2 plug using 30% EtOAc/ 70% hexanes to afford a white powder as the desired product in quantitative yield.

^1H NMR (500 MHz, $CDCl_3$) δ 7.69 (d, J = 2.0 Hz, 1H), 7.51 (d, J = 8.3 Hz, 1H), 7.44 (dd, J = 8.3, 2.0 Hz, 1H), 6.52 (s, 1H), 4.33 (t, J = 8.0 Hz, 2H), 1.75 (m, 1H), 1.63 (dt, J = 10.0 Hz, J = 8.0 Hz, 2H), 0.94 (s, 3H), 0.93 (s, 3H).

^{13}C NMR (126 MHz, $CDCl_3$) δ 166.5, 153.0, 132.7, 131.4, 122.8, 117.4, 115.9, 64.5, 37.5, 25.4, 23.4 (2C).

HRMS (ESI) m/z calculated for $C_{12}H_{15}BrO_3$ $[M+H]^+$: 287.0283, found 287.0275.

Summary of 1,3-Dienes Used

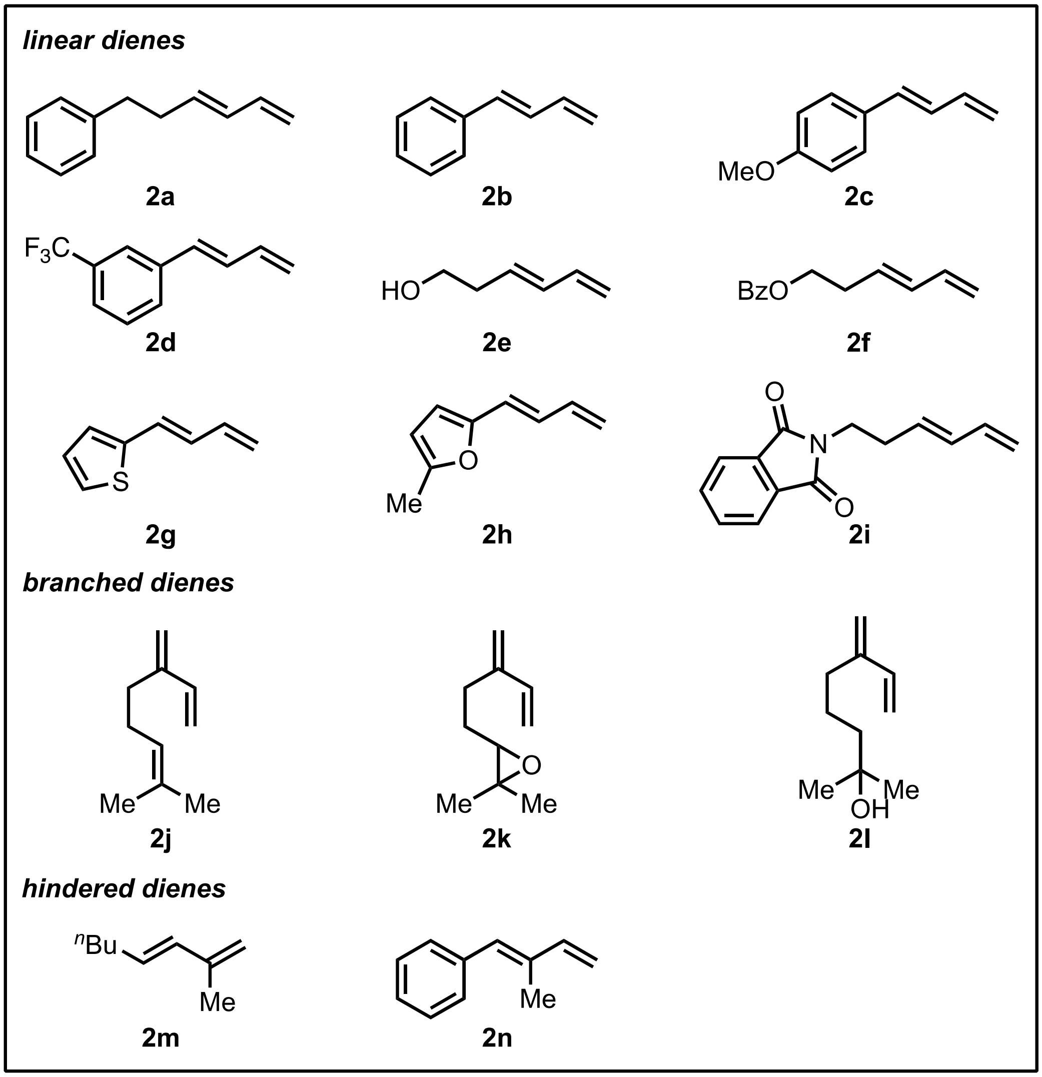

Substrates **2b-2e**, **2g**, **2i**, **2k, 2m-2n** were synthesized previously by our lab.[7] Substrate **2j** was purchased from Sigma-Aldrich.

Preparation of 1,3-Dienes

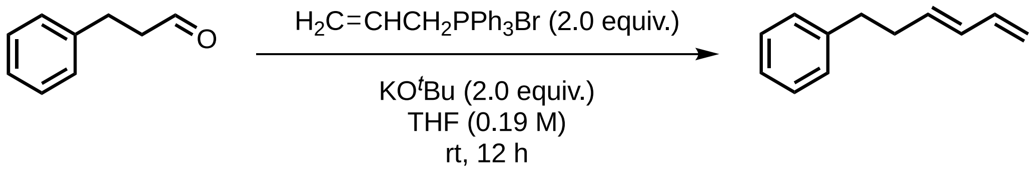

(*E*)-hexa-3,5-dien-1-ylbenzene (2a)

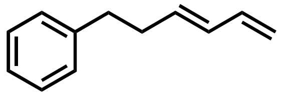

Prepared according to modified literature procedure.[8] Allyl triphenylphosphonium bromide (16.2 g, 42.3 mmol, 2.0 equiv.) was measured into an oven-dried round bottom flask equipped with a stir bar and septum. The flask was evacuated and refilled with nitrogen three times before the addition of dry THF (112 mL, 0.19 M). KO*t*Bu (4.75 g, 42.3 mmol, 2.0 equiv.) was added the mixture was stirred at rt (20°C) for 30 min. 3-phenylpropanal (2.79 mL, 21.2 mmol, 1.0 equiv.) was added dropwise at rt and the reaction mixture was stirred for 12 hours. Upon completion, the solution was quenched with NH_4Cl (3 x 40 mL). The organic layer was extracted and washed with brine (3 X 40 mL), then dried over $MgSO_4$. Solvent was removed under reduced pressure. Crude material was purified via column chromatography on SiO_2 using 100% pentane. Product was isolated as a colorless oil (1.09 g, 6.89 mmol, 33%). Material was used immediately as it decomposes over time.

^{1}H NMR (400 MHz, $CDCl_3$) δ 7.35 – 7.13 (m, 5H), 6.68 – 6.23 (m, 1H), 6.15 – 5.97 (m, 1H), 5.82 – 5.43 (m, 1H), 5.24 – 4.94 (m, 2H), 2.76 – 2.65 (m, 2H), 2.57 – 2.35 (m, 2H). Spectral data agree with that reported in literature.[8]

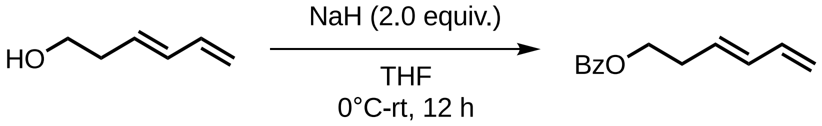

(*E*)-hexa-3,5-dien-1-yl benzoate (2f)

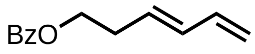

Prepared according to modified literature procedure.[9] NaH (628 mg, 15.7 mmol, 2.0 equiv.) was added to an oven-dried round bottom flask equipped with a stir bar and septum under nitrogen. The flask was cooled to 0°C and THF (17.0 mL, 0.92 M) was added. (*E*)-hexa-3,5-dien-1-ol (771 mg, 7.85 mmol, 1.0 equiv.) in THF (4.00 mL, 2.0 M) was added and the reaction mixture was left to stir for 30 min at 0°C. Benzoyl chloride (1.37 mL, 11.8 mmol, 1.5 equiv.) was added dropwise at 0°C and the reaction mixture was warmed up to rt (20°C) and left to stir for 12 hours. Upon completion, the reaction mixture was quenched with H_2O (10.0 mL) and the aqueous layer was separated and extracted with Et_2O (2 X 20 mL), then dried over $MgSO_4$. Crude material was purified via column chromatography on SiO_2 using pentane → 95% pentane/5% Et_2O. Pure product was isolated as a colorless oil (1.09 g, 5.37 mmol, 68%).

^1H NMR (400 MHz, $CDCl_3$) δ 8.09 – 8.00 (m, 2H), 7.60 – 7.51 (m, 1H), 7.48 – 7.40 (m, 2H), 6.34 (dt, *J* = 16.9, 10.2 Hz, 1H), 6.19 (dd, *J* = 15.2, 10.4 Hz, 1H), 5.75 (dt, *J* = 14.7, 7.0 Hz, 1H), 5.15 (d, *J* = 15.2, 1H), 5.09 – 4.99 (m, 1H), 4.41 – 4.28 (m, 2H), 2.57 (dt, *J* = 6.8, 6.8 Hz, 2H). Spectral data agree with that reported in literature.[9]

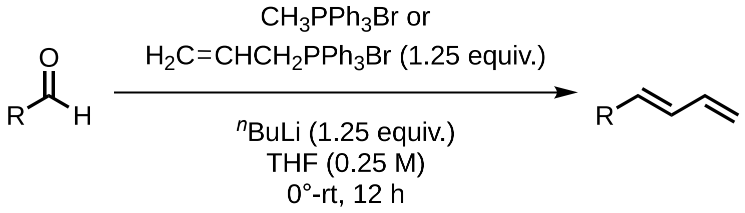

General Procedure for 1,3-diene Preparation from Aldehydes: Prepared according to a modified literature procedure.[10] Methyl triphenylphosphonium bromide or allyl triphenylphosphonium bromide (1.3 equiv.) was measured into an oven-dried round bottom flask equipped with a stir bar and septum. The flask was evacuated and refilled with nitrogen three times before the addition of dry THF (0.25M). *n*–butyllithium (1.3 equiv., 2.5 M in hexanes) was added dropwise to the suspension of the Wittig reagent at 0°C and the mixture was stirred at 0°C for 1 hour. Aldehyde (1.0 equiv.) was added dropwise at 0°C and the reaction mixture was warmed up to rt (20°C). The reaction mixture was stirred for 12 hours. Upon completion, the solution was quenched with NH_4Cl (3 X 40 mL). The organic layer was extracted and washed with brine (3 X 40 mL), then dried over $MgSO_4$. Solvent was removed under reduced pressure; then crude residue was purified via column chromatography on SiO_2. Purified dienes were stored at –28°C for no longer than one week.

(*E*)-2-(buta-1,3-dien-1-yl)-5-methylfuran (2h)

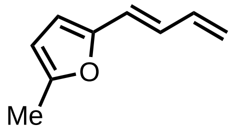

Prepared according to general procedure. Allyl triphenylphosphonium bromide (9.58 g, 25.0 mmol, 1.3 equiv.), THF (80.0 mL, 0.25M), *n*–butyllithium (25.0 mL, 10.0 mmol, 1.3 equiv., 2.5 M in hexanes), 5-methylfuran-2-carbaldehyde (2.20 g, 20.0 mmol, 1.0 equiv.) were used. Crude material was purified via column chromatography on SiO_2 using 100% pentane. Product was isolated as light-yellow liquid in a 2:1 mixture of *E*/*Z* isomers. (632 mg, 4.71 mmol, 24%). Material was used immediately as it decomposes over time.

E isomer: ^1H NMR (500 MHz, $CDCl_3$) δ 7.35 (ddd, *J* = 16.8, 10.2, 10.2 Hz, 1H), 6.64 (dd, *J* = 15.6, 10.8 Hz, 1H) 6.29 (d, *J* = 15.6 Hz, 1H), 6.21 (d, *J* = 3.2 Hz, 1H), 6.05 – 6.00 (m, 1H), 5.35 – 5.30 (m, 1H), 5.23 (dd, *J* = 9.9, 1.2 Hz, 1H), 2.34, (s, 3H).

Z isomer: ^1H NMR (500 MHz, $CDCl_3$) δ 6.43 (ddd, *J* = 17.0, 10.5, 10.5 Hz, 1H), 6.16 (d, *J* = 3.2 Hz, 1H), 6.00 – 5.94 (m, 3H), 5.30 – 5.26 (m, 1H), 5.11 (dd, *J* = 9.9, 1.5 Hz, 1H), 2.32 (s, 3H).

E isomer: ^{13}C NMR (126 MHz, $CDCl_3$) δ 152.2, 134.7, 126.4, 120.8, 119.0, 117.3, 112.0, 107.6, 14.0.

Z isomer: ^{13}C NMR (126 MHz, $CDCl_3$) δ 152.7, 137.1, 126.8, 120.8, 119.0, 117.0, 110.0, 107.9, 13.9.

HRMS (ESI) *m/z* calculated for $C_9H_{10}O$ $[M+H]^+$: 135.0810, found 135.0803.

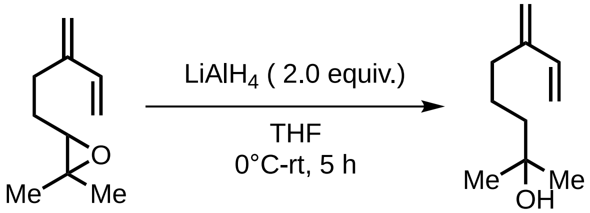

2-methyl-6-methyleneoct-7-en-2-ol (2l)

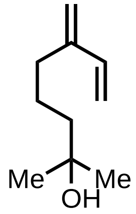

Prepared according to modified literature procedure.[11] $LiAlH_4$ (499 mg, 13.1 mmol, 2.0 equiv.) was added to an oven-dried round bottom flask equipped with a stir bar and septum under nitrogen. The flask was cooled to 0°C and THF (5.00 mL, 2.6 M) was added. 6,7-epoxymyrcene **2** (1.00 g, 6.57 mmol, 1.0 *equiv.)* in THF (1.00 mL, 6.6 M) was added dropwise at 0°C. The solution was warmed up to rt (20°C) and left to stir for 5 hours. Upon completion, the reaction mixture was cooled to 0°C and quenched by dropwise addition of H_2O (5.00 mL), 10 wt % aq. NaOH (5.00 mL), and H_2O (15.0 mL), in sequential order. The reaction mixture was extracted with DCM (3 X 30 mL). The combined organic layers were washed with H_2O (30 mL) and brine (30 mL), then dried over $MgSO_4$. Crude material was purified via column chromatography on SiO_2 using 90% pentane/10% Et_2O → 50% pentane/50% Et_2O. Pure product was isolated as a colorless oil (649 mg, 4.21 mmol, 64%).

^1H NMR (400 MHz, $CDCl_3$) δ 6.37 (ddd, *J* = 17.8, 10.7, 3.5 Hz, 1H), 5.22 (dd, *J* = 17.7, 3.6 Hz, 1H), 5.09 – 4.97 (m, 3H), 2.26 – 2.17 (m, 2H), 1.63 – 1.53 (m, 2H), 1.53 – 1.44 (m, 2H), 1.21 (s, 6H). Spectral data agree with that reported in literature.[12]

Preparation of Urea Ligands

General procedure for preparation of monosubstituted ureas from amines: Prepared according to literature procedure.[7] To a solution of corresponding primary or secondary amine (1.0 equiv.) in water/glacial acetic acid (1:1, ca. 0.25M) was added potassium cyanate (1.5 or 3.0 equiv.). The resulting suspension was stirred for 18 h and then quenched with ice. The resulting precipitate was collected by filtration and washed with water to afford the crude product. The crude product was recrystallized in refluxing ethanol/hexanes to give the desired urea.

1-(4-methoxyphenyl) urea (SI L1)

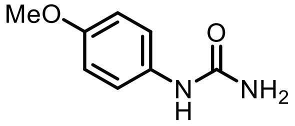

Prepared according to literature procedure.[7] 4-Methoxyaniline (1.00 g, 8.10 mmol, 1.0 equiv.), water/glacial acetic acid (1:1, 30.0 mL, 0.27 M), and potassium cyanate (1.97 g, 24.3 mmol, 3.0

equiv.) were used. The recrystallized product was obtained as an off-white solid (713 mg, 4.29 mmol, 53%).

1H NMR (400 MHz, DMSO-d6) δ 7.21 (d, J = 8.8 Hz, 2H), 6.82 (d, J = 8.8 Hz, 2H), 3.73 (s, 3H). Spectral data agree with that reported in the literature. [7]

1-(4-methoxy-2-methylphenyl) urea (SI L2)

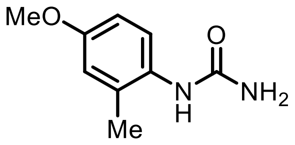

Prepared according to literature procedure.[7] 4-Methoxy-2-methylaniline (1.04 g, 7.32 mmol, 1.0 equiv.), water/glacial acetic acid (1:1, 30.0 mL, 0.24 M), and potassium cyanate (1.78 g, 22.0 mmol, 3.0 equiv.) were used. The recrystallized product was obtained as an off-white solid (1.20 g, 6.66 mmol, 91%).

1H NMR (400 MHz, DMSO-d6) δ 7.76 (m, 1H), 7.70 (s, 1H), 7.11–7.03 (m, 2H), 6.87 (m, 1H), 6.01 (s, 2H), 2.17 (s, 3H). Spectral data agree with that reported in the literature.[7]

1-butyl-3-phenylurea (4c)

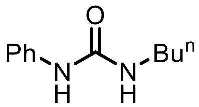

Prepared according to literature procedure.[7] To a solution of phenyl isocyanate (1.00 mL, 9.16 mmol, 1.0 equiv.) in hexanes (40.0 mL, 0.23 M), *n*–butylamine (0.910 mL, 9.16 mmol, 1.0 equiv.). The immediately formed white suspension was stirred vigorously for 10 min, filtered, and washed with hexanes (2 X 100 mL) to afford the product as a white solid (1.62 g, 8.42 mmol, 92%).

^{1}H NMR (400 MHz, DMSO-d6) δ 8.36 (s, 1H), 7.38 (d, J = 7.6 Hz, 2H), 7.20 (dd, J = 7.6, 7.2 Hz, 2H), 6.87 (t, J = 7.2 Hz, 1H), 6.01 (t, J = 5.6 Hz, 1H), 3.08 (q, J = 5.6 Hz, 2H), 1.44–1.26 (m, 4H), 0.89 (t, J = 7.2 Hz, 3H). Spectral data agree with that reported in the literature.[7]

***N*-methyl-*N*-phenylpiperidine-1-carboxamide (4g)**

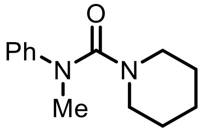

N-phenylpiperidine-1-carboxamide (0.250 g, 1.10 mmol, 1.0 equiv.) was dissolved in DMF (5.00 mL, 0.22 M). NaH (30.0 mg, 1.3 mmol, 1.1 equiv.) was added at 0°C. The reaction was allowed to stir for 20 min. Methyl iodide (0.070 mL, 1.10 mmol, 1.0 equiv.) was added dropwise at 0°C. The resulting mixture warmed to rt and stirred overnight. The reaction was quenched with 5.00 mL of saturated sodium thiosulfate was added and extracted with 5.00 mL EtOAc (3 X 5.00 mL). The combined organic layers were then washed copiously with water to remove DMF, dried with $MgSO_4$, and concentrated in vacuo. The resulting orange oil was purified via column chromatography with hexanes → 1:1 EtOAc/hexanes to afford the pure desired ligand as a pale-yellow oil (168 mg, 0.770 mmol, 70%).

^{1}H NMR (400 MHz, Chloroform-*d*) δ 7.28 – 7.19 (m, 2H), 7.05 – 6.96 (m, 3H), 3.13 (s, 3H), 3.11 – 3.06 (m, 4H), 1.39 (m, 2H), 1.32 – 1.21 (m, 4H).

^{13}C NMR (101 MHz, DMSO) δ 155.2, 154.4, 133.8, 121.6 (2C), 113.5 (2C), 55.1, 44.6 (2C), 25.6 (2C), 24.2.

HRMS (ESI) *m/z* calculated for $C_{13}H_{18}N_2O$ $[M+H]^+$: 219.1497, found 219.1492

Preparation of Trisubstituted Ureas Synthesized Using Phenyl Isocyanates

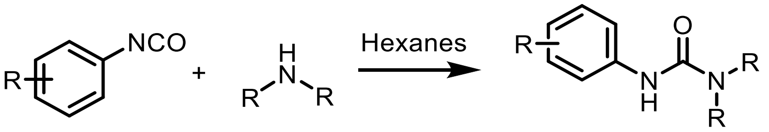

General procedure for preparation of urea ligands from isocyanate and amine precursors: To an oven-dried round bottom flask was added hexanes (50.0 mL) at rt. Phenyl isocyanate (1.0 equiv.) and relevant amine (3.0 equiv.) were added dropwise while stirring. Upon completion (~20 min), the resulting urea crashed out in solution, which was filtered and rinsed with hexanes resulting in the pure ligand as a white powder in quantitative yield.

***N*-phenylpiperidine-1-carboxamide (4d)**

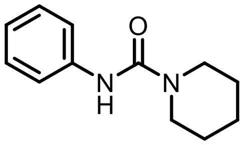

Prepared according to the general procedure. Phenyl isocyanate (0.842 mL, 7.70 mmol, 1.0 equiv.) was added to hexanes (50.0 mL, 0.15 M). Piperidine (2.30 mL, 23.1 mmol, 3.0 equiv.) was then added dropwise. The reaction was allowed to stir for 20 min, and the desired urea precipitated from solution. The product was filtered and rinsed with hexanes to afford the product as a white solid in quantitative yield. No further purification was required.

^{1}H NMR (400 MHz, DMSO-d_6) δ 8.45 (s, 1H), 7.49 (dd, J = 8.8 Hz, 8 Hz, 2H), 7.25 (t, J = 7.6 Hz, 2H), 6.95 (m, 1H), 3.45 (m, 4H), 1.60 (m, 2H), 1.52 (m, 4H).

^{13}C NMR (101 MHz, DMSO) δ 155.1, 141.0, 128.4 (2C), 121.7 (2C), 119.8, 44.9 (2C), 25.7 (2C), 24.3.

HRMS (ESI) *m/z* calculated for $C_{12}H_{16}N_2O$ $[M+H]^+$: 205.1341, found 205.1335.

***N*-(4-methoxyphenyl)piperidine-1-carboxamide (4e)**

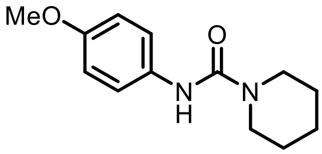

Prepared according to the general procedure. 4-methoxyphenyl isocyanate (1.00 mL, 7.70 mmol, 1.0 equiv.) was added to hexanes (50.0 mL, 0.15 M). Piperidine (2.30 mL, 23.1 mmol, 3.0 equiv.) was then added dropwise. The reaction was allowed to stir for 20 min, and the desired urea precipitated from solution. The product was filtered and rinsed with hexanes to afford the product as a white solid in quantitative yield. No further purification was required.

^{1}H NMR (400 MHz, DMSO-d_6) δ 8.28 (s, 1H), 7.36 (d, J = 9.1 Hz, 2H), 6.84 (d, J = 9.1 Hz, 2H), 3.74 (s, 3H), 3.42 (t, J = 5.4 Hz, 4H), 1.60 (m, 2H), 1.51 (m, 4H).

^{13}C NMR (101 MHz, DMSO) δ 155.2, 154.4, 133.8, 121.6 (2C), 113.5 (2C), 55.1, 44.6 (2C), 25.6 (2C), 24.2.

HRMS (ESI) *m/z* calculated for $C_{13}H_{18}N_2O_2$ $[M+H]^+$: 235.1446, found 235.1440.

***N*-(4-(trifluoromethyl)phenyl)piperidine-1-carboxamide (4f)**

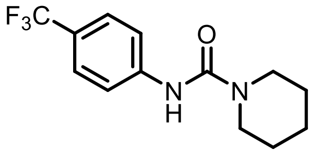

Prepared according to the general procedure. 4-(trifluormethyl) phenyl isocyanate (1.10 mL, 7.70 mmol, 1.0 equiv.) was added to hexanes (50.0 mL, 0.15 M). Piperidine (2.30 mL, 23.1 mmol, 3.0 equiv.) was then added dropwise. The reaction was allowed to stir for 20 min, and the desired urea precipitated from solution. The product was filtered and rinsed with hexanes to afford the product as a white solid in quantitative yield. No further purification was required.

^{1}H NMR (400 MHz, DMSO-d_6) δ 8.86 (s, 1H), 7.72 (d, J = 8.5 Hz, 2H), 7.58 (d, J = 8.6 Hz, 2H), 3.46 (t, J = 5.4 Hz, 4H), 1.60 (m, *2*H), 1.56 – 1.47 (m, 4H).

^{13}C NMR (101 MHz, DMSO, Fluorine not decoupled) δ 155.1, 144.7, 126.0 (q, $J_{1C\text{-}F}$ = 270.7 Hz, 1C) 125.5 (q, $J_{3C\text{-}F}$ = 4.0 Hz, 2C), 123.4, 121.5, (q, $J_{2C\text{-}F}$ = 31.7 Hz, 1C), 118.8 (2C), 48.6 (2C), 25.5 (2C), 24.1.

^{19}F NMR (376 MHz, DMSO) δ -55.4 (3F).

HRMS (ESI) *m/z* calculated for $C_{13}H_{15}F_3N_2O$ $[M+H]^+$: 273.1214, found 273.1206.

***N*-(4-methoxyphenyl)pyrrolidine-1-carboxamide (4h)**

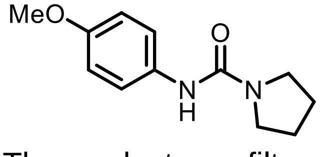

Prepared according to the general procedure. 4-methoxyphenyl isocyanate (1.00 mL, 7.70 mmol, 1.0 equiv.) was added to hexanes (50.0 mL, 0.15 M). Pyrrolidine (1.90 mL, 23.1 mmol, 3.0 equiv.) was then added dropwise. The reaction was allowed to stir for 20 min, and the desired urea precipitated from solution. The product was filtered and rinsed with hexanes to afford the product as a white solid in quantitative yield. No further purification was required.

^{1}H NMR (400 MHz, DMSO-d_6) δ 7.96 (s, 1H), 7.41 (d, J = 9.1 Hz, 2H), 6.84 (d, J = 9.2 Hz, 2H), 3.73 (s, 3H), 3.37 (m, 4H), 1.87 (m, 4H).

^{13}C NMR (101 MHz, DMSO) δ 154.4, 154.3, 133.7, 121.5 (2C), 113.5 (2C), 55.1, 45.7 (2C), 25.1 (2C).

HRMS (ESI) *m/z* calculated for $C_{12}H_{16}N_2O_2$ $[M+H]^+$: 221.1290, found 221.1283.

_N_-(4-methoxyphenyl)morpholine-4-carboxamide (4i)

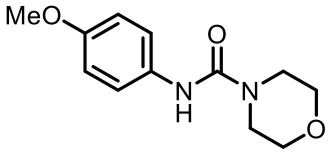

Prepared according to the general procedure. 4-methoxyphenyl isocyanate (1.00 mL, 7.70 mmol, 1.0 equiv.) was added to hexanes (50.0 mL, 0.15 M). Morpholine (2.0 mL, 23.1 mmol, 3.0 equiv.) was then added dropwise. The reaction was allowed to stir for 20 min, and the desired urea precipitated from solution. The product was filtered and rinsed with hexanes to afford the product as a white solid in quantitative yield. No further purification was required.

^{1}H NMR (400 MHz, DMSO-d_6) δ 8.39 (s, 1H), 7.36 (d, *J* = 9.2 Hz, 2H), 6.85 (d, *J* = 9.1 Hz, 2H), 3.73 (s, 3H), 3.66 – 3.59 (m, 4H), 3.42 (m, 4H).

^{13}C NMR (101 MHz, DMSO) δ 155.6, 154.6, 133.3, 121.7 (2C), 113.6 (2C), 66.1 (2C), 55.1, 44.3 (2C).

HRMS (ESI) *m/z* calculated for $C_{12}H_{16}N_2O_3$ $[M+H]^+$: 237.1239, found 237.1232.

1,1-diisopropyl-3-(4-methoxyphenyl)urea (4j)

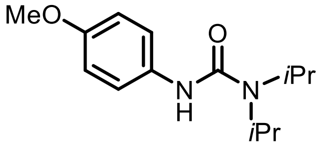

Prepared according to the general procedure. 4-methoxyphenyl isocyanate (1.00 mL, 7.70 mmol, 1.0 equiv.) was added to hexanes (50.0 mL, 0.15 M). Diisopropylamine (3.20 mL, 23.1 mmol, 3.0 equiv.) was then added dropwise. The reaction was allowed to stir for 20 min, and the desired urea precipitated from solution. The product was filtered and rinsed with hexanes to afford the product as a white solid in quantitative yield. No further purification was required.

^{1}H NMR (400 MHz, DMSO-d_6) δ 7.86 (s, 1H), 7.34 (d, *J* = 9.0 Hz, 2H), 6.82 (d, *J* = 9.0 Hz, 2H), 3.84 (m, 2H), 3.73 (s, 3H), 1.28 (s, 6H), 1.26 (s, 6H).

^{13}C NMR (101 MHz, DMSO) δ 154.3, 154.2, 133.9, 121.9 (2C), 113.3 (2C), 55.1, 45.4 (2C), 21.2 (4C).

HRMS (ESI) *m/z* calculated for $C_{14}H_{22}N_2O_2$ $[M+H]^+$: 251.1759, found 251.1753.

1,1-dicyclohexyl-3-(4-methoxyphenyl)urea (4k)

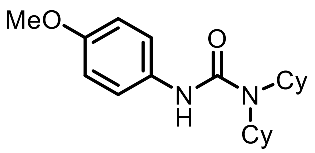

Prepared according to the general procedure. 4-methoxyphenyl isocyanate (1.00 mL, 7.70 mmol, 1.0 equiv.) was added to hexanes (50.0 mL, 0.15 M). Dicyclohexylamine (4.60 mL, 23.1 mmol, 3.0 equiv.) was then added dropwise. The reaction was allowed to stir for 20 min, and the desired urea precipitated from solution. The product was filtered and rinsed with hexanes to afford the product as a white solid in quantitative yield. No further purification was required.

^{1}H NMR (400 MHz, DMSO-d_6) δ 7.85 (s, 1H), 7.31 (d, *J* = 9.0 Hz, 2H), 6.84 (d, J = 9.0 Hz, 2H), 3.74 (s, 3H), 3.34 (m, 2H), 1.93 (m, 4H), 1.75 (m, 4H), 1.59 (m, 6H), 1.36 (m, 4H), 1.11 (m, 2H).

^{13}C NMR (101 MHz, DMSO) δ 154.4, 154.3, 133.8, 122.3 (2C), 113.2 (2C), 55.0, 54.8, 31.0 (4C), 25.9 (4C), 25.8 (2C), 25.1.

HRMS (ESI) *m/z* calculated for $C_{20}H_{30}N_2O_2$ $[M+H]^+$: 331.2385, found 331.2375.

Ligand Studies

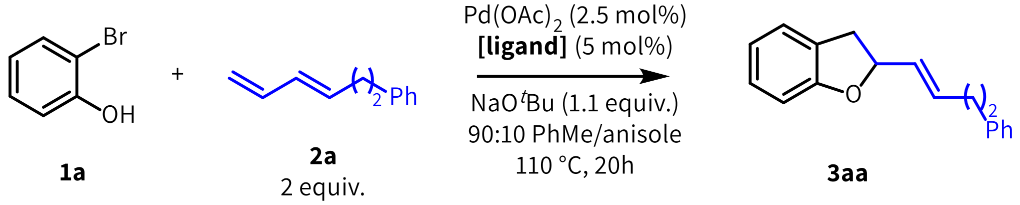

Entry	Ligand	Yield 3aa
	**Ligand Comparison**	
1	No Ligand	49%
2	PPh_3	51%
3	dppe	52%
4	Tris(*o*-tolyl)phosphine	53%
5	XPHOS	56%
6	^{t}BuXPHOS	48%
7	RuPHOS	51%
	**Urea Substitution**	
8	**4a**	68%
9	**4b**	54%
10	SI **L1**	59%
11	SI **L2**	56%
12	**4c**	51%
13	**4d**	57%
14	**4e**	65%
15	**4f**	56%
16	**4g**	35%
	**Substituent effects**	
17	**4h**	54%

18	**4i**	49%
19	**4j**	57%
20	**4k**	59%

General procedure for ligand screening: All reactions were performed at 0.500 mmol scale. Urea ligand (0.025 mmol, 5.00 mol%), $Pd(OAc)_2$ (2.8 mg, 0.0125 mmol, 2.50 mol%), 90:10 PhMe/PhOMe (2.00 mL, 0.25 M) were added to a 2 dram vial and allowed to stir for 20 min. 2-bromophenol **1a** (58.0 µL, 0.500 mmol, 1.0 equiv.) and 1,3-diene **2a** (158 mg, 1.00 mmol, 2.0 equiv.) were added. Finally, NaO*t*Bu (53.0 mg, 0.550 mmol, 1.1 equiv.) was added and the reaction was allowed to stir vigorously at 110°C in an aluminum block with 2-dram slots over a heated stir plate and thermo-probe for 20 hours. Upon reaction completion, the solution was cooled to room temperature, filtered through celite with EtOAc and concentrated under reduced pressure. The crude material was purified by column chromatography using 100% hexanes → 5% EtOAc/ 95% hexanes.

Urea Substitution

Entry 1: No added ligand. General procedure for ligand screening was followed. $Pd(OAc)_2$ (2.8 mg, 0.0125 mmol, 2.50 mol%), 90:10 PhMe/PhOMe (2.00 mL, 0.25 M), 2-bromophenol **1a** (58.0 µL, 0.500 mmol, 1.0 equiv.), **2a** (158 mg, 1.00 mmol, 2.0 equiv.), and NaO*t*Bu (53.0 mg, 0.550 mmol, 1.1 equiv.) were used. The crude material was then purified by column chromatography using 100% hexanes → 5% EtOAc/Hexanes.

Run Number: (isolated yield in mg, isolated yield in mmol, isolated yield in percent)

Run 1: (58.2 mg, 0.235 mmol, 47%)
Run 2: (61.0 mg, 0.245 mmol, 49%)
Run 3: (63.4 mg, 0.250 mmol, 50%)
Average: 49% yield

Entry 2: PPh_3. General procedure for ligand screening was followed. PPh_3 (6.60 mg, 0.025 mmol, 5.00 mol%), $Pd(OAc)_2$ (2.8 mg, 0.0125 mmol, 2.50 mol%), 90:10 PhMe/PhOMe (2.00 mL, 0.25 M), 2-bromophenol **1a** (58.0 µL, 0.500 mmol, 1.0 equiv.), **2a** (158 mg, 1.00 mmol, 2.0 equiv.), and NaO*t*Bu (53.0 mg, 0.550 mmol, 1.1 equiv.) were used. The crude material was then purified by column chromatography using 100% hexanes → 5% EtOAc/Hexanes.

Run Number: (isolated yield in mg, isolated yield in mmol, isolated yield in percent)

Run 1: (60.9 mg, 0.240 mmol, 48%)
Run 2: (64.8 mg, 0.260 mmol, 52%)
Run 3: (65.4 mg, 0.260 mmol, 52%)
Average: 51% yield

Entry 3: dppe. General procedure for ligand screening was followed. dppe, (10.3 mg, 0.025 mmol, 5.00 mol%), $Pd(OAc)_2$ (2.8 mg, 0.0125 mmol, 2.50 mol%), 90:10 PhMe/PhOMe (2.00 mL, 0.25 M), 2-bromophenol **1a** (58.0 µL, 0.500 mmol, 1.0 equiv.), **2a** (158 mg, 1.00 mmol, 2.0 equiv.), and NaO^{t}Bu (53.0 mg, 0.550 mmol, 1.1 equiv.) were used. The crude material was then purified by column chromatography using 100% hexanes → 5% EtOAc/Hexanes.

Run Number: (isolated yield in mg, isolated yield in mmol, isolated yield in percent)

Run 1: (61.3 mg, 0.240 mmol, 48%)
Run 2: (66.1 mg, 0.265 mmol, 53%)
Run 3: (66.9 mg, 0.265 mmol, 53%)
Average: 52% yield

Entry 4: Tris(*o*-tolyl)phosphine. General procedure for ligand screening was followed. Tris(*o*-tolyl)phosphine (7.60 mg, 0.025 mmol, 5.00 mol%), $Pd(OAc)_2$ (2.8 mg, 0.0125 mmol, 2.50 mol%), 90:10 PhMe/PhOMe (2.00 mL, 0.25 M), 2-bromophenol **1a** (58.0 µL, 0.500 mmol, 1.0 equiv.), **2a** (158 mg, 1.00 mmol, 2.0 equiv.), and NaO^{t}Bu (53.0 mg, 0.550 mmol, 1.1 equiv.) were used. The crude material was then purified by column chromatography using 100% hexanes → 5% EtOAc/Hexanes.

Run Number: (isolated yield in mg, isolated yield in mmol, isolated yield in percent)

Run 1: (58.2 mg, 0.260 mmol, 52%)
Run 2: (57.4 mg, 0.260 mmol, 52%)
Run 3: (60.9 mg, 0.270 mmol, 54%)
Average: 53% yield

Entry 5: XPhos. General procedure for ligand screening was followed. XPhos (11.9 mg, 0.025 mmol, 5.00 mol%), $Pd(OAc)_2$ (2.8 mg, 0.0125 mmol, 2.50 mol%), 90:10 PhMe/PhOMe (2.00 mL, 0.25 M), 2-bromophenol **1a** (58.0 µL, 0.500 mmol, 1.0 equiv.), **2a** (158 mg, 1.00 mmol, 2.0 equiv.), and NaO^{t}Bu (53.0 mg, 0.550 mmol, 1.1 equiv.) were used. The crude material was then purified by column chromatography using 100% hexanes → 5% EtOAc/Hexanes.

Run Number: (isolated yield in mg, isolated yield in mmol, isolated yield in percent)

Run 1: (71.8 mg, 0.285 mmol, 57%)
Run 2: (68.9 mg, 0.275 mmol, 55%)
Run 3: (70.9 mg, 0.285 mmol, 57%)
Average: 56% yield

Entry 6: ^{t}BuXPhos. General procedure for ligand screening was followed. ^{t}BuXPhos (10.6 mg, 0.025 mmol, 5.00 mol%), $Pd(OAc)_2$ (2.8 mg, 0.0125 mmol, 2.50 mol%), 90:10 PhMe/PhOMe (2.00 mL, 0.25 M), 2-bromophenol **1a** (58.0 µL, 0.500 mmol, 1.0 equiv.), **2a** (158 mg, 1.00 mmol, 2.0 equiv.), and NaO^{t}Bu (53.0 mg, 0.550 mmol, 1.1 equiv.) were

used. The crude material was then purified by column chromatography using 100% hexanes → 5% EtOAc/Hexanes.

Run Number: (isolated yield in mg, isolated yield in mmol, isolated yield in percent)

Run 1: (53.8 mg, 0.242 mmol, 48%)
Run 2: (52.8 mg, 0.240 mmol, 48%)
Run 3: (51.4 mg, 0.230 mmol, 46%)
Average: 48% yield

Entry 7: RuPhos. General procedure for ligand screening was followed. RuPhos, (11.7 mg, 0.025 mmol, 5.00 mol%), $Pd(OAc)_2$ (2.8 mg, 0.0125 mmol, 2.50 mol%), 90:10 PhMe/PhOMe (2.00 mL, 0.25 M), 2-bromophenol **1a** (58.0 μL, 0.500 mmol, 1.0 equiv.), **2a** (158 mg, 1.00 mmol, 2.0 equiv.), and NaO*t*Bu (53.0 mg, 0.550 mmol, 1.1 equiv.) were used. The crude material was then purified by column chromatography using 100% hexanes → 5% EtOAc/Hexanes.

Run Number: (isolated yield in mg, isolated yield in mmol, isolated yield in percent)

Run 1: (61.3 mg, 0.240 mmol, 48%)
Run 2: (66.1 mg, 0.265 mmol, 53%)
Run 3: (66.9 mg, 0.265 mmol, 53%)
Average: 51% yield

Entry 8: Urea (4a). General procedure for ligand screening was followed. **4a** (1.5 mg, 0.025 mmol, 5.00 mol%), $Pd(OAc)_2$ (2.8 mg, 0.0125 mmol, 2.50 mol%), 90:10 PhMe/PhOMe (2.00 mL, 0.25 M), 2-bromophenol **1a** (58.0 μL, 0.500 mmol, 1.0 equiv.), **2a** (158 mg, 1.00 mmol, 2.0 equiv.), and NaO*t*Bu (53.0 mg, 0.550 mmol, 1.1 equiv.) were used. The crude material was then purified by column chromatography using 100% hexanes → 5% EtOAc/Hexanes.

Run Number: (isolated yield in mg, isolated yield in mmol, isolated yield in percent)

Run 1: (83.8 mg, 0.335 mmol, 67%)
Run 2: (84.1 mg, 0.335 mmol, 67%)
Run 3: (86.2 mg, 0.345 mmol, 69%)
Average: 68% yield

Entry 9: Phenyl Urea (4b). General procedure for ligand screening was followed. **4b** (3.4 mg, 0.025 mmol, 5.00 mol%), $Pd(OAc)_2$ (2.8 mg, 0.0125 mmol, 2.50 mol%), 90:10 PhMe/PhOMe (2.00 mL, 0.25 M), 2-bromophenol **1a** (58.0 μL, 0.500 mmol, 1.0 equiv.), **2a** (158 mg, 1.00 mmol, 2.0 equiv.), and NaO*t*Bu (53.0 mg, 0.550 mmol, 1.1 equiv.) were used. The crude material was then purified by column chromatography using 100% hexanes → 5% EtOAc/Hexanes.

Run Number: (isolated yield in mg, isolated yield in mmol, isolated yield in percent)

Run 1: (67.2 mg, 0.270 mmol, 54%)
Run 2: (65.1 mg, 0.260 mmol, 52%)
Run 3: (68.7 mg, 0.275 mmol, 55%)
Average: 54% yield

Entry 10: 1-(4-methoxyphenyl)urea (SI L1). General procedure for ligand screening was followed. 1-(4-methoxyphenyl)urea (4.2 mg, 0.025 mmol, 5.00 mol%), $Pd(OAc)_2$ (2.8 mg, 0.0125 mmol, 2.50 mol%), 90:10 PhMe/PhOMe (2.00 mL, 0.25 M), 2-bromophenol **1a** (58.0 µL, 0.500 mmol, 1.0 equiv.), **2a** (158 mg, 1.00 mmol, 2.0 equiv.), and NaO^{t}Bu (53.0 mg, 0.550 mmol, 1.1 equiv.) were used. The crude material was then purified by column chromatography using 100% hexanes → 5% EtOAc/Hexanes.

Run Number: (isolated yield in mg, isolated yield in mmol, isolated yield in percent)

Run 1: (64.6 mg, 0.290 mmol, 58%)
Run 2: (67.2 mg, 0.300 mmol, 60%)
Average: 59% yield

Entry 11: 1-(4-methoxy-2-methylphenyl)urea (SI L2). General procedure for ligand screening was followed. 1-(4-methoxy-2-methylphenyl) urea (4.6 mg, 0.025 mmol, 5.00 mol%), $Pd(OAc)_2$ (2.8 mg, 0.0125 mmol, 2.50 mol%), 90:10 PhMe/PhOMe (2.00 mL, 0.25 M), 2-bromophenol **1a** (58.0 µL, 0.500 mmol, 1.0 equiv.), **2a** (158 mg, 1.00 mmol, 2.0 equiv.), and NaO^{t}Bu (53.0 mg, 0.550 mmol, 1.1 equiv.) were used. The crude material was then purified by column chromatography using 100% hexanes → 5% EtOAc/Hexanes.

Run Number: (isolated yield in mg, isolated yield in mmol, isolated yield in percent)

Run 1: (60.0 mg, 0.290 mmol, 54%)
Run 2: (64.1 mg, 0.300 mmol, 57%)
Average: 56% yield

Entry 12: Urea (4c). General procedure for ligand screening was followed. **4c** (4.80 mg, 0.025 mmol, 5.00 mol%), $Pd(OAc)_2$ (2.8 mg, 0.0125 mmol, 2.50 mol%), 90:10 PhMe/PhOMe (2.00 mL, 0.25 M), 2-bromophenol **1a** (58.0 µL, 0.500 mmol, 1.0 equiv.), **2a** (158 mg, 1.00 mmol, 2.0 equiv.), and NaO^{t}Bu (53.0 mg, 0.550 mmol, 1.1 equiv.) were used. The crude material was then purified by column chromatography using 100% hexanes → 5% EtOAc/Hexanes.

Run Number: (isolated yield in mg, isolated yield in mmol, isolated yield in percent)

Run 1: (61.3 mg, 0.245 mmol, 49%)
Run 2: (64.0 mg, 0.255 mmol, 51%)
Run 3: (66.2 mg, 0.265 mmol, 53%)
Average: 51% yield

Entry 13: Urea (4d). General procedure for ligand screening was followed. **4d** (5.1 mg, 0.025 mmol, 5.00 mol%), $Pd(OAc)_2$ (2.8 mg, 0.0125 mmol, 2.50 mol%), 90:10 PhMe/PhOMe (2.00 mL, 0.25 M), 2-bromophenol **1a** (58.0 µL, 0.500 mmol, 1.0 equiv.), **2a** (158 mg, 1.00 mmol, 2.0 equiv.), and NaO*t*Bu (53.0 mg, 0.550 mmol, 1.1 equiv.) were used. The crude material was then purified by column chromatography using 100% hexanes → 5% EtOAc/Hexanes.

Run Number: (isolated yield in mg, isolated yield in mmol, isolated yield in percent)

Run 1: (67.5 mg, 0.270 mmol, 54%)
Run 2: (71.2 mg, 0.285 mmol, 57%)
Run 3: (73.9 mg, 0.295 mmol, 59%)
Average: 57% yield

Entry 14: Urea (4e). General procedure for ligand screening was followed. **4e** (5.9 mg, 0.025 mmol, 5.00 mol%), $Pd(OAc)_2$ (2.8 mg, 0.0125 mmol, 2.50 mol%), 90:10 PhMe/PhOMe (2.00 mL, 0.25 M), 2-bromophenol **1a** (58.0 µL, 0.500 mmol, 1.0 equiv.), **2a** (158 mg, 1.00 mmol, 2.0 equiv.), and NaO*t*Bu (53.0 mg, 0.550 mmol, 1.1 equiv.) were used. The crude material was then purified by column chromatography using 100% hexanes → 5% EtOAc/Hexanes.

Run Number: (isolated yield in mg, isolated yield in mmol, isolated yield in percent)

Run 1: (80.0 mg, 0.320 mmol, 64%)
Run 2: (81.5 mg, 0.330 mmol, 65%
Run 3: (83.5 mg, 0.335 mmol, 67%)
Average: 65% yield

Entry 15: Urea (4f). General procedure for ligand screening was followed. **4f** (6.8 mg, 0.025 mmol, 5.00 mol%), $Pd(OAc)_2$ (2.8 mg, 0.0125 mmol, 2.50 mol%), 90:10 PhMe/PhOMe (2.00 mL, 0.25 M), 2-bromophenol **1a** (58.0 µL, 0.500 mmol, 1.0 equiv.), **2a** (158 mg, 1.00 mmol, 2.0 equiv.), and NaO*t*Bu (53.0 mg, 0.550 mmol, 1.1 equiv.) were used. The crude material was then purified by column chromatography using 100% hexanes → 5% EtOAc/Hexanes.

Run Number: (isolated yield in mg, isolated yield in mmol, isolated yield in percent)

Run 1: (71.1 mg, 0.285 mmol, 57%)
Run 2: (71.9 mg, 0.285 mmol, 57%)
Run 3: (66.0 mg, 0.265 mmol, 53%)
Average: 56% yield

Entry 16: Urea (4g). General procedure for ligand screening was followed. **4g** (5.5 mg, 0.025 mmol, 5.00 mol%), $Pd(OAc)_2$ (2.8 mg, 0.0125 mmol, 2.50 mol%), 90:10 PhMe/PhOMe (2.00 mL, 0.25 M), 2-bromophenol **1a** (58.0 µL, 0.500 mmol, 1.0 equiv.), **2a** (158 mg, 1.00 mmol, 2.0 equiv.), and NaO*t*Bu (53.0 mg, 0.550 mmol, 1.1 equiv.) were

used. The crude material was then purified by column chromatography using 100% hexanes → 5% EtOAc/Hexanes.

Run Number: (isolated yield in mg, isolated yield in mmol, isolated yield in percent)

Run 1: (41.6 mg, 0.165 mmol, 33%)
Run 2: (45.7 mg, 0.180 mmol, 36%)
Run 3: (46.2 mg, 0.185 mmol, 37%)
Average: 35% yield

Entry 17: Urea (4h). General procedure for ligand screening was followed. **4h** (5.5 mg, 0.025 mmol, 5.00 mol%), $Pd(OAc)_2$ (2.8 mg, 0.0125 mmol, 2.50 mol%), 90:10 PhMe/PhOMe (2.00 mL, 0.25 M), 2-bromophenol **1a** (58.0 μL, 0.500 mmol, 1.0 equiv.), **2a** (158 mg, 1.00 mmol, 2.0 equiv.), and NaO*t*Bu (53.0 mg, 0.550 mmol, 1.1 equiv.) were used. The crude material was then purified by column chromatography using 100% hexanes → 5% EtOAc/Hexanes.

Run Number: (isolated yield in mg, isolated yield in mmol, isolated yield in percent)

Run 1: (70.1 mg, 0.280 mmol, 56%)
Run 2: (67.5 mg, 0.270 mmol, 54%)
Run 3: (66.1 mg, 0.265 mmol, 53%)
Average: 54% yield

Entry 18: Urea (4i). General procedure for ligand screening was followed. **4i** (5.9 mg, 0.025 mmol, 5.00 mol%), $Pd(OAc)_2$ (2.8 mg, 0.0125 mmol, 2.50 mol%), 90:10 PhMe/PhOMe (2.00 mL, 0.25 M), 2-bromophenol **1a** (58.0 μL, 0.500 mmol, 1.0 equiv.), **2a** (158 mg, 1.00 mmol, 2.0 equiv.), and NaO*t*Bu (53.0 mg, 0.550 mmol, 1.1 equiv.) were used. The crude material was then purified by column chromatography using 100% hexanes → 5% EtOAc/Hexanes.

Run Number: (isolated yield in mg, isolated yield in mmol, isolated yield in percent)

Run 1: (64.9 mg, 0.260 mmol, 52%)
Run 2: (61.5 mg, 0.245 mmol, 49%)
Run 3: (60.7 mg, 0.280 mmol, 48%)
Average: 49% yield

Entry 19: Urea (4j). General procedure for ligand screening was followed. **4j** (6.3 mg, 0.025 mmol, 5.00 mol%), $Pd(OAc)_2$ (2.8 mg, 0.0125 mmol, 2.50 mol%), 90:10 PhMe/PhOMe (2.00 mL, 0.25 M), 2-bromophenol **1a** (58.0 μL, 0.500 mmol, 1.0 equiv.), **2a** (158 mg, 1.00 mmol, 2.0 equiv.), and NaO*t*Bu (53.0 mg, 0.550 mmol, 1.1 equiv.) were used. The crude material was then purified by column chromatography using 100% hexanes → 5% EtOAc/Hexanes.

Run Number: (isolated yield in mg, isolated yield in mmol, isolated yield in percent)

Run 1: (67.2 mg, 0.270 mmol, 54%)
Run 2: (73.0 mg, 0.290 mmol, 58%)
Run 3: (73.5 mg, 0.295 mmol, 59%)
Average: 57% yield

Entry 20: Urea (4k). General procedure for ligand screening was followed. **4k** (8.3 mg, 0.025 mmol, 5.00 mol%), $Pd(OAc)_2$ (2.8 mg, 0.0125 mmol, 2.50 mol%), 90:10 PhMe/PhOMe (2.00 mL, 0.25 M), 2-bromophenol **1a** (58.0 μL, 0.500 mmol, 1.0 equiv.), **2a** (158 mg, 1.00 mmol, 2.0 equiv.), and NaO*t*Bu (53.0 mg, 0.550 mmol, 1.1 equiv.) were used. The crude material was then purified by column chromatography using 100% hexanes → 5% EtOAc/Hexanes.

Run Number: (isolated yield in mg, isolated yield in mmol, isolated yield in percent)

Run 1: (71.5 mg, 0.285 mmol, 57%)
Run 2: (72.7 mg, 0.290 mmol, 58%)
Run 3: (78.1 mg, 0.310 mmol, 62%)
Average: 59% yield

XPHOS Palladium Binding and Oxidative Addition Derivative Studies

Due to the lack of a strong ligand effect (either beneficial or inhibitory) observed for most phosphines in our reaction, ^{31}P studies were performed to probe whether phosphine ligands are successfully binding to palladium and surviving reaction conditions. Procedures for the synthesis of XPHOS and $Pd(OAc)_2$ complex and oxidative addition XPHOS, $Pd(OAc)_2$ and bromophenol derivative:

Experiment 1: XPHOS ^{31}P Sample: 9.4 mg (0.197 mmol) of XPHOS was dissolved in 1.0 mL of $CDCl_3$ in preparation for ^{31}P NMR study. ^{31}P NMR experiment was run at 23 °C for 10 min.

^{31}P NMR (162 MHz, $CDCl_3$) δ –9.5.

Experiment 2: XPHOS, $Pd(OAc)_2$ ^{31}P Sample: 48.0 mg (0.100 mmol, 2 equiv.) of XPHOS and 11.3 mg (0.050 mmol, 1 equiv.) was dissolved in 1.0 mL of toluene-*d*8 in preparation for ^{31}P NMR study. ^{31}P NMR experiment was run at 80 °C for 10 min.

^{31}P NMR (162 MHz, $CDCl_3$) δ 129.2, 42.4, –9.5.

Experiment 3: XPHOS, $Pd(OAc)_2$, 2-bromophenol and NaO*t*Bu Oxidative Addition Derivative ^{31}P sample: 48.0 mg (0.100 mmol, 2 equiv.) of XPHOS,11.3 mg (0.050 mmol, 1 equiv.), 6.0 μL (0.050 mmol, 1 equiv.) of 2-bromophenol and 6.0 mg (0.050 mmol, 1 equiv.) of NaO*t*Bu was dissolved in 1.0 mL of toluene-*d*8 in preparation for ^{31}P NMR experimentation. ^{31}P NMR experiment was run at 80 °C for 10 min.

^{31}P NMR (162 MHz, $CDCl_3$) δ 46.3, 44.6, 33.3, –9.5.

Spectra are consistent with similar phosphine complex derivatives characterized by Mazet in 2017 with CPhos and 2-bromophenol.[13]

Experiment 4: XPHOS, 2-bromophenol and NaO*t*Bu. Control ^{31}P Sample: 48.0 mg (0.100 mmol, 2 equiv.) of XPHOS,11.3 mg (0.050 mmol, 1 equiv.), 6.0 µL (0.050 mmol, 1 equiv.) of 2-bromophenol and 6.0 mg (0.050 mmol, 1 equiv.) of NaO*t*Bu was dissolved in 1.0 mL of toluene-*d*8 in preparation for ^{31}P NMR study. ^{31}P NMR experiment was run at 80 °C for 10 min. No ligand degradation was observed.

^{31}P NMR (162 MHz, $CDCl_3$) δ –9.5.

^{31}P NMR spectra of complexes:

XPHOS Ligand

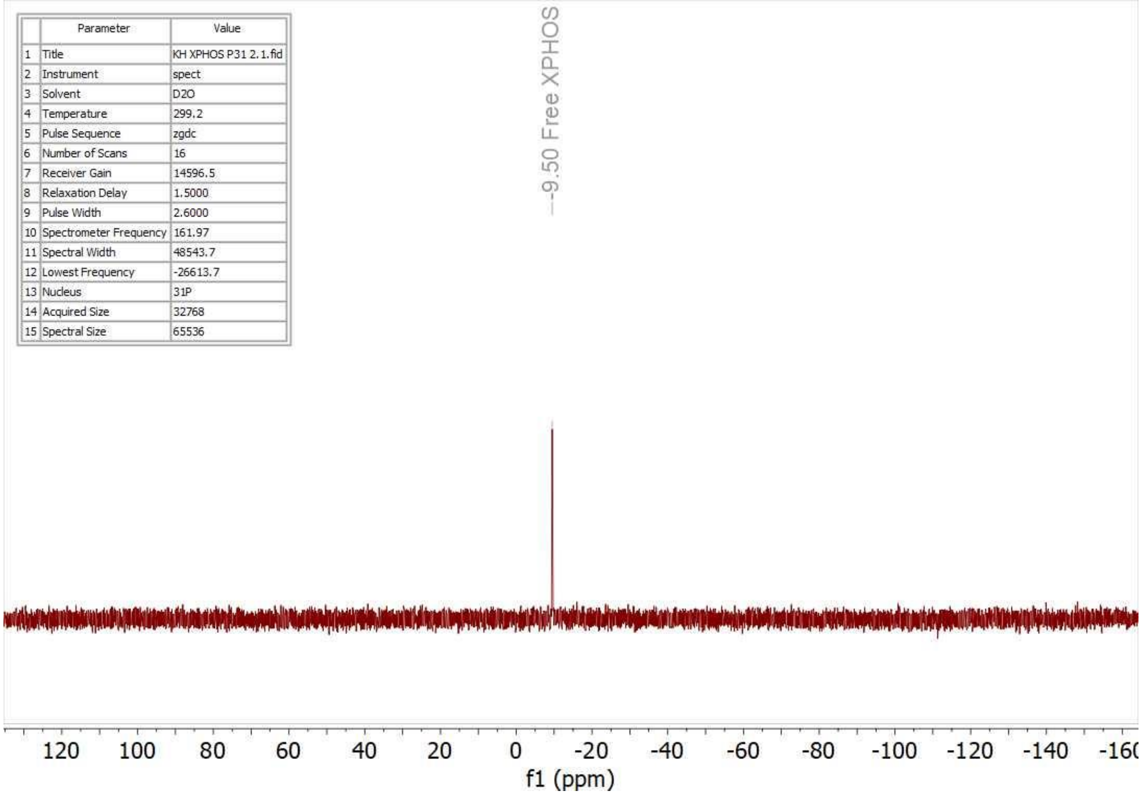

XPHOS and Pd(OAc)$_2$, 80 °C

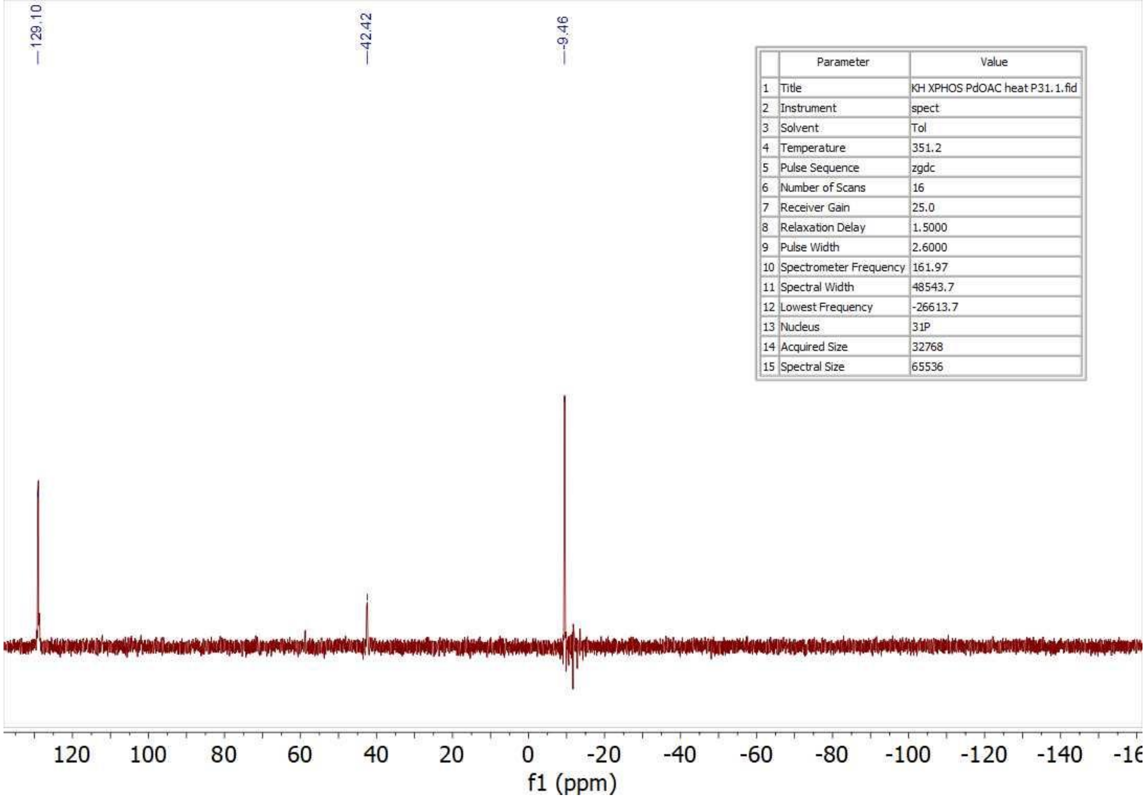

Comparison of Free XPHOS and XPHOS and Pd(OAc)$_2$, 80 °C

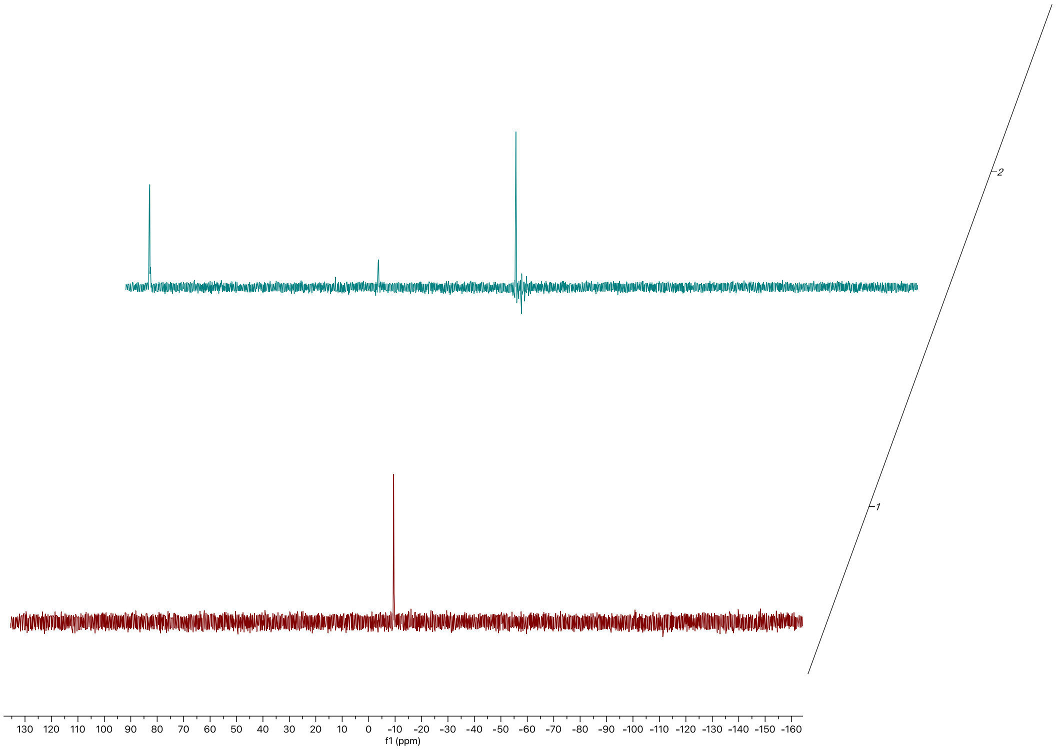

XPHOS, $Pd(OAc)_2$, 2-bromophenol and NaO^{t}Bu, 80 °C

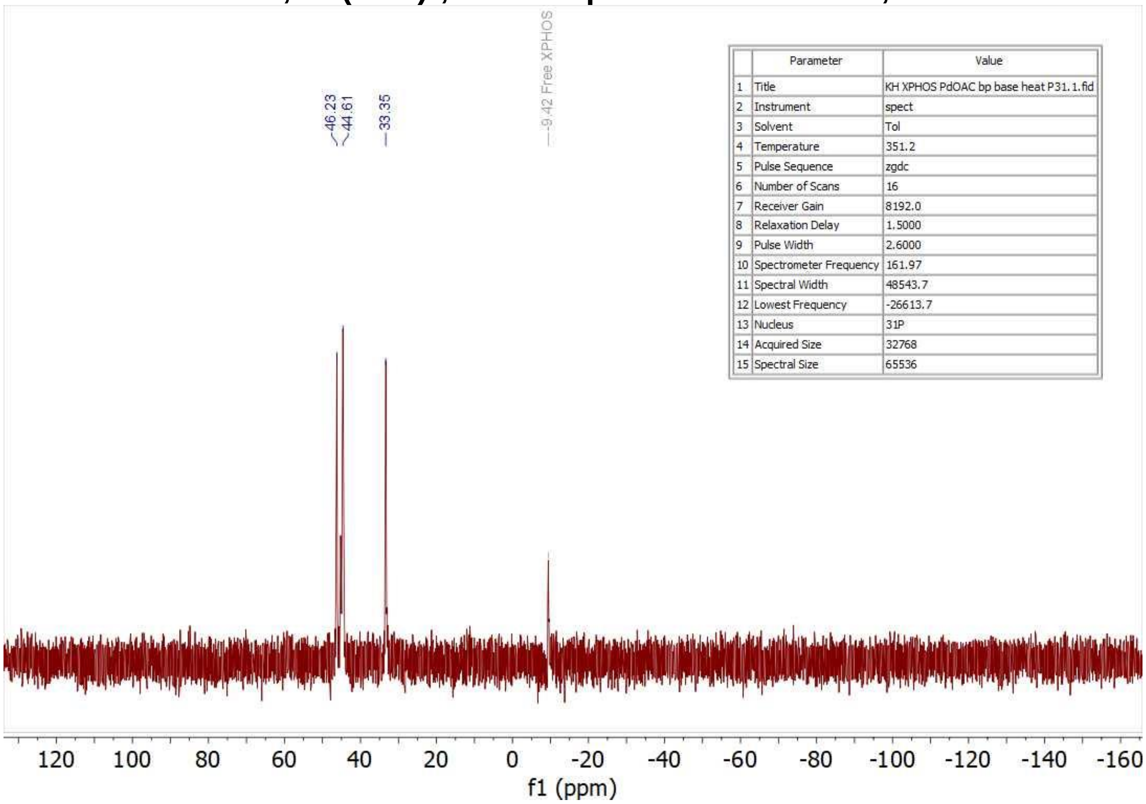

Comparison of XPHOS and XPHOS, $Pd(OAc)_2$, 2-bromophenol and NaO^{t}Bu, 80 °C

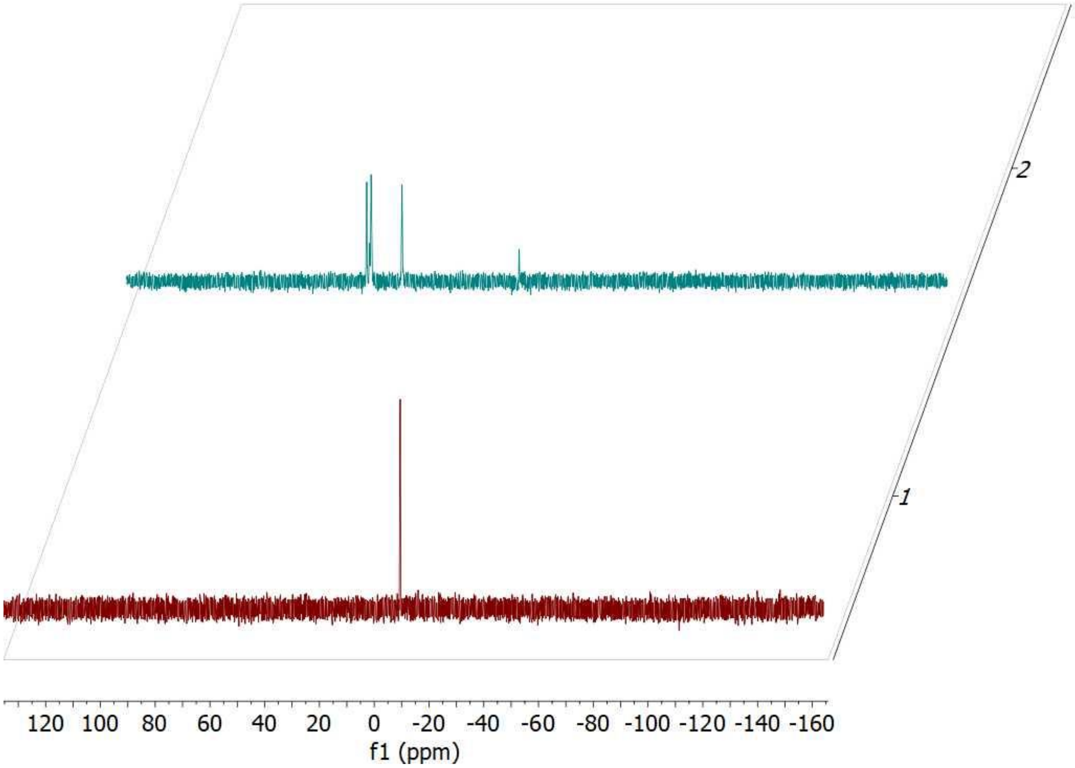

XPHOS, 2-bromophenol and NaO^{t}Bu, heated at 80 °C

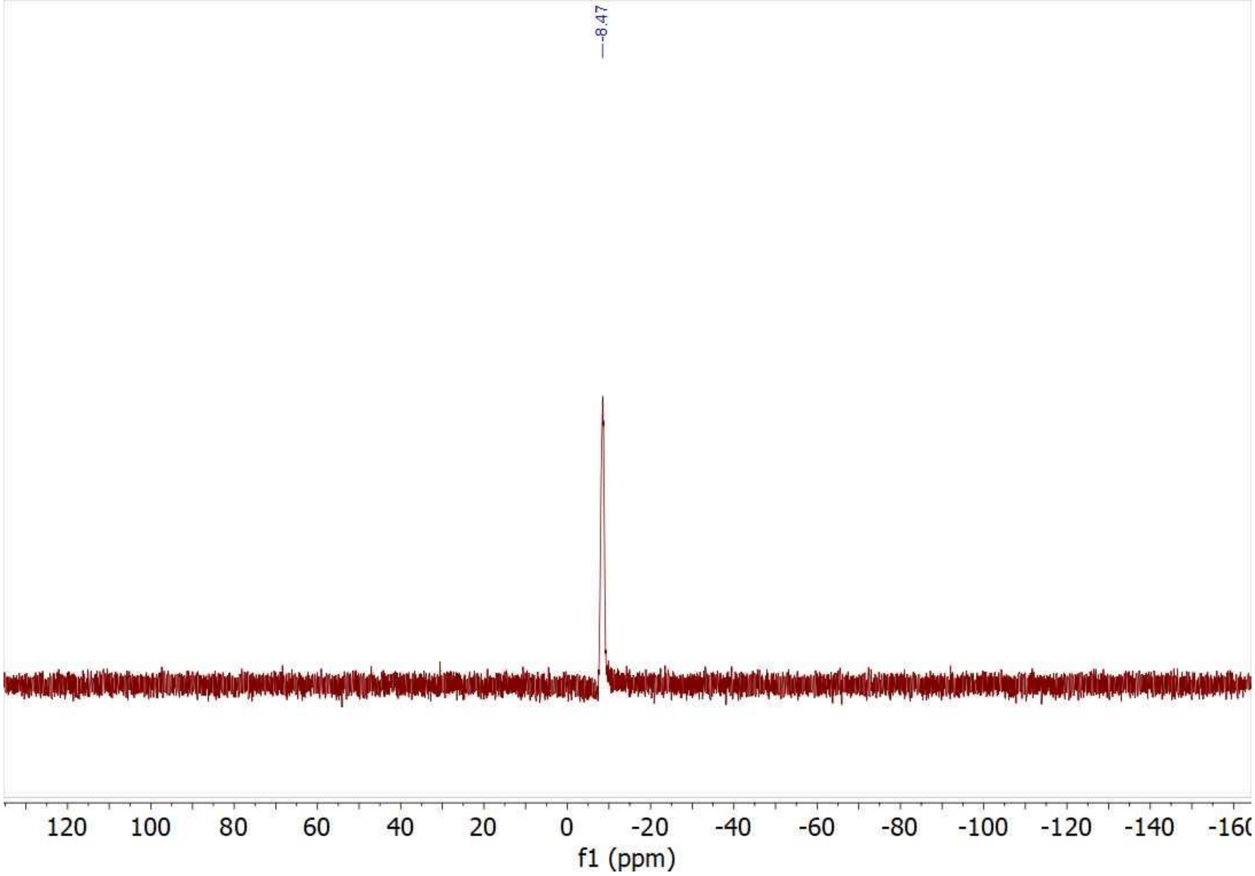

Ligand Screen with Myrcene as More Challenging Olefin

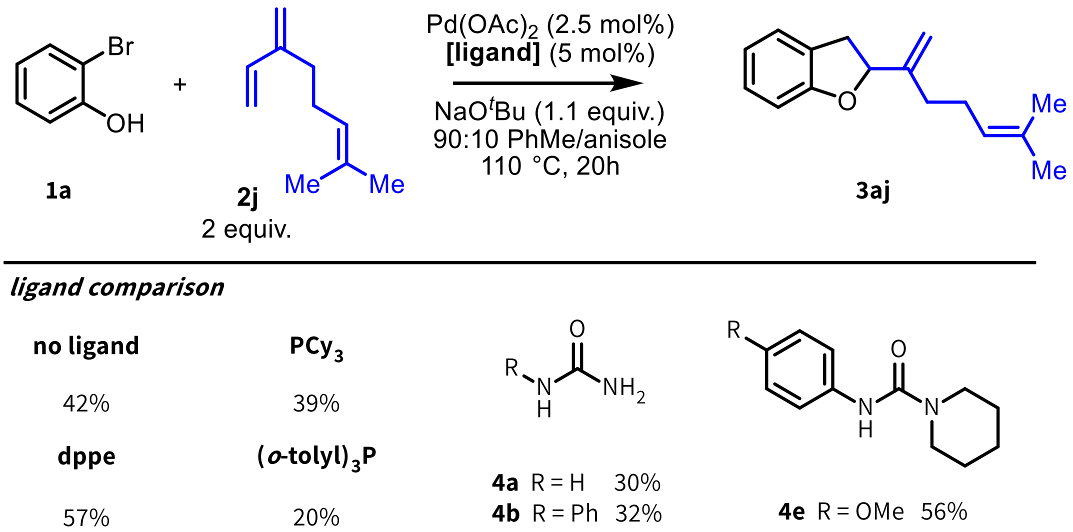

General procedure for ligand screening: All reactions were performed at 0.500 mmol scale. Urea ligand (0.025 mmol, 5.00 mol%), $Pd(OAc)_2$ (2.8 mg, 0.0125 mmol, 2.50 mol%), 90:10 PhMe/PhOMe (2.00 mL, 0.25 M) were added to a 2 dram vial and allowed

to stir for 20 min. 2-bromophenol **1a** (58.0 µL, 0.500 mmol, 1.0 equiv.) and 1,3-diene **2a** (158 mg, 1.00 mmol, 2.0 equiv.) were added. Finally, NaO^{t}Bu (53.0 mg, 0.550 mmol, 1.1 equiv.) was added and the reaction was allowed to stir vigorously at 110°C in an aluminum block with 2-dram slots over a heated stir plate and thermo-probe for 20 hours. Upon reaction completion, the solution was cooled to room temperature, filtered through celite with EtOAc and concentrated under reduced pressure. The crude material was purified by column chromatography using 100% hexanes → 5% EtOAc/ 95% hexanes.

No added ligand. General procedure for ligand screening was followed. $Pd(OAc)_2$ (2.8 mg, 0.0125 mmol, 2.50 mol%), 90:10 PhMe/PhOMe (2.00 mL, 0.25 M), 2-bromophenol **1a** (58.0 µL, 0.500 mmol, 1.0 equiv.), **2a** (158 mg, 1.00 mmol, 2.0 equiv.), and NaO^{t}Bu (53.0 mg, 0.550 mmol, 1.1 equiv.) were used. Product yield was determined by GC calibration curve against internal standard dodecane (50.0 µL, 0.220 mmol).

Run Number: (yield in mmol, yield in percent)

Run 1: (0.196 mmol, 39%)
Run 2: (0.220 mmol, 44%)
Average: 42% yield

PCy_3. General procedure for ligand screening was followed. PCy_3 (14.0 mg, 0.025 mmol, 5.00 mol%), $Pd(OAc)_2$ (2.8 mg, 0.0125 mmol, 2.50 mol%), 90:10 PhMe/PhOMe (2.00 mL, 0.25 M), 2-bromophenol **1a** (58.0 µL, 0.500 mmol, 1.0 equiv.), **2a** (158 mg, 1.00 mmol, 2.0 equiv.), and NaO^{t}Bu (53.0 mg, 0.550 mmol, 1.1 equiv.) were used. Product yield was determined by GC calibration curve against internal standard dodecane (50.0 µL, 0.220 mmol).

Run Number: (yield in mmol, yield in percent)

Run 1: (0.190mmol, 39%)
Run 2: (0.195mmol, 38%)
Average: 39% yield

Tris(*o*-tolyl) phosphine: General procedure for ligand screening was followed.
Tris(*o*-tolyl) phosphine (7.6 mg, 0.025 mmol, 5.00 mol%), $Pd(OAc)_2$ (2.8 mg, 0.0125 mmol, 2.50 mol%), 90:10 PhMe/PhOMe (2.00 mL, 0.25 M), 2-bromophenol **1a** (58.0 µL, 0.500 mmol, 1.0 equiv.), **2a** (158 mg, 1.00 mmol, 2.0 equiv.), and NaO^{t}Bu (53.0 mg, 0.550 mmol, 1.1 equiv.) were used. Product yield was determined by GC calibration curve against internal standard dodecane (50.0 µL, 0.220 mmol).

Run Number: (yield in mmol, yield in percent)

Run 1: (0.095 mmol, 19%)
Run 2: (0.100 mmol, 20%)
Average: 20%

dppe. General procedure for ligand screening was followed. dppe, (10.3 mg, 0.025 mmol, 5.00 mol%), $Pd(OAc)_2$ (2.8 mg, 0.0125 mmol, 2.50 mol%), 90:10 PhMe/PhOMe (2.00 mL, 0.25 M), 2-bromophenol **1a** (58.0 μL, 0.500 mmol, 1.0 equiv.), **2a** (158 mg, 1.00 mmol, 2.0 equiv.), and NaO^{t}Bu (53.0 mg, 0.550 mmol, 1.1 equiv.) were used. Product yield was determined by GC calibration curve against internal standard dodecane (50.0 μL, 0.220 mmol).

Run Number: (yield in mmol, yield in percent)

Run 1: (0.285 mmol, 57%)
Run 2: (0.290 mmol, 56%)
Average: 57% yield

Urea (4a). General procedure for ligand screening was followed. **4a** (1.5 mg, 0.025 mmol, 5.00 mol%), $Pd(OAc)_2$ (2.8 mg, 0.0125 mmol, 2.50 mol%), 90:10 PhMe/PhOMe (2.00 mL, 0.25 M), 2-bromophenol **1a** (58.0 μL, 0.500 mmol, 1.0 equiv.), **2a** (158 mg, 1.00 mmol, 2.0 equiv.), and NaO^{t}Bu (53.0 mg, 0.550 mmol, 1.1 equiv.) were used. Crude material was purified via column chromatography using 100% hexanes → 5% EtOAc/ 95% hexanes to afford the product as a clear oil.

Run Number: (isolated yield in mg, isolated yield in mmol, isolated yield in percent)

Run 1: (38.9 mg, 0.170 mmol, 34%)
Run 2: (34.6 mg, 0.150 mmol, 30%)
Run 3: (31.2 mg, 0.135 mmol, 27%)
Average: 30%

Urea (4b). General procedure for ligand screening was followed. **4b** (3.4 mg, 0.025 mmol, 5.00 mol%), $Pd(OAc)_2$ (2.8 mg, 0.0125 mmol, 2.50 mol%), 90:10 PhMe/PhOMe (2.00 mL, 0.25 M), 2-bromophenol **1a** (58.0 μL, 0.500 mmol, 1.0 equiv.), **2a** (158 mg, 1.00 mmol, 2.0 equiv.), and NaO^{t}Bu (53.0 mg, 0.550 mmol, 1.1 equiv.) were used. Product yield was determined by GC calibration curve against internal standard dodecane (50.0 μL, 0.220 mmol).

Run Number: (yield in mmol, yield in percent)

Run 1: (0.165 mmol, 33%)
Run 2: (0.150 mmol, 30%)
Average: 32% yield

Urea (4e). General procedure for ligand screening was followed. **4e** (5.9 mg, 0.025 mmol, 5.00 mol%), $Pd(OAc)_2$ (2.8 mg, 0.0125 mmol, 2.50 mol%), 90:10 PhMe/PhOMe (2.00 mL, 0.25 M), 2-bromophenol **1a** (58.0 μL, 0.500 mmol, 1.0 equiv.), **2a** (158 mg, 1.00 mmol, 2.0 equiv.), and NaO^{t}Bu (53.0 mg, 0.550 mmol, 1.1 equiv.) were used. Crude material was purified via column chromatography using 100% hexanes → 5% EtOAc/ 95% hexanes to afford the product as a clear oil.

Run Number: (isolated yield in mg, isolated yield in mmol, isolated yield in percent)

Run 1: (61.8 mg, 0.271 mmol, 54%)
Run 2: (64.9 mg, 0.284 mmol, 57%)
Run 3: (65.2 mg, 0.286 mmol, 57%)
Average: 56% yield

Reaction Condition Optimization

Table S1. Reaction Optimization Studies

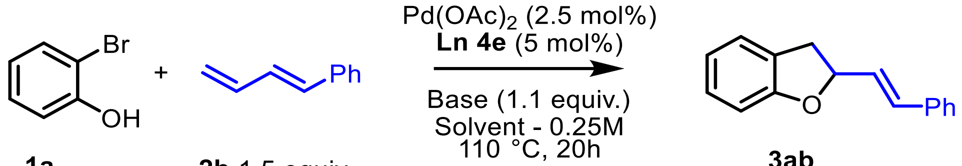

1a **2b** 1.5 equiv. **3ab**

Entry	Base	Solvent	Yield 3ab
**Solvent Screen**			
1	NaO^{t}Bu	Toluene	71%
2	NaO^{t}Bu	Anisole	78%
3	NaO^{t}Bu	DMF	53%
4	NaO^{t}Bu	1:1 Toluene/Anisole	80%
5	NaO^{t}Bu	9:1 Toluene/Anisole	78%
**Base Screen**			
6	Na_2CO_3	9:1 Toluene/Anisole	7%
7	NaO^{t}Bu	9:1 Toluene/Anisole	68%
8	NaOAc	9:1 Toluene/Anisole	5%
9	Na_3PO_4	9:1 Toluene/Anisole	n.r.
10	NaOH	9:1 Toluene/Anisole	8%
**Cation Screen**			
11	NaO^{t}Bu	9:1 Toluene/Anisole	79%
12	KO^{t}Bu	9:1 Toluene/Anisole	86%
13	LiO^{t}Bu	9:1 Toluene/Anisole	7%
**Ligand Loading 4e (Pd:Ln)**			
14	1:1 Pd:Ln		41%
15	1:2 Pd:Ln		65%
16	1:4 Pd:Ln		63%
**Reaction Tolerance** (NaO^{t}Bu, 9:1 Toluene/Anisole)			

17	Inert atmosphere	52%
18	H_2O (1.0 equiv.) added	75%
19	H_2O (5.0 equiv.) added	67%

General Procedure for dihydrobenzofurans from 1,3-diene and 2-bromophenol: All reactions were performed at 0.500 mmol scale. Trisubstituted urea **4e** (5.9 mg, 0.0250 mmol, 5.00 mol%), $Pd(OAc)_2$ (2.8 mg, 0.0125 mmol, 2.50 mol%), 2.0 mL solvent (0.25 M total concentration) were added to a 2 dram vial and allowed to stir for 20 min. 2-bromophenol **1a** (58.0 μL, 0.500 mmol, 1.0 equiv.) and 1,3-diene **2b** (98.0 μL, 0.750 mmol, 1.5 equiv.) were then added. Finally, base (0.550 mmol, 1.1 equiv.) was added and the reaction was allowed to stir vigorously at 110°C in an aluminum block with 2-dram slots over a heated stir plate and thermo-probe for 20 hours. Upon reaction completion, the solution was cooled to room temperature, filtered through celite with EtOAc and was concentrated under reduced pressure. Product yield was determined by GC calibration curve against internal standard dodecane (50.0 μL, 0.220 mmol).

Solvent Screen

Entry 1: Toluene. General procedure for screening was followed. **4e** (5.9 mg, 0.025 mmol, 5.00 mol%), $Pd(OAc)_2$ (2.8 mg, 0.0125 mmol, 2.50 mol%), toluene (2.00 mL, 0.25 M), 2-bromophenol **1a** (58.0 μL, 0.500 mmol, 1.0 equiv.), **2b** (98.0 μL, 0.750 mmol, 1.5 equiv.), and NaO^tBu (53.0 mg, 0.550 mmol, 1.1 equiv.) were used. Product yield was determined by GC calibration curve against internal standard dodecane (50.0 μL, 0.220 mmol).

Run Number: (yield in mmol, yield in percent)

Run 1: (0.350 mmol, 70%)
Run 2: (0.355 mmol, 71%)
Average: 71% yield

Entry 2: Anisole. General procedure for screening was followed. **4e** (5.9 mg, 0.025 mmol, 5.00 mol%), $Pd(OAc)_2$ (2.8 mg, 0.0125 mmol, 2.50 mol%), anisole (2.00 mL, 0.25 M), 2-bromophenol **1a** (58.0 μL, 0.500 mmol, 1.0 equiv.), **2b** (98.0 μL, 0.750 mmol, 1.5 equiv.), and NaO^tBu (53.0 mg, 0.550 mmol, 1.1 equiv.) were used. Product yield was determined by GC calibration curve against internal standard dodecane (50.0 μL, 0.220 mmol).

Run Number: (yield in mmol, yield in percent)

Run 1: (0.375 mmol, 75%)
Run 2: (0.405 mmol, 81%)
Average: 78% yield

Entry 3: DMF. General procedure for ligand was followed. **4e** (5.9 mg, 0.025 mmol, 5.00 mol%), $Pd(OAc)_2$ (2.8 mg, 0.0125 mmol, 2.50 mol%), DMF (2.00 mL, 0.25 M), 2-bromophenol **1a** (58.0 μL, 0.500 mmol, 1.0 equiv.), **2b** (98.0 μL, 0.750 mmol, 1.5 equiv.),

and NaO^{t}Bu (53.0 mg, 0.550 mmol, 1.1 equiv.) were used. Product yield was determined by GC calibration curve against internal standard dodecane (50.0 µL, 0.220 mmol).

Run Number: (yield in mmol, yield in percent)

Run 1: (0.255 mmol, 51%)
Run 2: (0.275 mmol, 55%)
Average: 53% yield

Entry 4: 1:1 Toluene/Anisole. General procedure for screening was followed. **4e** (5.9 mg, 0.025 mmol, 5.00 mol%), Pd(OAc)$_2$ (2.8 mg, 0.0125 mmol, 2.50 mol, 2-bromophenol **1a** (58.0 µL, 0.500 mmol, 1.0 equiv.), **2b** (98.0 µL, 0.750 mmol, 1.5 equiv.), 1.00 mL anisole and 1.00 mL toluene (0.25 M total concentration) and NaO^{t}Bu (53.0 mg, 0.550 mmol, 1.1 equiv.) were used. Product yield was determined by GC calibration curve against internal standard dodecane (50.0 µL, 0.220 mmol).

Run Number: (yield in mmol, yield in percent)

Run 1: (0.415 mmol, 83%)
Run 2: (0.380 mmol, 76%)
Average: 80% yield

Entry 5: 9:1 Toluene/Anisole. General procedure for screening was followed. **4e** (5.9 mg, 0.025 mmol, 5.00 mol%), Pd(OAc)$_2$ (2.8 mg, 0.0125 mmol, 2.50 mol%), 2-bromophenol **1a** (58.0 µL, 0.500 mmol, 1.0 equiv.), **2b** (98.0 µL, 0.750 mmol, 1.5 equiv.), 200 µL anisole and 1.8 mL toluene (0.25 M total concentration) and NaO^{t}Bu (53.0 mg, 0.550 mmol, 1.1 equiv.) were used. Product yield was determined by GC calibration curve against internal standard dodecane (50.0 µL, 0.220 mmol).

Run Number: (yield in mmol, yield in percent)

Run 1: (0.390 mmol, 78%)
Run 2: (0.390 mmol, 78%)
Average: 78% yield

Base Screen

Entry 6: Na_2CO_3. General procedure for screening was followed. **4e** (5.9 mg, 0.025 mmol, 5.00 mol%), Pd(OAc)$_2$ (2.8 mg, 0.0125 mmol, 2.50 mol%), 2-bromophenol **1a** (58.0 µL, 0.500 mmol, 1.0 equiv.), **2b** (98.0 µL, 0.750 mmol, 1.5 equiv.), 200 µL anisole and 1.8 mL toluene (0.25 M total concentration)and Na_2CO_3 (58.0 mg, 0.550 mmol, 1.1 equiv.) were used. Product yield was determined by GC calibration curve against internal standard dodecane (50.0 µL, 0.220 mmol).

Run Number: (yield in mmol, yield in percent)

Run 1: (0.035 mmol, 7%)
Run 2: (0.035 mmol, 7%)
Average: 7% yield

Entry 7: NaO^{t}Bu. General procedure for screening was followed. **4e** (5.9 mg, 0.025 mmol, 5.00 mol%), $Pd(OAc)_2$ (2.8 mg, 0.0125 mmol, 2.50 mol%), 2-bromophenol **1a** (58.0 µL, 0.500 mmol, 1.0 equiv.), **2b** (98.0 µL, 0.750 mmol, 1.5 equiv.), 200 µL anisole and 1.8 mL toluene (0.25 M total concentration) and NaO^{t}Bu (53.0 mg, 0.550 mmol, 1.1 equiv.) were used. Product yield was determined by GC calibration curve against internal standard dodecane (50.0 µL, 0.220 mmol). mmol).

Run Number: (yield in mmol, yield in percent)

Run 1: (0.340 mmol, 68%)
Run 2: (0.340 mmol, 68%)
Average: 68% yield

Entry 8: NaOAc. General procedure for screening was followed. **4e** (5.9 mg, 0.025 mmol, 5.00 mol%), $Pd(OAc)_2$ (2.8 mg, 0.0125 mmol, 2.50 mol%), 2-bromophenol **1a** (58.0 µL, 0.500 mmol, 1.0 equiv.), **2b** (98.0 µL, 0.750 mmol, 1.5 equiv.), 200 µL anisole and 1.8 mL toluene (0.25 M total concentration) and NaOAc (45.0 mg, 0.550 mmol, 1.1 equiv.) were used. Product yield was determined by GC calibration curve against internal standard dodecane (50.0 µL, 0.220 mmol).

Run Number: (yield in mmol, yield in percent)

Run 1: (0.020 mmol, 4%)
Run 2: (0.025 mmol, 5%)
Average: 5% yield

Entry 9: Na_3PO_4. General procedure for screening was followed. **4e** (5.9 mg, 0.025 mmol, 5.00 mol%), $Pd(OAc)_2$ (2.8 mg, 0.0125 mmol, 2.50 mol%), 2-bromophenol **1a** (58.0 µL, 0.500 mmol, 1.0 equiv.), **2b** (98.0 µL, 0.750 mmol, 1.5 equiv.), 200 µL anisole and 1.8 mL toluene (0.25 M total concentration) and Na_3PO_4 (78.0 mg, 0.550 mmol, 1.1 equiv.) were used. Product yield was determined by GC calibration curve against internal standard dodecane (50.0 µL, 0.220 mmol).

Run Number: (yield in mmol, yield in percent)

Run 1: n.r.
Run 2: n.r.
Average: n.r.

Entry 10: NaOH. General procedure for screening was followed. **4e** (5.9 mg, 0.025 mmol, 5.00 mol%), $Pd(OAc)_2$ (2.8 mg, 0.0125 mmol, 2.50 mol%), 2-bromophenol **1a** (58.0 µL, 0.500 mmol, 1.0 equiv.), **2b** (98.0 µL, 0.750 mmol, 1.5 equiv.), 200 µL anisole and 1.8 mL

toluene (0.25 M total concentration) and NaOH (22.0 mg, 0.550 mmol, 1.1equiv.) were used. Product yield was determined by GC calibration curve against internal standard dodecane (50.0 μL, 0.220 mmol).

Run Number: (yield in mmol, yield in percent)

Run 1: (0.045 mmol, 9%)
Run 2: (0.035 mmol, 7%)
Average: 8% yield

Cation Screen

Entry 11: NaO^{t}Bu. General procedure for screening was followed. **4e** (5.9 mg, 0.025 mmol, 5.00 mol%), $Pd(OAc)_2$ (2.8 mg, 0.0125 mmol, 2.50 mol%), 2-bromophenol **1a** (58.0 μL, 0.500 mmol, 1.0 equiv.), **2b** (98.0 μL, 0.750 mmol, 1.5 equiv.), 200 μL anisole and 1.8 mL toluene (0.25 M total concentration)and NaO^{t}Bu (53.0 mg, 0.550 mmol, 1.1 equiv.) were used. Product yield was determined by GC calibration curve against internal standard dodecane (50.0 μL, 0.220 mmol).

Run Number: (yield in mmol, yield in percent)

Run 1: (0.380 mmol, 76%)
Run 2: (0.410 mmol, 82%)
Average: 79% yield

Entry 12: KO^{t}Bu. General procedure for screening was followed. **4e** (5.9 mg, 0.025 mmol, 5.00 mol%), $Pd(OAc)_2$ (2.8 mg, 0.0125 mmol, 2.50 mol%), 2-bromophenol **1a** (58.0 μL, 0.500 mmol, 1.0 equiv.), **2b** (98.0 μL, 0.750 mmol, 1.5 equiv.), 200 μL anisole and 1.8 mL toluene (0.25 M total concentration) and KO^{t}Bu (62 mg, 0.550 mmol, 1.1 equiv.) were used. Product yield was determined by GC calibration curve against internal standard dodecane (50.0 μL, 0.220 mmol).

Run Number: (yield in mmol, yield in percent)

Run 1: (0.425 mmol, 85%)
Run 2: (0.430 mmol, 86%)
Average: 86% yield

Entry 13: LiO^{t}Bu. General procedure for screening was followed. **4e** (5.9 mg, 0.025 mmol, 5.00 mol%), $Pd(OAc)_2$ (2.8 mg, 0.0125 mmol, 2.50 mol%), 2-bromophenol **1a** (58.0 μL, 0.500 mmol, 1.0 equiv.), **2b** (98.0 μL, 0.750 mmol, 1.5 equiv.), 200 μL anisole and 1.8 mL toluene (0.25 M total concentration) and LiO^{t}Bu (550 μL of a 1.0 M solution in THF, 44.0 mg, 0.550 mmol, 1.1 equiv.) were used. Product yield was determined by GC calibration curve against internal standard dodecane (50.0 μL, 0.220 mmol).

Run Number: (yield in mmol, yield in percent)

Run 1: (0.025 mmol, 5%)
Run 2: (0.045 mmol, 9%)
Average: 7% yield

Urea Ligand Loading

Entry 14: Ligand Loading (Pd:Ln) 1:1 with Urea (4e). General procedure for ligand screening was followed. **4e** (53.0 mg, 0.013 mmol, 2.50 mol%), $Pd(OAc)_2$ (2.8 mg, 0.0125 mmol, 2.50 mol%), 90:10 PhMe/PhOMe (2.00 mL, 0.25 M), 2-bromophenol **1a** (58.0 µL, 0.500 mmol, 1.0 equiv.), **2b** (98.0 µL, 0.750 mmol, 1.5 equiv.), and NaO^{t}Bu (53.0 mg, 0.550 mmol, 1.1 equiv.) were used. Product yield was determined by GC calibration curve against internal standard dodecane (50.0 µL, 0.220 mmol).

Run Number: (yield in mmol, yield in percent)

Run 1: (0.195 mmol, 38%)
Run 2: (0.220 mmol, 44%)
Average: 41% yield

Entry 15: Ligand Loading (Pd:Ln) 1:2 with Urea (4e). General procedure for ligand screening was followed. **4e** (5.9 mg, 0.025 mmol, 5.00 mol%), $Pd(OAc)_2$ (2.8 mg, 0.0125 mmol, 2.50 mol%), 90:10 PhMe/PhOMe (2.00 mL, 0.25 M), 2-bromophenol **1a** (58.0 µL, 0.500 mmol, 1.0 equiv.), **2b** (98.0 µL, 0.750 mmol, 1.5 equiv.), and NaO^{t}Bu (53.0 mg, 0.550 mmol, 1.1 equiv.) were used. Product yield was determined by GC calibration curve against internal standard dodecane (50.0 µL, 0.220 mmol).

Run Number: (yield in mmol, yield in percent)

Run 1: (0.320 mmol, 64%)
Run 2: (0.330 mmol, 65%)
Average: 65% yield

Entry 16: Ligand Loading (Pd:Ln) 1:4 with Urea (4e). General procedure for ligand screening was followed. **4e** (11.8 mg, 0.050 mmol, 5.00 mol%), $Pd(OAc)_2$ (2.8 mg, 0.0125 mmol, 2.50 mol%), 90:10 PhMe/PhOMe (2.00 mL, 0.25 M), 2-bromophenol **1a** (58.0 µL, 0.500 mmol, 1.0 equiv.), **2b** (98.0 µL, 0.750 mmol, 1.5 equiv.), and NaO^{t}Bu (53.0 mg, 0.550 mmol, 1.1 equiv.) were used. Product yield was determined by GC calibration curve against internal standard dodecane (50.0 µL, 0.220 mmol).

Run Number: (yield in mmol, yield in percent)

Run 1: (0.300 mmol, 60%)
Run 2: (0.325 mmol, 65%)
Average: 63% yield

Reaction Tolerance

Entry 17: Inert atmosphere Control: General procedure was followed. **4e** (5.9 mg, 0.0250 mmol, 5.00 mol%), $Pd(OAc)_2$ (2.8 mg, 0.0125 mmol, 2.50 mol%), 200 μL anisole and 1.8 mL toluene (0.25M total concentration) were added to a flame dried 2 dram vial and allowed to stir for 20 min while sparging under N_2. 2-bromophenol **1a** (58.0 μL, 0.500 mmol, 1.0 equiv.) and 1,3-diene **2b** (98.0 μL, 0.750 mmol, 1.5 equiv.) were added. Finally, NaO*t*Bu (53.0 mg, 0.550 mmol, 1.1 equiv.) was added and the reaction was allowed to stir vigorously at 110°C in an aluminum block with 2-dram slots over a heated stir plate and thermo-probe for 20 hours. Upon reaction completion, the solution was cooled to room temperature, filtered through celite with EtOAc and was concentrated under reduced pressure. The crude material was then purified by column chromatography using 100% hexanes → 5% EtOAc/Hexanes.

<u>Run Number:</u> (isolated yield in mg, isolated yield in mmol, isolated yield in percent)

<u>Run 1:</u> (60.1 mg, 0.270 mmol, 54%)
<u>Run 2:</u> (55.4 mg, 0.250 mmol, 50%)
Average: 52% yield

Entry 18: Water Control (1 equiv. of water): General procedure was followed. **4e** (5.9 mg, 0.0250 mmol, 5.00 mol%), $Pd(OAc)_2$ (2.8 mg, 0.0125 mmol, 2.50 mol%), 200 μL anisole and 1.8 mL toluene (0.25 M total concentration) were added to a 2 dram vial and allowed to stir for 20 min. 2-bromophenol **1a** (58.0 μL, 0.500 mmol, 1.0 equiv.), 1,3-diene **2b** (98.0 μL, 0.750 mmol, 1.5 equiv.) and H_2O (9.00 μL, 0.500 mmol, 1.0 equiv.) were added. Finally, NaO*t*Bu (53.0 mg, 0.550 mmol, 1.1 equiv.) was added and the reaction was allowed to stir vigorously at 110°C in an aluminum block with 2-dram slots over a heated stir plate and thermo-probe for 20 hours. Upon reaction completion, the solution was cooled to room temperature, filtered through celite with EtOAc and was concentrated under reduced pressure. The crude material was then purified by column chromatography using 100% hexanes → 5% EtOAc/Hexanes.

<u>Run Number:</u> (isolated yield in mg, isolated yield in mmol, isolated yield in percent)

<u>Run 1:</u> (83.7 mg, 0.377 mmol, 75%)
<u>Run 2:</u> (82.6 mg, 0.372 mmol, 75%)
Average: 75% yield

Entry 19: Water Control (5 equiv. of water): General procedure was followed. **4e** (5.9 mg, 0.0250 mmol, 5.00 mol%), $Pd(OAc)_2$ (2.8 mg, 0.0125mmol, 2.50 mol%), 200 μL anisole and 1.8 mL toluene (0.25 M total concentration) were added to a 2 dram vial and allowed to stir for 20 min. 2-bromophenol **1a** (58.0 μL, 0.500 mmol, 1.0 equiv.) , 1,3-diene **2b** (98.0 μL, 0.750 mmol, 1.5 equiv.) and H_2O (45.0 μL, 0.500 mmol, 5.0 equiv.) were added. Finally, NaO*t*Bu (53.0 mg, 0.550 mmol, 1.1 equiv.) was added and the reaction was allowed to stir vigorously at 110°C in an aluminum block with 2-dram slots over a heated stir plate and thermo-probe for 20 hours. Upon reaction completion, the solution

was cooled to room temperature, filtered through celite with EtOAc and was concentrated under reduced pressure. The crude material was then purified by column chromatography using 100% hexanes → 5% EtOAc/Hexanes.

Run Number: (isolated yield in mg, isolated yield in mmol, isolated yield in percent)

Run 1: (73.6 mg, 0.331 mmol, 66%)
Run 2: (74.9 mg, 0.337 mmol, 67%)
Average: 67% yield

Gram Scale Reaction

Gram scale: General procedure was followed. Reaction was performed in duplicate at 8.60 mmol scale. **4e** (101 mg, 0.430 mmol, 5.00 mol%), $Pd(OAc)_2$ (48.3 mg, 0.215 mmol, 2.50 mol%), 3.80 mL anisole and 30.6 mL toluene (0.25 M total concentration) were added to a 100 mL RBF and allowed to stir for 20 min. 2-bromophenol **1a** (1.00 mL, 8.60 mmol, 1.0 equiv.) and 1,3-diene **2b** (1.68 mL, 12.9 mmol, 1.5 equiv.) were added. Finally, $NaO^{t}Bu$ (910 mg, 0.550 mmol, 1.1 equiv.) was added and the reaction was allowed to stir vigorously at 110°C in a silicone oil bath for 20 hours. Upon reaction completion, the solution was cooled to room temperature, filtered through celite with EtOAc and was concentrated under reduced pressure. The crude material was then purified by column chromatography using 100% hexanes → 5% EtOAc/Hexanes.

Run Number: (isolated yield in mg, isolated yield in mmol, isolated yield in percent)

Run 1: (1.18 g, 5.33 mmol, 62%)
Run 2: (1.24 g, 5.59 mmol, 65%)
Average: 64% yield

Bromophenol Scope

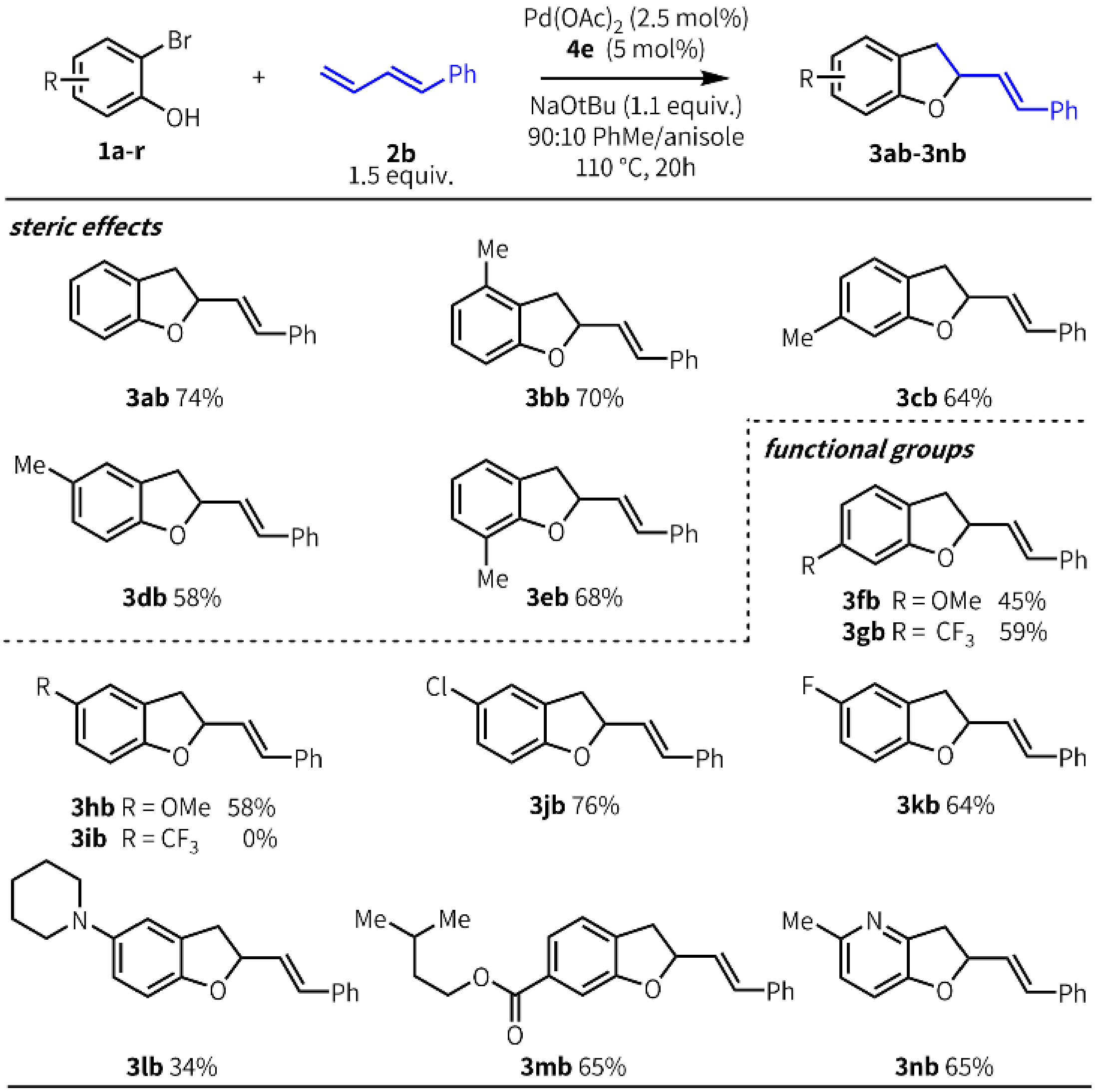

General procedure A: All reactions were performed at 0.500 mmol scale. To a 2-dram vial, a 0.0625M stock solution of $Pd(OAc)_2$ in anisole (0.200 mL) and PhMe (1.80 mL) were added. *N*-(4-methoxyphenyl)piperidine-1-carboxamide **4e** (5.9 mg, 0.025 mmol) was added. After 20 min of rapidly stirring, 2-bromophenol (0.500 mmol, 1.0 equiv.) and diene **2b** (98.0 uL, 0.750 mmol, 1.5 equiv.) were added, followed by NaO^{t}Bu (52.9 mg, 0.550 mmol, 1.1 equiv). The reaction was stirred at 110°C in an aluminum block with 2-dram slots over a heated stir plate and thermo-probe for 20 hours. The reaction mixture was filtered over celite with ethyl acetate, evaporated under reduced pressure. The crude residue was purified by flash chromatography. In cases where anisole was particularly challenging to remove from the final product after the first purification, one additional purification by prep TLC using a 30% DCM/ 70% pentane solution was performed. Each instance of this is noted for the compounds it applies. Yields in triplicate are accurate to reflect this second purification and NMR spectra depict these exact compounds as they are reported. In cases where NMR spectra still contains trace solvent of any kind, the peaks are annotated and represent less than 2% impurity.

(*E*)-2-styryl-2,3-dihydrobenzofuran (3ab)

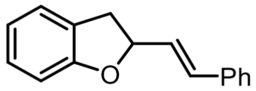

Prepared according to the general procedure A. 2-bromophenol **1a** (58.0 µL, 0.500 mmol, 1.0 equiv.), 1,3-diene **2b (**98.0 µL, 0.750 mmol, 1.5 equiv.), NaO^{t}Bu (53.0 mg, 0.550 mmol, 1.1 equiv.), $Pd(OAc)_2$ (2.8 mg, 0.0125 mmol, 0.025 equiv.), urea **4e** (5.9 mg, 0.025 mmol, 0.05 equiv.), and 90:10 PhMe/PhOMe (2.00 mL, 0.25M) were added. Crude material was purified via column chromatography using 100% hexanes → 5% EtOAc/ 95% hexanes to afford the product as a yellow solid.

Run Number: (isolated yield in mg, isolated yield in mmol, isolated yield in percent)

Run 1: (84.6 mg, 0.380 mmol, 76%)
Run 2: (80.4 mg, 0.360 mmol, 72%)
Run 3: (83.7 mg, 0.375 mmol, 75%)
Average: 74% yield

^{1}H NMR (400 MHz, $CDCl_3$) δ 7.51 (m, 2H), 7.43 (m, 2H), 7.37 (m, 1H), 7.32 – 7.22 (m, 2H), 7.04 – 6.94 (m, 2H), 6.81 (d, *J* = 15.8 Hz, 1H), 6.47 (dd, *J* = 15.8, 7.3 Hz, 1H), 5.50 – 5.39 (dt, J = 15.8 Hz, 9.1 Hz, 1H), 3.51 (dd, *J* = 15.6, 9.2 Hz, 1H), 3.17 (dd, *J* = 15.6, 7.8 Hz, 1H).

^{13}C NMR (126 MHz, $CDCl_3$) δ 159.4, 136.2, 132.1, 128.5 (2C), 128.4, 128.1, 127.9, 126.6 (2C), 126.5, 124.9, 120.5, 109.0, 83.3, 36.2.

HRMS (ESI) m/z calculated for $C_{16}H_{14}O$ $[M+H]^+$ 223.1123, found 223.1116.

(*E*)-4-methyl-2-styryl-2,3-dihydrobenzofuran (3bb)

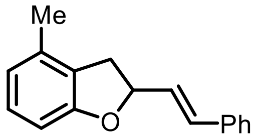

Prepared according to the general procedure A. 2-bromo-3-methyl phenol **1b** (94.0 mg, 0.500 mmol, 1.0 equiv.), 1,3-diene **2b (**98.0 µL, 0.750 mmol, 1.5 equiv.), NaO^{t}Bu (53.0 mg, 0.550 mmol, 1.1 equiv.), $Pd(OAc)_2$ (2.8 mg, 0.0125 mmol, 0.025 equiv.), urea **4e** (5.9 mg, 0.025 mmol, 0.05 equiv.), and 90:10 PhMe/PhOMe (2.00 mL, 0.25M) were added. Crude material was first purified via column chromatography using 100% hexanes → 5% EtOAc/ 95% hexanes to afford the product as a yellow solid. Further purification by prep TLC and 30% DCM/ 70% pentane was performed to remove anisole.

Run Number: (isolated yield in mg, isolated yield in mmol, isolated yield in percent)

Run 1: (82.0 mg, 0.345 mmol, 69%)
Run 2: (85.4 mg, 0.360 mmol, 72%)
Run 3: (80.3 mg, 0.340 mmol, 68%)
Average: 70% yield

^{1}H NMR (400 MHz, $CDCl_3$) δ 7.51 (m, 2H), 7.47 – 7.35 (m, 2H), 7.36 (m, 1H), 7.17 (m, 1H), 6.80 (m, 3H), 6.48 (dd, *J* = 15.8, 7.3 Hz, 1H), 5.46 (dt, *J* = 15.9 Hz, 9.2 Hz, 1H), 3.44 (dd, *J* = 15.5, 9.4 Hz, 1H), 3.08 (dd, *J* = 15.6, 7.7 Hz, 1H), 2.35 (s, 3H).

^{13}C NMR (126 MHz, $CDCl_3$) δ 159.2, 136.3, 134.8, 132.1, 128.7, 128.6 (2C), 128.1, 128.0, 126.7 (2C), 125.6, 121.5, 106.8, 83.3, 37.3, 19.0.

HRMS (ESI) m/z calculated for $C_{17}H_{16}O$ $[M+H]^+$: 237.1279, found 237.1273.

(*E*)-6-methyl-2-styryl-2,3-dihydrobenzofuran (3cb)

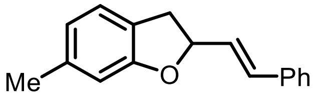

Prepared according to the general procedure A. 2-bromo-5-methyl phenol **1c** (94.0 mg, 0.500 mmol, 1.0 equiv.), 1,3-diene **2b (**98.0 µL, 0.750 mmol, 1.5 equiv.), NaO*t*Bu (53.0 mg, 0.550 mmol, 1.1 equiv.), $Pd(OAc)_2$ (2.8 mg, 0.0125 mmol, 0.025 equiv.), urea **4e** (5.9 mg, 0.025 mmol, 0.05 equiv.), and 90:10 PhMe/PhOMe (2.00 mL, 0.25M) were added. Crude material was purified via column chromatography using 100% hexanes → 5% EtOAc/ 95% hexanes to afford the product as a yellow solid.

<u>Run Number:</u> (isolated yield in mg, isolated yield in mmol, isolated yield in percent)

<u>Run 1:</u> (73.3 mg, 0.310 mmol, 62%)
<u>Run 2:</u> (72.5 mg, 0.310 mmol, 62%)
<u>Run 3:</u> (79.2 mg, 0.33 mmol, 67%)
Average: 64% yield

^{1}H NMR (400 MHz, $CDCl_3$) δ 7.42 (m, 2H), 7.33 (m, 2H), 7.26 (m, 1H) 7.08 (d, *J* = 7.4 Hz, 1H), 6.74 – 6.71 (m, 1H) 6.70 – 6.65 (m, 2H), 6.39 (dd, *J* = 15.8, 7.3 Hz, 1H), 5.37 (dt, *J* = 15.2 Hz, 8.3 Hz, 1H), 3.42 (dd, *J* = 15.4, 9.1 Hz, 1H), 3.06 (dd, *J* = 15.4, 7.7 Hz, 1H), 2.34 (s, 3H).

^{13}C NMR (126 MHz, $CDCl_3$) δ 159.8, 138.4, 136.5, 132.3, 128.8, 128.7 (2C), 128.1, 126.8 (2C), 124.6, 123.7, 121.3, 109.27, 83.2, 36.2, 21.6.

HRMS (ESI) m/z calculated for $C_{17}H_{16}O$ $[M+H]^+$: 237.1279, found 237.1271.

(*E*)-5-methyl-2-styryl-2,3-dihydrobenzofuran (3db)

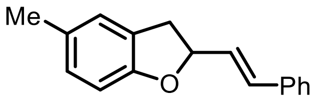

Prepared according to the general procedure A. 2-bromo-4-methyl phenol **1d** (94.0 mg, 0.500 mmol, 1.0 equiv.), 1,3-diene **2b (**98.0 µL, 0.750 mmol, 1.5 equiv.), NaO*t*Bu (53.0 mg, 0.550 mmol, 1.1 equiv.), $Pd(OAc)_2$ (2.8 mg, 0.0125 mmol, 0.025 equiv.), urea **4e** (5.9 mg, 0.025 mmol, 0.05 equiv.), and 90:10 PhMe/PhOMe (2.00 mL, 0.25M) were added. Crude material was purified via column chromatography using 100% hexanes → 5% EtOAc/ 95% hexanes to afford the product as a yellow solid. Further purification by prep TLC and 30% DCM/ 70% pentane was performed to remove anisole.

<u>Run Number:</u> (isolated yield in mg, isolated yield in mmol, isolated yield in percent)

<u>Run 1:</u> (66.3 mg, 0.280 mmol, 56%)
<u>Run 2:</u> (67.7 mg, 0.285 mmol, 57%)

Run 3: (71.0 mg, 0.300 mmol, 60%)
Average: 58% yield

^{1}H NMR (400 MHz, $CDCl_3$) δ 7.42 (m, 2H), 7.34 (m, 2H), 7.32 – 7.23 (m, 1H), 7.02 (s, 1H), 6.96 (m, 1H), 6.78 – 6.68 (m, 2H), 6.39 (dd, *J* = 15.8, 7.3 Hz, 1H), 5.42 – 5.31 (dt, *J* = 15.9 Hz, 8.9 Hz, 1H), 3.42 (dd, *J* = 15.5, 9.1 Hz, 1H), 3.08 (dd, *J* = 15.5, 7.7 Hz, 1H), 2.31 (s, 3H).

^{13}C NMR (126 MHz, $CDCl_3$) δ 157.4, 136.4, 132.3, 129.9, 128.7 (2C), 128.6, 128.5, 128.1, 126.8 (2C), 126.7, 125.6, 109.1, 83.6, 36.5, 20.9.

HRMS (ESI) m/z calculated for $C_{17}H_{16}O$ $[M+H]^+$: 237.1279, found 237.1272.

(*E*)-7-methyl-2-styryl-2,3-dihydrobenzofuran (3eb)

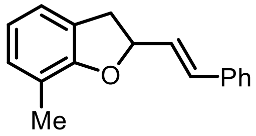

Prepared according to the general procedure A. 2-bromo-6-methyl phenol **1e** (94.0 mg, 0.500 mmol, 1.0 equiv.), 1,3-diene **2b (**98.0 µL, 0.750 mmol, 1.5 equiv.), NaO^{t}Bu (53.0 mg, 0.550 mmol, 1.1 equiv.), $Pd(OAc)_2$ (2.8 mg, 0.0125 mmol, 0.025 equiv.), urea **4e** (5.9 mg, 0.025 mmol, 0.05 equiv.), and 90:10 PhMe/PhOMe (2.00 mL, 0.25M) were added. Crude material was purified via column chromatography using 100% hexanes → 5% EtOAc/ 95% hexanes to afford the product as a yellow solid.

Run Number: (isolated yield in mg, isolated yield in mmol, isolated yield in percent)

Run 1: (80.3mg, 0.340 mmol, 68%)
Run 2: (78.0mg, 0.330 mmol, 66%)
Run 3: (82.7mg, 0.350 mmol. 70%)
Average: 68% yield

^{1}H NMR (400 MHz, $CDCl_3$) δ 7.47 (m, 2H), 7.38 – 7.27 (m, 2H), 7.31 (m, 1H), 6.85 (m, 4H), 6.44 (dd, *J* = 15.8, 7.4 Hz, 1H), 5.46 – 5.35 (dt, J = 15.6 Hz, 7.8 Hz, 1H), 3.49 (dd, *J* = 15.5, 9.2 Hz, 1H), 3.15 (dd, *J* = 15.5, 8.0 Hz, 1H), 2.33 (s, 3H).

^{13}C NMR (101 MHz, $CDCl_3$) δ 157.9, 136.4, 132.2, 129.4, 129.0, 128.8 (2C), 128.1, 126.8 (2C), 125.9, 122.3, 120.5, 119.8, 83.3, 36.7, 15.5.

HRMS (ESI) m/z calculated for $C_{17}H_{16}O$ $[M+H]^+$: 237.1279, found 237.1272.

(*E*)-6-methoxy-2-styryl-2,3-dihydrobenzofuran (3fb)

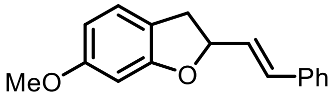

Prepared according to the general procedure A. 2-bromo-5-methoxy phenol **1f** (102 mg, 0.500 mmol, 1.0 equiv.), 1,3-diene **2b (**98.0 µL, 0.750 mmol, 1.5 equiv.), NaO^{t}Bu (53.0 mg, 0.550 mmol, 1.1 equiv.), $Pd(OAc)_2$ (2.8 mg, 0.0125 mmol, 0.025 equiv.), urea **4e** (5.9 mg, 0.025 mmol, 0.05 equiv.), and 90:10 PhMe/PhOMe (2.00 mL, 0.25M) were added. Crude material was purified via column chromatography using 100% hexanes → 5% EtOAc/

95% hexanes to afford the product as a yellow solid. Further purification by prep TLC and 30% DCM/ 70% pentane was performed to remove anisole.

Run Number: (isolated yield in mg, isolated yield in mmol, isolated yield in percent)

Run 1: (58.0 mg, 0.230 mmol, 46%)
Run 2: (55.5 mg, 0.220 mmol, 44%)
Run 3: (57.7 mg, 0.230 mmol, 46%)
Average: 45% yield

^{1}H NMR (400 MHz, $CDCl_3$) δ 7.45 (m, 2H), 7.37 (m, 2H), 7.31 (d, *J* = 7.3 Hz, 1H), 7.09 (d, *J* = 8.0 Hz, 1H), 6.76 – 6.72 (d, J = 15.8 Hz, 1H), 6.48 (m, 2H), 6.41 (dd, *J* = 15.9, 7.3 Hz, 1H), 5.47 – 5.36 (dt, J = 15.8 Hz, 7.7 Hz, 1H), 3.81 (s, 3H), 3.41 (dd, *J* = 15.2, 9.1 Hz, 1H), 3.05 (dd, *J* = 15.6, 8.3 Hz, 1H).

^{13}C NMR (126 MHz, $CDCl_3$) δ 160.7, 160.5, 136.3, 132.3, 128.7 (2C), 128.5, 128.1, 126.5 (2C), 124.9, 118.6, 105.9, 96.1, 84.2, 55.5, 35.7.

HRMS (ESI) m/z calculated for $C_{17}H_{16}O_2$ $[M+H]^+$: 253.1228, found 253.1221.

(*E*)-2-styryl-6-(trifluoromethyl)-2,3-dihydrobenzofuran (3gb)

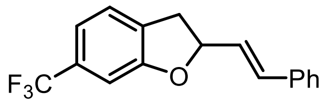

Prepared according to the general procedure A. 2-bromo-5-trifluoromethyl phenol **1g** (112 mg, 0.500 mmol, 1.0 equiv.), 1,3-diene **2b (**98.0 µL, 0.750 mmol, 1.5 equiv.), NaO*t*Bu (53.0 mg, 0.550 mmol, 1.1 equiv.), $Pd(OAc)_2$ (2.8 mg, 0.0125 mmol, 0.025 equiv.), urea **4e** (5.9 mg, 0.025 mmol, 0.05 equiv.), and 90:10 PhMe/PhOMe (2.00 mL, 0.25M) were added. Crude material was purified via column chromatography using 100% hexanes → 5% EtOAc/ 95% hexanes to afford the product as a yellow solid. Further purification by prep TLC and 30% DCM/ 70% pentane was performed to remove anisole.

Run Number: (isolated yield in mg, isolated yield in mmol, isolated yield in percent)

Run 1: (82.7 mg, 0.285 mmol, 57%)
Run 2: (89.7 mg, 0.310 mmol, 62%)
Run 3: (86.0 mg, 0.295 mmol, 59%)
Average: 59% yield

^{1}H NMR (400 MHz, $CDCl_3$) δ 7.43 (m, 2H), 7.36 – 7.33 (m, 2H), 7.30 – 7.26 (m, 2H), 7.15 (d, *J* = 7.7 Hz, 1H), 7.05 (s, 1H), 6.74 (d, *J* = 15.8 Hz, 1H), 6.36 (dd, *J* = 15.8 Hz, 7.3 Hz, 1H), 5.51 – 5.40 (m, J = 15.8 Hz, 7.7 Hz, 1H), 3.50 (dd, *J* = 16.1, 9.3 Hz, 1H), 3.14 (dd, *J* = 16.1, 7.7 Hz, 1H).

^{13}C NMR (126 MHz, $CDCl_3$) δ 159.8, 136.1, 132.9, 131.0, 130.8 (q, $J_{2C\text{-}F}$ = 33.0 Hz, 1C), 128.8 (2C), 128.4, 127.7, 126.9 (2C), 125.4 (q, $J_{1C\text{-}F}$ = 272.3 Hz, 1C), 125.2, 117.8 (q, $J_{3C\text{-}F}$ = 4.3 Hz, 1C), 106.6 (q, $J_{3C\text{-}F}$ = 4.0 Hz, 1C), 84.4, 36.2.

^{19}F NMR (376 MHz, $CDCl_3$) δ -62.3 (3F).

HRMS (ESI) m/z calculated for $C_{17}H_{13}F_3O$ $[M+H]^+$: 291.0996, found 291.0989.

(*E*)-5-methoxy-2-styryl-2,3-dihydrobenzofuran (3hb)

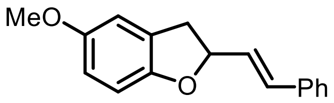

Prepared according to the general procedure A. 2-bromo-4-methoxy phenol **1h** (102 mg, 0.500 mmol, 1.0 equiv.), 1,3-diene **2b (**98.0 µL, 0.750 mmol, 1.5 equiv.), NaO^{t}Bu (53.0 mg, 0.550 mmol, 1.1 equiv.), $Pd(OAc)_2$ (2.8 mg, 0.0125 mmol, 0.025 equiv.), urea **4e** (5.9 mg, 0.025 mmol, 0.05 equiv.), and 90:10 PhMe/PhOMe (2.00 mL, 0.25M) were added. Crude material was purified via column chromatography using 100% hexanes → 5% EtOAc/ 95% hexanes to afford the product as a yellow solid. Further purification by prep TLC and 30% DCM/ 70% pentane was performed to remove anisole.

<u>Run Number:</u> (isolated yield in mg, isolated yield in mmol, isolated yield in percent)

<u>Run 1:</u> (75.7 mg, 0.300 mmol, 60%)
<u>Run 2:</u> (72.0 mg, 0.285 mmol, 57%)
<u>Run 3:</u> (70.6 mg, 0.280 mmol, 56%)
Average: 58% yield

^1H NMR (400 MHz, $CDCl_3$) δ 7.42 – 7.40 (m, 2H), 7.35 – 7.31 (m, 2H), 7.25 (m, 1H), 6.87 – 6.63 (m, 4H), 6.38 (dd, *J* = 15.8, 7.3 Hz, 1H), 5.40 – 5.29 (dt, J = 15.9 Hz, 7.8 Hz, 1H), 3.77 (s, 3H), 3.42 (dd, *J* = 15.5, 8.7 Hz, 1H), 3.08 (dd, *J* = 16.4, 8.5 Hz, 1H).

^{13}C NMR (126 MHz, $CDCl_3$) δ 154.3, 153.6, 136.4, 132.3 128.7 (2C), 128.6, 128.1, 127.8, 126.8 (2C), 113.1, 111.4, 109.4, 83.8, 56.2, 36.9.

HRMS (ESI) m/z calculated for $C_{17}H_{16}O_2$ $[M+H]^+$: 253.1228, found 253.1228.

(*E*)-2-styryl-5-(trifluoromethyl)-2,3-dihydrobenzofuran (3ib)

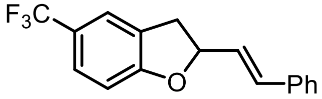

Prepared according to the general procedure A. 2-bromo-4-trifluoromethyl phenol **1i** (112 mg, 0.500 mmol, 1.0 equiv.), 1,3-diene **2b (**98.0 µL, 0.750 mmol, 1.5 equiv.), NaO^{t}Bu (53.0 mg, 0.550 mmol, 1.1 equiv.), $Pd(OAc)_2$ (2.8 mg, 0.0125 mmol, 0.025 equiv.), urea **4e** (5.9 mg, 0.025 mmol, 0.05 equiv.), and 90:10 PhMe/PhOMe (2.00 mL, 0.25M) were added. Crude material was purified via column chromatography using 100% hexanes → 5% EtOAc/ 95% hexanes to afford the product as a yellow solid.

<u>Run Number:</u> (isolated yield in mg, isolated yield in mmol, isolated yield in percent)

<u>Run 1:</u> n.r.
<u>Run 2:</u> n.r.
<u>Run 3:</u> n.r.
Average: 0%, n.r.

(*E*)-5-chloro-2-styryl-2,3-dihydrobenzofuran (3jb)

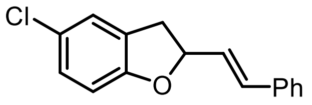

Prepared according to the general procedure A. 2-bromo-4-chloro phenol **1j** (104 mg, 0.500 mmol, 1.0 equiv.), 1,3-diene **2b** **(**98.0 µL, 0.750 mmol, 1.5 equiv.), NaO^{t}Bu (53.0 mg, 0.550 mmol, 1.1 equiv.), $Pd(OAc)_2$ (2.8 mg, 0.0125 mmol, 0.025 equiv.), urea **4e** (5.9 mg, 0.025 mmol, 0.05 equiv.), and 90:10 PhMe/PhOMe (2.00 mL, 0.25M) were added. Crude material was purified via column chromatography using 100% hexanes → 5% EtOAc/ 95% hexanes to afford the product as a yellow solid.

<u>Run Number:</u> (isolated yield in mg, isolated yield in mmol, isolated yield in percent)

<u>Run 1:</u> (98.1 mg, 0.381 mmol, 77%)
<u>Run 2:</u> (95.7 mg, 0.373 mmol, 75%)
<u>Run 3:</u> (96.2 mg, 0.375 mmol, 75%)
Average Yield: 76%

^{1}H NMR (400 MHz, $CDCl_3$) δ 7.44 (m, 2H), 7.38 – 7.35 (m, 2H), 7.30 (m, 1H), 7.17 (s, 1H), 7.13 (m, 1H) 6.81 – 6.68 (m, 2H), 6.38 (dd, *J* = 15.8, 7.3 Hz, 1H), 5.43 – 5.37 (dt, J = 15.8 Hz, 7.7 Hz, 1H), 3.43 (dd, *J* = 15.8, 9.3 Hz, 1H), 3.09 (dd, *J* = 15.9, 7.9 Hz, 1H).

^{13}C NMR (126 MHz, $CDCl_3$) δ 158.2, 136.1, 132.7, 128.7 (2C), 128.6, 128.2, 128.0, 127.9, 126.8 (2C), 125.2, 125.1, 110.4, 84.2, 36.3.

HRMS (ESI) m/z calculated for $C_{16}H_{13}ClO$ $[M+H]^+$: 257.0733, found 257.0725.

(*E*)-5-fluoro-2-styryl-2,3-dihydrobenzofuran (3kb)

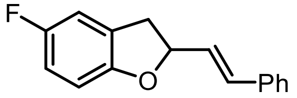

Prepared according to the general procedure A. 2-bromo-4-fluorophenol **1k** (96.0 mg, 0.500 mmol, 1.0 equiv.), 1,3-diene **2b** **(**98.0 µL, 0.750 mmol, 1.5 equiv.), NaO^{t}Bu (53.0 mg, 0.550 mmol, 1.1 equiv.), $Pd(OAc)_2$ (2.8 mg, 0.0125 mmol, 0.025 equiv.), urea **4e** (5.9 mg, 0.025 mmol, 0.05 equiv.), and 90:10 PhMe/PhOMe (2.00 mL, 0.25M) were added. Crude material was purified via column chromatography using 100% hexanes → 5% EtOAc/ 95% hexanes to afford the product as a yellow solid. Further purification by prep TLC and 30% DCM/ 70% pentane was performed to remove anisole.

<u>Run Number:</u> (isolated yield in mg, isolated yield in mmol, isolated yield in percent)

<u>Run 1:</u> (75.7 mg, 0.315 mmol, 63%)
<u>Run 2:</u> (79.2 mg, 0.330 mmol, 66%)
<u>Run 3:</u> (75.1 mg, 0.313 mmol, 63%)
Average Yield: 64%

^{1}H NMR (400 MHz, $CDCl_3$) δ 7.41 (m, 2H), 7.35 – 7.31 (m, 2H), 7.27 – 7.25 (m, 1H), 6.90 (m, 1H), 6.81 (m, 1H), 6.76 – 6.67 (m, 2H), 6.36 (dd, *J* = 15.9, 7.3 Hz, 1H), 5.44 (dt, *J* = 15.8 Hz. 7.8 Hz, 1H), 3.43 (dd, *J* = 15.8, 9.1 Hz, 1H), 3.09 (dd, *J* = 15.8, 7.9 Hz, 1H).

^{13}C NMR (126 MHz, $CDCl_3$, Fluorine not decoupled) δ 158.6 (d, J_1C_F = 236.9 Hz, 1C), 155.5, 136.3, 132.6, 128.8 (2 C's), 128.3, 128.1, 126.8 (2 C's), 114.8 (1C, d, J_2C_F = 24.1 Hz), 112.2 (d, J_2C_F = 25.0 Hz, 1C), 109.6, 109.5, 84.2, 36.7.

^{19}F NMR (376 MHz, $CDCl_3$) δ -124.5 (1F).

HRMS (ESI) m/z calculated for $C_{16}H_{13}FO$ $[M+H]^+$: 241.1028, found 241.1024

(*E*)-1-(2-styryl-2,3-dihydrobenzofuran-5-yl)piperidine (3lb)

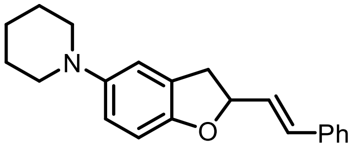

Prepared according to the general procedure A. 2-bromo-4-(piperidin-1-yl) phenol **1l** (128 mg, 0.500 mmol, 1.0 equiv.), 1,3-diene **2b (**98.0 µL, 0.750 mmol, 1.5 equiv.), NaO^{t}Bu (53.0 mg, 0.550 mmol, 1.1 equiv.), $Pd(OAc)_2$ (2.8 mg, 0.0125 mmol, 0.025 equiv.), urea **4e** (5.9 mg, 0.025 mmol, 0.05 equiv.), and 90:10 PhMe/PhOMe (2.00 mL, 0.25M) were added. Crude material was purified via column chromatography using 100% hexanes → 5% EtOAc/ 95% hexanes to afford the product as a yellow solid.

Run Number: (isolated yield in mg, isolated yield in mmol, isolated yield in percent)

Run 1: (49.3 mg, 0.160 mmol, 32%)
Run 2: (49.9 mg, 0.165 mmol, 33%)
Run 3: (56.0 mg, 0.185 mmol, 37%)
Average: 34% yield

^1H NMR (400 MHz, $CDCl_3$) δ 7.40 (m, 2H), 7.32 (m, 2H), 7.26 (m, 1H), 6.88 (s, 1H), 6.78 – 6.67 (m, 3H), 6.37 (dd, *J* = 15.8, 7.4 Hz, 1H), 5.33 (dt, J = 15.9 Hz, 7.7 Hz, 1H), 3.40 (dd, *J* = 15.5, 9.1 Hz, 1H), 3.11 – 3.01 (dd, J =15.5 Hz, 7.8 Hz, 1H), 3.00 (m, 4H), 1.73 (m, 4H), 1.54 (m, 2H).

^{13}C NMR (126 MHz, $CDCl_3$) δ 153.8, 147.6, 136.5, 132.2, 128.8, 128.7 (2C), 128.1, 127.3, 126.8 (2C), 117.9, 115.7, 109.3, 83.7, 53.2 (2C), 40.0, 26.4 (2C), 24.3.

HRMS (ESI) m/z calculated for $C_{21}H_{23}NO$ $[M+H]^+$: 306.1858, found 306.1846.

Isopentyl (*E*)-2-styryl-2,3-dihydrobenzofuran-6-carboxylate (3mb)

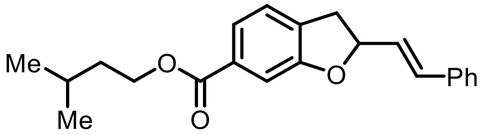

Prepared according to the general procedure A. Isopentyl 4-bromo-3-hydroxybenzoate **1m** (144 mg, 0.500 mmol, 1.0 equiv.), 1,3-diene **2b (**98.0 µL, 0.750 mmol, 1.5 equiv.), NaO^{t}Bu (53.0 mg, 0.550 mmol, 1.1 equiv.), $Pd(OAc)_2$ (2.8 mg, 0.0125 mmol, 0.025 equiv.), urea **4e** (5.9 mg, 0.025 mmol, 0.05 equiv.), and 90:10 PhMe/PhOMe (2.00 mL, 0.25M) were added. Crude material was purified via column chromatography using 100% hexanes → 5% EtOAc/ 95% hexanes to afford the product as a yellow solid.

Run Number: (isolated yield in mg, isolated yield in mmol, isolated yield in percent)

Run 1: (109 mg, 0.320 mmol, 65%)
Run 2: (117 mg, 0.345 mmol, 69%)
Run 3: (104 mg, 0.310 mmol, 62%)
Average: 65% yield
*** reaction with this substrate less reproducible***

^{1}H NMR (400 MHz, $CDCl_3$) δ 7.64 (m, 1H), 7.50 (s, 1H), 7.43 – 7.41 (m, 2H), 7.36 – 7.32 (m, 2H), 7.29 (m, 1H), 7.23 (m, 1H), 6.73 (d, *J* = 15.8 Hz, 1H), 6.36 (dd, *J* = 15.9, 7.2 Hz, 1H), 5.48 – 5.37 (m, 1H), 4.36 (dt, *J* = 15.8 Hz, 7.7 Hz, 2H), 3.48 (dd, *J* = 16.3, 9.2 Hz, 1H), 3.12 (dd, *J* = 16.3, 7.7 Hz, 1H), 1.82 (m, 1H), 1.68 (m, 2H), 1.01 (d, J = 6.7 Hz, 6H).

^{13}C NMR (126 MHz, $CDCl_3$) δ 166.6, 159.7, 136.2, 132.7, 132.3, 131.1, 128.8 (2C), 128.3, 128.0, 126.8 (2C), 124.7, 122.7, 110.3, 84.0, 63.7, 37.5, 36.4, 25.3, 22.6 (2C).

HRMS (ESI) m/z calculated for $C_{22}H_{24}O_3$ $[M+H]^+$: 337.1803, found 337.1792.

(*E*)-5-methyl-2-styryl-2,3-dihydrofuro[3,2-*b*]pyridine (3nb)

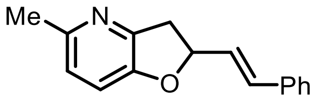

Prepared according to the general procedure A. 2-bromo-6-methylpyridin-3-ol **1n** (94.0 mg, 0.500 mmol, 1.0 equiv.), 1,3-diene **2b (**98.0 µL, 0.750 mmol, 1.5 equiv.), NaO^{t}Bu (53.0 mg, 0.550 mmol, 1.1 equiv.), $Pd(OAc)_2$ (2.8 mg, 0.0125 mmol, 0.025 equiv.), urea **4e** (5.9 mg, 0.025 mmol, 0.05 equiv.), and 90:10 PhMe/PhOMe (2.00 mL, 0.25M) were added. Crude material was purified via column chromatography using 100% hexanes → 5% EtOAc/ 95% hexanes to afford the product as a yellow solid.

Run Number: (isolated yield in mg, isolated yield in mmol, isolated yield in percent)

Run 1: (77.3 mg, 0.325 mmol, 65%)
Run 2: (78.0 mg, 0.330 mmol, 66%)
Run 3: (76.9 mg, 0.325 mmol, 65%)
Average: 65% yield

^{1}H NMR (400 MHz, $CDCl_3$) δ 7.43 – 7.39 (m, 2H), 7.34 – 7.30 (m, 2H), 7.28 – 7.24 (m, 1H), 6.97 (d, J = 8.9 Hz, 1H), 6.87 (d, J = 8.2 Hz, 1H), 6.72 (d, *J* = 15.8 Hz, 1H), 6.36 (dd, *J* = 15.8, 7.4 Hz, 1H), 5.48 – 5.37 (dt, J = 15.9 Hz, 7.8 Hz, 1H), 3.52 (dd, *J* = 16.5, 9.4 Hz, 1H), 3.18 (dd, *J* = 16.5, 7.9 Hz, 1H), 2.48 (s, 3H).

^{13}C NMR (126 MHz, $CDCl_3$) δ 151.2, 150.1, 149.6, 136.0, 133.0, 128.7 (2C), 128.3, 127.8, 126.8 (2C), 121.7, 116.1, 83.0, 37.6, 23.4.

HRMS (ESI) m/z calculated for $C_{16}H_{15}NO$ $[M+H]^+$: 238.1232, found found 238.1226.

1,3-Diene Scope

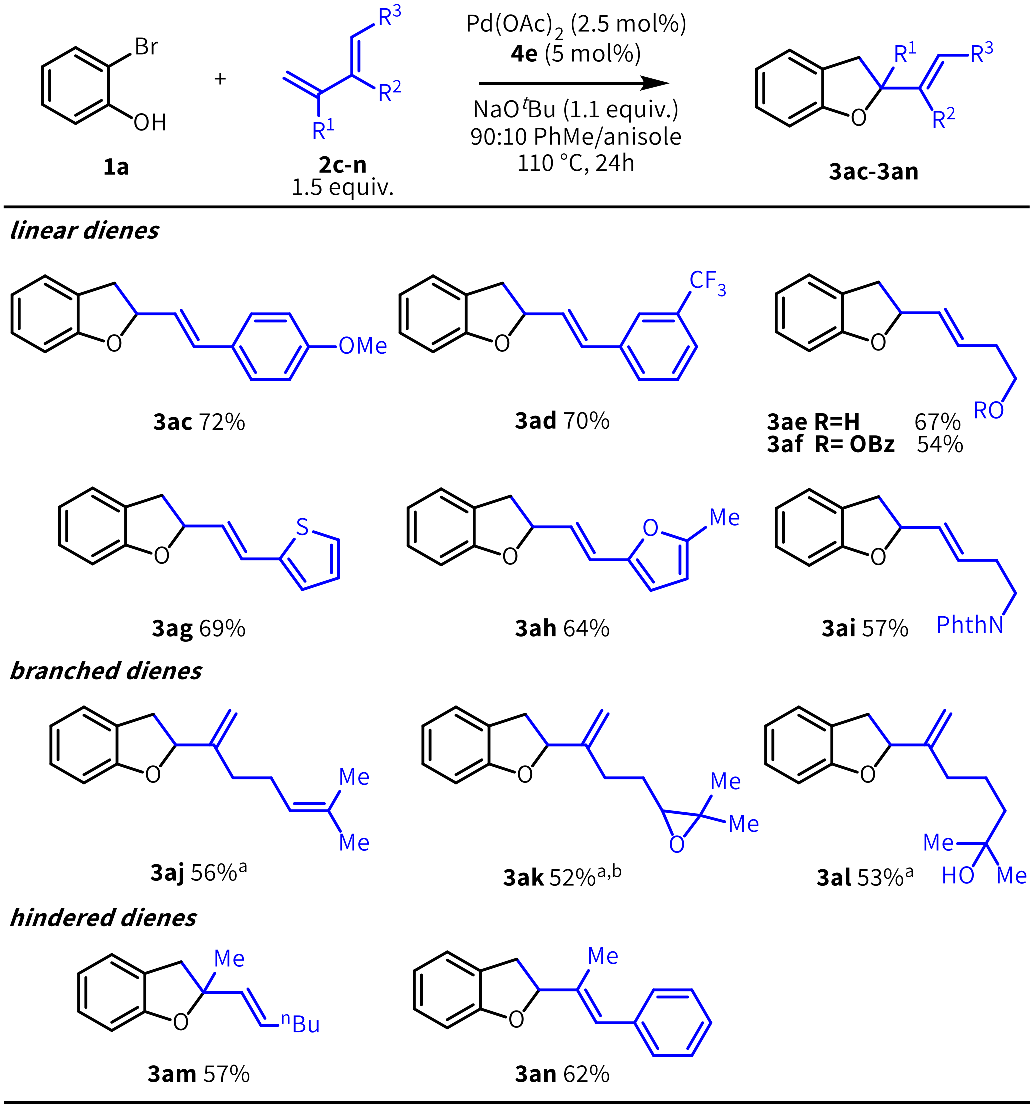

Legend: a) 2.0 equiv. diene used. b) Reaction ran for 48h.

General procedure B: All reactions were run at 0.500 mmol scale. To a 2-dram vial, a 0.0625M stock solution of $Pd(OAc)_2$ in anisole (0.200 mL) and PhMe (1.80 mL) were added. *N*-(4-methoxyphenyl)piperidine-1-carboxamide **4e** (5.9 mg, 0.025 mmol) was added. After 20 min of rapidly stirring, 2-bromophenol **1a** (58.0 µL, 0.500 mmol, 1.0 equiv.) and diene (1.5-2.0 equiv.) were added, followed by NaO^{t}Bu (52.9 mg, 0.550 mmol, 1.1 equiv.). The reaction was stirred at 110°C in an aluminum block with 2-dram slots over a heated stir plate and thermo-probe for 24 hours. The reaction mixture was filtered over celite with ethyl acetate, evaporated under reduced pressure. The crude residue was purified by flash chromatography.

(*E*)-2-(4-methoxystyryl)-2,3-dihydrobenzofuran (3ac)

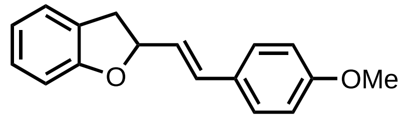

Prepared according to general procedure B. 2-bromophenol **1a** (58.0 μL, 0.500 mmol, 1.0 equiv.), (*E*)-1-(buta-1,3-dien-1-yl)-4-methoxybenzene **2c** (120 mg, 0.750 mmol, 1.5 equiv.), NaO^{t}Bu (52.9 mg, 0.550 mmol, 1.1 equiv.), $Pd(OAc)_2$ (2.8 mg, 0.0125 mmol, 0.025 equiv.), urea **4e** (5.9 mg, 0.025 mmol, 0.05 equiv.), and 90:10 PhMe/PhOMe (2.00 mL, 0.25M) were used. Crude material was purified via column chromatography on 45 g pf SiO_2 using 1:1 hexanes/PhMe to afford product as a light green solid. (>95:5 *E/Z*)

<u>Run Number:</u> (isolated yield in mg, isolated yield in mmol, isolated yield in percent)

<u>Run 1:</u> (88.4 mg, 0.350 mmol, 70%)
<u>Run 2:</u> (90.8 mg, 0.360 mmol, 72%)
<u>Run 3:</u> (92.0 mg, 0.365 mmol, 73%)
Average: 72% yield

^{1}H NMR (400 MHz, $CDCl_3$) δ 7.39 (d, *J* = 8.2 Hz, 2H), 7.29 – 7.12 (m, 2H), 6.97 – 6.83 (m, 4H), 6.70 (d, *J* = 15.7 Hz, 1H), 6.28 (dd, *J* = 15.8, 7.6 Hz, 1H), 5.37 (td, *J* = 8.2, 8.2 Hz, 1H), 3.83 (s, 3H), 3.45 (dd, *J* = 15.6, 9.1 Hz, 1H), 3.12 (dd, *J* = 15.6, 7.9 Hz, 1H).

^{13}C NMR (101 MHz, $CDCl_3$) δ 159.6, 159.5, 132.1, 129.0, 128.2, 128.0 (2C), 126.8, 126.2, 125.0, 120.5, 114.1 (2C), 109.5, 83.9, 55.3, 36.4.

HRMS (ESI) *m/z* calculated for $C_{17}H_{16}O_2$ $[M+H]^+$: 253.1228, found 253.1221.

(*E*)-2-(4-(trifluoromethyl)styryl)-2,3-dihydrobenzofuran (3ad)

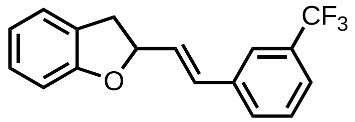

Prepared according to general procedure B. 2-bromophenol **1a** (58.0 μL, 0.500 mmol, 1.0 equiv.), (*E*)-1-(buta-1,3-dien-1-yl)-4-(trifluoromethyl)benzene **2d** (149 mg, 0.750 mmol, 1.5 equiv.), NaO^{t}Bu (52.9 mg, 0.550 mmol, 1.1 equiv.), $Pd(OAc)_2$ (2.8 mg, 0.0125 mmol, 0.025 equiv.), urea **4e** (5.9 mg, 0.025 mmol, 0.05 equiv.), and 90:10 PhMe/PhOMe (2.00 mL, 0.25M) were used. Crude material was purified via column chromatography on 45 g of SiO_2 using 97:3 hexanes/EtOAc to afford product as a yellow liquid. (>95:5 *E/Z*)

<u>Run Number:</u> (isolated yield in mg, isolated yield in mmol, isolated yield in percent)

<u>Run 1:</u> (104.3 mg, 0.359 mmol, 72%)
<u>Run 2:</u> (105.2 mg, 0.362 mmol, 72%)
<u>Run 3:</u> (97.8 mg, 0.337 mmol, 67%)
Average: 70% yield

^{1}H NMR (400 MHz,) δ 7.68 (s, 1H), 7.58 (d, *J* = 7.7 Hz, 1H), 7.54 (d, *J* = 7.9 Hz, 1H), 7.50 – 7.41 (m, 1H), 7.26 – 7.15 (m, 2H), 6.96 – 6.81 (m, 2H), 6.77 (d, *J* = 16.0 Hz, 1H), 6.47

(dd, J = 15.9, 6.9 Hz, 1H), 5.41 (td, J = 7.8, 7.8 Hz, 1H), 3.50 (dd, J = 15.5, 9.2 Hz, 1H), 3.13 (dd, J = 15.5, 7.5 Hz, 1H).

^{13}C NMR (101 MHz, $CDCl_3$) δ 159.4, 137.2, 130.6 (2C), 129.9, 129.2, 128.3, 126.4, 125.1, 124.6 (2C), 123.4 (2C), 120.8, 109.6, 82.9, 36.3.

^{19}F NMR (376 MHz, $CDCl_3$) δ -62.9 (3F).

HRMS (ESI) *m/z* calculated for $C_{17}H_{13}F_3O$ $[M+H]^+$: 291.0996, found 291.0989.

(*E*)-4-(2,3-dihydrobenzofuran-2-yl)but-3-en-1-ol (3ae)

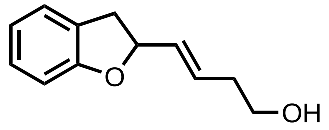

Prepared according to general procedure B. 2-bromophenol **1a** (58.0 µL, 0.500 mmol, 1.0 equiv.), (*E*)-hexa-3,5-dien-1-ol **2e** (73.6 mg, 0.750 mmol, 1.5 equiv.), NaO^{t}Bu (52.9 mg, 0.550 mmol, 1.1 equiv.), $Pd(OAc)_2$ (2.8 mg, 0.0125 mmol, 0.025 equiv.), urea **4e** (5.9 mg, 0.025 mmol, 0.05 equiv.), and 90:10 PhMe/PhOMe (2.00 mL, 0.25M) were used. Crude material was purified via column chromatography on 45 g of SiO_2 using 75:25 hexanes/EtOAc to afford product as a tan oil. Trace impurities, based on internal standard, compound is 99% pure. (>95:5 *E/Z*)

<u>Run Number:</u> (isolated yield in mg, isolated yield in mmol, isolated yield in percent)

<u>Run 1:</u> (65.0 mg, 0.342 mmol, 68%)
<u>Run 2:</u> (65.5 mg, 0.344 mmol, 69%)
<u>Run 3:</u> (61.3 mg, 0.322 mmol, 64%)
Average: 67% yield

^{1}H NMR (400 MHz, $CDCl_3$) δ 7.19 – 7.07 (m, 2H), 6.85 (dd, J = 7.4, 7.4 Hz, 1H), 6.78 (d, J = 7.9 Hz, 1H), 5.91 – 5.73 (m, 2H), 5.17 (td, J = 8.5, 6.6 Hz, 1H), 3.69 (t, J = 6.4 Hz, 2H), 3.34 (dd, J = 15.6, 9.2 Hz, 1H), 2.99 (dd, J = 15.6, 8.1 Hz, 1H), 2.35 (td, J = 6.3, 6.3 Hz, 2H), 2.20 (s, 1H).

^{13}C NMR (101 MHz, $CDCl_3$) δ 159.4, 132.3, 130.4, 128.2, 126.8, 125.0, 120.6, 109.5, 83.5, 61.9, 36.2, 35.7.

HRMS (ESI) *m/z* calculated for $C_{12}H_{14}O_2$ $[M+H]^+$: 191.1072, found 191.1064.

(*E*)-4-(2,3-dihydrobenzofuran-2-yl)but-3-en-1-yl benzoate (3af)

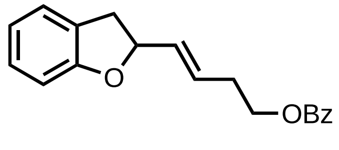

Prepared according to general procedure B. 2-bromophenol **1a** (58.0 µL, 0.500 mmol, 1.0 equiv.), (*E*)-hexa-3,5-dien-1-yl benzoate **2f** (152 mg, 0.750 mmol, 1.5 equiv.), NaO^{t}Bu (52.9 mg, 0.550 mmol, 1.1 equiv.), $Pd(OAc)_2$ (2.8 mg, 0.0125 mmol, 0.025 equiv.), urea **4e** (5.9 mg, 0.025 mmol, 0.05 equiv.), and 90:10 PhMe/PhOMe (2.00 mL, 0.25M) were used. Crude material was purified via column chromatography on 45 g of SiO_2 using 97:3 hexanes/EtOAc to afford product as a light-

yellow oil. Trace impurities, based on internal standard, compound is 99% pure. (>95:5 *E/Z*)

Run Number: (isolated yield in mg, isolated yield in mmol, isolated yield in percent)

Run 1: (78.7 mg, 0.267 mmol, 53%)
Run 2: (80.2 mg, 0.272 mmol, 54%)
Run 3: (80.9 mg, 0.275 mmol, 55%)
Average: 54% yield

^{1}H NMR (400 MHz, $CDCl_3$) δ 8.11 – 8.02 (m, 2H), 7.61 – 7.53 (m, 1H), 7.50 – 7.41 (m, 2H), 7.18 – 7.08 (m, 2H), 6.89 – 6.82 (m, 1H), 6.82 – 6.76 (m, 1H), 5.97 – 5.78 (m, 2H), 5.18 (td, *J* = 8.0, 8.0 Hz, 1H), 4.40 (t, *J* = 6.5 Hz, 2H), 3.34 (dd, *J* = 15.5, 9.1 Hz, 1H), 2.96 (dd, *J* = 15.5, 7.9 Hz, 1H), 2.57 (td, *J* = 6.6, 6.6 Hz, 2H).

^{13}C NMR (101 MHz, $CDCl_3$) δ 166.6, 159.4, 133.0, 132.3, 130.4, 129.7 (2C), 129.3, 128.5 (2C), 128.2, 126.7, 125.0, 120.5, 109.5, 83.3, 63.9, 36.2, 31.8.

HRMS (ESI) *m/z* calculated for $C_{19}H_{18}O_3$ $[M+H]^+$: 295.1334, found 295.1327.

(*E*)-2-(2-(thiophen-2-yl)vinyl)-2,3-dihydrobenzofuran (3ag)

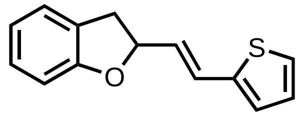

Prepared according to general procedure B. 2-bromophenol **1a** (58.0 µL, 0.500 mmol, 1.0 equiv.), 2-(buta-1,3-dien-1-yl)thiophene **2g** (102 mg, 0.750 mmol, 1.5 equiv.), NaO^{t}Bu (52.9 mg, 0.550 mmol, 1.1 equiv.), $Pd(OAc)_2$ (2.8 mg, 0.0125 mmol, 0.025 equiv.), urea **4e** (5.9 mg, 0.025 mmol, 0.05 equiv.), and 90:10 PhMe/PhOMe (2.00 mL, 0.25M) were used. Crude material was purified via column chromatography on 45 g of SiO_2 using 8:2 hexanes/PhMe to afford product as a yellow solid. (>95:5 *E/Z*)

Run Number: (isolated yield in mg, isolated yield in mmol, isolated yield in percent)

Run 1: (79.6 mg, 0.349 mmol, 70%)
Run 2: (80.4 mg, 0.352 mmol, 70%)
Run 3: (77.2 mg, 0.338 mmol, 68%)
Average: 69% yield

^{1}H NMR (400 MHz, $CDCl_3$) δ 7.25 – 7.14 (m, 3H), 7.06 – 6.97 (m, 2H), 6.95 – 6.87 (m, 2H), 6.86 (d, *J* = 5.0 Hz, 1H), 6.24 (dd, *J* = 15.6, 7.1 Hz, 1H), 5.35 (td, *J* = 8.0, 8.0 Hz, 1H), 3.46 (dd, *J* = 15.5, 9.2 Hz, 1H), 3.11 (dd, *J* = 15.6, 7.7 Hz, 1H).

^{13}C NMR (101 MHz, $CDCl_3$) δ 159.4, 141.4, 128.2, 127.9, 127.5, 126.6, 126.6, 125.4, 125.0, 124.9, 120.6, 109.5, 83.1, 36.3.

HRMS (ESI) *m/z* calculated for $C_{14}H_{12}OS$ $[M+H]^+$: 229.0687, found 229.0680.

(E)-2-(2-(5-methylfuran-2-yl)vinyl)-2,3-dihydrobenzofuran (3ah)

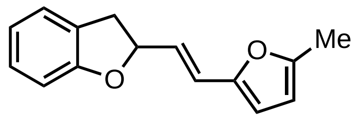

Prepared according to general procedure B. 2-bromophenol **1a** (58.0 µL, 0.500 mmol, 1.0 equiv.), (*E*)-2-(buta-1,3-dien-1-yl)-5-methylfuran **2h** (101 mg, 0.750 mmol, 1.5 equiv.), NaO^{t}Bu (52.9 mg, 0.550 mmol, 1.1 equiv.), $Pd(OAc)_2$ (2.8 mg, 0.0125 mmol, 0.025 equiv.), urea **4e** (5.9 mg, 0.025 mmol, 0.05 equiv.), and 90:10 PhMe/PhOMe (2.00 mL, 0.25M) were used. Purification by flash column chromatography on 45 g SiO_2 using 7:3 hexanes/PhMe. Pure product was isolated as a yellow solid. (>95:5 *E/Z*)

Run Number: (isolated yield in mg, isolated yield in mmol, isolated yield in percent)

Run 1: (72.0 mg, 0.318 mmol, 64%)
Run 2: (69.7 mg, 0.308 mmol, 62%)
Run 3: (75.7 mg, 0.335 mmol, 67%)
Average: 64% yield

^{1}H NMR (400 MHz, $CDCl_3$) δ 7.23 – 7.07 (m, 2H), 6.93 – 6.76 (m, 2H), 6.48 (d, *J* = 15.8 Hz, 1H), 6.25 (dd, *J* = 15.7, 7.1 Hz, 1H), 6.19 (d, *J* = 3.2 Hz, 1H), 6.01 – 5.96 (m, 1H), 5.33 (td, *J* = 7.8, 7.8 Hz, 1H), 3.44 (dd, *J* = 15.5, 9.1 Hz, 1H), 3.09 (dd, *J* = 15.5, 7.6 Hz, 1H), 2.32 (s, 3H).

^{13}C NMR (101 MHz, $CDCl_3$) δ 159.5, 152.5, 150.5, 128.2, 126.7, 125.3, 125.0, 120.5 (2C), 110.3, 109.5, 107.6, 83.3, 36.4, 13.8.

HRMS (ESI) *m/z* calculated for $C_{15}H_{14}O_2$ $[M+H]^+$: 227.1072, found 227.1065.

(*E*)-2-(4-(2,3-dihydrobenzofuran-2-yl)but-3-en-1-yl)isoindoline-1,3-dione (3ai)

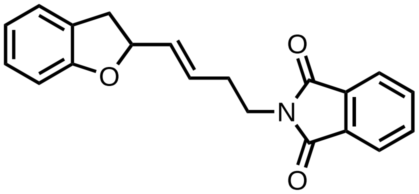

Prepared according to general procedure B. 2-bromophenol **1a** (58.0 µL, 0.500 mmol, 1.0 equiv.), (*E*)-2-(hexa-3,5-dien-1-yl)isoindoline-1,3-dione **2i** (170 mg, 0.750 mmol, 1.5 equiv.), NaO^{t}Bu (52.9 mg, 0.550 mmol, 1.1 equiv.), $Pd(OAc)_2$ (2.8 mg, 0.0125 mmol, 0.025 equiv.), urea **4e** (5.9 mg, 0.025 mmol, 0.05 equiv.), and 90:10 PhMe/PhOMe (2.00 mL, 0.25M) were used. Crude material was purified via column chromatography on 45 g of SiO_2 using 17:3 hexanes/EtOAc to afford product as a light-yellow oil. Trace impurities, based on internal standard, compound is 99% pure. (>95:5 *E/Z*)

Run Number: (isolated yield in mg, isolated yield in mmol, isolated yield in percent)

Run 1: (92.3 mg, 0.289 mmol, 58%)
Run 2: (86.1 mg, 0.270 mmol, 54%)
Run 3: (94.8 mg, 0.297 mmol, 59%)
Average: 57% yield

^{1}H NMR (400 MHz, $CDCl_3$) δ 7.89 – 7.77 (m, 2H), 7.76 – 7.65 (m, 2H), 7.11 – 6.97 (m, 2H), 6.83 – 6.75 (m, 1H), 6.70 (d, *J* = 7.9 Hz, 1H), 5.80 (dt, *J* = 15.4, 6.7 Hz, 1H), 5.70 (dd, *J* = 15.3, 7.0 Hz, 1H), 5.09 (td, *J* = 8.9, 8.4 Hz, 1H), 3.77 (t, *J* = 7.2 Hz, 2H), 3.25 (dd, *J* = 15.5, 9.2 Hz, 1H), 2.81 (dd, *J* = 15.5, 7.8 Hz, 1H), 2.47 (dt, *J* = 7.1, 2.1 Hz, 2H).

^{13}C NMR (101 MHz, $CDCl_3$) δ 168.3 (2C), 159.3, 134.0 (2C), 132.4, 132.1 (2C), 129.6, 128.0, 126.6, 124.8, 123.3 (2C), 120.4, 109.4, 83.1, 37.2, 36.0, 31.3.

HRMS (ESI) *m/z* calculated for $C_{20}H_{17}NO_3$ $[M+H]^+$: 320.1286, found 320.1276.

2-(6-methylhepta-1,5-dien-2-yl)-2,3-dihydrobenzofuran (3aj)

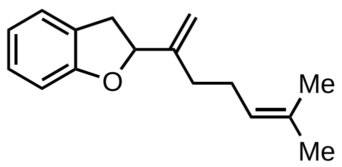

Prepared according to general procedure B. 2-bromophenol **1a** (58.0 μL, 0.500 mmol, 1.0 equiv.), myrcene **2j** (172 μL, 1.00 mmol, 2.0 equiv.), NaO^{t}Bu (52.9 mg, 0.550 mmol, 1.1 equiv.), $Pd(OAc)_2$ (2.8 mg, 0.0125 mmol, 0.025 equiv.), urea **4e** (5.9 mg, 0.025 mmol, 0.05 equiv.), and 90:10 PhMe/PhOMe (2.00 mL, 0.25M) were used. Crude material was purified via column chromatography on 25 g of SiO_2 using 97:3 hexanes/ethyl acetate to afford product as a pale-yellow liquid. Yields are reported as a mixture of isomers (84:16 r.r.).

<u>Run Number:</u> (isolated yield in mg, isolated yield in mmol, isolated yield in percent)

<u>Run 1:</u> (61.8 mg, 0.271 mmol, 54%)
<u>Run 2:</u> (64.9 mg, 0.284 mmol, 57%)
<u>Run 3:</u> (65.2 mg, 0.286 mmol, 57%)
Average: 56% yield

^{1}H NMR (400 MHz, $CDCl_3$) δ 7.20 – 7.10 (m, 2H), 6.90 – 6.80 (m, 2H), 5.26 – 5.10 (m, 3H), 4.97 (s, 1H), 3.37 (dd, *J* = 15.6, 9.5 Hz, 1H), 3.08 (dd, *J* = 15.6, 8.3 Hz, 1H), 2.27 – 2.20 (m, 2H), 2.20 – 2.04 (m, 2H), 1.71 (s, 3H), 1.64 (s, 3H).

^{13}C NMR (101 MHz, $CDCl_3$) δ 159.8, 148.2, 132.1, 128.1, 126.7, 124.9, 124.0, 120.5, 110.5, 109.4, 85.4, 35.4, 31.2, 26.5, 25.8, 17.9.

HRMS (ESI) *m/z* calculated for $C_{16}H_{20}O$ $[M+H]^+$: 229.1592, found 229.1584.

2-(4-(3,3-dimethyloxiran-2-yl)but-1-en-2-yl)-2,3-dihydrobenzofuran (3ak)

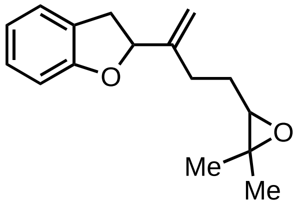

Prepared according to general procedure B. 2-bromophenol **1a** (58.0 μL, 0.500 mmol, 1.0 equiv.), 6,7-epoxymyrcene **2k** (152 mg, 1.00 mmol, 2.0 equiv.), NaO^{t}Bu (52.9 mg, 0.550 mmol, 1.1 equiv.), $Pd(OAc)_2$ (2.8 mg, 0.0125 mmol, 0.025 equiv.), urea **4e** (5.9 mg, 0.025 mmol, 0.05 equiv.), and 90:10 PhMe/PhOMe (2.00 mL, 0.25M) were used. Crude material was purified via column chromatography on 45 g of SiO_2 using 9:1

hexanes/diethyl ether to afford product as a pale-yellow oil. Isolated as an inseparable mixture of diastereomers. Yields are reported as a mixture of isomers (85:15 r.r.).

*Reaction time: 48 hours.

Run Number: (isolated yield in mg, isolated yield in mmol, isolated yield in percent)

Run 1: (61.0 mg, 0.250 mmol, 50%)
Run 2: (63.1 mg, 0.258 mmol, 52%)
Run 3: (67.0 mg, 0.274 mmol, 55%)
Average: 52% yield

^{1}H NMR (400 MHz, $CDCl_3$) δ 7.19 – 7.08 (m, 2H), 6.89 – 6.76 (m, 2H), 5.26 – 5.17 (m, 2H), 4.97 (s, 1H), 3.37 (dd, *J* = 15.7, 9.7 Hz, 1H), 3.07 (dd, *J* = 15.7, 8.4 Hz, 1H), 2.81 – 2.71 (m, 1H), 2.38 – 2.14 (m, 2H), 1.84 – 6.76 (m, 2H), 1.31 (s, 3H), 1.27 (s, 3H).

^{13}C NMR (101 MHz, $CDCl_3$) δ 159.7, 147.5, 128.2, 126.5, 124.9, 120.5, 111.1, 109.3, 85.2, 64.0, 58.5, 35.3, 27.9, 27.4, 25.0, 18.8.

HRMS (ESI) *m/z* calculated for $C_{16}H_{20}O_2$ $[M+H]^+$: 245.1540, found 245.1534.

6-(2,3-dihydrobenzofuran-2-yl)-2-methylhept-6-en-2-ol (3al)

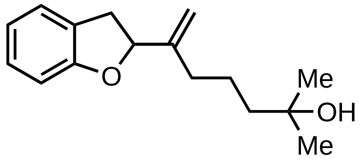

Prepared according to general procedure B. 2-bromophenol **1a** (58.0 µL, 0.500 mmol, 1.0 equiv.), myrcenol **2l** (154 mg, 1.00 mmol, 2.0 equiv.), NaO^{t}Bu (52.9 mg, 0.550 mmol, 1.1 equiv.), $Pd(OAc)_2$ (2.8 mg, 0.0125 mmol, 0.025 equiv.), urea **4e** (5.9 mg, 0.025 mmol, 0.05 equiv.), and 90:10 PhMe/PhOMe (2.00 mL, 0.25M) were used. Crude material was purified via column chromatography on 45 g of SiO_2 using 9:1 hexanes/EtOAc to afford product as a tan oil. Yields are reported as a mixture of isomers (85:15 r.r.).

Run Number: (isolated yield in mg, isolated yield in mmol, isolated yield in percent)

Run 1: (64.0 mg, 0.260 mmol, 52%)
Run 2: (64.8 mg, 0.263 mmol, 53%)
Run 3: (67.7 mg, 0.275 mmol, 55%)
Average: 53% yield

^{1}H NMR (400 MHz, $CDCl_3$) δ 7.19 – 7.07 (m, 2H), 6.88 – 6.78 (m, 2H), 5.21 – 5.16 (m, 2H), 4.95 (d, *J* = 1.6 Hz, 1H), 3.35 (dd, *J* = 15.5, 9.6 Hz, 1H), 3.05 (dd, *J* = 15.6, 8.4 Hz, 1H), 2.11 (ddd, *J* = 17.1, 8.8, 8.8 Hz, 2H), 1.68 – 1.57 (m, 2H), 1.51 – 1.45 (m, 3H), 1.22 (s, 6H).

^{13}C NMR (101 MHz, $CDCl_3$) δ 159.7, 148.1, 128.1, 126.6, 124.9, 120.5, 110.6, 109.3, 85.3, 71.0, 43.6, 35.3, 31.5, 29.4, 29.3, 22.4.

HRMS (ESI) *m/z* calculated for $C_{16}H_{22}O_2$ $[M+H]^+$: 247.1698, found 247.1691.

(*E*)-2-(hex-1-en-1-yl)-2-methyl-2,3-dihydrobenzofuran (3am)

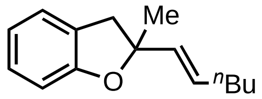

Prepared according to general procedure B. 2-bromophenol **1a** (58.0 µL, 0.500 mmol, 1.0 equiv.), (*E*)-2-methylocta-1,3-diene **2m** (124 mg, 1.00 mmol, 2.0 equiv.), NaO*t*Bu (52.9 mg, 0.550 mmol, 1.1 equiv.), $Pd(OAc)_2$ (2.8 mg, 0.0125 mmol, 0.025 equiv.), urea **4e** (5.9 mg, 0.025 mmol, 0.05 equiv.), and 90:10 PhMe/PhOMe (2.00 mL, 0.25M) were used. Crude material was purified via column chromatography on 45 g of SiO_2 using 8:2 hexanes/PhMe to afford product as a colorless oil. (>95:5 *E/Z*)

<u>Run Number:</u> (isolated yield in mg, isolated yield in mmol, isolated yield in percent)

<u>Run 1:</u> (61.5 mg, 0.284 mmol, 57%)
<u>Run 2:</u> (62.6 mg, 0.289 mmol, 58%)
<u>Run 3:</u> (60.4 mg, 0.280 mmol, 56%)
Average: 57% yield

^{1}H NMR (400 MHz, $CDCl_3$) δ 7.13 (dd, *J* = 10.2, 7.4 Hz, 2H), 6.90 – 6.76 (m, 2H), 5.84 – 5.67 (m, 2H), 3.19 (d, *J* = 15.4 Hz, 1H), 3.04 (d, *J* = 15.4 Hz, 1H), 2.06 (td, *J* = 6.7 Hz, 2H), 1.55 (s, 3H), 1.42 – 1.31 (m, 4H), 0.91 (t, *J* = 6.9 Hz, 3H).

^{13}C NMR (126 MHz, $CDCl_3$) δ 158.9, 133.7, 129.6, 128.1, 126.9, 125.1, 120.2, 109.6, 87.6, 42.6, 32.0, 31.4, 26.5, 22.3, 14.1.

HRMS (ESI) *m/z* calculated for $C_{15}H_{20}O$ $[M+H]^+$: 217.1592, found 217.1585.

(*E*)-2-(1-phenylprop-1-en-2-yl)-2,3-dihydrobenzofuran (3an)

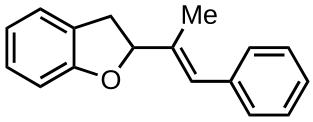

Prepared according to general procedure B. 2-bromophenol **1a** (58.0 µL, 0.500 mmol, 1.0 equiv.), (*E*)-(2-methylbuta-1,3-diene-1-yl)benzene **2n** (108 mg, 0.750 mmol, 1.5 equiv.), NaO*t*Bu (52.9 mg, 0.550 mmol, 1.1 equiv.), $Pd(OAc)_2$ (2.8 mg, 0.0125 mmol, 0.025 equiv.), urea **4e** (5.9 mg, 0.025 mmol, 0.05 equiv.), and 90:10 PhMe/PhOMe (2.00 mL, 0.25M) were used. Crude material was purified via column chromatography on 45 g of SiO_2 using 97:3 hexanes/ethyl acetate to afford product as a yellow oil. (>95:5 *E/Z*)

<u>Run Number:</u> (isolated yield in mg, isolated yield in mmol, isolated yield in percent)

<u>Run 1:</u> (72.9 mg, 0.308 mmol, 62%)
<u>Run 2:</u> (74.7 mg, 0.316 mmol, 63%)
<u>Run 3:</u> (70.8 mg, 0.300 mmol, 60%)
Average: 62% yield

^{1}H NMR (400 MHz, $CDCl_3$) δ 7.44 – 7.31 (m, 4H), 7.33 – 7.15 (m, 3H), 6.96 – 6.81 (m, 2H), 6.70 (s, 1H), 5.35 (t, *J* = 9.0 Hz, 1H), 3.45 (dd, *J* = 15.8, 9.5 Hz, 1H), 3.21 (dd, *J* = 15.7, 8.4 Hz, 1H), 1.96 (s, 3H).

^{13}C NMR (101 MHz, $CDCl_3$) δ 160.0, 137.2, 136.8, 129.1, 128.3 (2C), 128.2 (2C), 126.9, 126.8 (2C), 124.9, 120.5, 109.4, 87.9, 34.9, 13.0.

HRMS (ESI) *m/z* calculated for $C_{17}H_{16}O$ $[M+H]^+$: 237.1279, found 237.1272.

Poorly Reactive Substrates

Table S2. Unsuccessful substrates

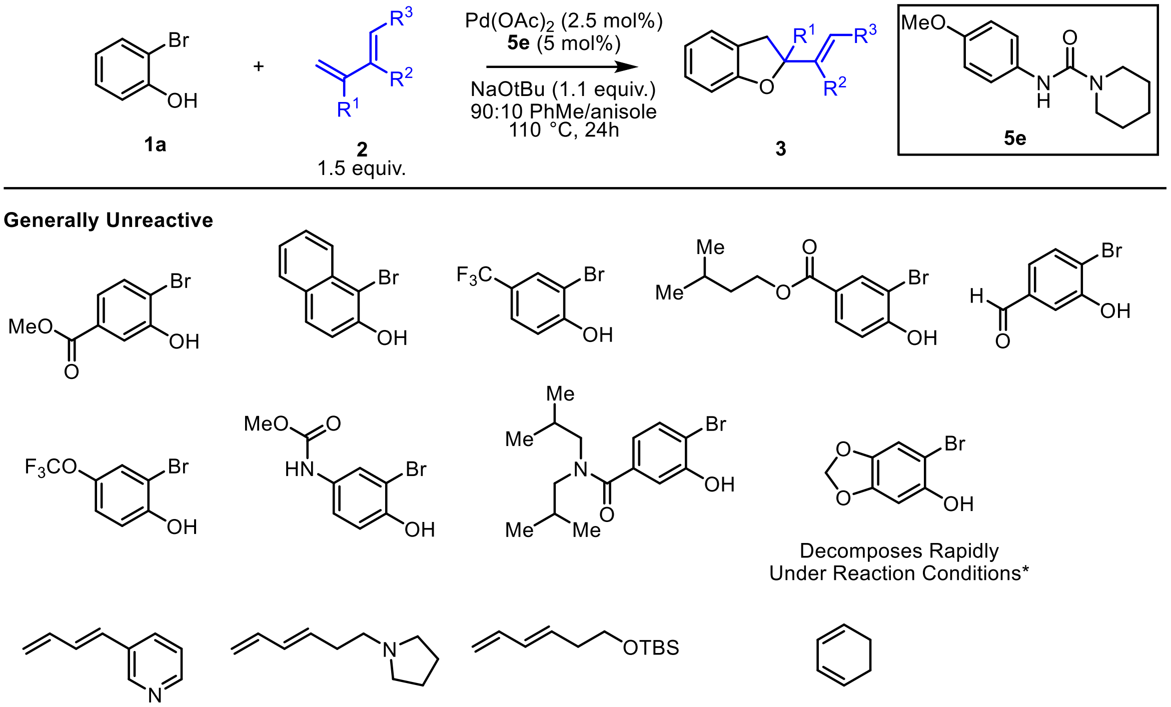

Computational studies

Density functional theory (DFT) calculations were performed with Gaussian 16 package using University of Rochester's BlueHive computer cluster.[14] Geometry optimizations and frequency calculations were performed at the SMD(acetone)-MN15-def2TZVPP computational level unless otherwise indicated.[15] This computational level was previously benchmarked on a prior solution-state study of urea binding of urea to $Pd(en)(H_2O)^{2+}$ cation. When the substrates showed conformational freedom, conformational analysis was performed and only the most stable conformer of each stationary point was considered and reported, unless otherwise noted. The Percent Buried Volume ($\%V_{bur}$) calculations were performed using the SambVca 2.1 web application.[16]

Computational level benchmarking and Urea vs Ureate binding modes

Our previous reports compare the relative energies of different urea-Pd or ureate-Pd binding modes using SMD(acetone)-MN15- def2TZVPP level of theory. This was found to be a sufficient functional, basis set, and solvation condition for our own studies. We include the corresponding $PdCl_2$ ureate *N*-bound complexes are the basic scaffolds for our binding studies as well.[7]

Comparison of urea-Pd binding modes

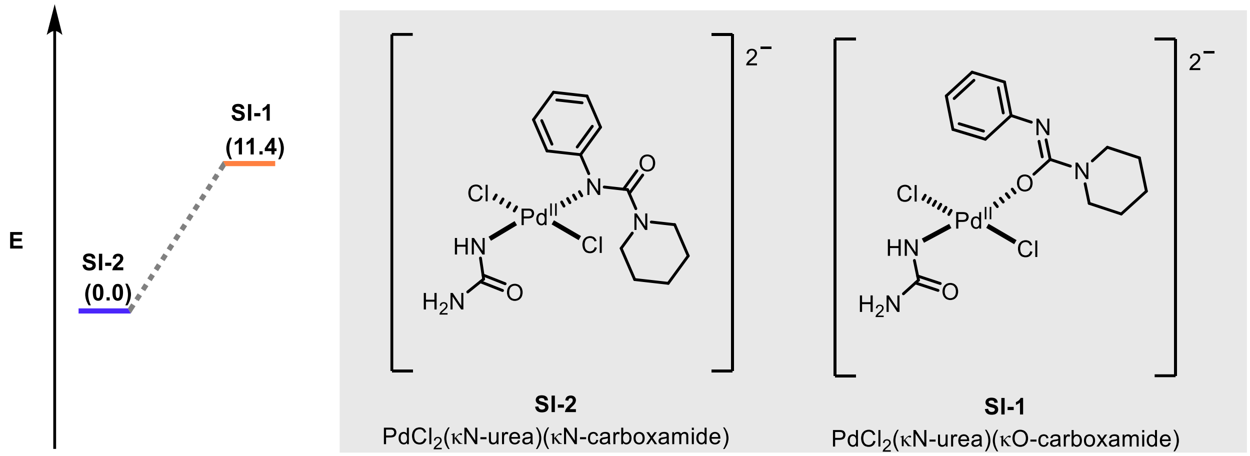

Figure S1. Comparison of carboxamide-Pd binding modes (values in parentheses are Gibbs free energies in kcal mol-1). Ground state energy calculations were carried out at the SMD(acetone)-MN15- def2TZVPP level of theory.

SI-1 ($PdCl_2$(***κN***-urea)(***κO***-carboxamide) (SMD(acetone)-MN15-def2TZVPP)

		Hartree
Sum of electronic and zero-point Energies	=	-1923.052699
Sum of electronic and thermal Energies	=	-1923.029459
Sum of electronic and thermal Enthalpies	=	-1923.028515
Sum of electronic and thermal Free Energies	=	-1923.108328
Lowest energy vibration	=	23.60 cm^{-1}

-2 1

Pd	1.86320400	-0.11385300	0.28884700	N	4.42402000	-0.69980700	-1.68160000
Cl	2.47355900	-0.07001000	2.51925600	H	3.88050900	1.44939400	0.59557900
Cl	1.19920600	-0.18611500	-1.95526400	H	3.46293700	-0.82935400	-1.98190000
N	3.58198100	0.79737200	-0.12158900	H	5.12151400	-0.79157600	-2.40690300
C	-0.98178400	-0.45724100	0.64132300	O	0.13237800	-1.08110000	0.73769100
C	4.64359400	0.36986800	-0.82731000	N	-1.08031400	0.84295300	0.82660000
O	5.78192900	0.86647600	-0.74089900	C	-1.87567500	-2.65156900	0.00571100

C	-3.36309900	-1.02768700	1.01742400	H	-4.43475300	-3.52817200	0.59356300
C	-2.98329600	-3.11179500	-0.93175900	N	-2.08163800	-1.25965900	0.36910100
H	-1.87432600	-3.28411600	0.90723600	C	-2.07219500	1.62102200	0.26649400
H	-0.90114100	-2.74575600	-0.46610100	C	-2.57513400	2.72087800	0.98624700
C	-4.50984900	-1.43933200	0.10764300	C	-2.56248400	1.43657100	-1.04236100
H	-3.40891200	-1.63252400	1.93729700	C	-3.52395000	3.57407000	0.44058000
H	-3.44786700	0.01619600	1.30724000	H	-2.19908400	2.88833100	1.98946200
C	-4.35190100	-2.89826400	-0.30024400	C	-3.51005400	2.29427600	-1.58282600
H	-2.82890400	-4.16305100	-1.18382400	H	-2.18185400	0.61014600	-1.63163300
H	-2.91557500	-2.53530600	-1.86060200	C	-4.00715600	3.36939600	-0.84916100
H	-5.45893500	-1.27745700	0.62257500	H	-3.88905200	4.40802200	1.02907900
H	-4.50014900	-0.80288100	-0.78355700	H	-3.86037600	2.12390100	-2.59469100
H	-5.14771600	-3.20039100	-0.98384500	H	-4.74565000	4.03575900	-1.2763030

SI-2 ($PdCl_2$(***κN***-urea)(***κN***-carboxamide) (SMD(acetone)-MN15-def2TZVPP)

		**Hartree**
Sum of electronic and zero-point Energies	=	-1923.072261
Sum of electronic and thermal Energies	=	-1923.049108
Sum of electronic and thermal Enthalpies	=	-1923.048164
Sum of electronic and thermal Free Energies	=	-1923.126514
Lowest energy vibration	=	29.76 cm^{-1}

-2 1

Pd	-0.10582800	0.83199000	-0.04596100	C	0.72768400	-2.01510600	-0.28649600
Cl	-0.88331100	1.01995800	2.16921900	C	-0.80563500	3.77536900	-0.25593100
Cl	0.63633000	0.65660000	-2.23799100	O	-0.63419800	4.92087300	-0.71410900
N	0.14029900	2.82286400	-0.20889400	O	0.63946900	-3.02363700	-1.00145200
N	-0.32849000	-1.21499600	0.06665500	C	2.17517100	-0.90417000	1.42472400

C	3.16531600	-2.19748600	-0.36869300
C	3.18493100	0.21948600	1.24666800
H	2.57735300	-1.61955200	2.16081400
H	1.22415900	-0.53395300	1.79943300
C	4.21542900	-1.11721600	-0.60013200
H	3.56596100	-2.94683400	0.33222600
H	2.91026300	-2.71048400	-1.29274200
C	4.48584800	-0.34076000	0.68329400
H	3.35652800	0.71662500	2.20428600
H	2.76484000	0.96144100	0.55720000
H	5.13320100	-1.57356800	-0.97786400
H	3.84497200	-0.43183600	-1.36965800
H	5.21075900	0.45655200	0.50703300
H	4.92634400	-1.02043900	1.42324800
N	-2.05571800	3.41478900	0.23345000
N	1.96234700	-1.60438100	0.17326700
H	0.94635500	3.10666900	-0.75589100
H	-2.05461100	2.65166100	0.90329800
H	-2.63663100	4.20350000	0.48257400
C	-1.61591100	-1.72891300	-0.01865500
C	-2.69699900	-0.89257000	-0.34772100
C	-1.92281100	-3.07020700	0.28808800
C	-4.00328000	-1.36718300	-0.37460300
H	-2.50023600	0.14903300	-0.58118100
C	-3.22732100	-3.53698000	0.25787600
H	-1.11949900	-3.74271200	0.55514200
C	-4.28656800	-2.69432000	-0.07589000
H	-4.80510200	-0.68605100	-0.63531300
H	-3.42039300	-4.57450900	0.50621900
H	-5.30384300	-3.06405500	-0.09753800

V_{bur} value

The Percent Buried Volume (%V_{bur}) calculations were performed using the SambVca 2.1 web application. [16] The recommended parameters were used (i.e. Bondii radii scaled by 1.17, sphere radius = 3.5, mesh spacing for numerical integration = 0.10, H atoms not included). %Vbur was calculated for [*N*'-arlycarboxamide complex **SI-3**] and [*N*'-arlycarboxamide complex **SI-4**]. [16]

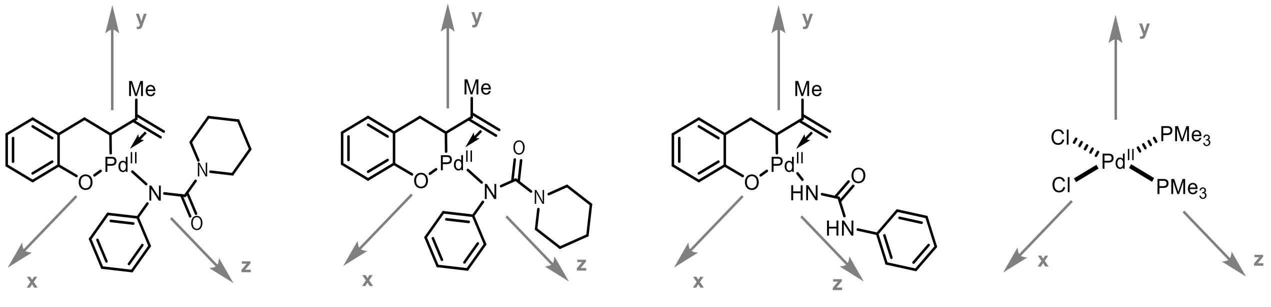

**SI-3**	**SI-4**	**SI-5**	**SI-6**
**%V_{bur} = 32.5%**	**%$_{Vbur}$ = 25.4%**	**%Vbur = 16.9%**	**%V_{bur} = 23.5%**

SI-6 calculation performed previously by Jakub Vaith (2022). [7]

[*N*'-arlycarboxamide complex SI-3]

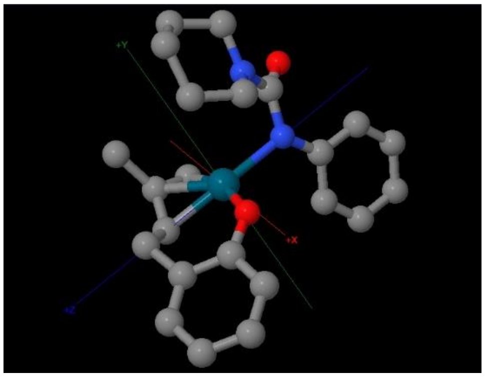

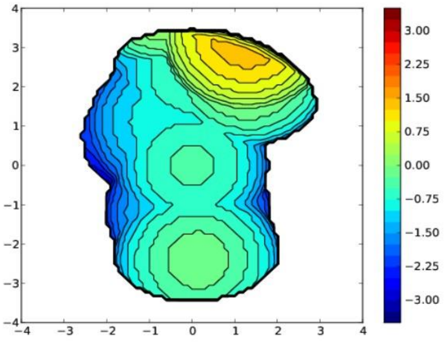

[*N*’-arylcarboxamide complex SI-4]

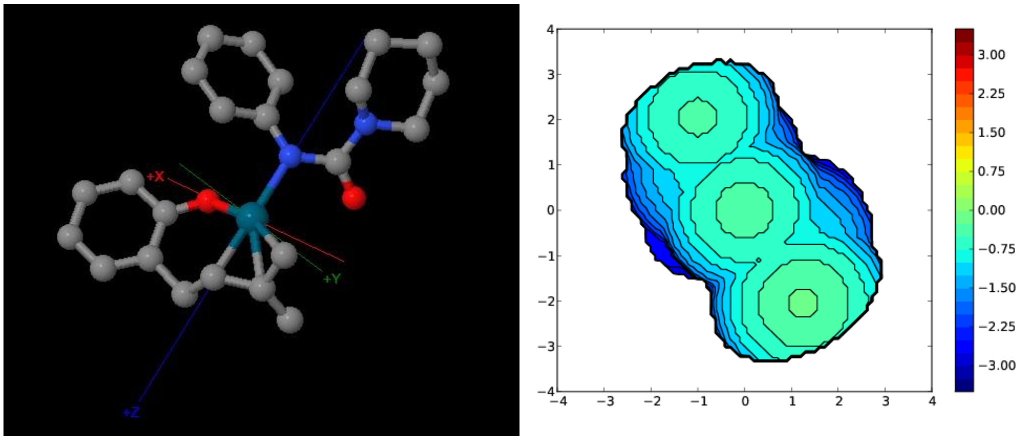

[*N*’-phenylurea complex SI-5]

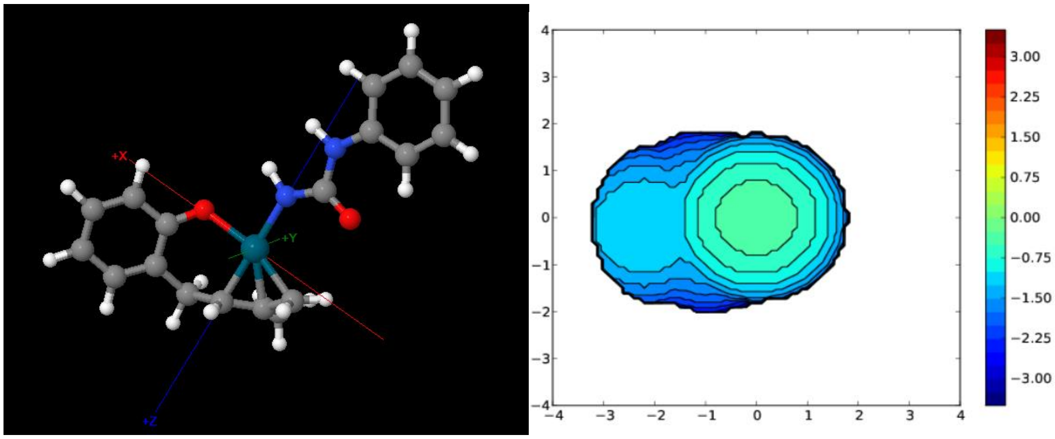

SI-6[17]

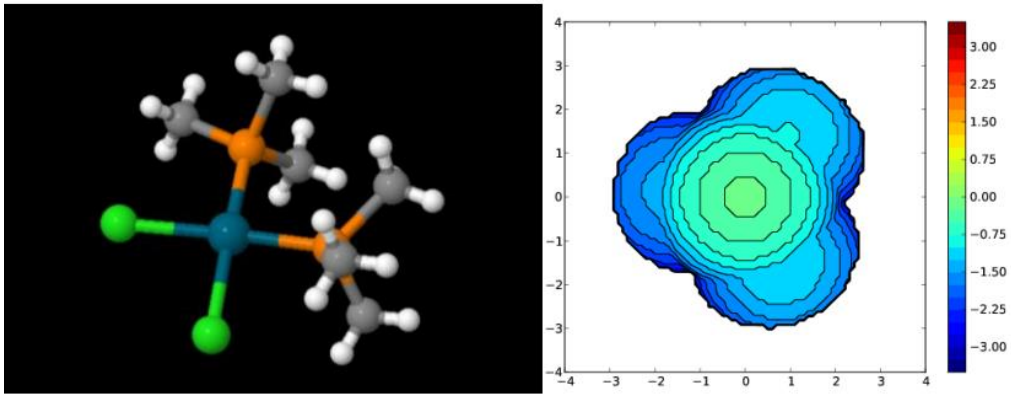

References Cited

(1) Wu, Y.-Q.; Lu, H.-J.; Zhao, W.-T.; Zhao, H.-Y.; Lin, Z.-Y.; Zhang, D.-F.; Huang, H.-H., A convenient and efficient H2SO4-promoted regioselective monobromination of phenol derivatives using N-bromosuccinimide. *Synth. Commun.* **2020,** *50*, 813-822.
(2) D. Cirillo, S. Sarowar, P. Øyvind Enger, H.-R. Bjørsvik, *Chem. Med. Chem.* **2021**, *16*, 2650.
(3) Hasegawa, T.; Yamamoto, H., Development of New Chiral Auxiliary Derived from (S)-(-)-Phenylethylamine for a Synthesis of Enantiopure (R)-2-Propyloctanoic Acid. *Synthesis* **2003,** *2003*, 1181-1186.
(4) Bum-Erdene, K.; Liu, D.; Xu, D.; Ghozayel, M. K.; Meroueh, S. O., Design and Synthesis of Fragment Derivatives with a Unique Inhibition Mechanism of the uPAR·uPA Interaction. *ACS Med. Chem. Lett.* **2021,** *12*, 60-66.
(5) Pacherille, A.; Tuga, B.; Hallooman, D.; Dos Reis, I.; Vermette, M.; Issack, B. B.; Rhyman, L.; Ramasami, P.; Sunasee, R., BiCl3-Facilitated removal of methoxymethyl-ether/ester derivatives and DFT study of –O–C–O– bond cleavage. *New J. Chem.* **2021,** *45*, 7109-7116.
(6) de Oliveira, C. S.; Lira, B. F.; dos Santos Falcão-Silva, V.; Siqueira, J. P., Jr.; Barbosa-Filho, J. M.; de Athayde-Filho, P. F., Synthesis, molecular properties prediction, and anti-staphylococcal activity of N-acylhydrazones and new 1,3,4-oxadiazole derivatives. *Molecules* **2012,** *17*, 5095-107.
(7) Vaith, J.; Rodina, D.; Spaulding, G. C.; Paradine, S. M., Pd-Catalyzed Heteroannulation Using N-Arylureas as a Sterically Undemanding Ligand Platform. *J. Am. Chem. Soc.* **2022**, 144, 6667-6673.
(8) Sun, Y.; Zhang, G., Palladium-Catalyzed Formal [4+2] Cycloaddition of Benzoic and Acrylic Acids with 1,3-Dienes via C—H Bond Activation: Efficient Access to 3,4-Dihydroisocoumarin and 5,6-Dihydrocoumalins. *Chinese Journal of Chemistry* **2018,** *36*, 708-711.
(9) Khan, I.; Chidipudi, S. R.; Lam, H. W., Synthesis of spiroindanes by palladium-catalyzed oxidative annulation of non- or weakly activated 1,3-dienes involving C–H functionalization. *Chemical Communications* **2015,** *51*, 2613-2616.
(10) Adamson, N. J.; Hull, E.; Malcolmson, S. J., Enantioselective Intermolecular Addition of Aliphatic Amines to Acyclic Dienes with a Pd–PHOX Catalyst. *J. Ame. Chem. Soc.* **2017,** *139*, 7180-7183.
(11) Xu, Y.; Qi, X.; Zheng, P.; Berti, C. C.; Liu, P.; Dong, G., Deacylative transformations of ketones via aromatization-promoted C–C bond activation. *Nature* **2019,** *567*, 373-378.
(12) Ling, F.-W.; Luo, M.-C.; Chen, M.-K.; Zeng, J.; Li, S.-Q.; Yin, H.-B.; Wu, J.-R.; Xu, Y.-X.; Huang, G., Terminally and randomly functionalized polyisoprene lead to distinct aggregation behaviors of polar groups. *Polymer* **2019,** *178*, 121629.
(13) Gustavo M. Borrajo-Calleja, Vincent Bizet, Céline Besnard, and Clément Mazet., Mechanistic Investigation of the Pd-Catalyzed Intermolecular Carboetherification and Carboamination of 2,3-Dihydrofuran: Similarities, Differences, and Evidence for Unusual Reaction Intermediates. *Organometallics* **2017,** *36*, 3553-3563.
(14) Gaussian 16, Revision A.03, Frisch, M. J.; Trucks, G. W.; Schlegel, H. B.; Scuseria, G. E.; Robb, M. A.; Cheeseman, J. R.; Scalmani, G.; Barone, V.; Petersson, G. A.;

Nakatsuji, H.; Li, X.; Caricato, M.; Marenich, A. V.; Bloino, J.; Janesko, B. G.; Gomperts, R.; Mennucci, B.; Hratchian, H. P.; Ortiz, J. V.; Izmaylov, A. F.; Sonnenberg, J. L.; Williams-Young, D.; Ding, F.; Lipparini, F.; Egidi, F.; Goings, J.; Peng, B.; Petrone, A.; Hender-son, T.; Ranasinghe, D.; Zakrzewski, V. G.; Gao, J.; Rega, N.; Zheng, G.; Liang, W.; Hada, M.; Ehara, M.; Toyota, K.; Fukuda, R.; Haseg-awa, J.; Ishida, M.; Nakajima, T.; Honda, Y.; Kitao, O.; Nakai, H.; Vreven, T.; Throssell, K.; Montgomery, J. A., Jr.; Peralta, J. E.; Ogliaro, F.; Bearpark, M. J.; Heyd, J. J.; Brothers, E. N.; Kudin, K. N.; Staroverov, V. N.; Keith, T. A.; Kobayashi, R.; Normand, J.; Raghavachari, K.; Rendell, A. P.; Burant, J. C.; Iyengar, S. S.; To-masi, J.; Cossi, M.; Millam, J. M.; Klene, M.; Adamo, C.; Cammi, R.; Ochterski, J. W.; Martin, R. L.; Morokuma, K.; Farkas, O.; For-esman, J. B.; Fox, D. J. Gaussian, Inc., Wallingford CT, 2016.

(15) Yu, H. S.; He, X.; Li, S. L.; Truhlar, D. G., MN15: A Kohn–Sham global-hybrid exchange–correlation density functional with broad accuracy for multi-reference and single-reference systems and noncovalent interactions. *Chem. Sci.* **2016,** *7*, 5032-5051.

(16) Falivene, L.; Cao, Z.; Petta, A.; Serra, L.; Poater, A.; Oliva, R.; Scarano, V.; Cavallo, L., Towards the online computer-aided design of catalytic pockets. *Nat. Chem.* **2019,** *11*, 872-879.

(17) Schultz, G.; Subbotina, N. Yu.; Jensen, C. M.; Golen, J. A.; Hargittai, I. Gas phase and crystal molecular structures of cis-PdCl2[P(CH3)3]2. Inorg. Chim. Acta 1992, 191, 85−90.

NMR Spectra of New Compounds

2-bromo-4-iodo-1-(methoxymethoxy)benzene

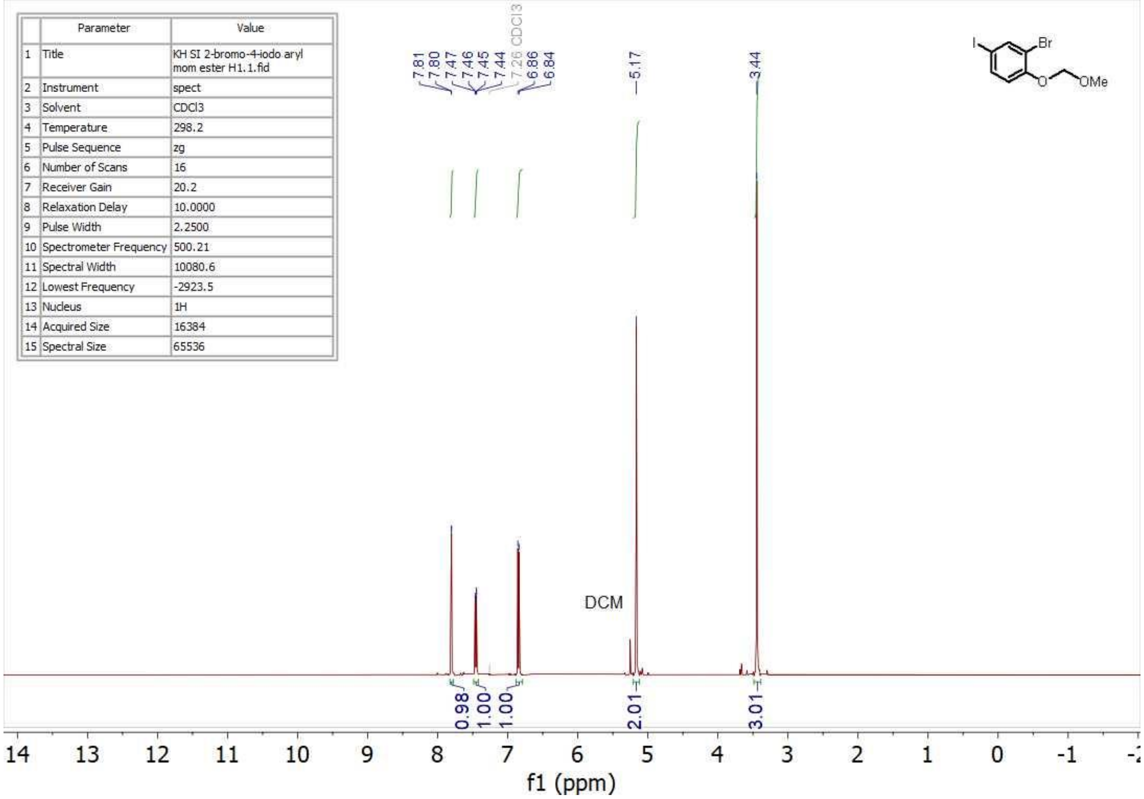

	Parameter	Value
1	Title	KH SI 2-bromo-4-iodo aryl mom ester H1.1.fid
2	Instrument	spect
3	Solvent	CDCl3
4	Temperature	298.2
5	Pulse Sequence	zg
6	Number of Scans	16
7	Receiver Gain	20.2
8	Relaxation Delay	10.0000
9	Pulse Width	2.2500
10	Spectrometer Frequency	500.21
11	Spectral Width	10080.6
12	Lowest Frequency	-2923.5
13	Nucleus	1H
14	Acquired Size	16384
15	Spectral Size	65536

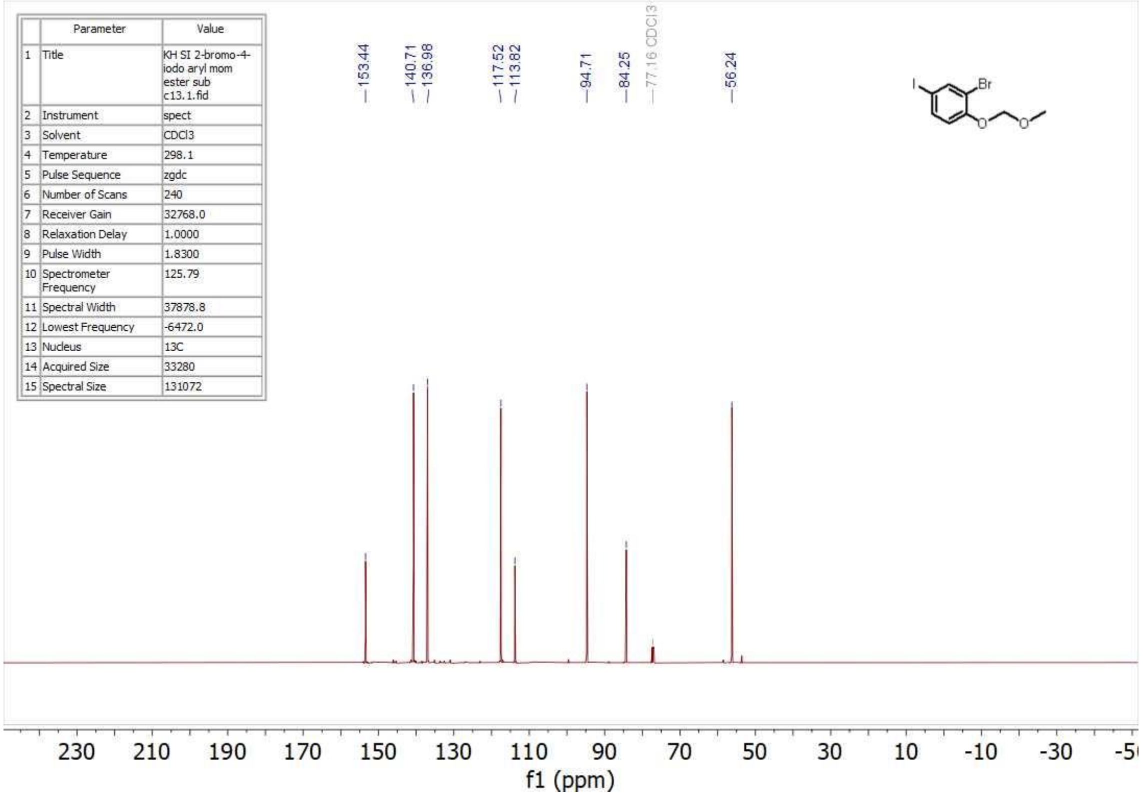

	Parameter	Value
1	Title	KH SI 2-bromo-4-iodo aryl mom ester sub c13.1.fid
2	Instrument	spect
3	Solvent	CDCl3
4	Temperature	298.1
5	Pulse Sequence	zgdc
6	Number of Scans	240
7	Receiver Gain	32768.0
8	Relaxation Delay	1.0000
9	Pulse Width	1.8300
10	Spectrometer Frequency	125.79
11	Spectral Width	37878.8
12	Lowest Frequency	-6472.0
13	Nucleus	13C
14	Acquired Size	33280
15	Spectral Size	131072

1-(3-bromo-4-(methoxymethoxy)phenyl)piperidine

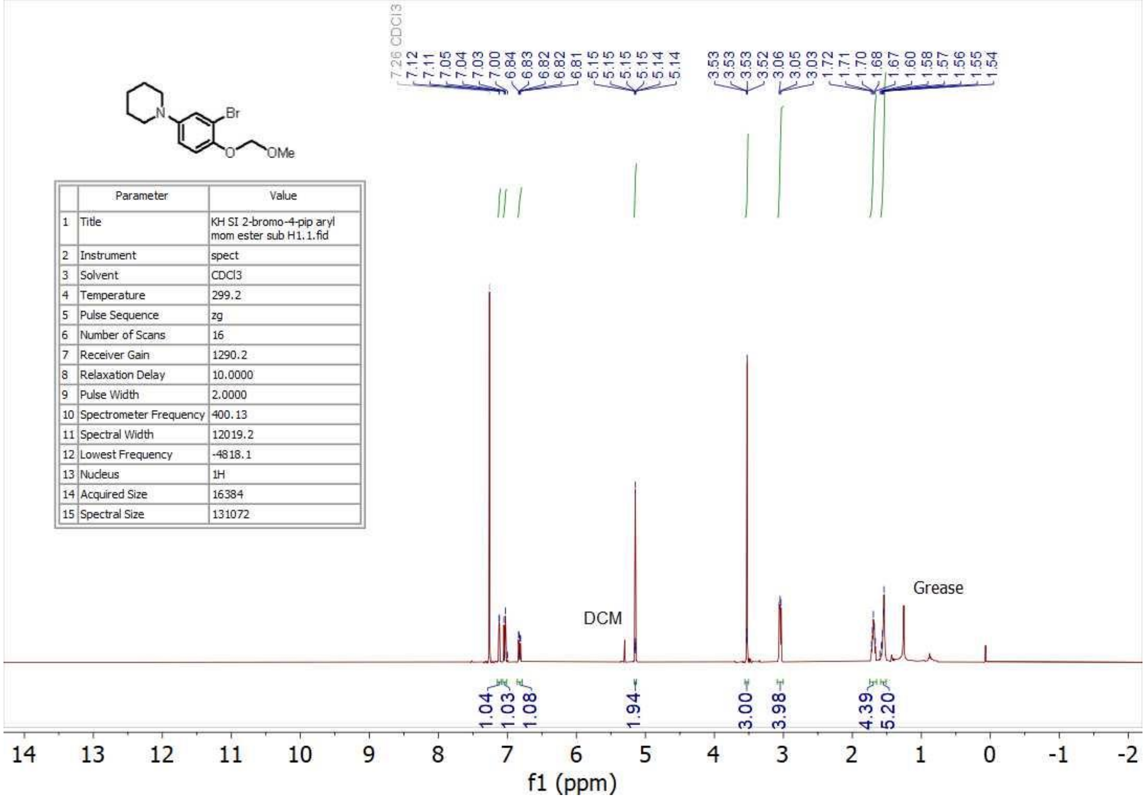

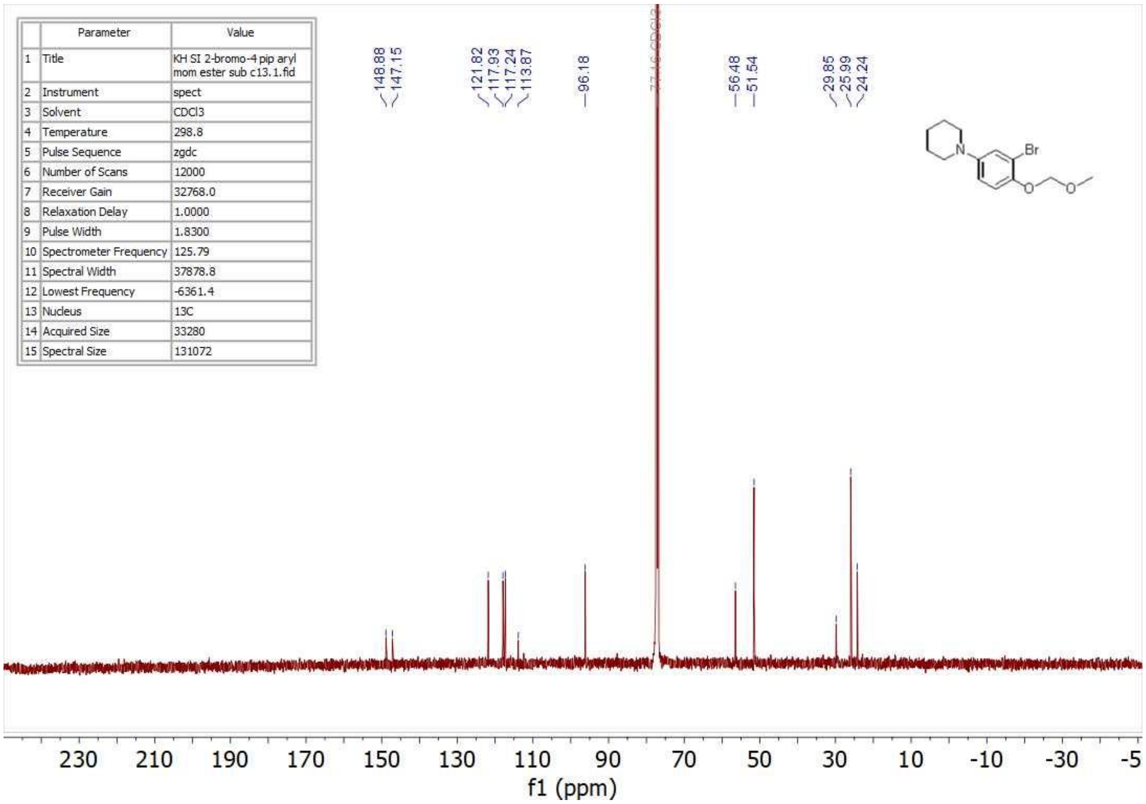

2-bromo-4-(piperidin-1-yl) phenol (1l)

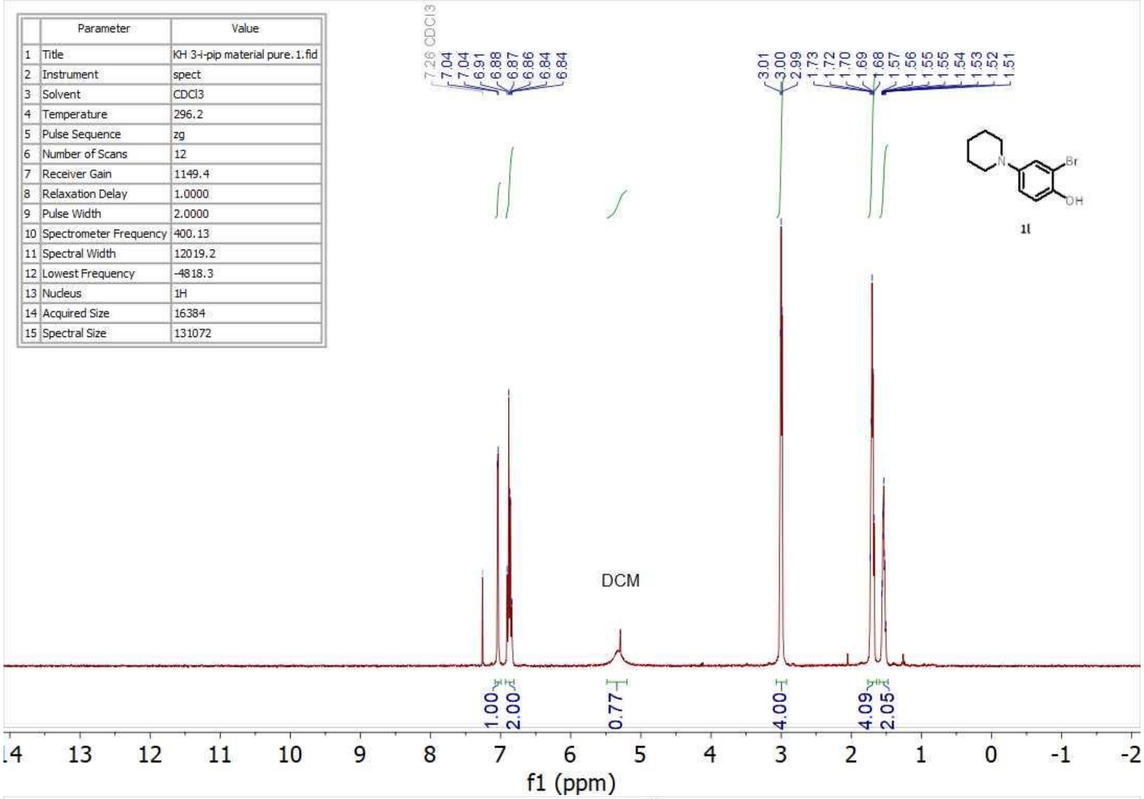

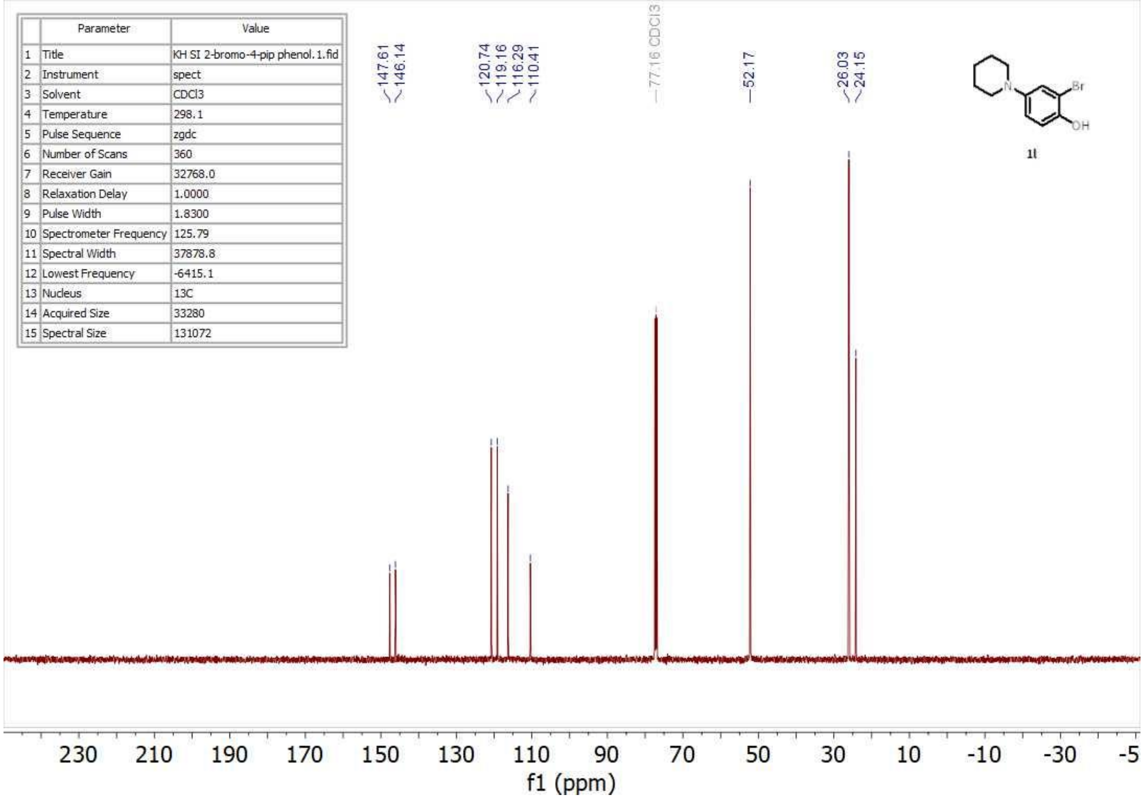

Isopentyl 4-bromo-3-hydroxybenzoate (1m)

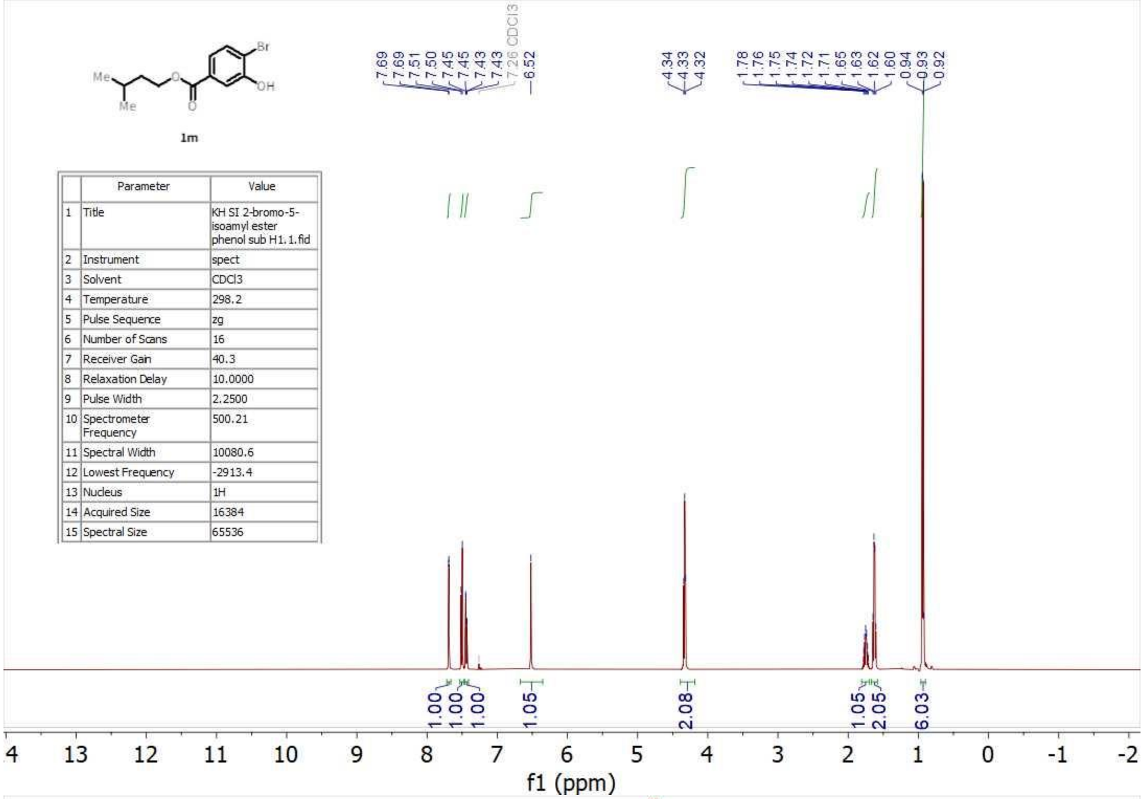

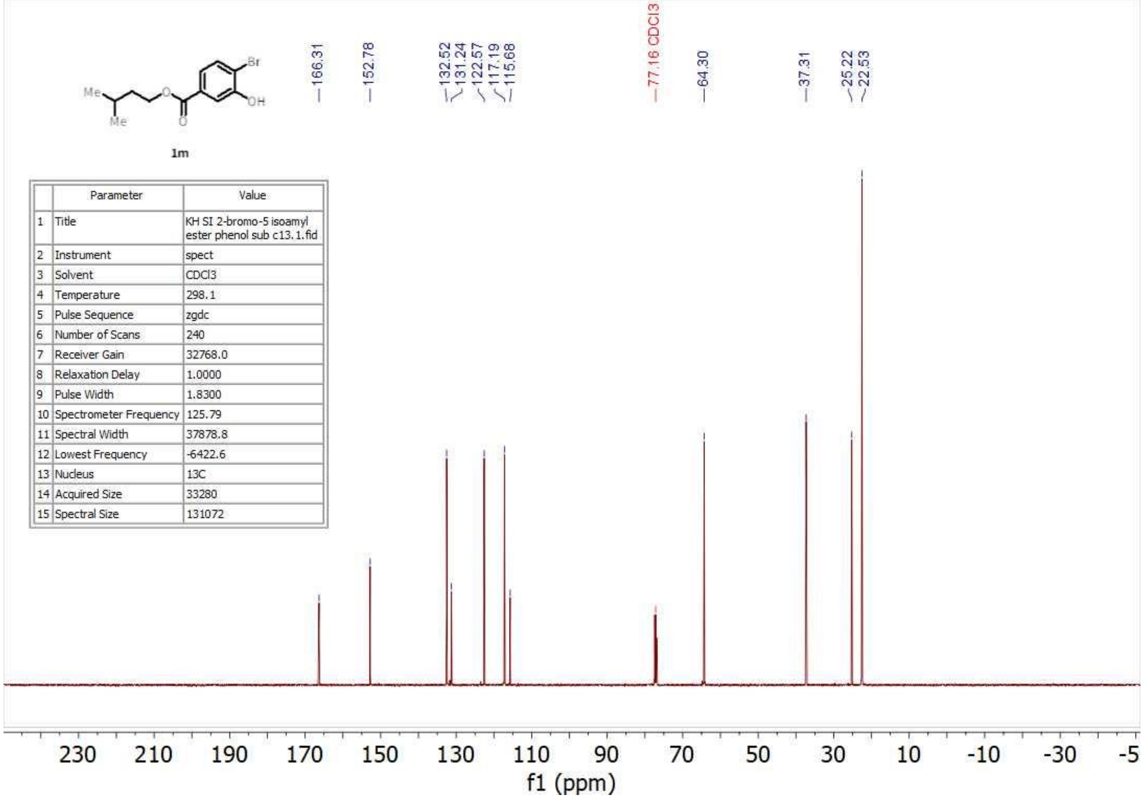

(*E*)-2-(buta-1,3-dien-1-yl)-5-methylfuran (2h)

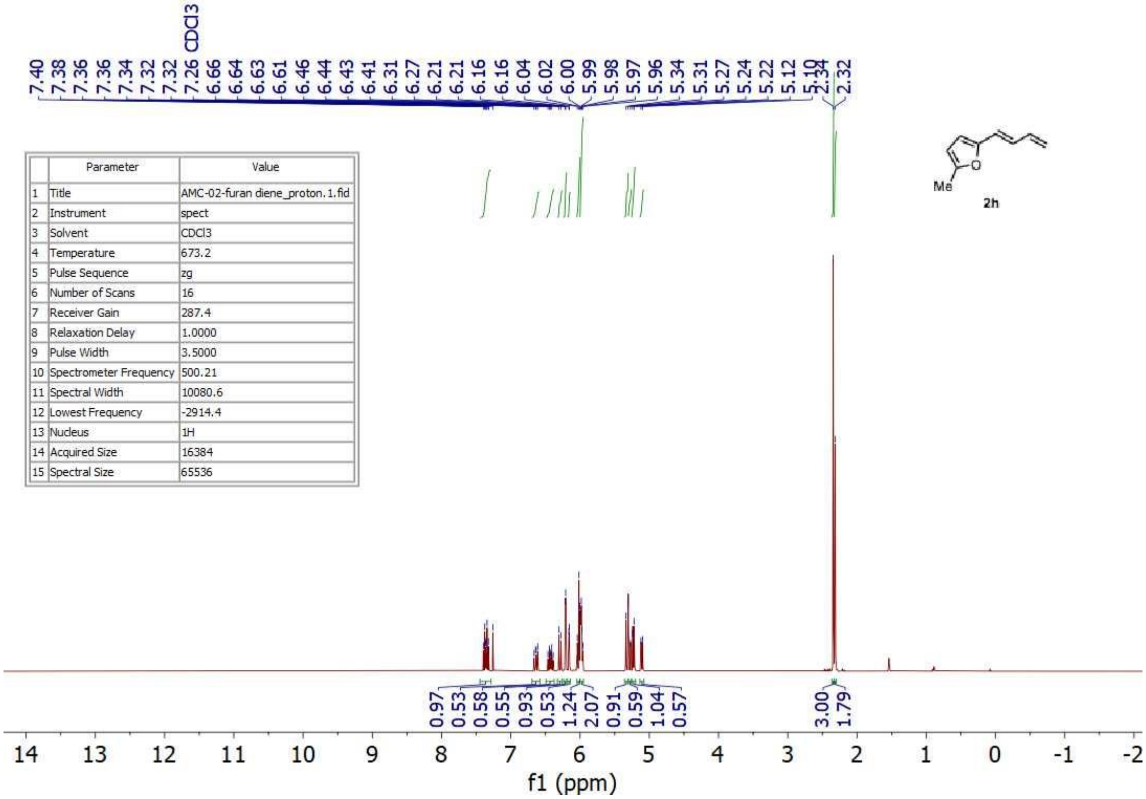

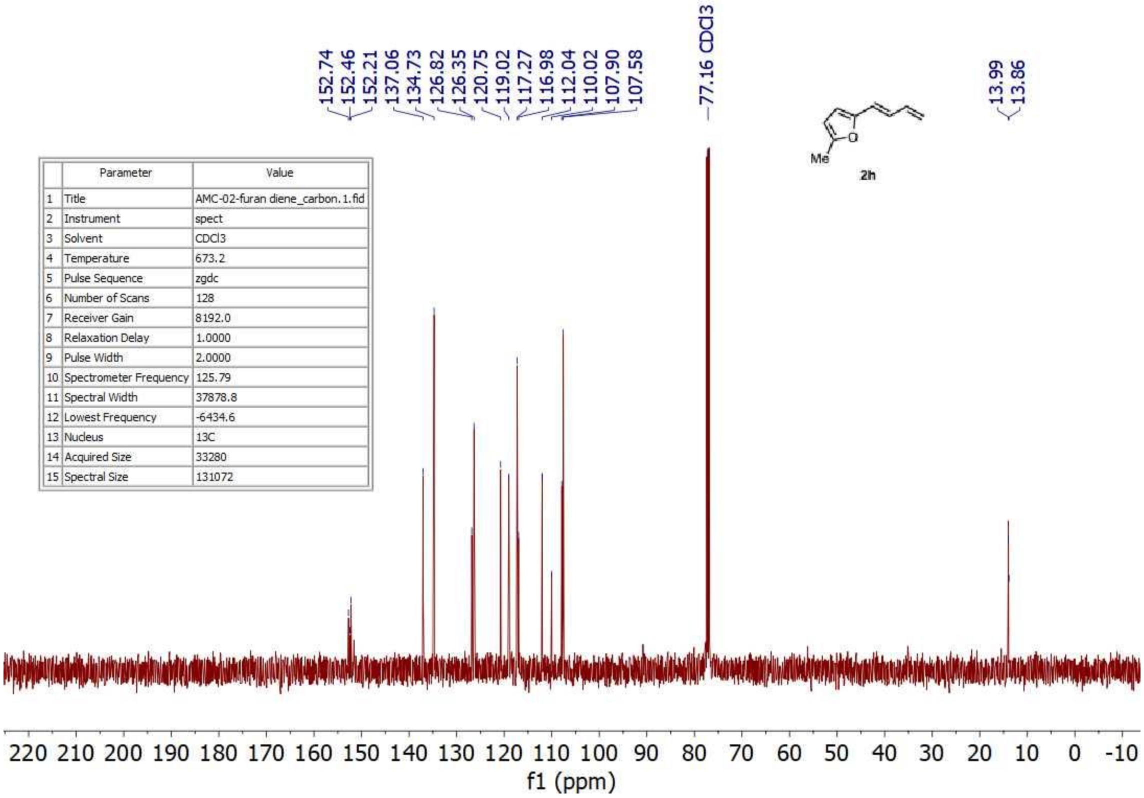

N-phenylpiperidine-1-carboxamide (4d)

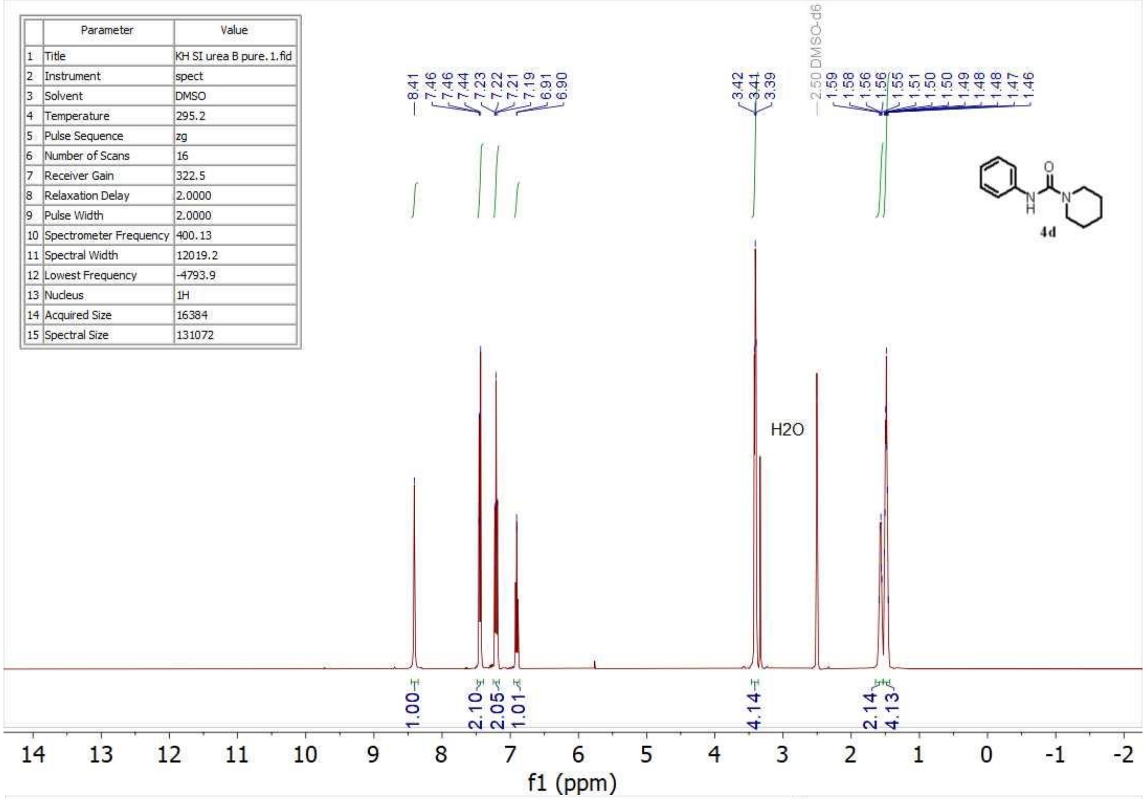

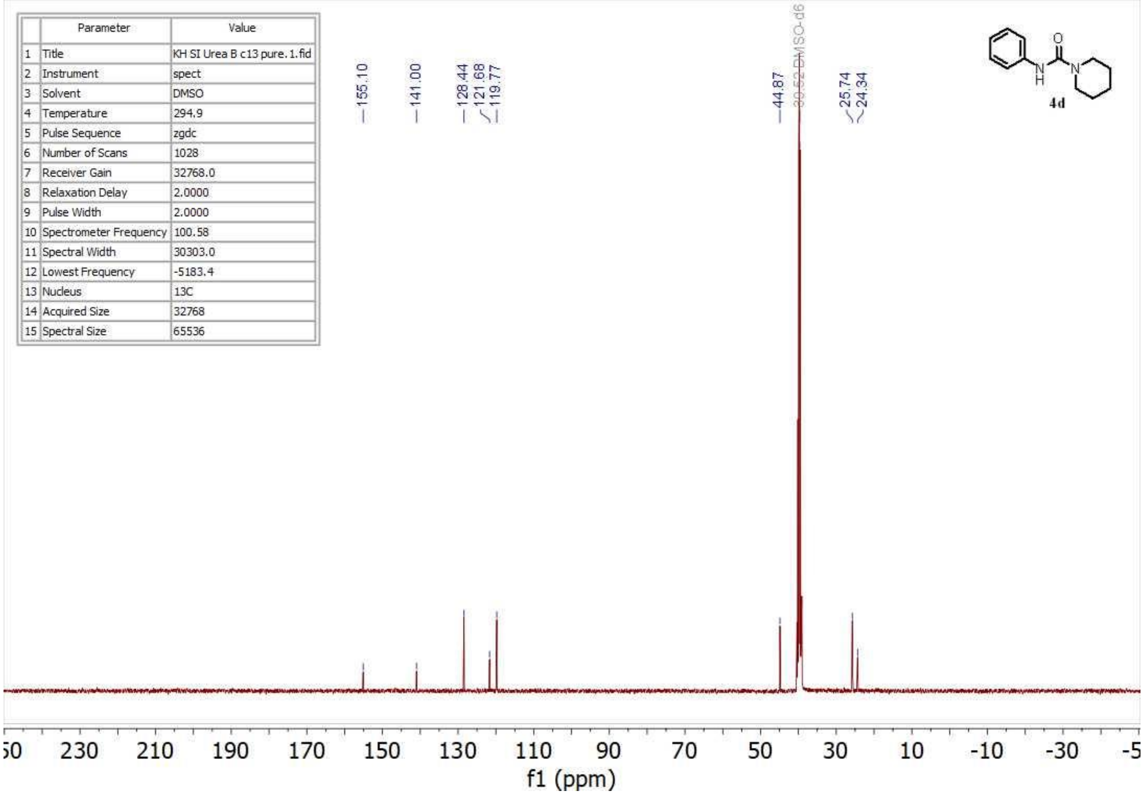

_N_-(4-methoxyphenyl)piperidine-1-carboxamide (4e)

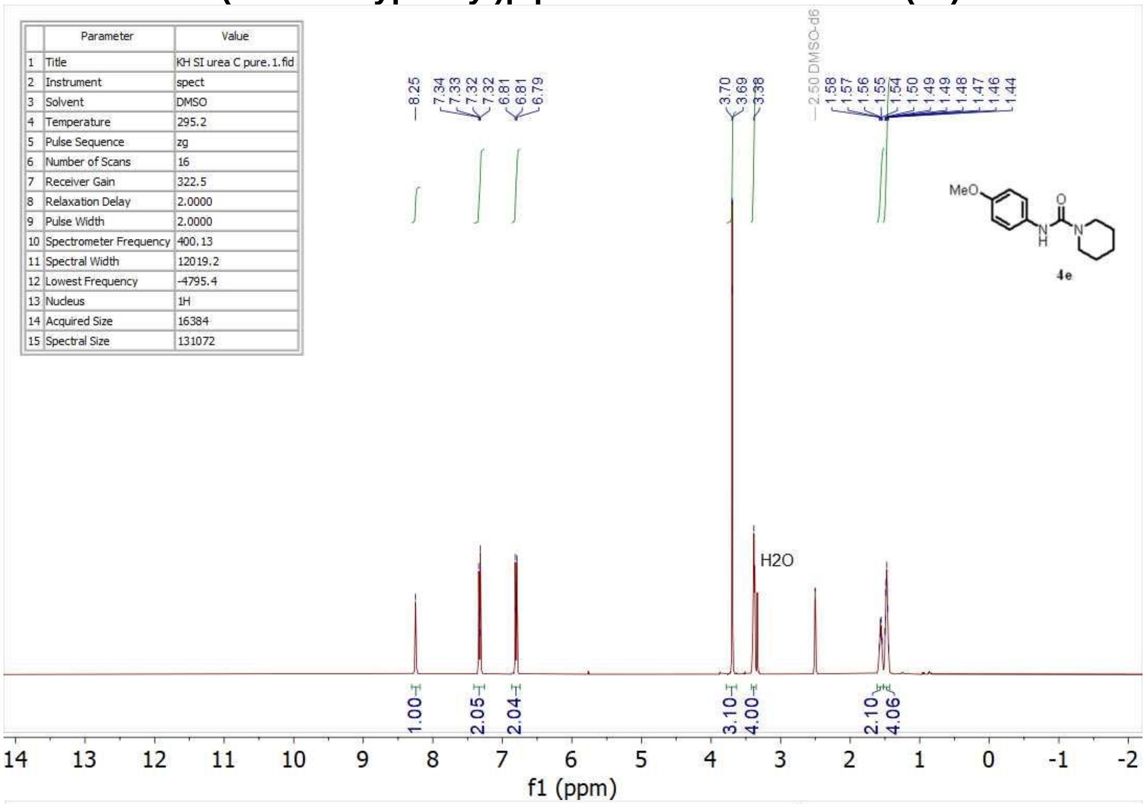

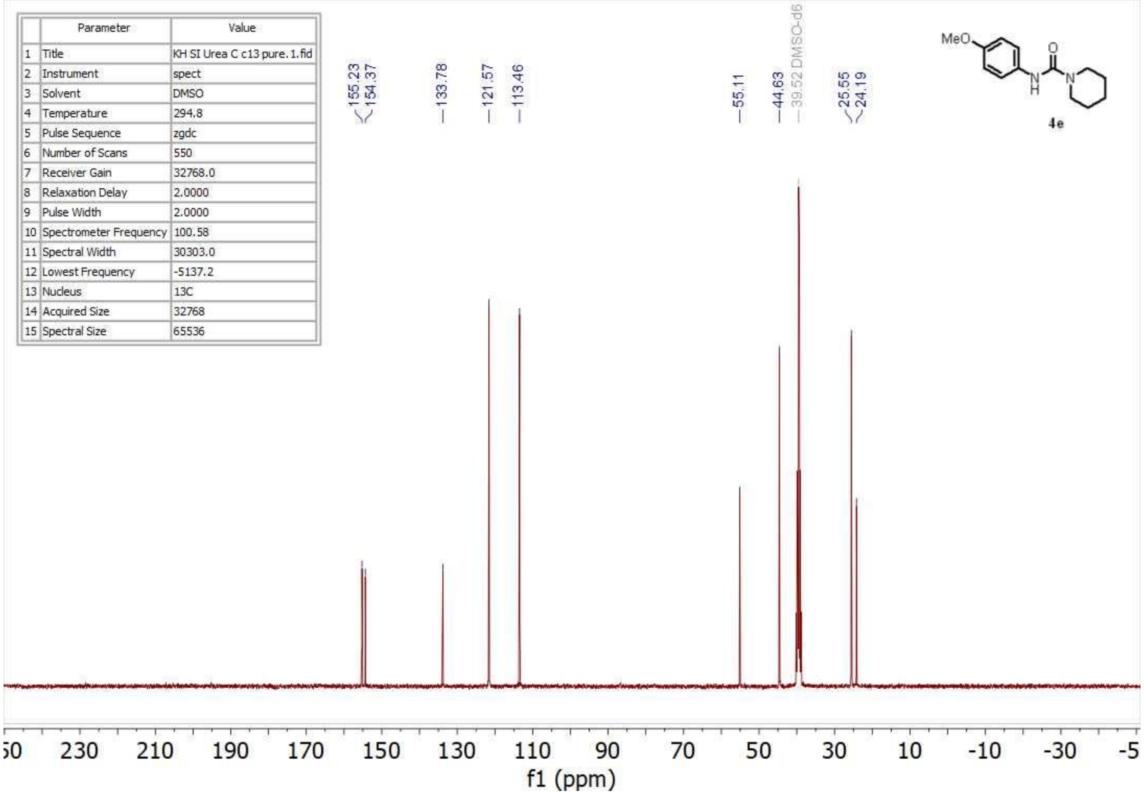

N-(4-(trifluoromethyl)phenyl)piperidine-1-carboxamide (4f)

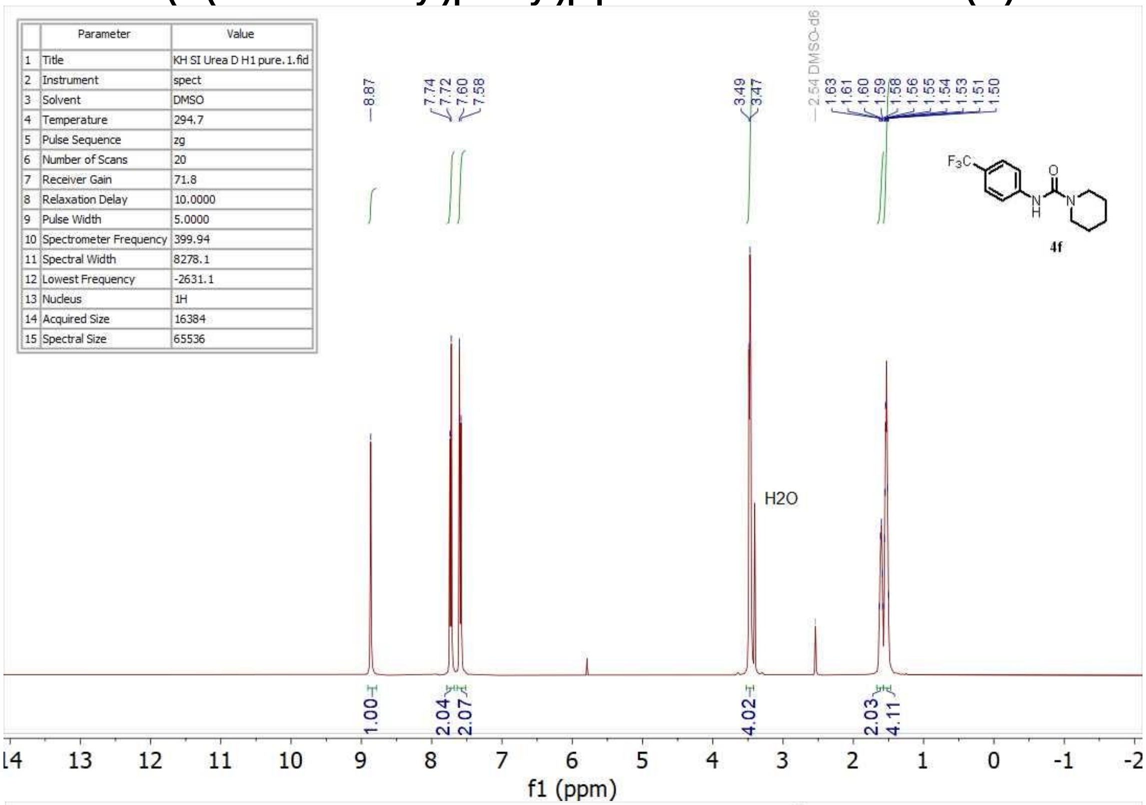

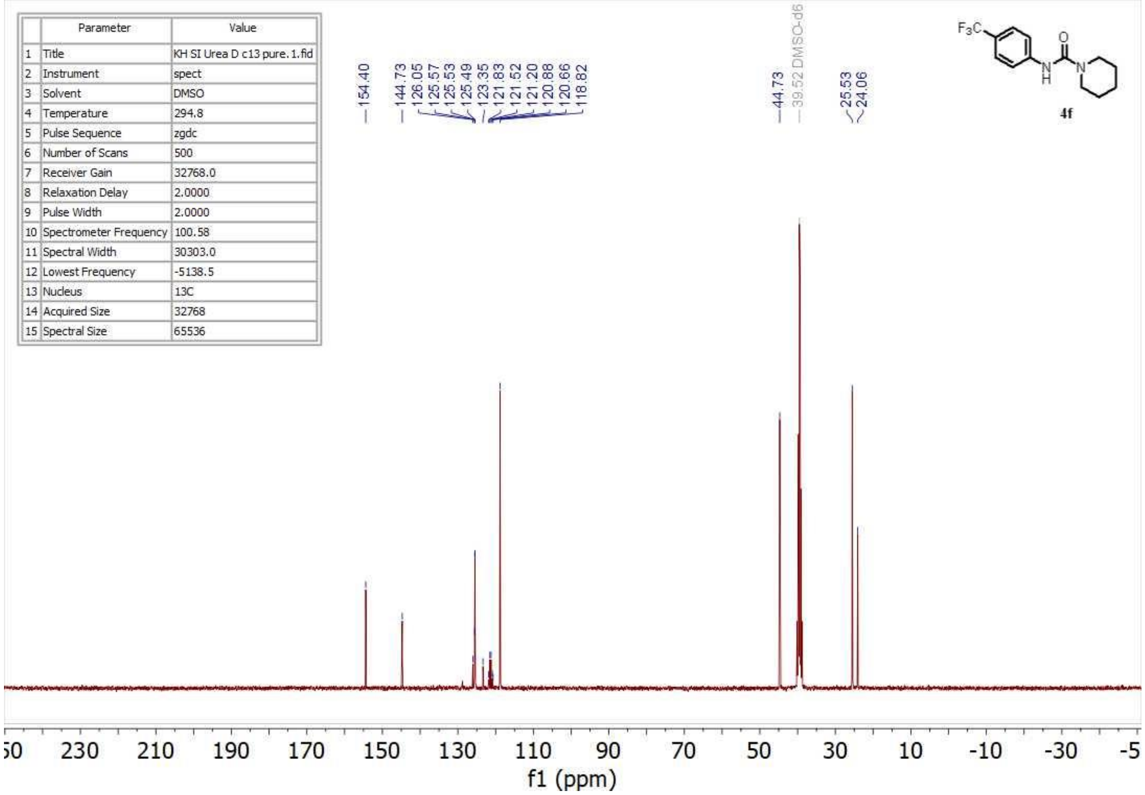

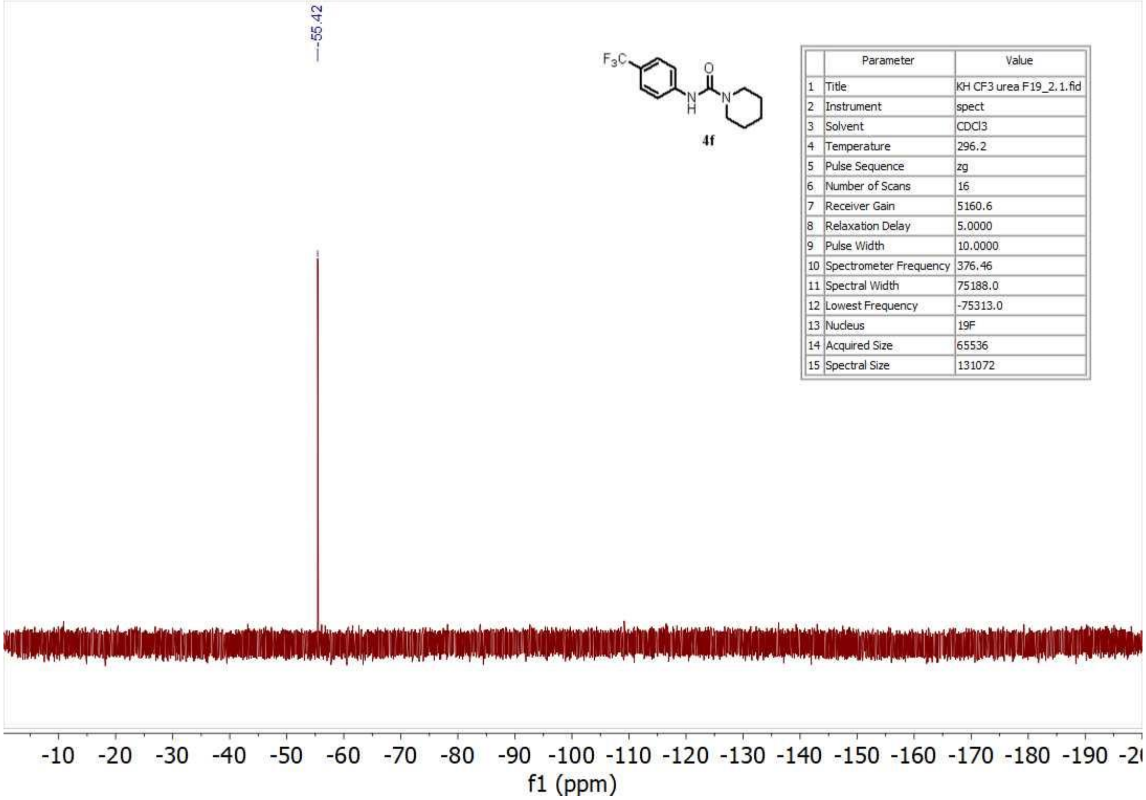

-55.42
F3C
O
N
H
N
4f
Parameter | Value
1 Title | KH CF3 urea F19_2.1.fid
2 Instrument | spect
3 Solvent | CDCl3
4 Temperature | 296.2
5 Pulse Sequence | zg
6 Number of Scans | 16
7 Receiver Gain | 5160.6
8 Relaxation Delay | 5.0000
9 Pulse Width | 10.0000
10 Spectrometer Frequency | 376.46
11 Spectral Width | 75188.0
12 Lowest Frequency | -75313.0
13 Nucleus | 19F
14 Acquired Size | 65536
15 Spectral Size | 131072
-10 -20 -30 -40 -50 -60 -70 -80 -90 -100 -110 -120 -130 -140 -150 -160 -170 -180 -190
f1 (ppm)

_N_-methyl-_N_-phenylpiperidine-1-carboxamide (4g)

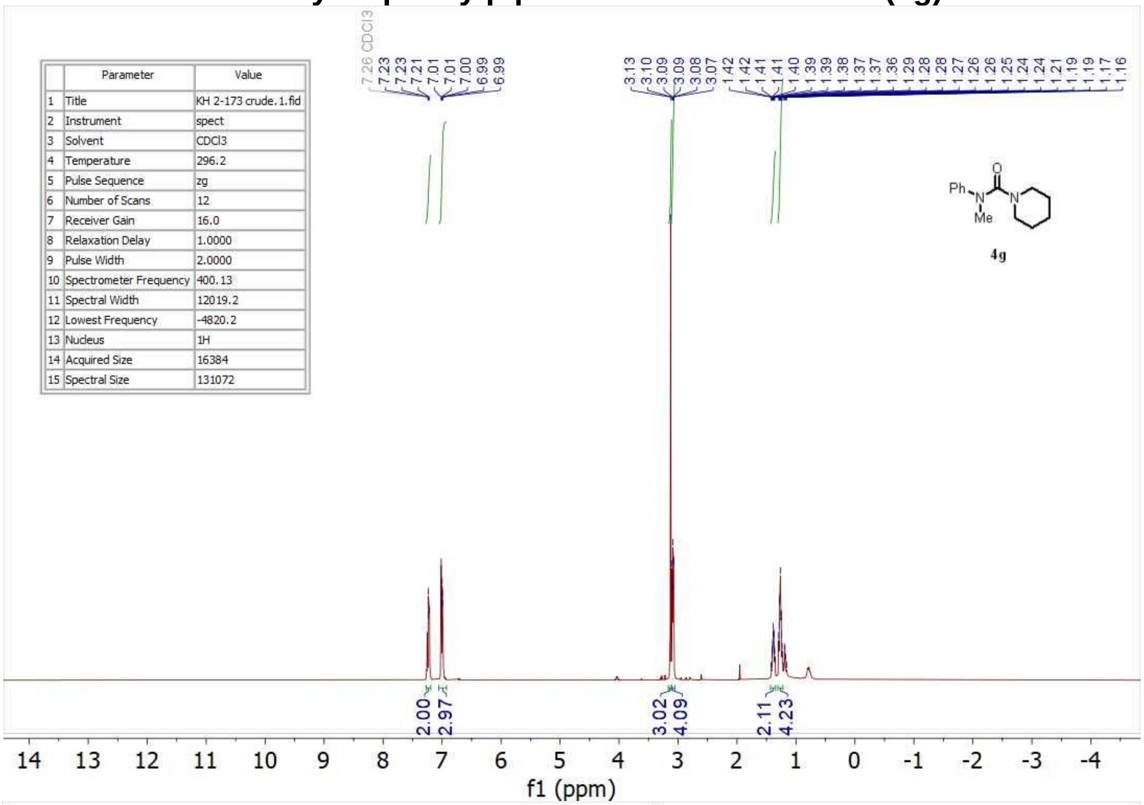

	Parameter	Value
1	Title	KH 2-173 crude.1.fid
2	Instrument	spect
3	Solvent	CDCl3
4	Temperature	296.2
5	Pulse Sequence	zg
6	Number of Scans	12
7	Receiver Gain	16.0
8	Relaxation Delay	1.0000
9	Pulse Width	2.0000
10	Spectrometer Frequency	400.13
11	Spectral Width	12019.2
12	Lowest Frequency	-4820.2
13	Nucleus	1H
14	Acquired Size	16384
15	Spectral Size	131072

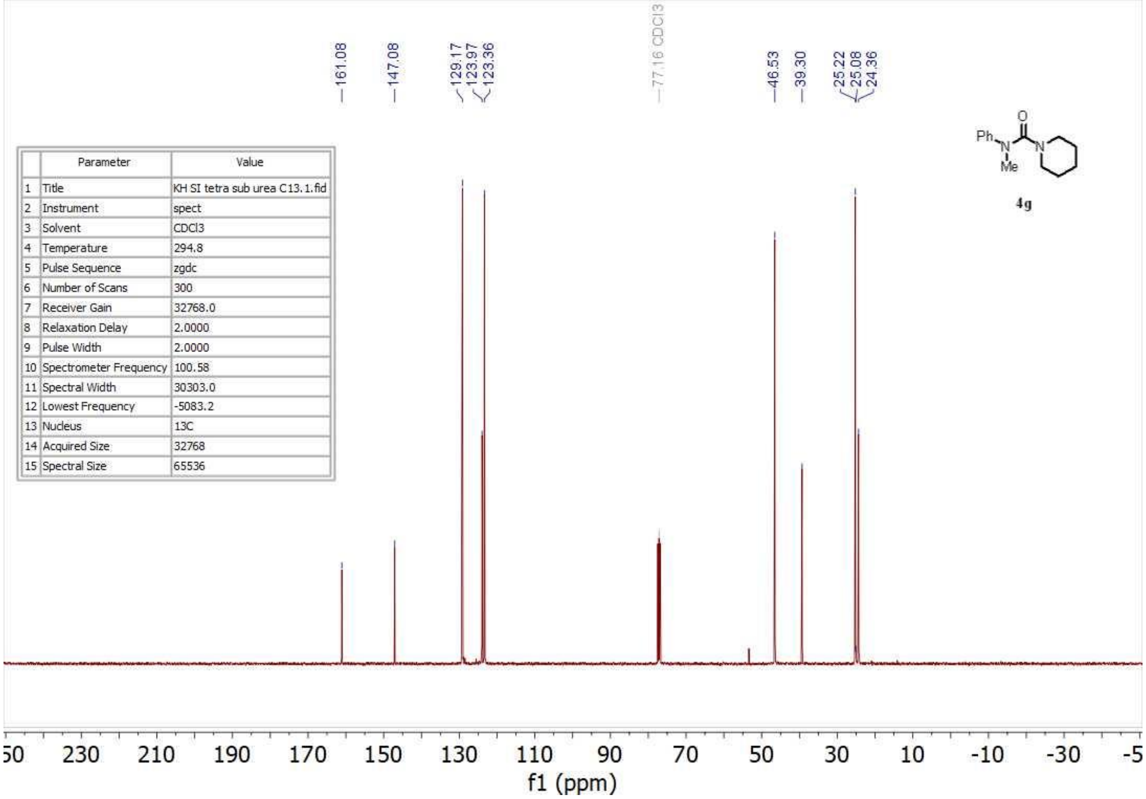

	Parameter	Value
1	Title	KH SI tetra sub urea C13.1.fid
2	Instrument	spect
3	Solvent	CDCl3
4	Temperature	294.8
5	Pulse Sequence	zgdc
6	Number of Scans	300
7	Receiver Gain	32768.0
8	Relaxation Delay	2.0000
9	Pulse Width	2.0000
10	Spectrometer Frequency	100.58
11	Spectral Width	30303.0
12	Lowest Frequency	-5083.2
13	Nucleus	13C
14	Acquired Size	32768
15	Spectral Size	65536

N-(4-methoxyphenyl)pyrrolidine-1-carboxamide (4h)

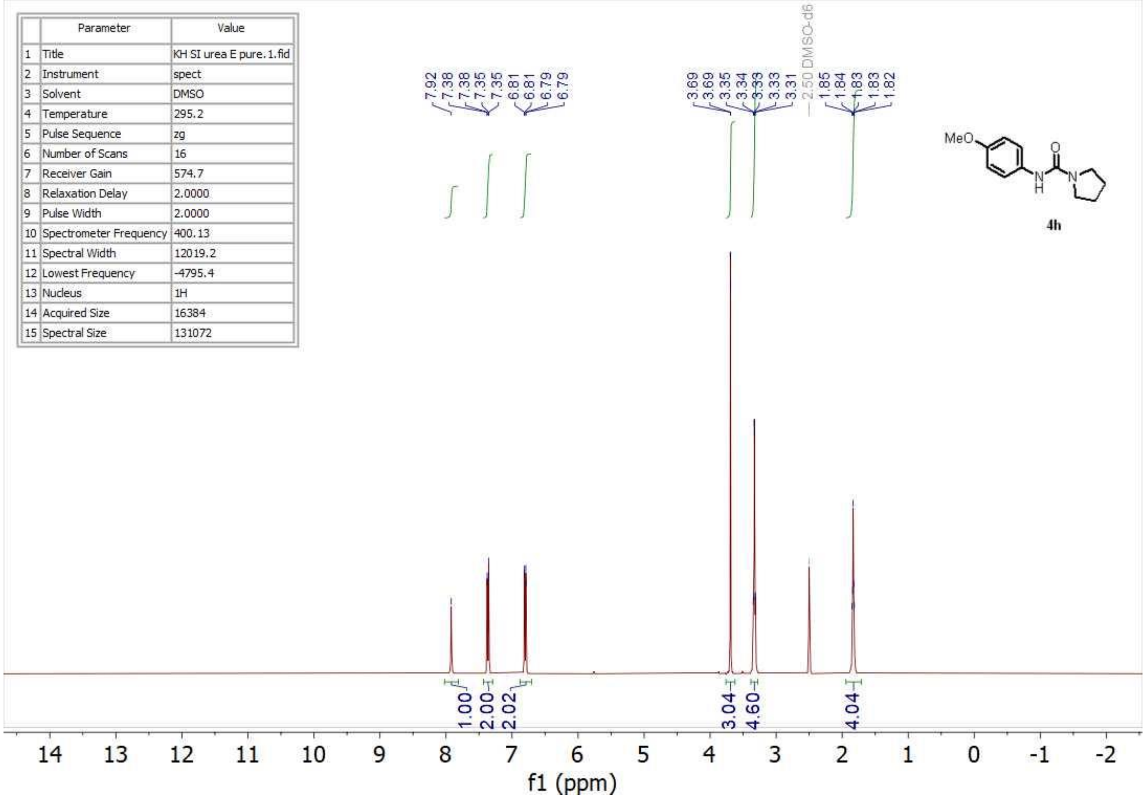

	Parameter	Value
1	Title	KH SI urea E pure.1.fid
2	Instrument	spect
3	Solvent	DMSO
4	Temperature	295.2
5	Pulse Sequence	zg
6	Number of Scans	16
7	Receiver Gain	574.7
8	Relaxation Delay	2.0000
9	Pulse Width	2.0000
10	Spectrometer Frequency	400.13
11	Spectral Width	12019.2
12	Lowest Frequency	-4795.4
13	Nucleus	1H
14	Acquired Size	16384
15	Spectral Size	131072

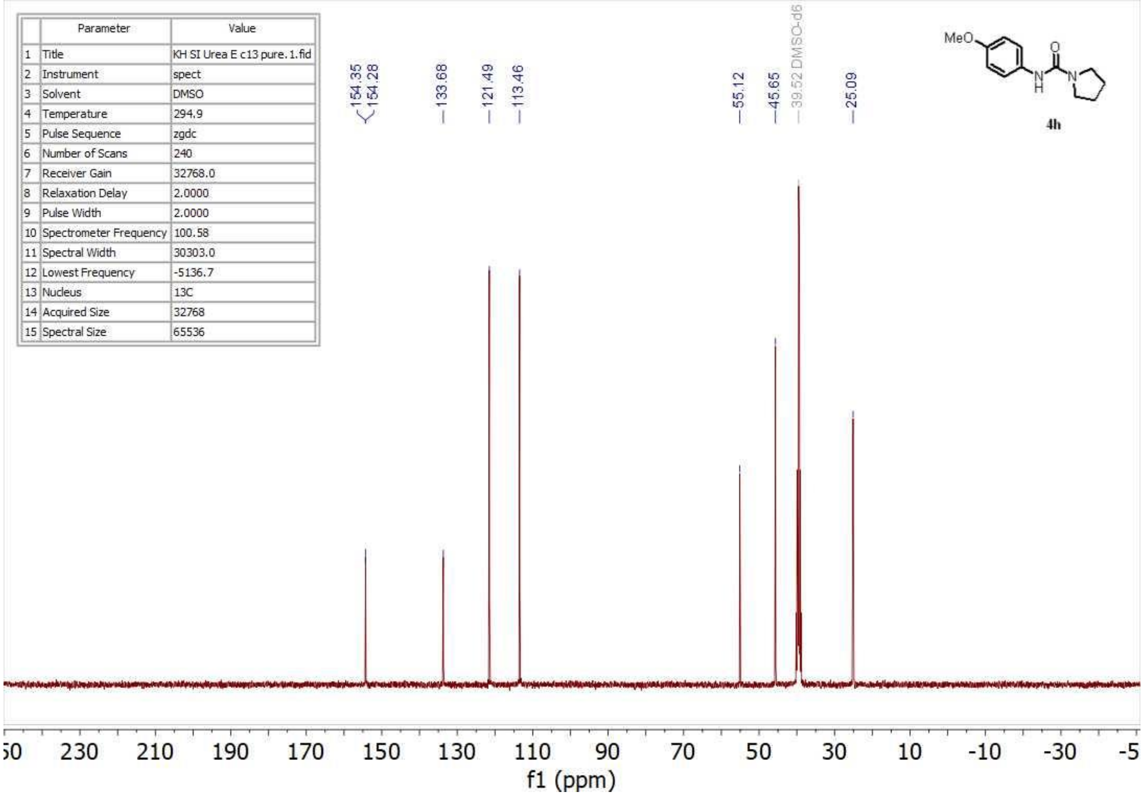

	Parameter	Value
1	Title	KH SI Urea E c13 pure.1.fid
2	Instrument	spect
3	Solvent	DMSO
4	Temperature	294.9
5	Pulse Sequence	zgdc
6	Number of Scans	240
7	Receiver Gain	32768.0
8	Relaxation Delay	2.0000
9	Pulse Width	2.0000
10	Spectrometer Frequency	100.58
11	Spectral Width	30303.0
12	Lowest Frequency	-5136.7
13	Nucleus	13C
14	Acquired Size	32768
15	Spectral Size	65536

N-(4-methoxyphenyl)morpholine-4-carboxamide (4i)

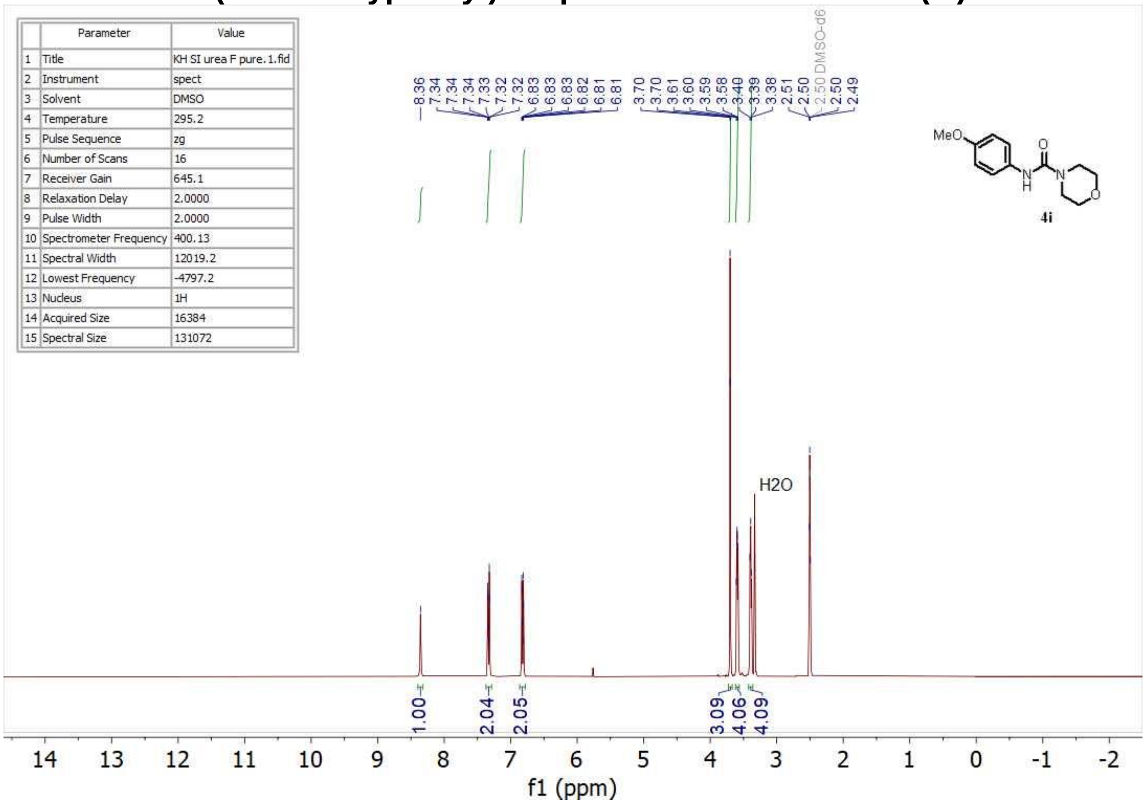

	Parameter	Value
1	Title	KH SI urea F pure.1.fid
2	Instrument	spect
3	Solvent	DMSO
4	Temperature	295.2
5	Pulse Sequence	zg
6	Number of Scans	16
7	Receiver Gain	645.1
8	Relaxation Delay	2.0000
9	Pulse Width	2.0000
10	Spectrometer Frequency	400.13
11	Spectral Width	12019.2
12	Lowest Frequency	-4797.2
13	Nucleus	1H
14	Acquired Size	16384
15	Spectral Size	131072

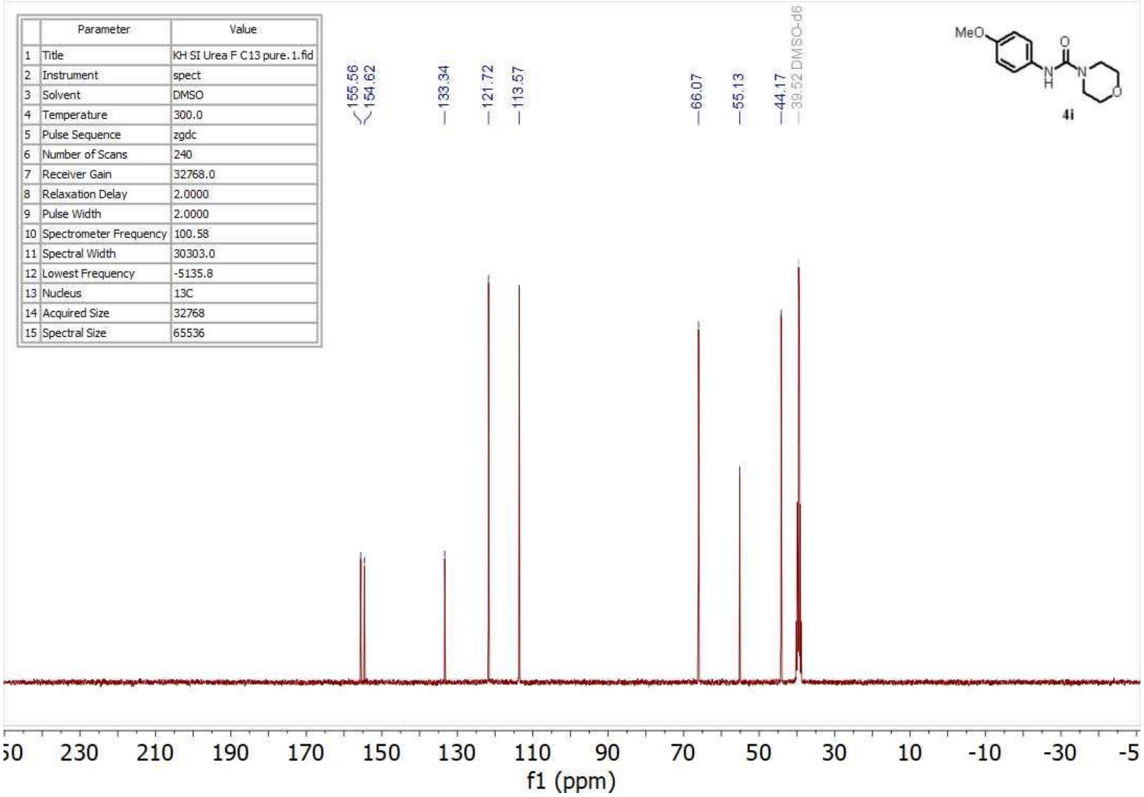

	Parameter	Value
1	Title	KH SI Urea F C13 pure.1.fid
2	Instrument	spect
3	Solvent	DMSO
4	Temperature	300.0
5	Pulse Sequence	zgdc
6	Number of Scans	240
7	Receiver Gain	32768.0
8	Relaxation Delay	2.0000
9	Pulse Width	2.0000
10	Spectrometer Frequency	100.58
11	Spectral Width	30303.0
12	Lowest Frequency	-5135.8
13	Nucleus	13C
14	Acquired Size	32768
15	Spectral Size	65536

1,1-diisopropyl-3-(4-methoxyphenyl)urea (4j)

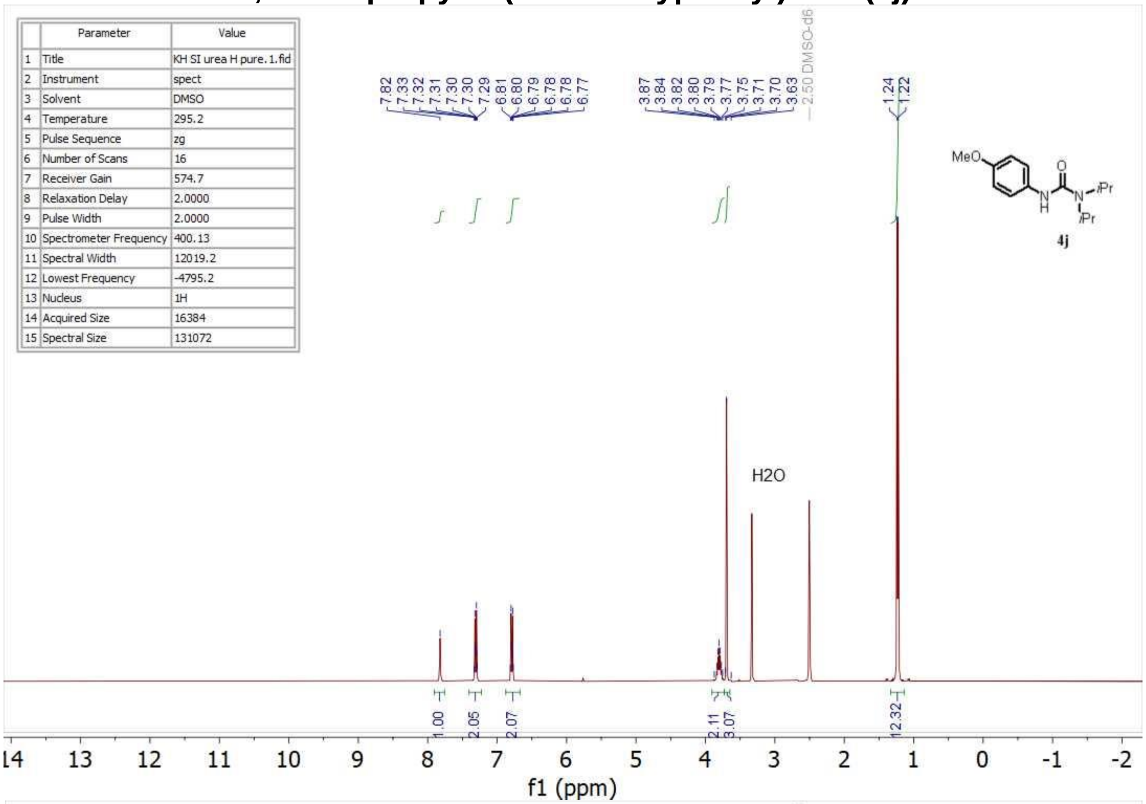

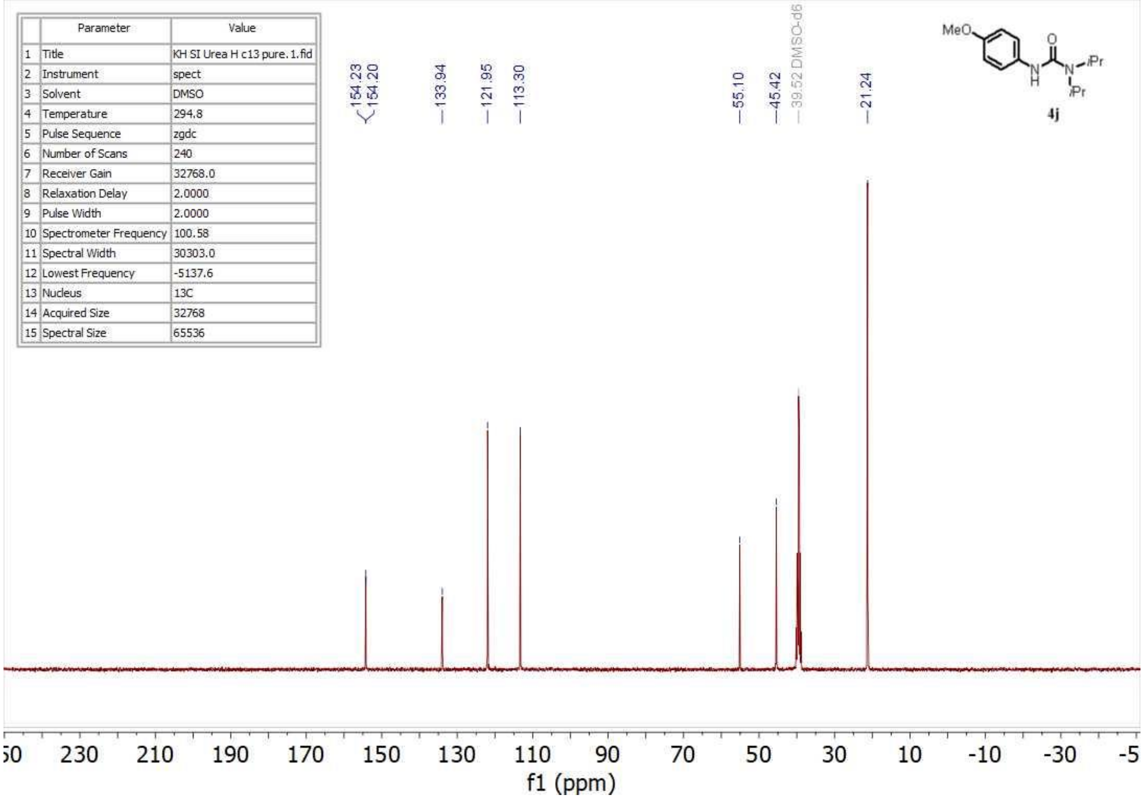

1,1-dicyclohexyl-3-(4-methoxyphenyl)urea (4k)

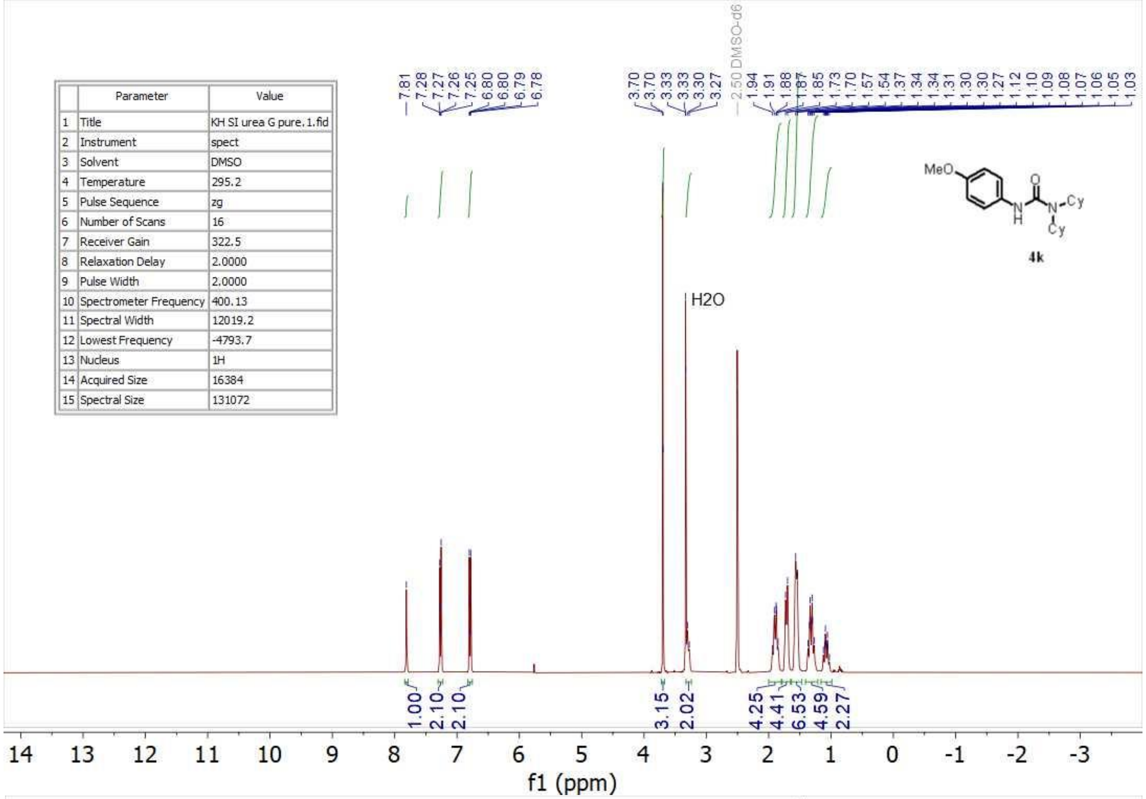

	Parameter	Value
1	Title	KH SI urea G pure.1.fid
2	Instrument	spect
3	Solvent	DMSO
4	Temperature	295.2
5	Pulse Sequence	zg
6	Number of Scans	16
7	Receiver Gain	322.5
8	Relaxation Delay	2.0000
9	Pulse Width	2.0000
10	Spectrometer Frequency	400.13
11	Spectral Width	12019.2
12	Lowest Frequency	-4793.7
13	Nucleus	1H
14	Acquired Size	16384
15	Spectral Size	131072

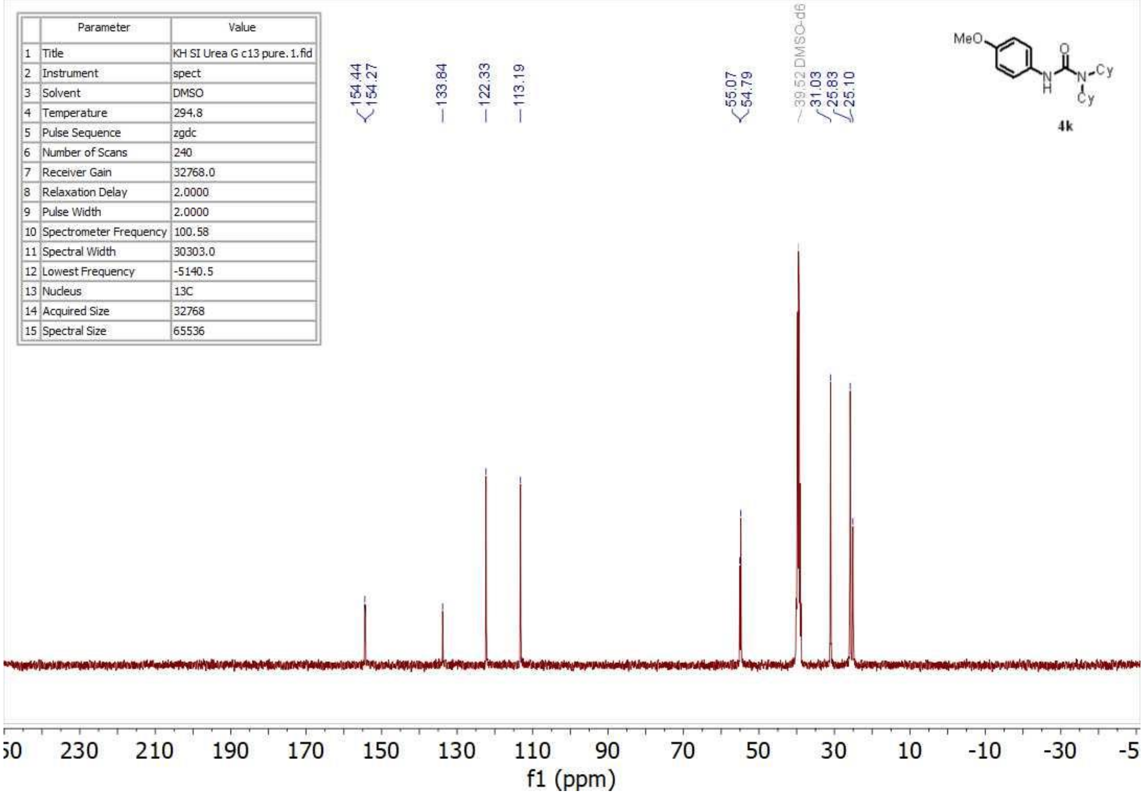

	Parameter	Value
1	Title	KH SI Urea G c13 pure.1.fid
2	Instrument	spect
3	Solvent	DMSO
4	Temperature	294.8
5	Pulse Sequence	zgdc
6	Number of Scans	240
7	Receiver Gain	32768.0
8	Relaxation Delay	2.0000
9	Pulse Width	2.0000
10	Spectrometer Frequency	100.58
11	Spectral Width	30303.0
12	Lowest Frequency	-5140.5
13	Nucleus	13C
14	Acquired Size	32768
15	Spectral Size	65536

(*E*)-2-styryl-2,3-dihydrobenzofuran (3ab)

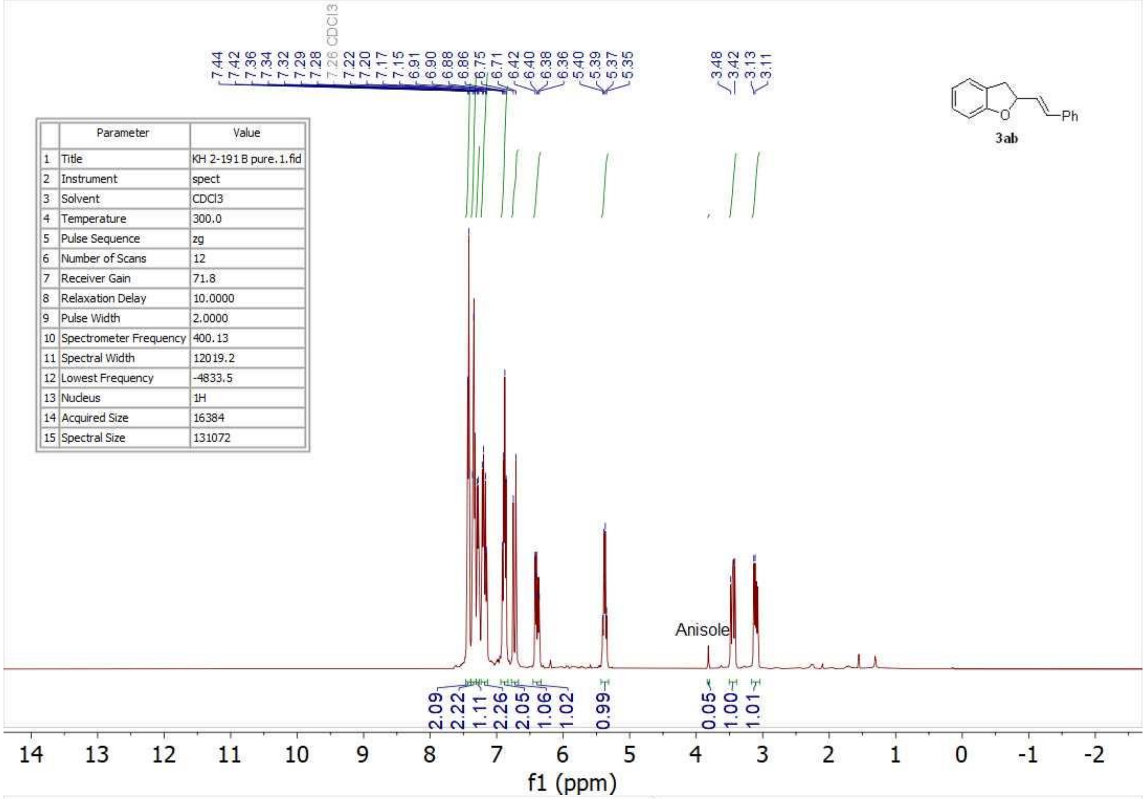

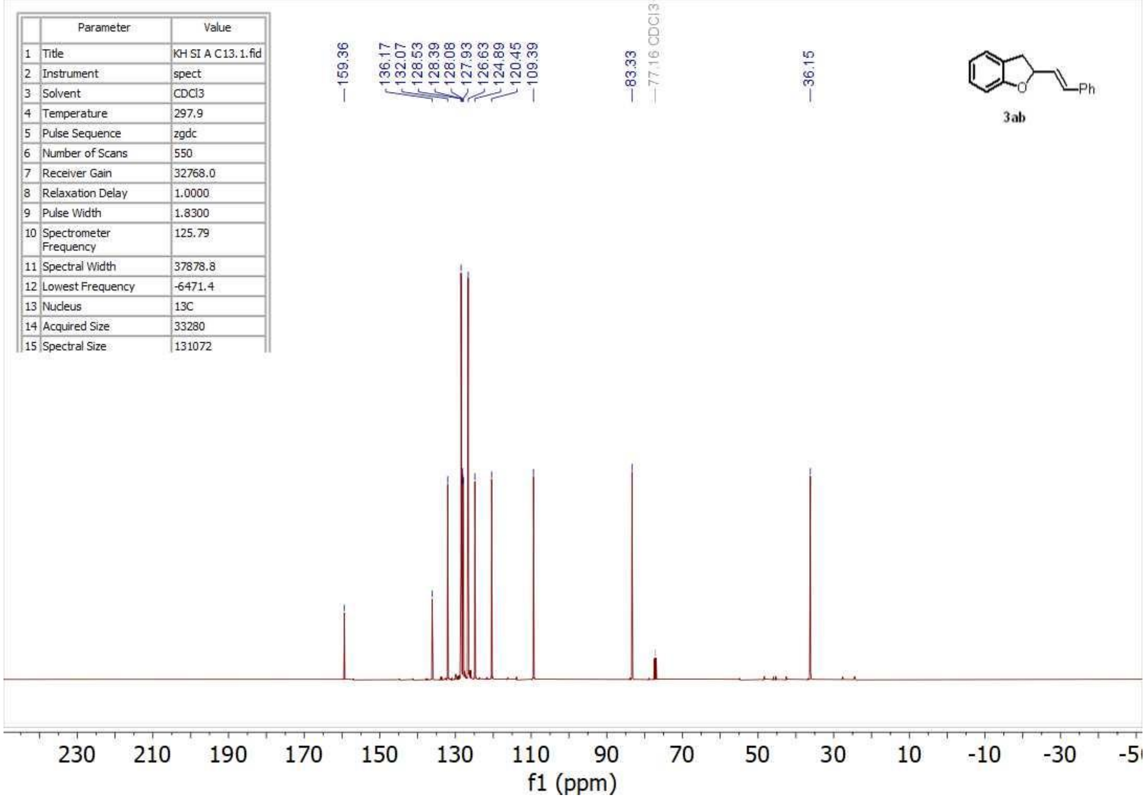

(*E*)-4-methyl-2-styryl-2,3-dihydrobenzofuran (3bb)

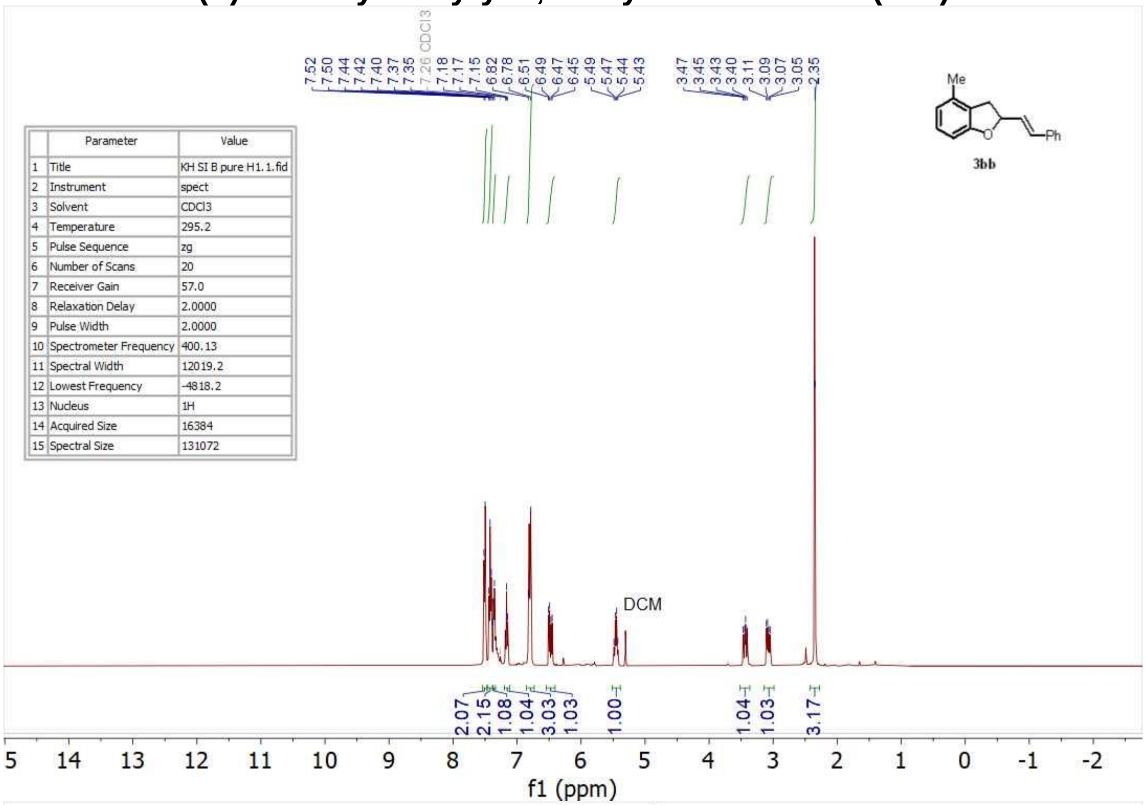

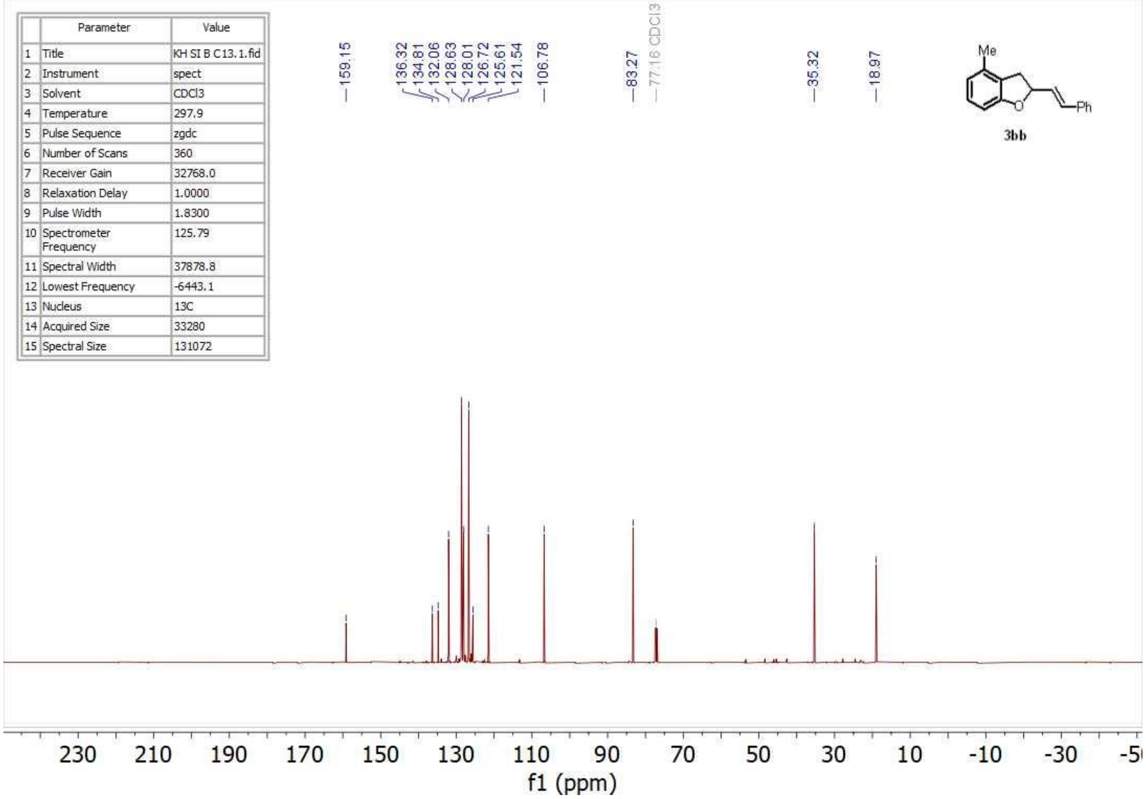

(*E*)-6-methyl-2-styryl-2,3-dihydrobenzofuran (3cb)

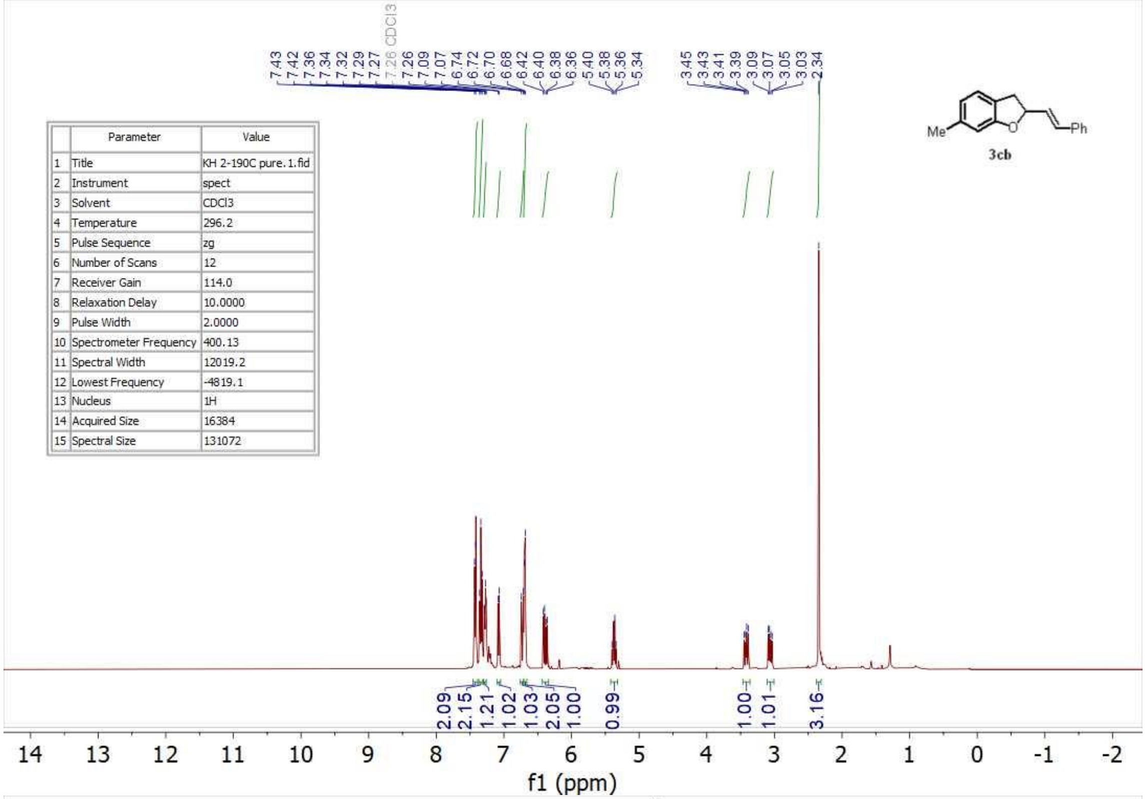

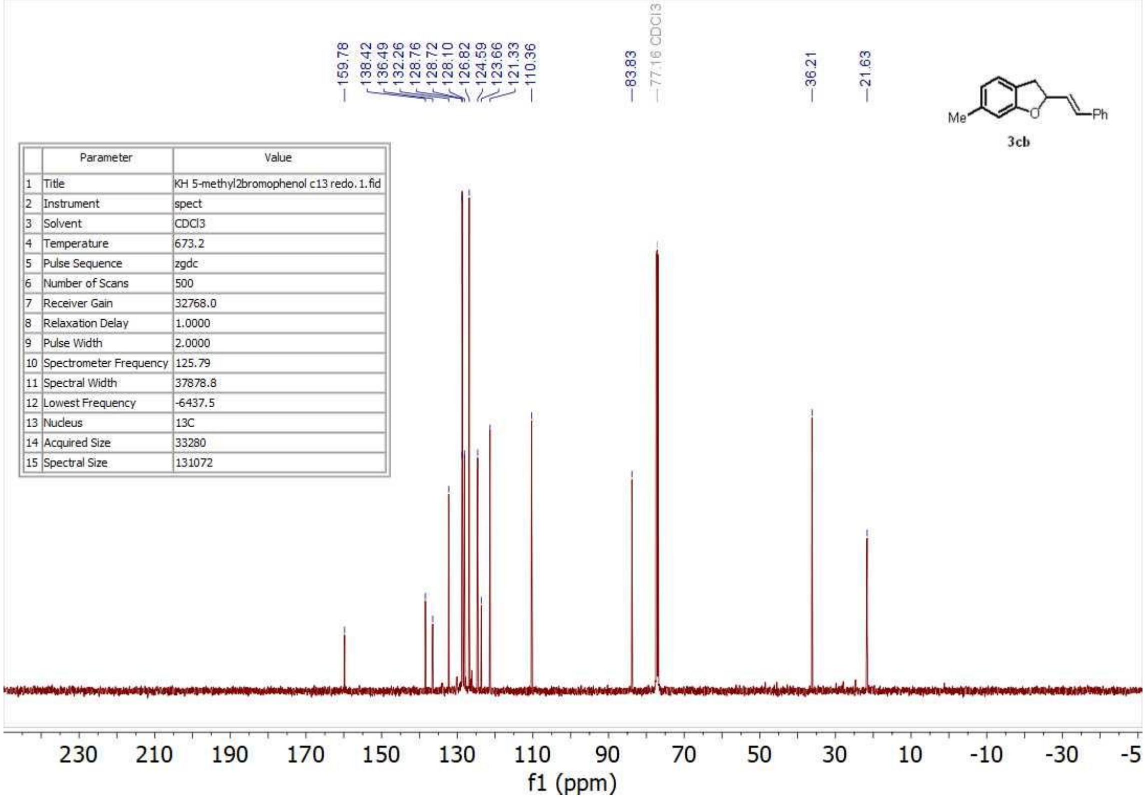

(*E*)-5-methyl-2-styryl-2,3-dihydrobenzofuran (3db)

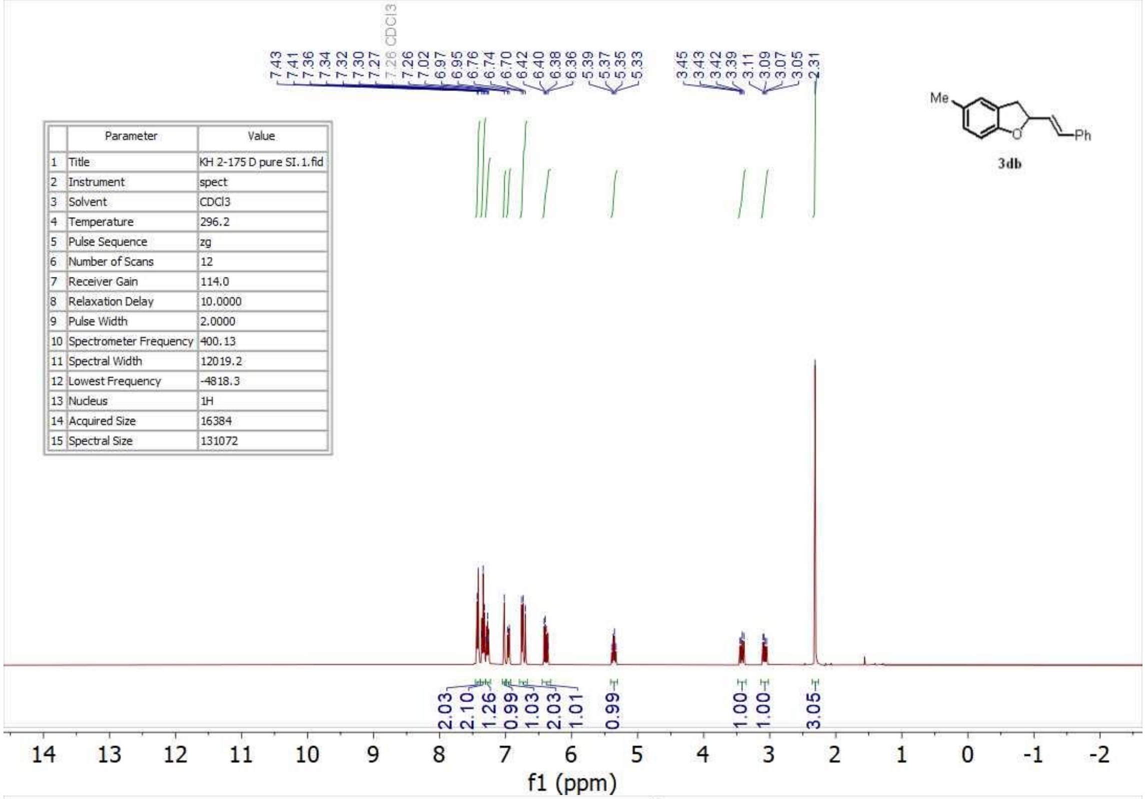

	Parameter	Value
1	Title	KH 2-175 D pure SI.1.fid
2	Instrument	spect
3	Solvent	CDCl3
4	Temperature	296.2
5	Pulse Sequence	zg
6	Number of Scans	12
7	Receiver Gain	114.0
8	Relaxation Delay	10.0000
9	Pulse Width	2.0000
10	Spectrometer Frequency	400.13
11	Spectral Width	12019.2
12	Lowest Frequency	-4818.3
13	Nucleus	1H
14	Acquired Size	16384
15	Spectral Size	131072

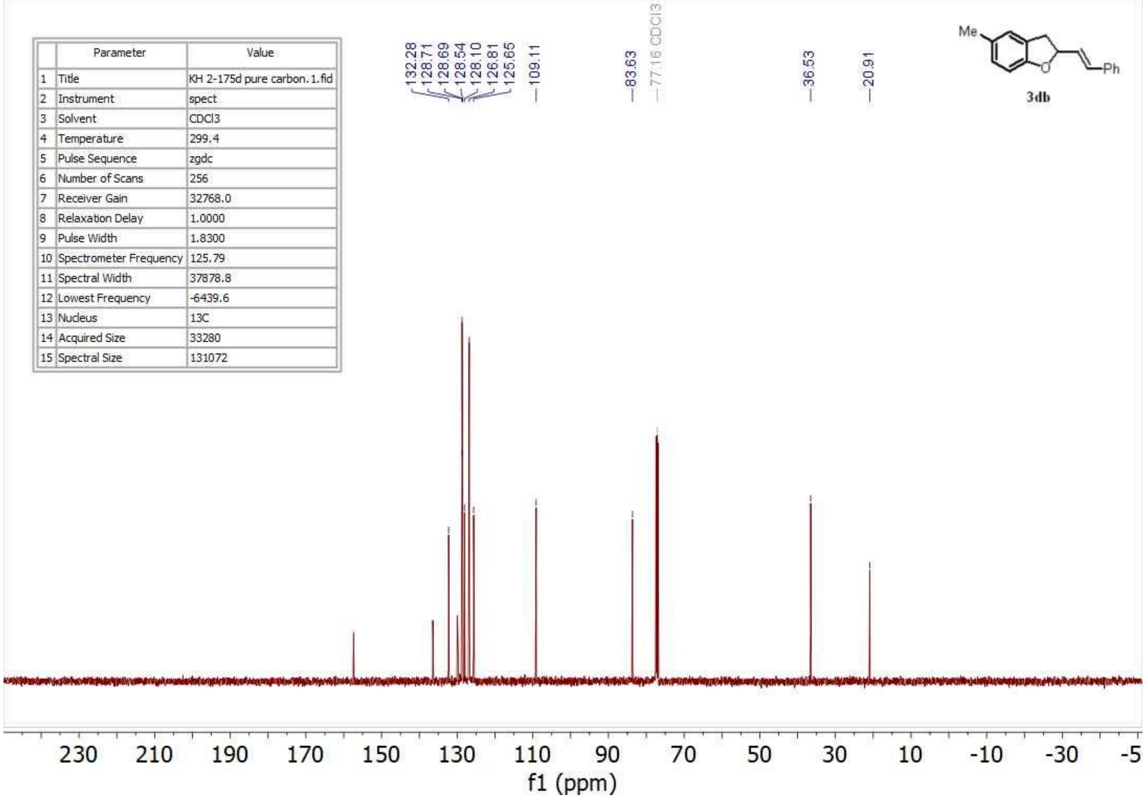

	Parameter	Value
1	Title	KH 2-175d pure carbon.1.fid
2	Instrument	spect
3	Solvent	CDCl3
4	Temperature	299.4
5	Pulse Sequence	zgdc
6	Number of Scans	256
7	Receiver Gain	32768.0
8	Relaxation Delay	1.0000
9	Pulse Width	1.8300
10	Spectrometer Frequency	125.79
11	Spectral Width	37878.8
12	Lowest Frequency	-6439.6
13	Nucleus	13C
14	Acquired Size	33280
15	Spectral Size	131072

(*E*)-7-methyl-2-styryl-2,3-dihydrobenzofuran (3eb)

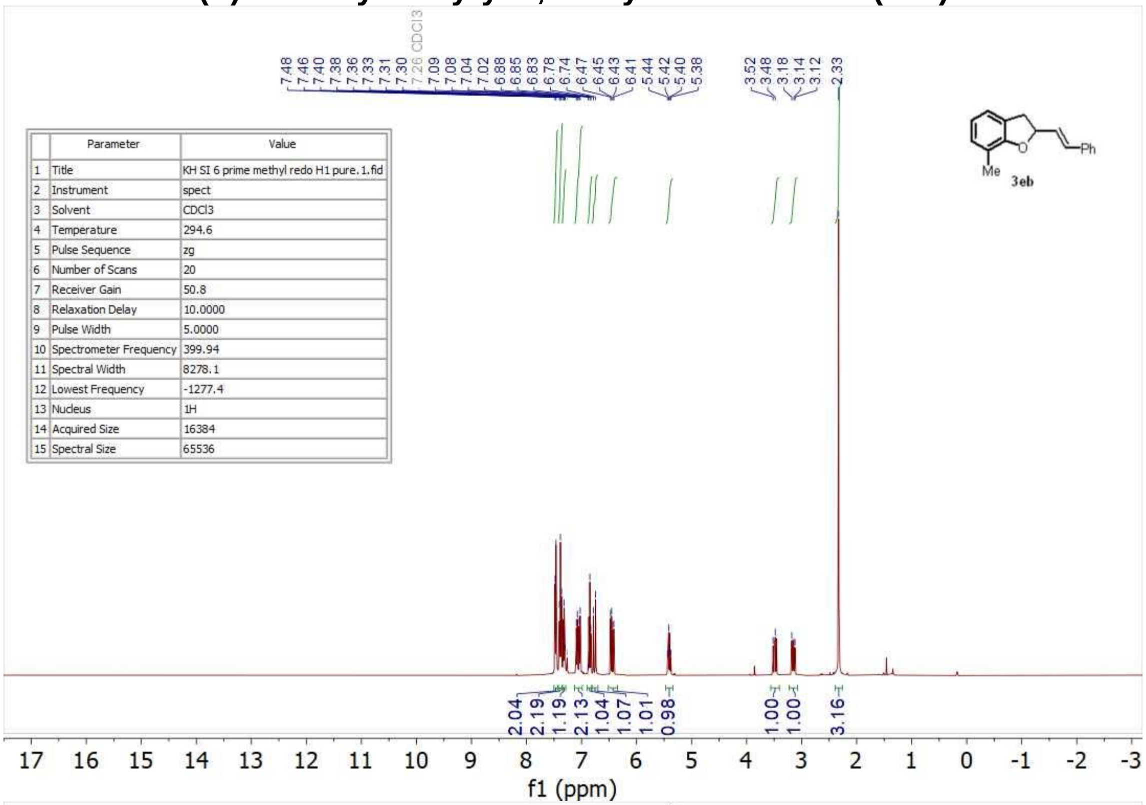

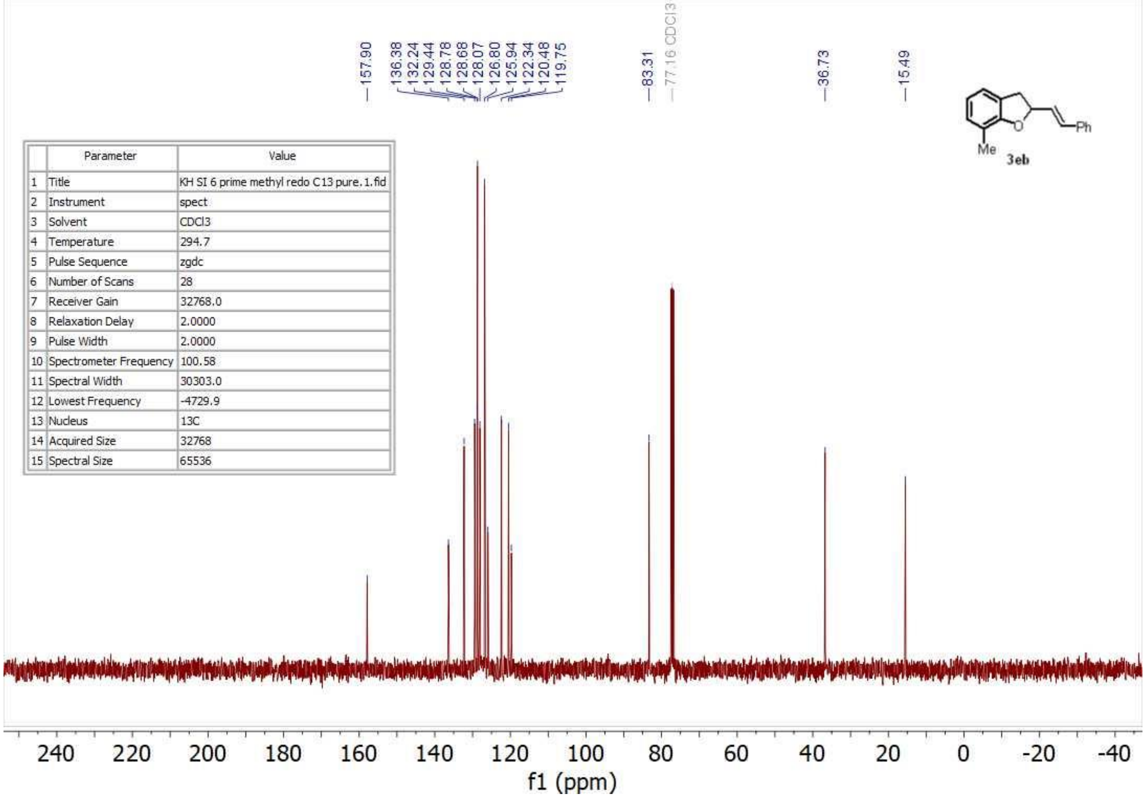

(E)-6-methoxy-2-styryl-2,3-dihydrobenzofuran (3fb)

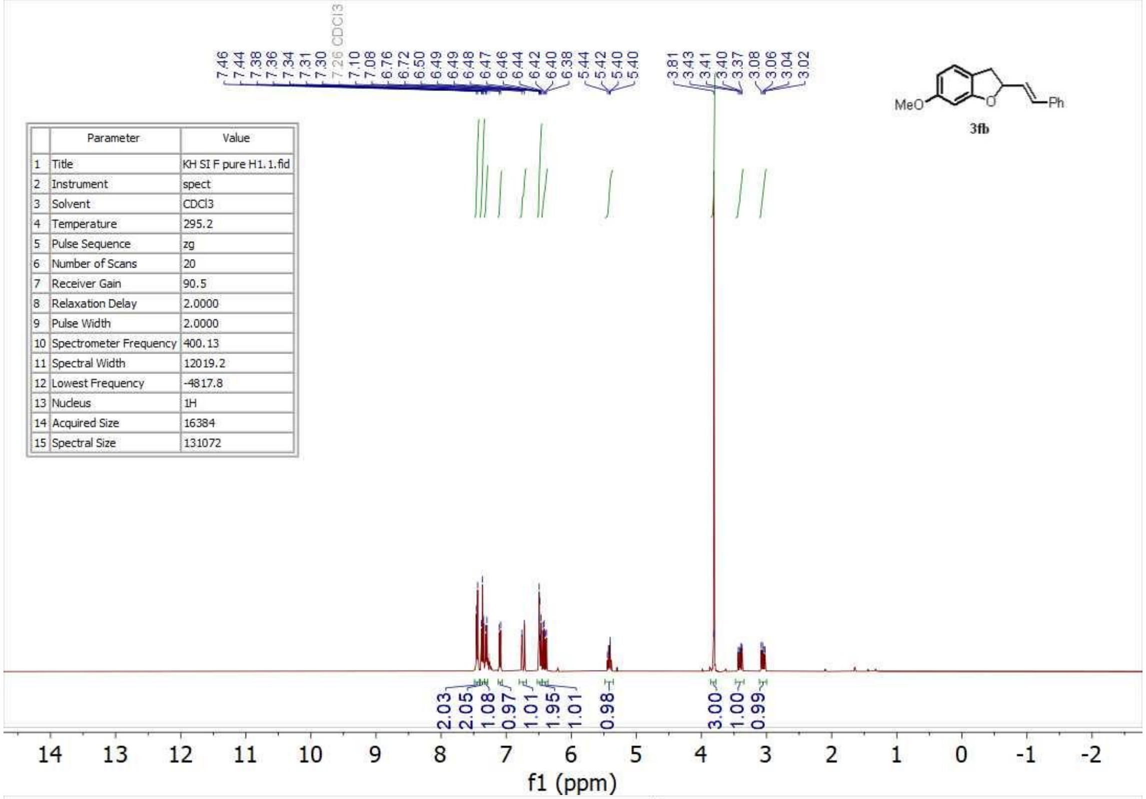

	Parameter	Value
1	Title	KH SI F pure H1.1.fid
2	Instrument	spect
3	Solvent	CDCl3
4	Temperature	295.2
5	Pulse Sequence	zg
6	Number of Scans	20
7	Receiver Gain	90.5
8	Relaxation Delay	2.0000
9	Pulse Width	2.0000
10	Spectrometer Frequency	400.13
11	Spectral Width	12019.2
12	Lowest Frequency	-4817.8
13	Nucleus	1H
14	Acquired Size	16384
15	Spectral Size	131072

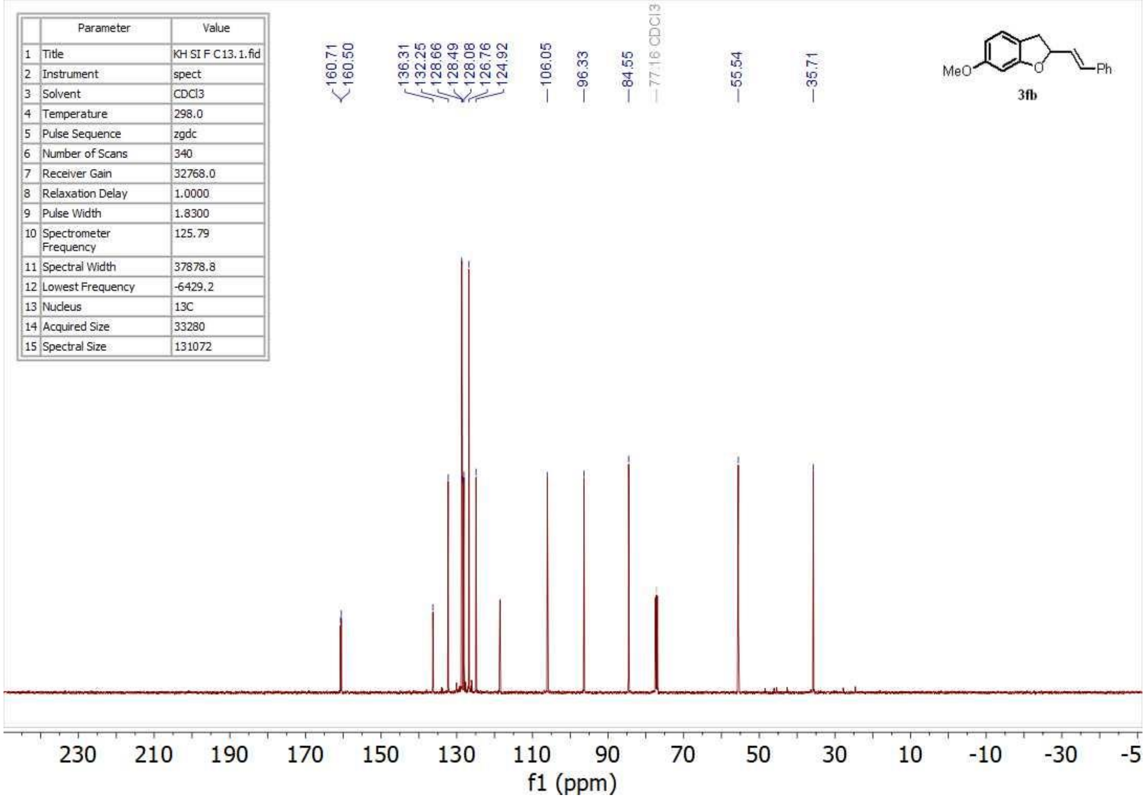

	Parameter	Value
1	Title	KH SI F C13.1.fid
2	Instrument	spect
3	Solvent	CDCl3
4	Temperature	298.0
5	Pulse Sequence	zgdc
6	Number of Scans	340
7	Receiver Gain	32768.0
8	Relaxation Delay	1.0000
9	Pulse Width	1.8300
10	Spectrometer Frequency	125.79
11	Spectral Width	37878.8
12	Lowest Frequency	-6429.2
13	Nucleus	13C
14	Acquired Size	33280
15	Spectral Size	131072

(*E*)-2-styryl-6-(trifluoromethyl)-2,3-dihydrobenzofuran (3gb)

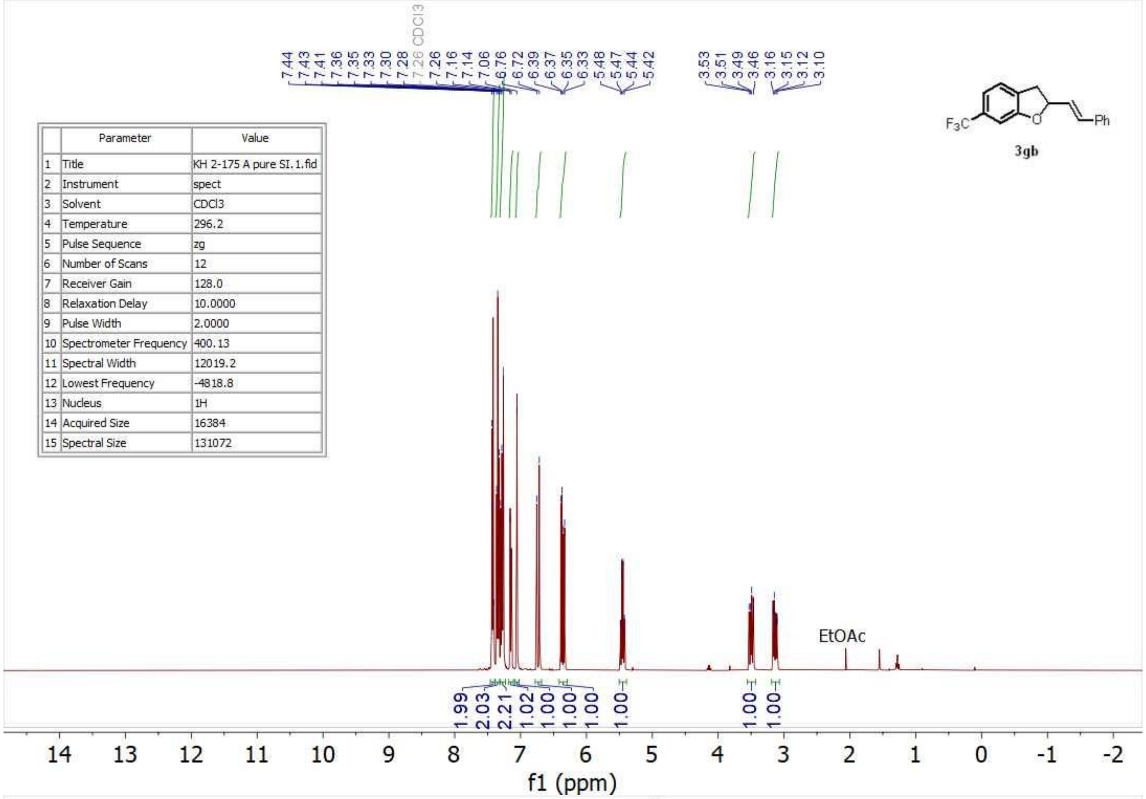

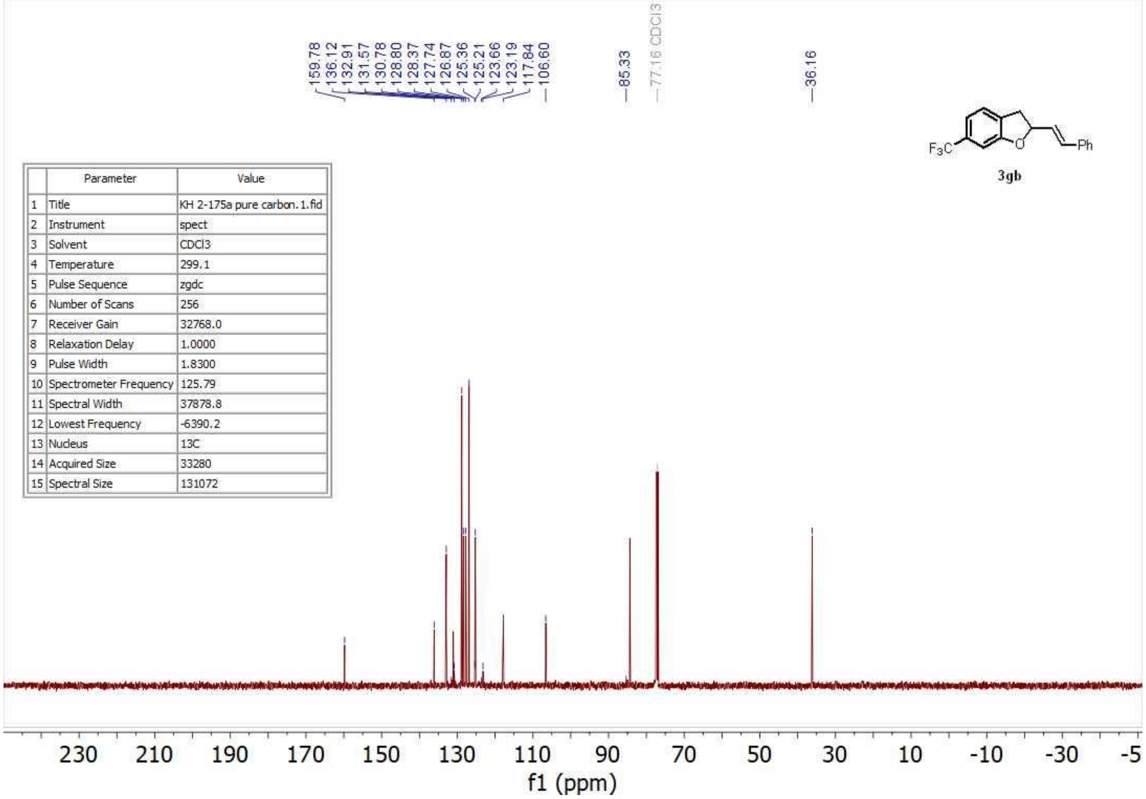

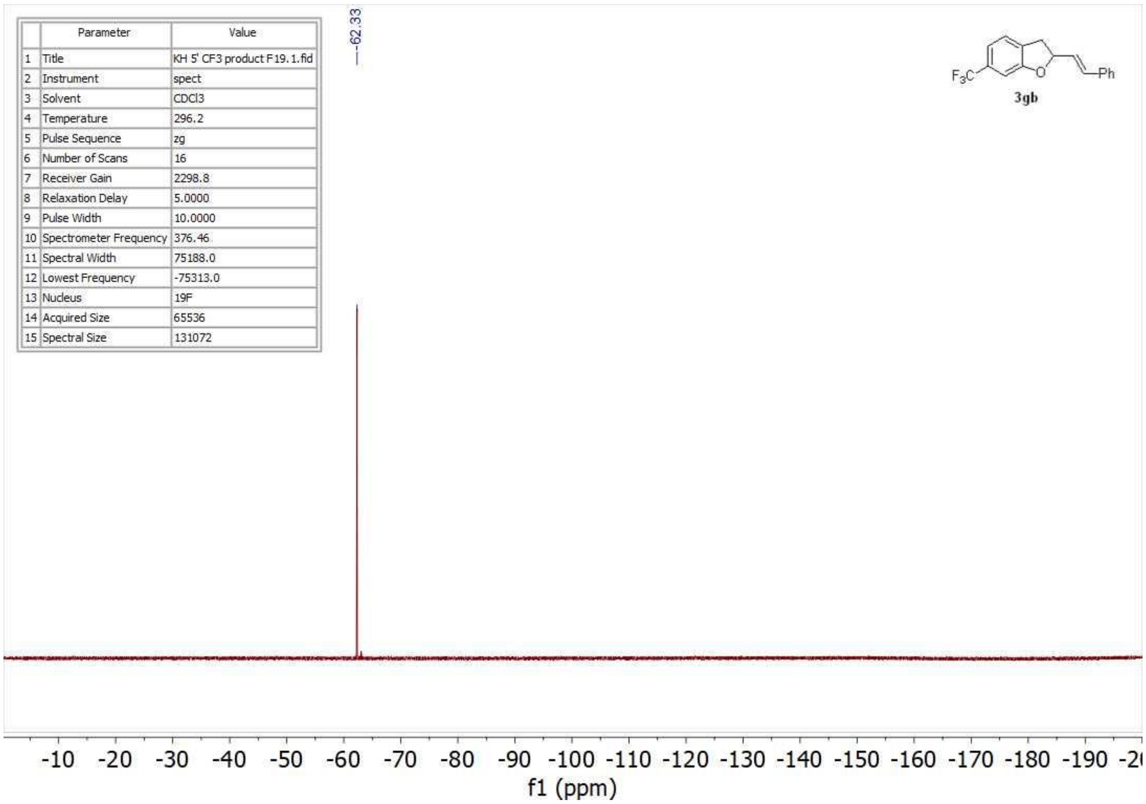

(E)-5-methoxy-2-styryl-2,3-dihydrobenzofuran (3hb)

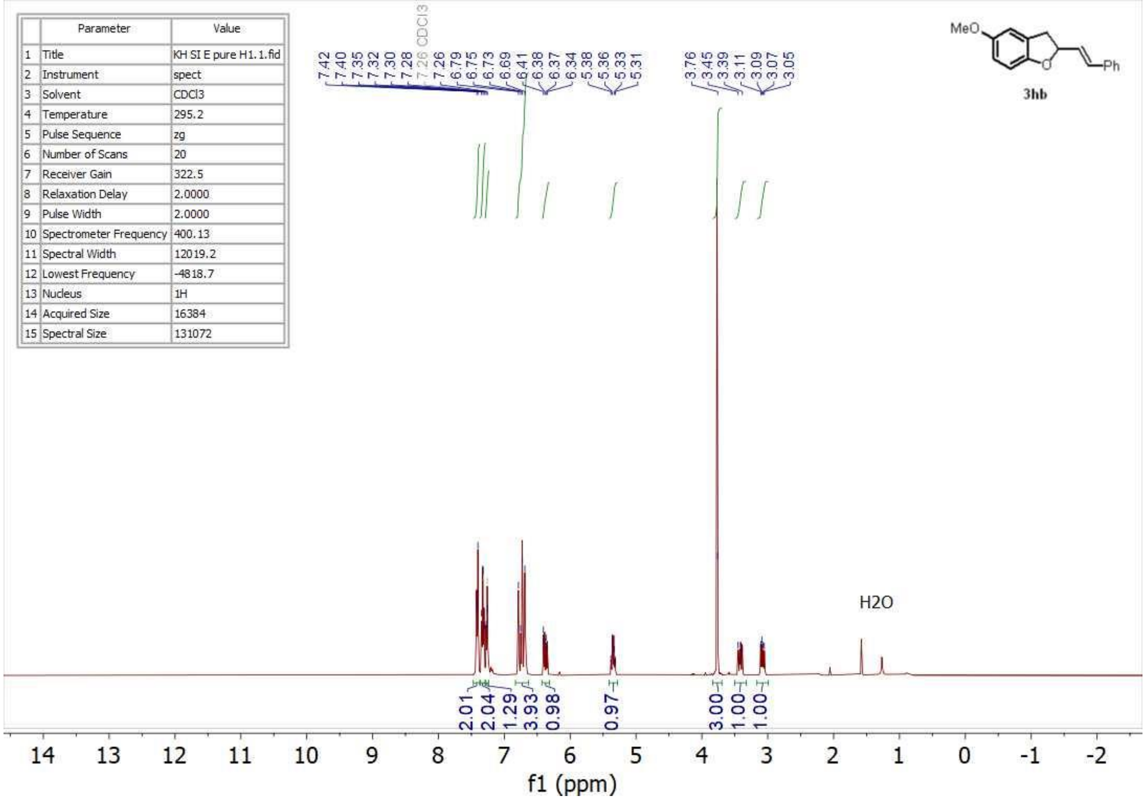

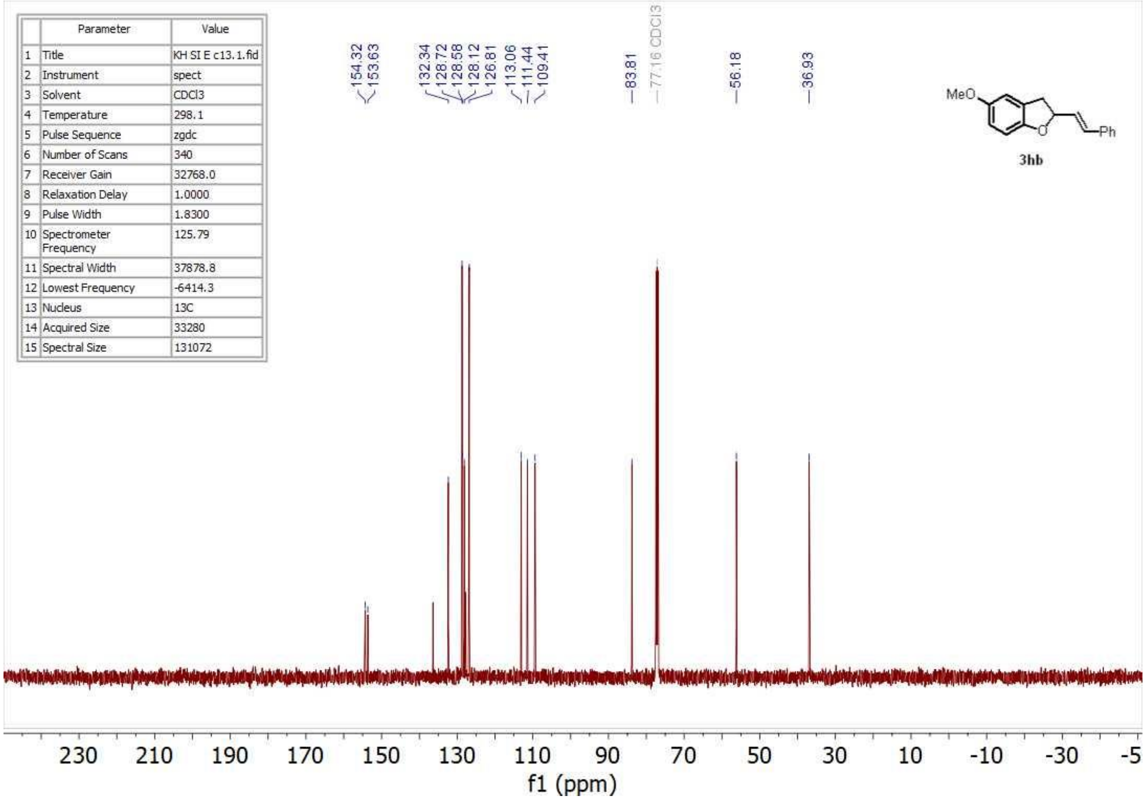

(*E*)-5-chloro-2-styryl-2,3-dihydrobenzofuran (3jb)

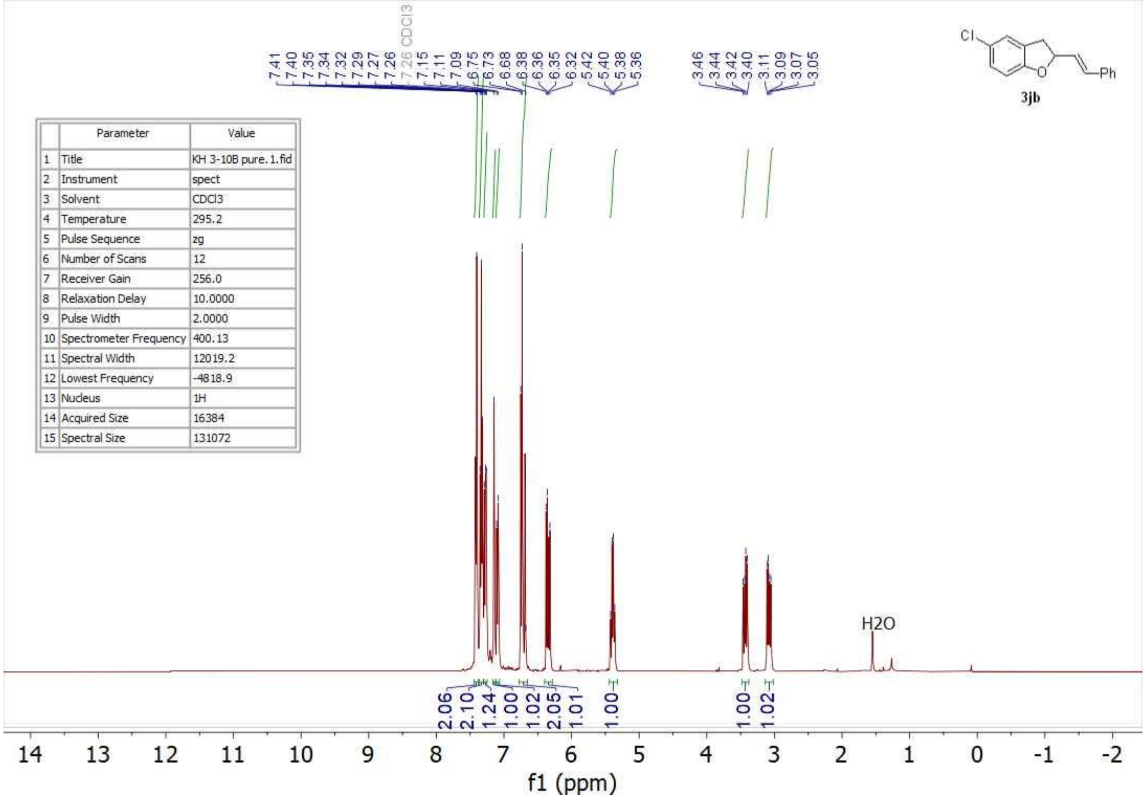

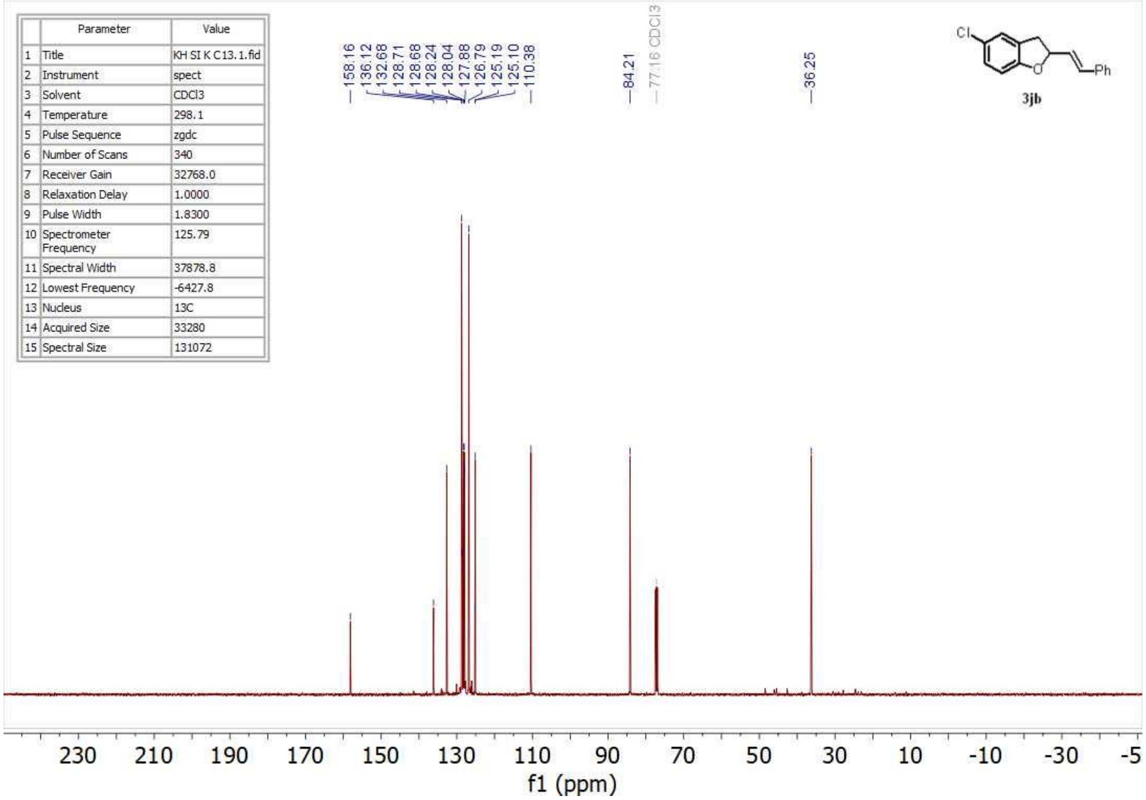

(*E*)-5-fluoro-2-styryl-2,3-dihydrobenzofuran (3kb)

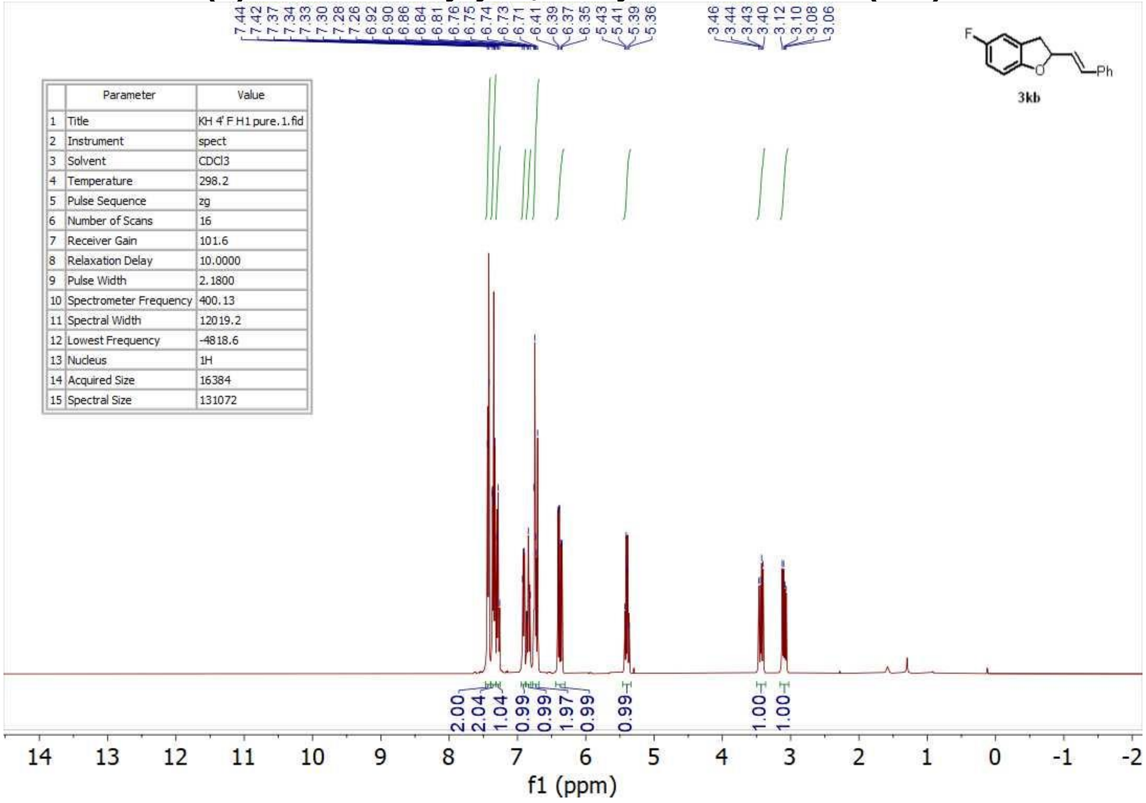

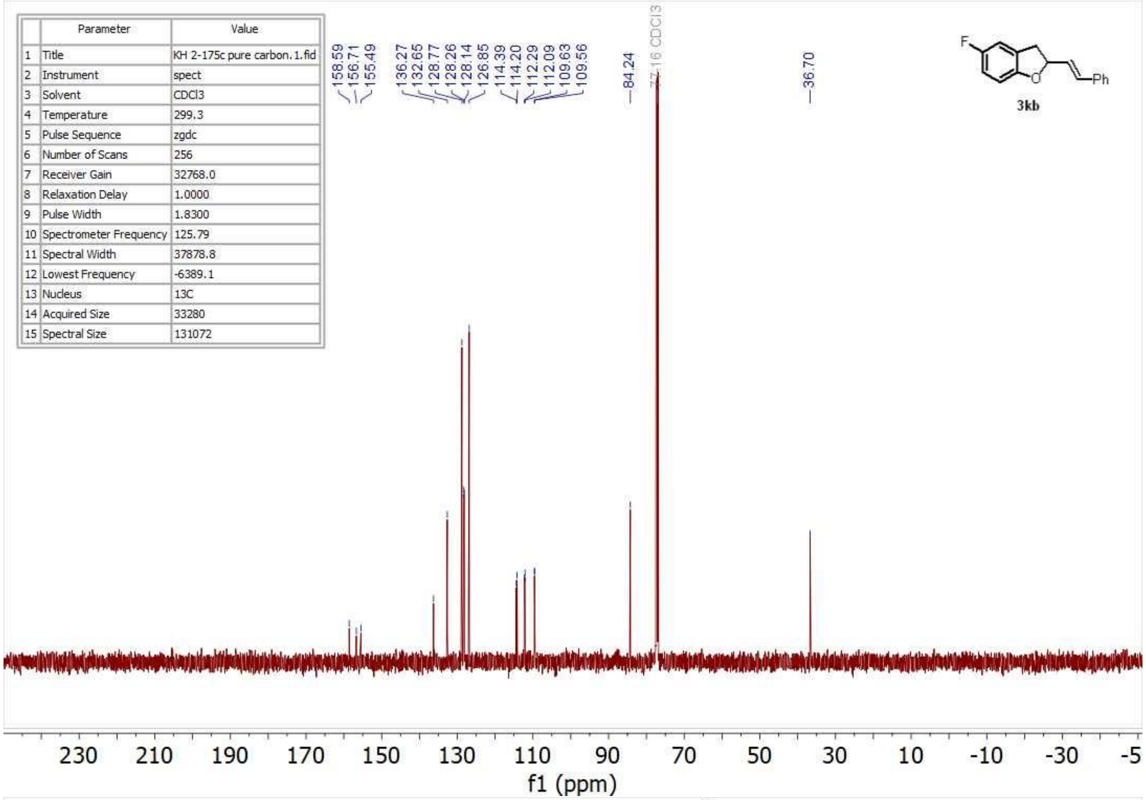

	Parameter	Value
1	Title	KH 2-175c pure carbon.1.fid
2	Instrument	spect
3	Solvent	CDCl3
4	Temperature	299.3
5	Pulse Sequence	zgdc
6	Number of Scans	256
7	Receiver Gain	32768.0
8	Relaxation Delay	1.0000
9	Pulse Width	1.8300
10	Spectrometer Frequency	125.79
11	Spectral Width	37878.8
12	Lowest Frequency	-6389.1
13	Nucleus	13C
14	Acquired Size	33280
15	Spectral Size	131072

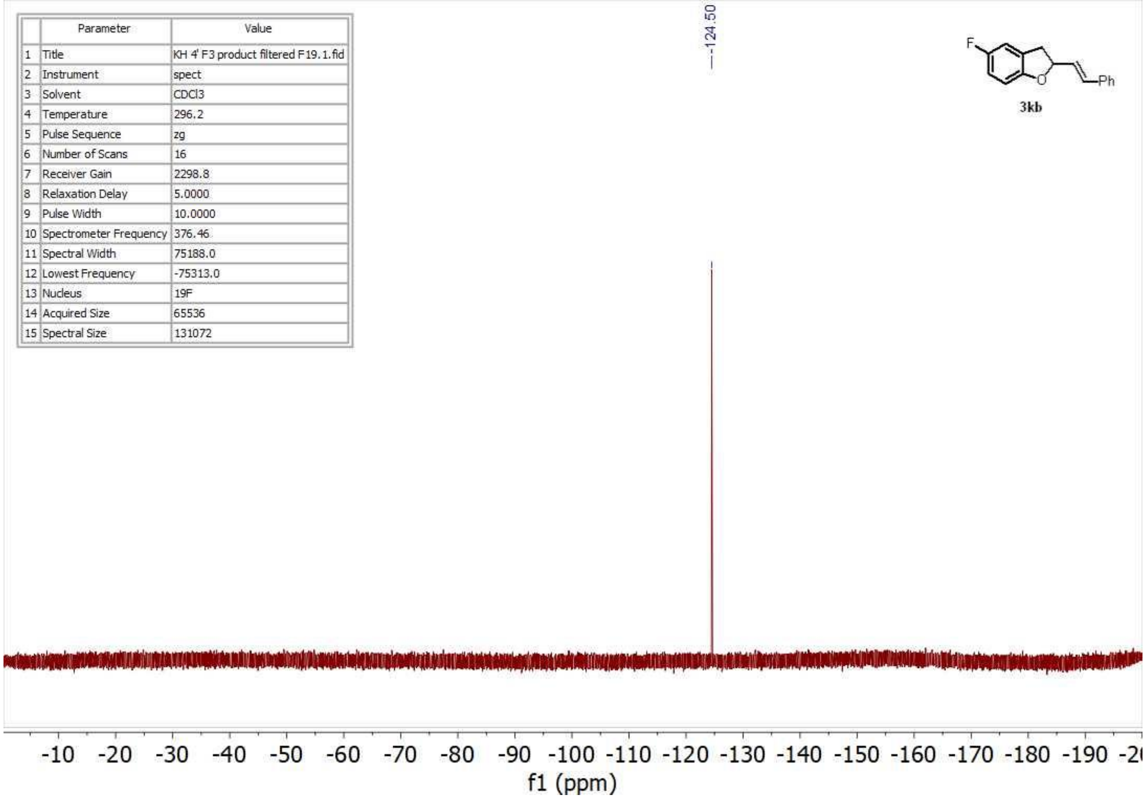

	Parameter	Value
1	Title	KH 4' F3 product filtered F19.1.fid
2	Instrument	spect
3	Solvent	CDCl3
4	Temperature	296.2
5	Pulse Sequence	zg
6	Number of Scans	16
7	Receiver Gain	2298.8
8	Relaxation Delay	5.0000
9	Pulse Width	10.0000
10	Spectrometer Frequency	376.46
11	Spectral Width	75188.0
12	Lowest Frequency	-75313.0
13	Nucleus	19F
14	Acquired Size	65536
15	Spectral Size	131072

(*E*)-1-(2-styryl-2,3-dihydrobenzofuran-5-yl)piperidine (3lb)

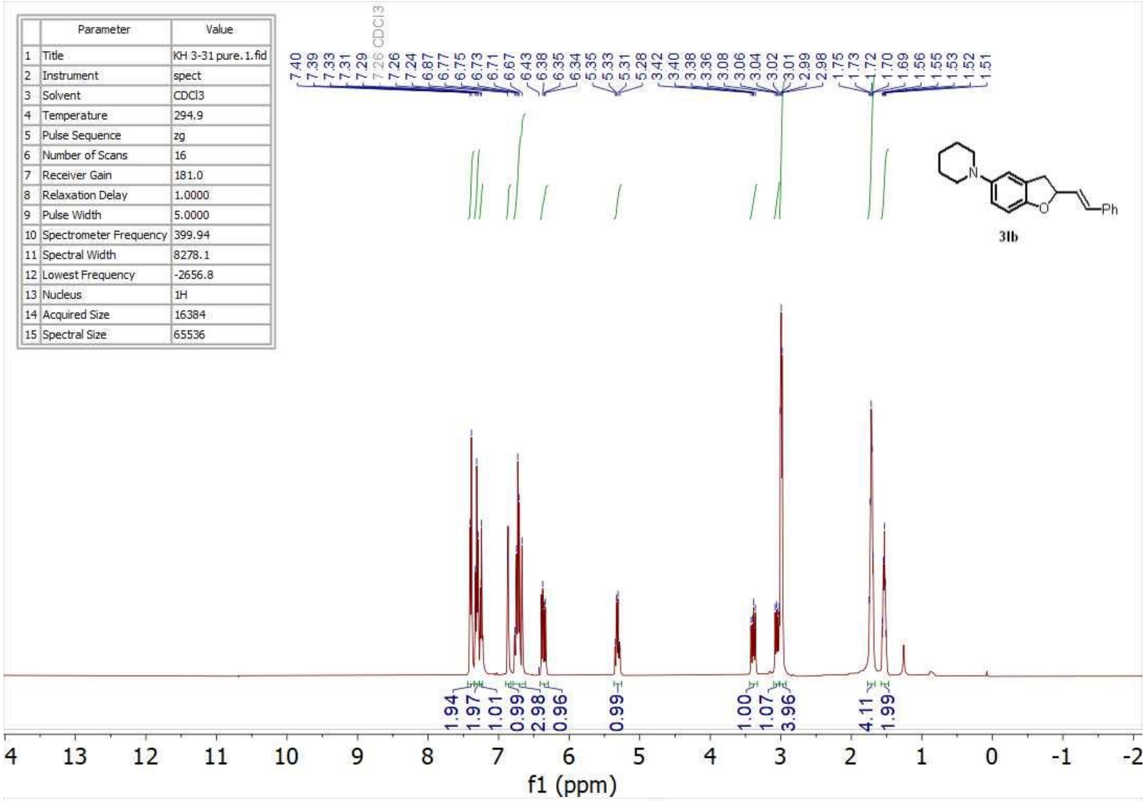

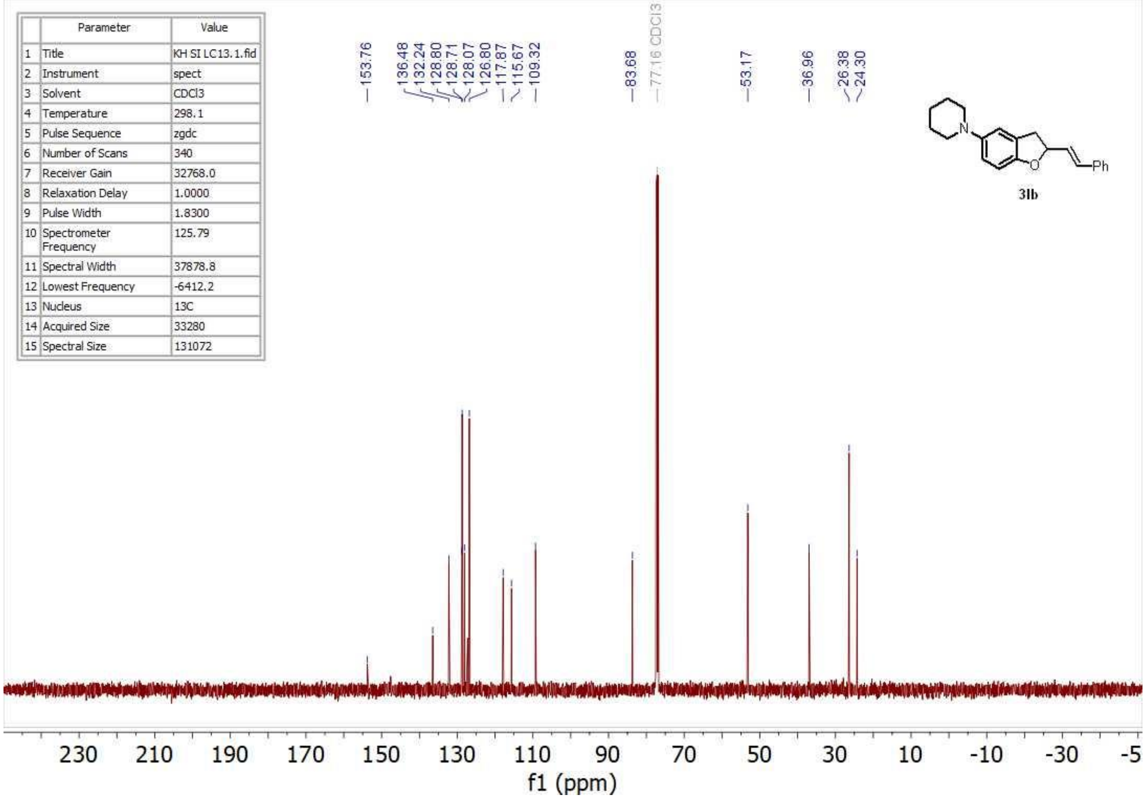

Isopentyl (*E*)-2-styryl-2,3-dihydrobenzofuran-6-carboxylate (3mb)

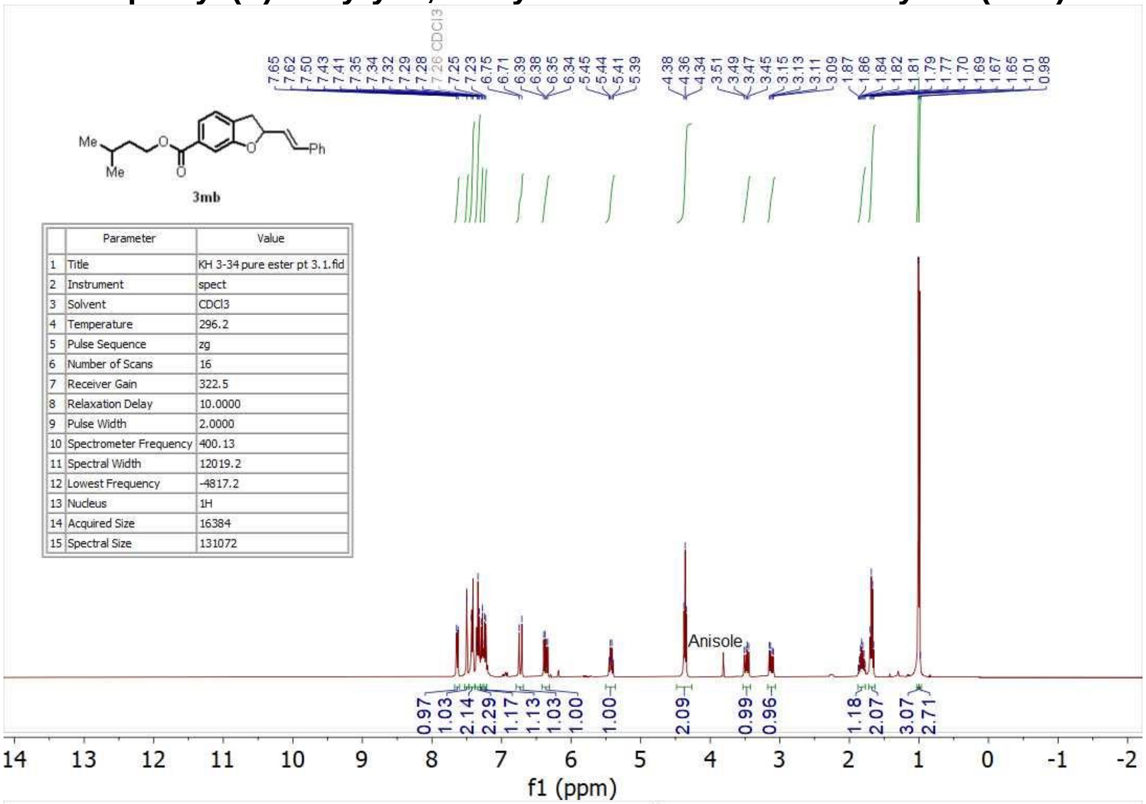

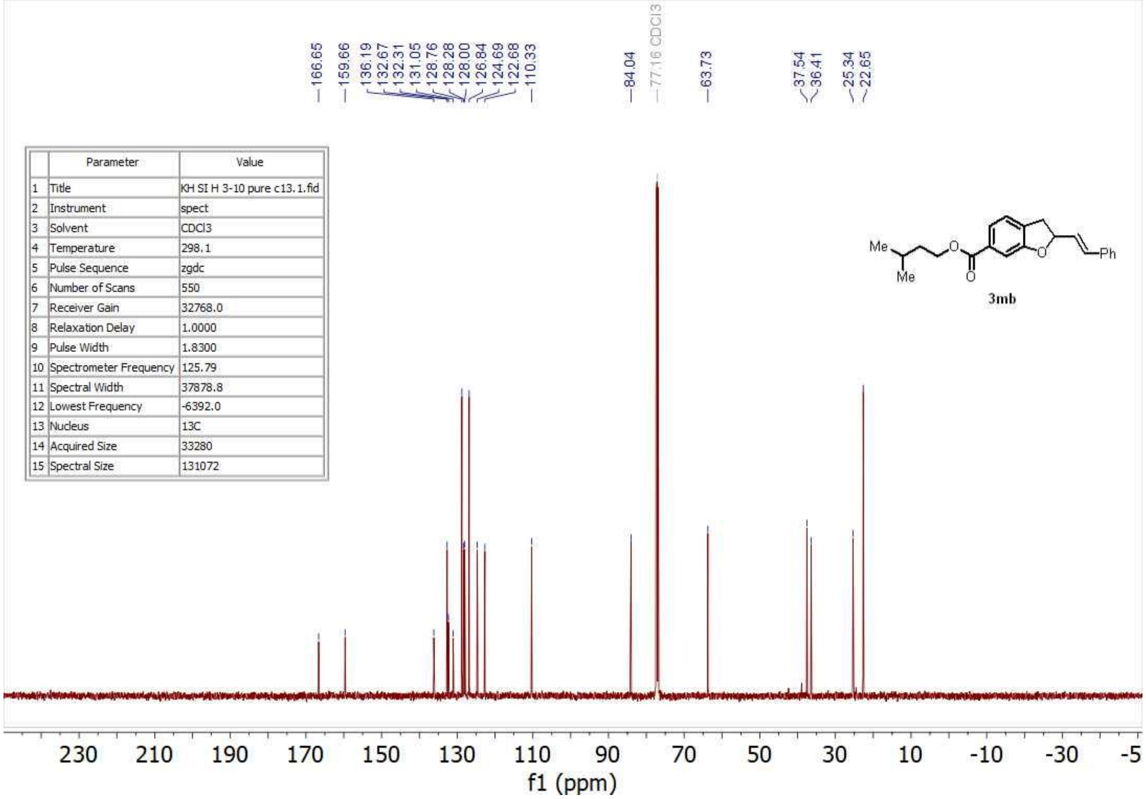

(*E*)-5-methyl-2-styryl-2,3-dihydrofuro[3,2-*b*]pyridine (3nb)

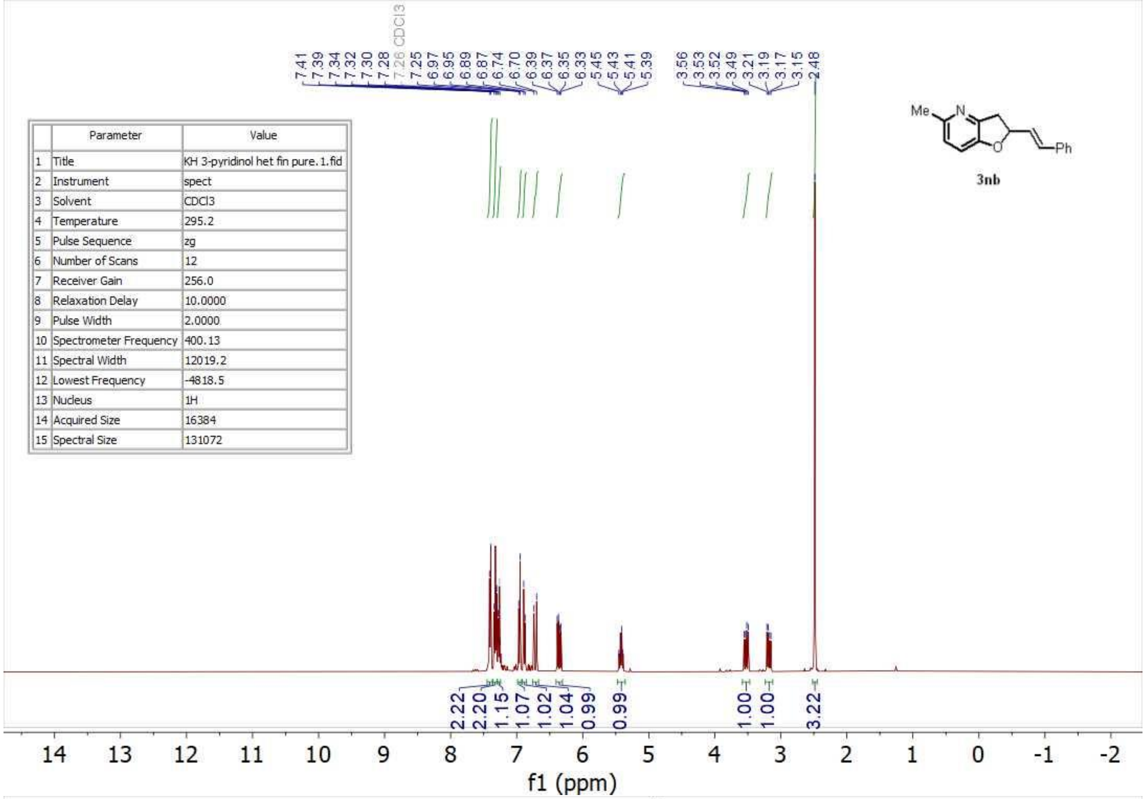

	Parameter	Value
1	Title	KH 3-pyridinol het fin pure.1.fid
2	Instrument	spect
3	Solvent	CDCl3
4	Temperature	295.2
5	Pulse Sequence	zg
6	Number of Scans	12
7	Receiver Gain	256.0
8	Relaxation Delay	10.0000
9	Pulse Width	2.0000
10	Spectrometer Frequency	400.13
11	Spectral Width	12019.2
12	Lowest Frequency	-4818.5
13	Nucleus	1H
14	Acquired Size	16384
15	Spectral Size	131072

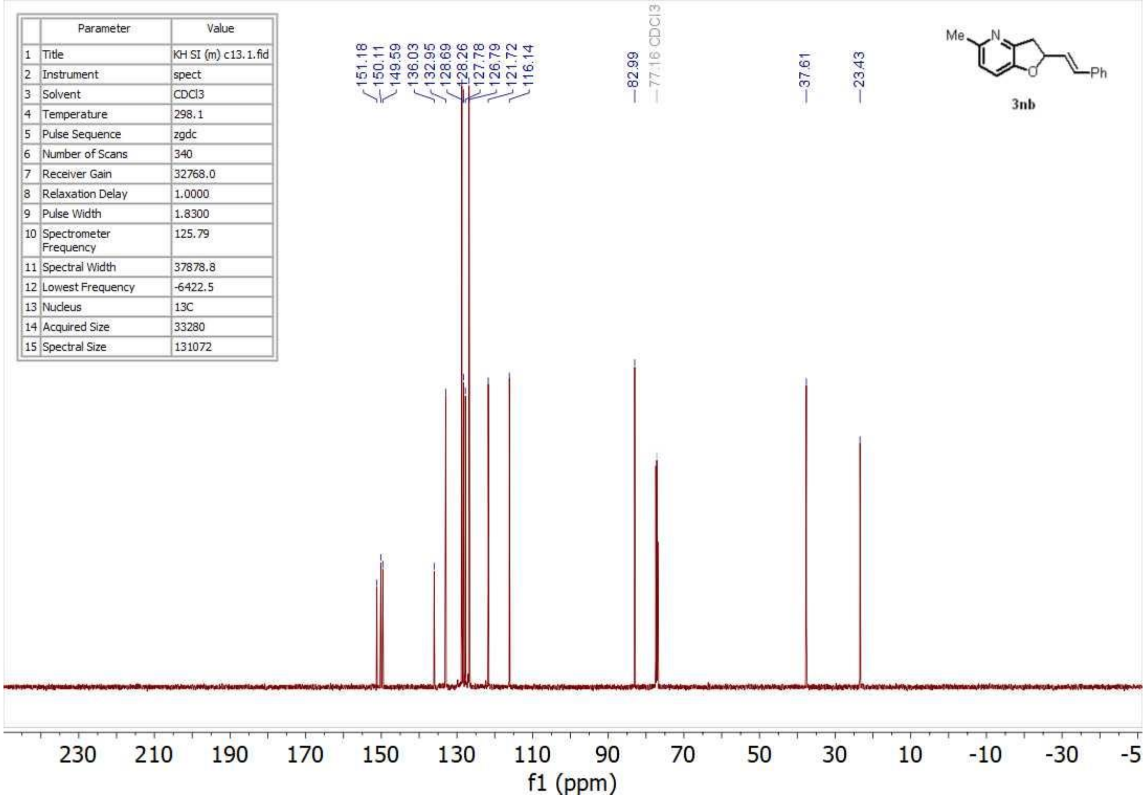

	Parameter	Value
1	Title	KH SI (m) c13.1.fid
2	Instrument	spect
3	Solvent	CDCl3
4	Temperature	298.1
5	Pulse Sequence	zgdc
6	Number of Scans	340
7	Receiver Gain	32768.0
8	Relaxation Delay	1.0000
9	Pulse Width	1.8300
10	Spectrometer Frequency	125.79
11	Spectral Width	37878.8
12	Lowest Frequency	-6422.5
13	Nucleus	13C
14	Acquired Size	33280
15	Spectral Size	131072

(*E*)-2-(4-methoxystyryl)-2,3-dihydrobenzofuran (3ac)

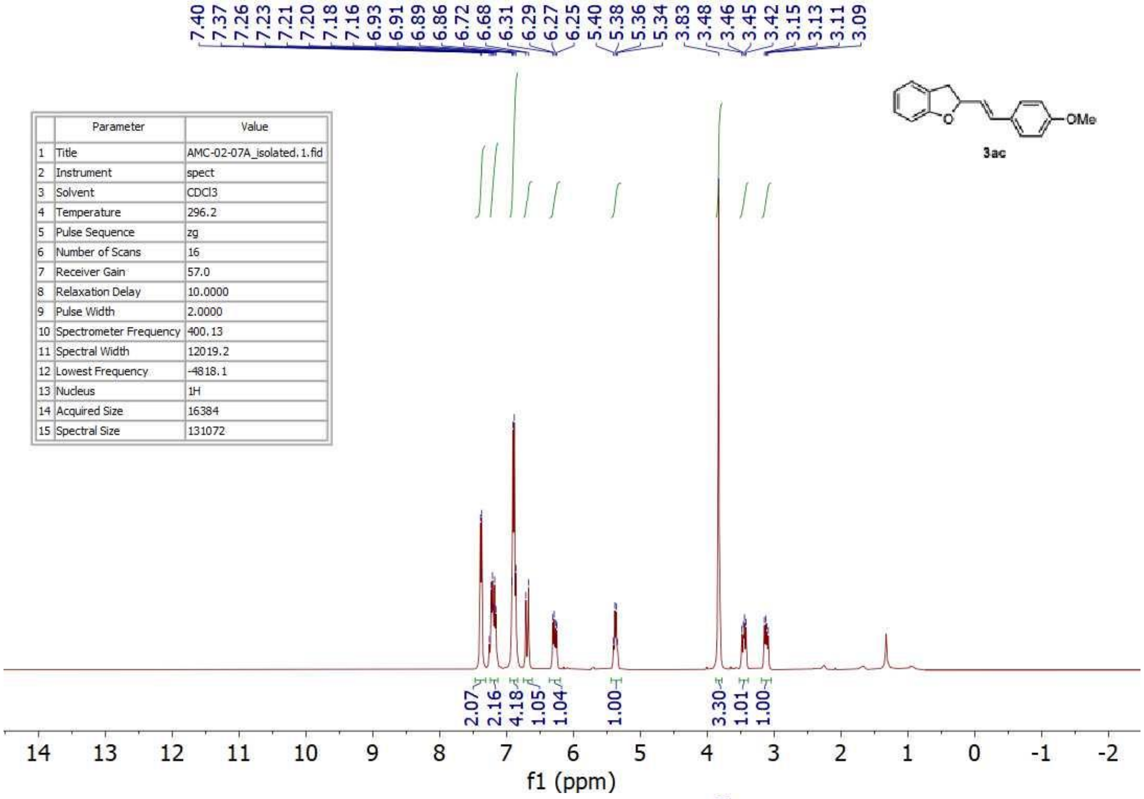

	Parameter	Value
1	Title	AMC-02-07A_isolated.1.fid
2	Instrument	spect
3	Solvent	CDCl3
4	Temperature	296.2
5	Pulse Sequence	zg
6	Number of Scans	16
7	Receiver Gain	57.0
8	Relaxation Delay	10.0000
9	Pulse Width	2.0000
10	Spectrometer Frequency	400.13
11	Spectral Width	12019.2
12	Lowest Frequency	-4818.1
13	Nucleus	1H
14	Acquired Size	16384
15	Spectral Size	131072

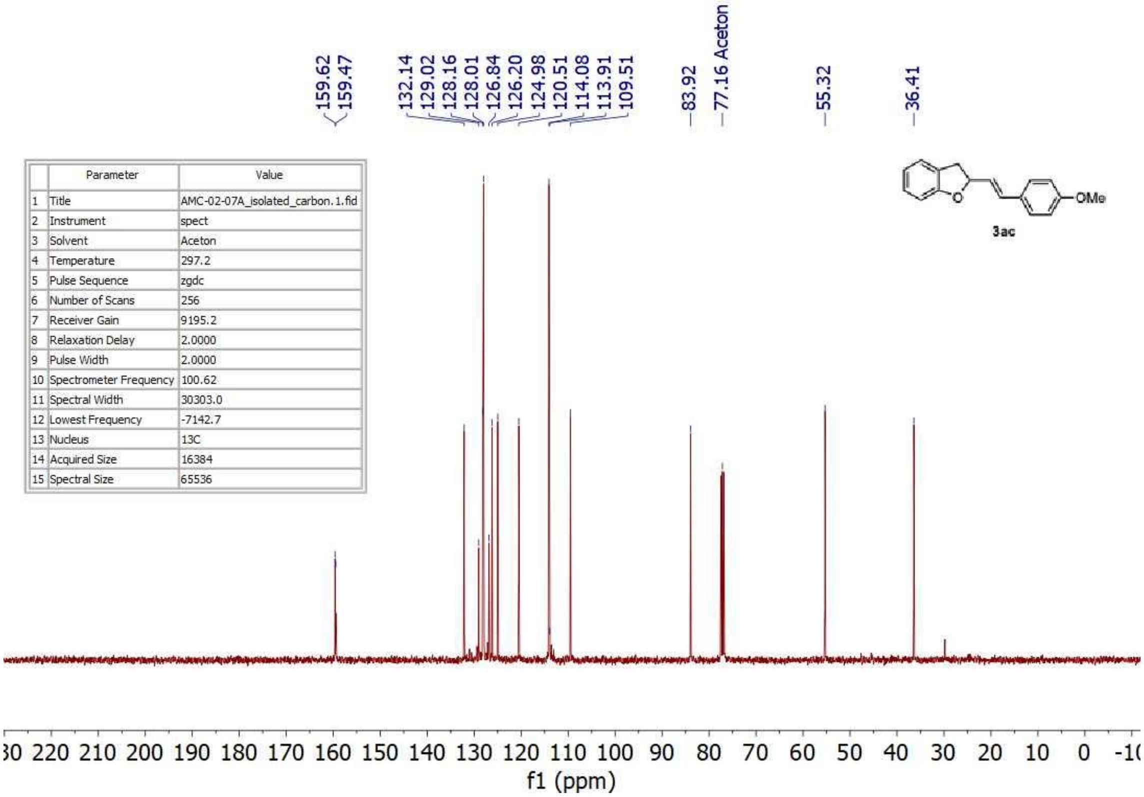

	Parameter	Value
1	Title	AMC-02-07A_isolated_carbon.1.fid
2	Instrument	spect
3	Solvent	Aceton
4	Temperature	297.2
5	Pulse Sequence	zgdc
6	Number of Scans	256
7	Receiver Gain	9195.2
8	Relaxation Delay	2.0000
9	Pulse Width	2.0000
10	Spectrometer Frequency	100.62
11	Spectral Width	30303.0
12	Lowest Frequency	-7142.7
13	Nucleus	13C
14	Acquired Size	16384
15	Spectral Size	65536

(*E*)-2-(4-(trifluoromethyl)styryl)-2,3-dihydrobenzofuran (3ad)

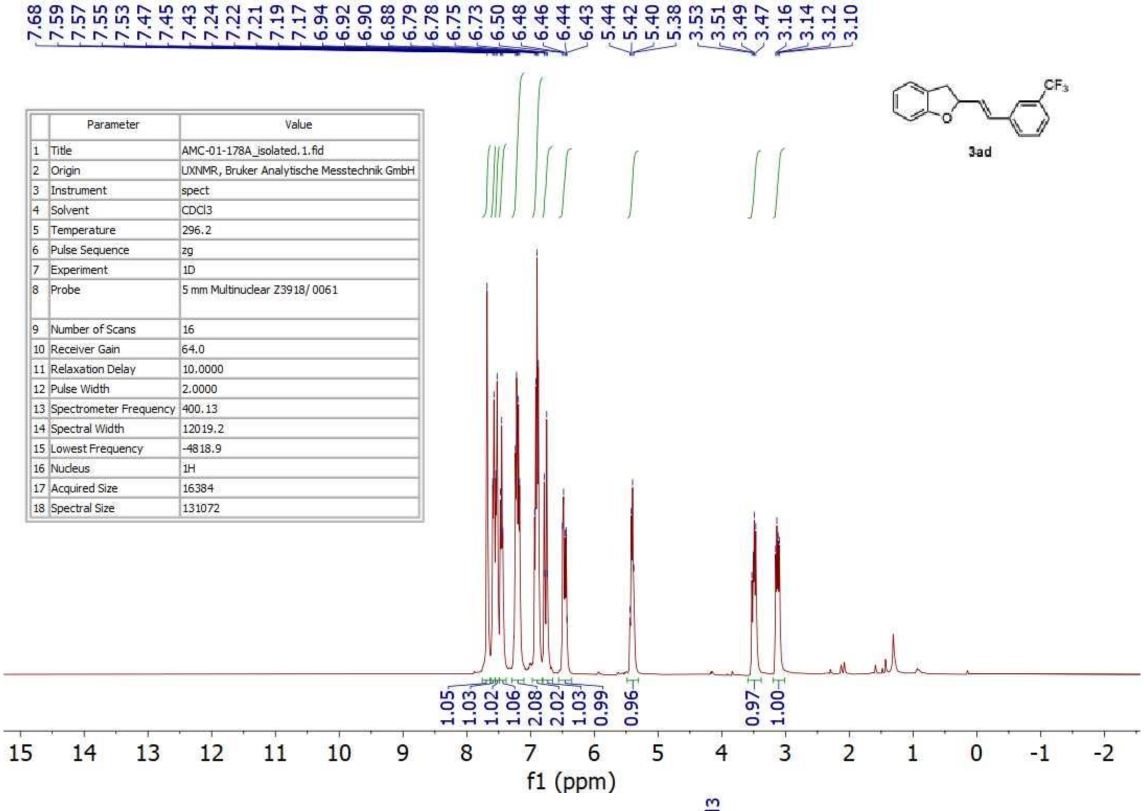

	Parameter	Value
1	Title	AMC-01-178A_isolated.1.fid
2	Origin	UXNMR, Bruker Analytische Messtechnik GmbH
3	Instrument	spect
4	Solvent	CDCl3
5	Temperature	296.2
6	Pulse Sequence	zg
7	Experiment	1D
8	Probe	5 mm Multinuclear Z3918/ 0061
9	Number of Scans	16
10	Receiver Gain	64.0
11	Relaxation Delay	10.0000
12	Pulse Width	2.0000
13	Spectrometer Frequency	400.13
14	Spectral Width	12019.2
15	Lowest Frequency	-4818.9
16	Nucleus	1H
17	Acquired Size	16384
18	Spectral Size	131072

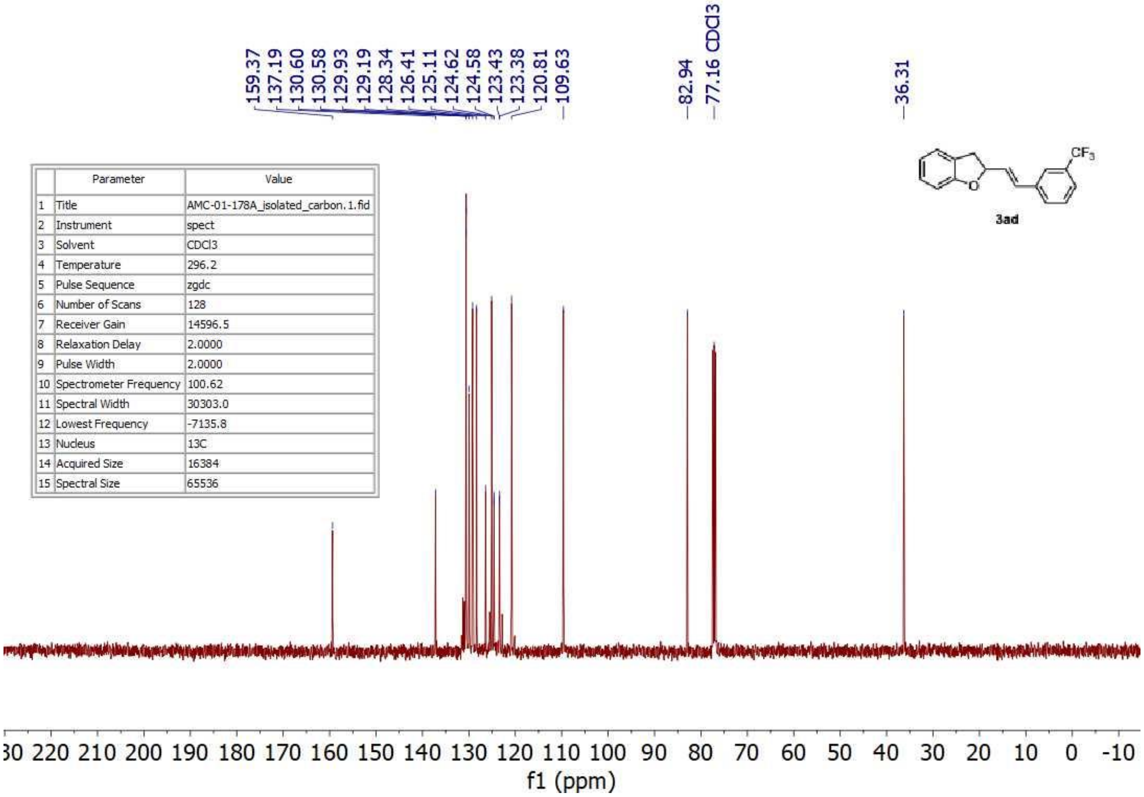

	Parameter	Value
1	Title	AMC-01-178A_isolated_carbon.1.fid
2	Instrument	spect
3	Solvent	CDCl3
4	Temperature	296.2
5	Pulse Sequence	zgdc
6	Number of Scans	128
7	Receiver Gain	14596.5
8	Relaxation Delay	2.0000
9	Pulse Width	2.0000
10	Spectrometer Frequency	100.62
11	Spectral Width	30303.0
12	Lowest Frequency	-7135.8
13	Nucleus	13C
14	Acquired Size	16384
15	Spectral Size	65536

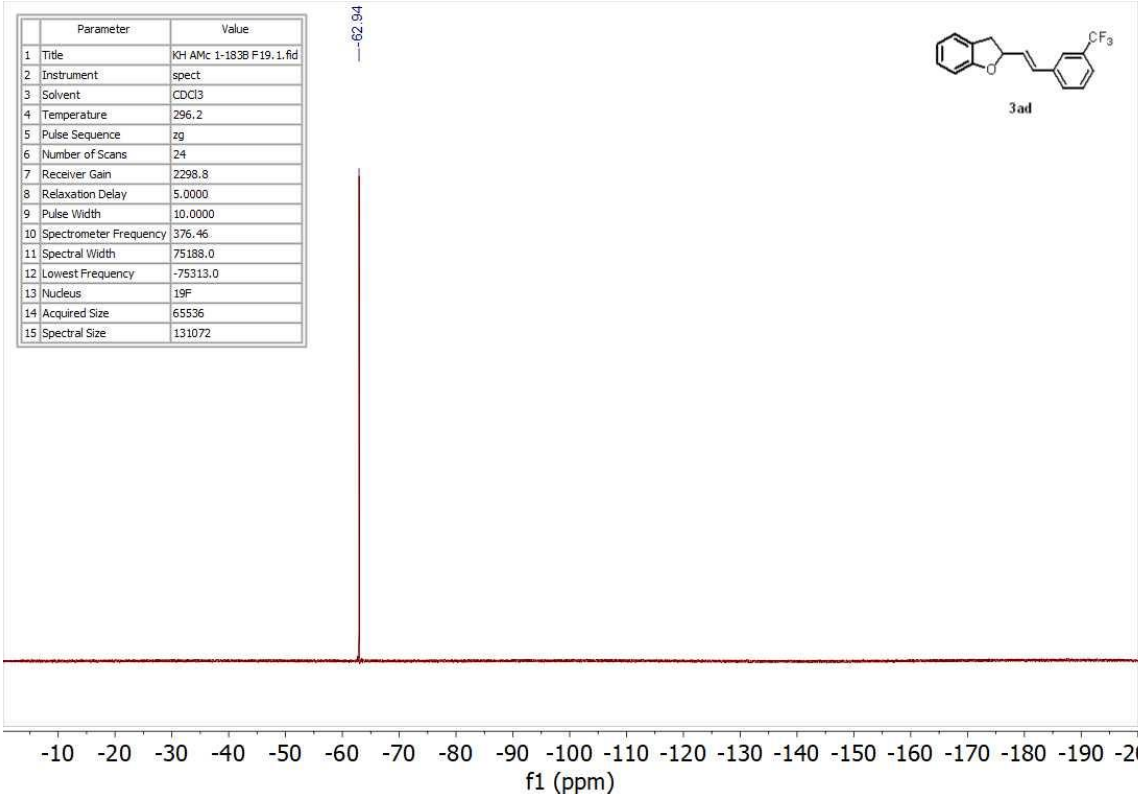

Parameter | Value
1 Title | KH AMc 1-183B F19.1.fid
2 Instrument | spect
3 Solvent | CDCl3
4 Temperature | 296.2
5 Pulse Sequence | zg
6 Number of Scans | 24
7 Receiver Gain | 2298.8
8 Relaxation Delay | 5.0000
9 Pulse Width | 10.0000
10 Spectrometer Frequency | 376.46
11 Spectral Width | 75188.0
12 Lowest Frequency | -75313.0
13 Nucleus | 19F
14 Acquired Size | 65536
15 Spectral Size | 131072
-62.94
CF3
O
3ad
-10 -20 -30 -40 -50 -60 -70 -80 -90 -100 -110 -120 -130 -140 -150 -160 -170 -180 -190
f1 (ppm)

(*E*)-4-(2,3-dihydrobenzofuran-2-yl)but-3-en-1-ol (3ae)

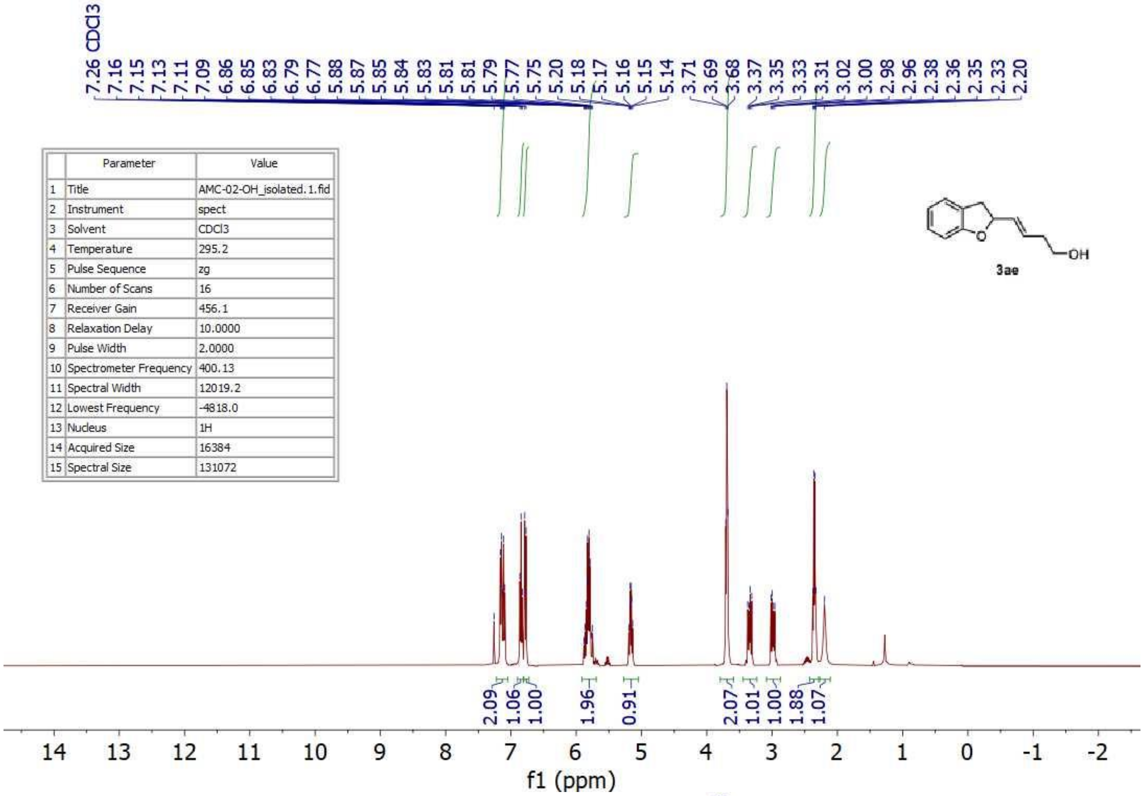

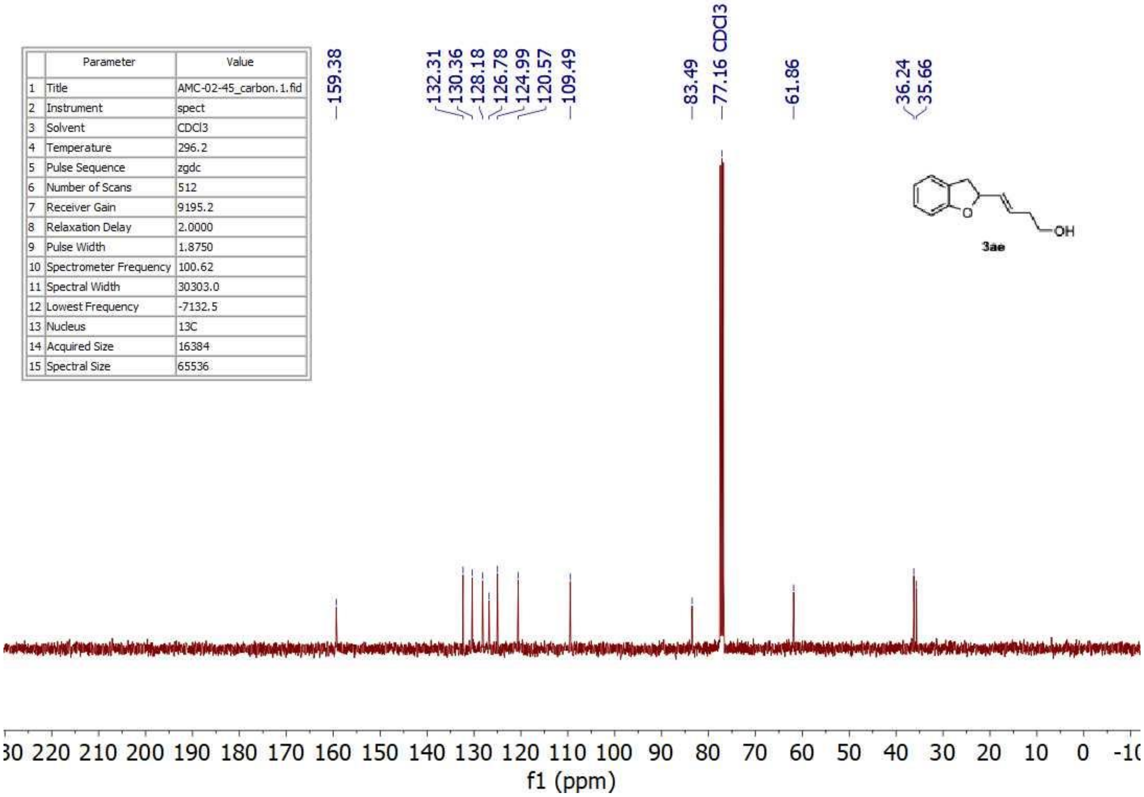

(*E*)-4-(2,3-dihydrobenzofuran-2-yl)but-3-en-1-yl benzoate (3af)

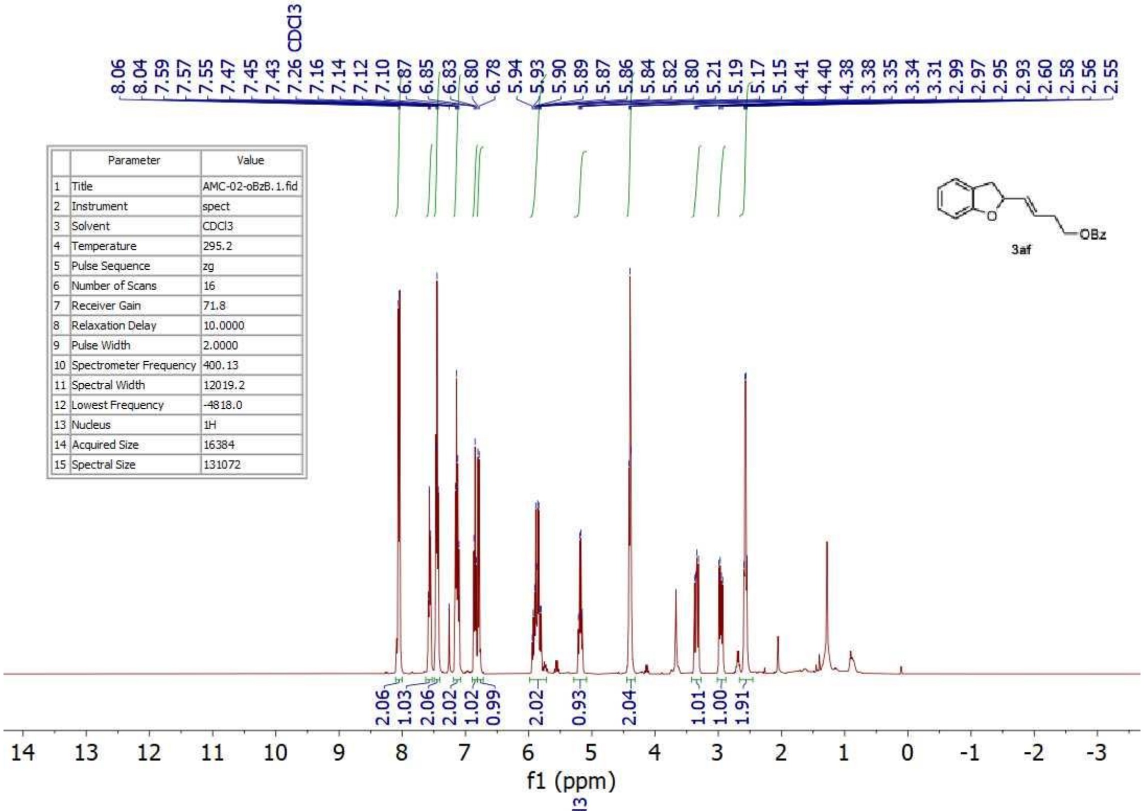

	Parameter	Value
1	Title	AMC-02-oBzB.1.fid
2	Instrument	spect
3	Solvent	CDCl3
4	Temperature	295.2
5	Pulse Sequence	zg
6	Number of Scans	16
7	Receiver Gain	71.8
8	Relaxation Delay	10.0000
9	Pulse Width	2.0000
10	Spectrometer Frequency	400.13
11	Spectral Width	12019.2
12	Lowest Frequency	-4818.0
13	Nucleus	1H
14	Acquired Size	16384
15	Spectral Size	131072

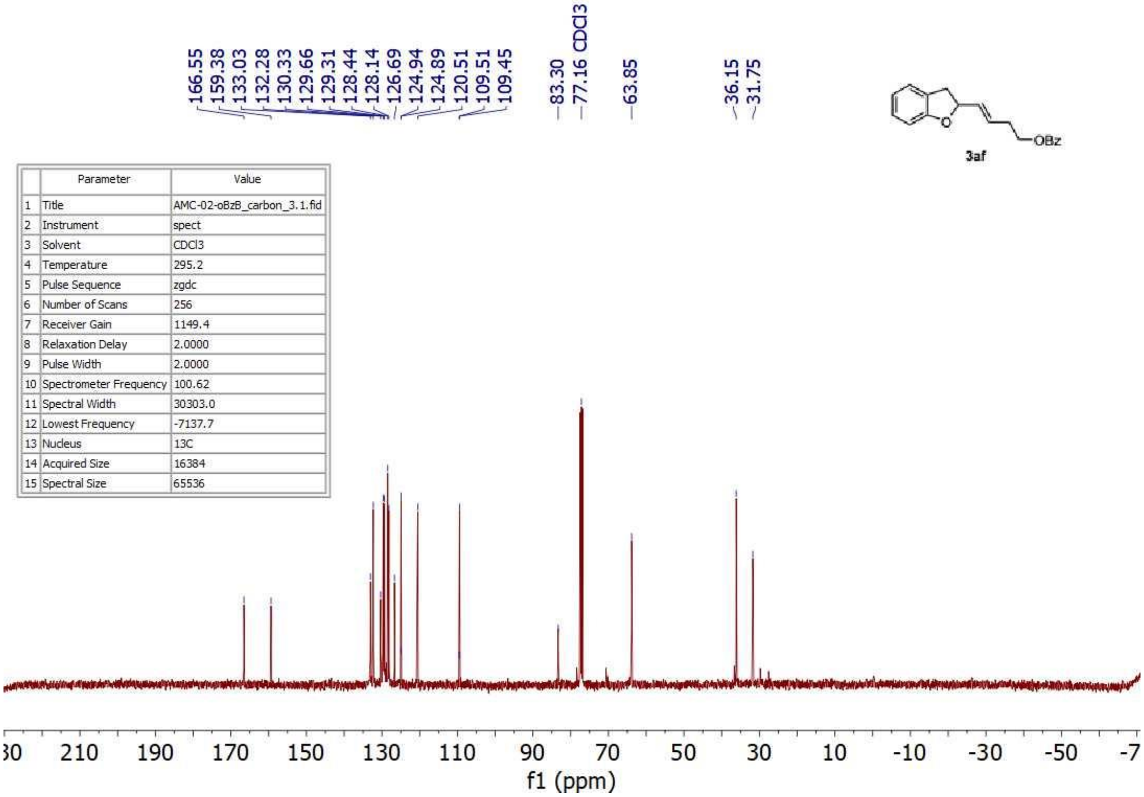

	Parameter	Value
1	Title	AMC-02-oBzB_carbon_3.1.fid
2	Instrument	spect
3	Solvent	CDCl3
4	Temperature	295.2
5	Pulse Sequence	zgdc
6	Number of Scans	256
7	Receiver Gain	1149.4
8	Relaxation Delay	2.0000
9	Pulse Width	2.0000
10	Spectrometer Frequency	100.62
11	Spectral Width	30303.0
12	Lowest Frequency	-7137.7
13	Nucleus	13C
14	Acquired Size	16384
15	Spectral Size	65536

(*E*)-2-(2-(thiophen-2-yl)vinyl)-2,3-dihydrobenzofuran (3ag)

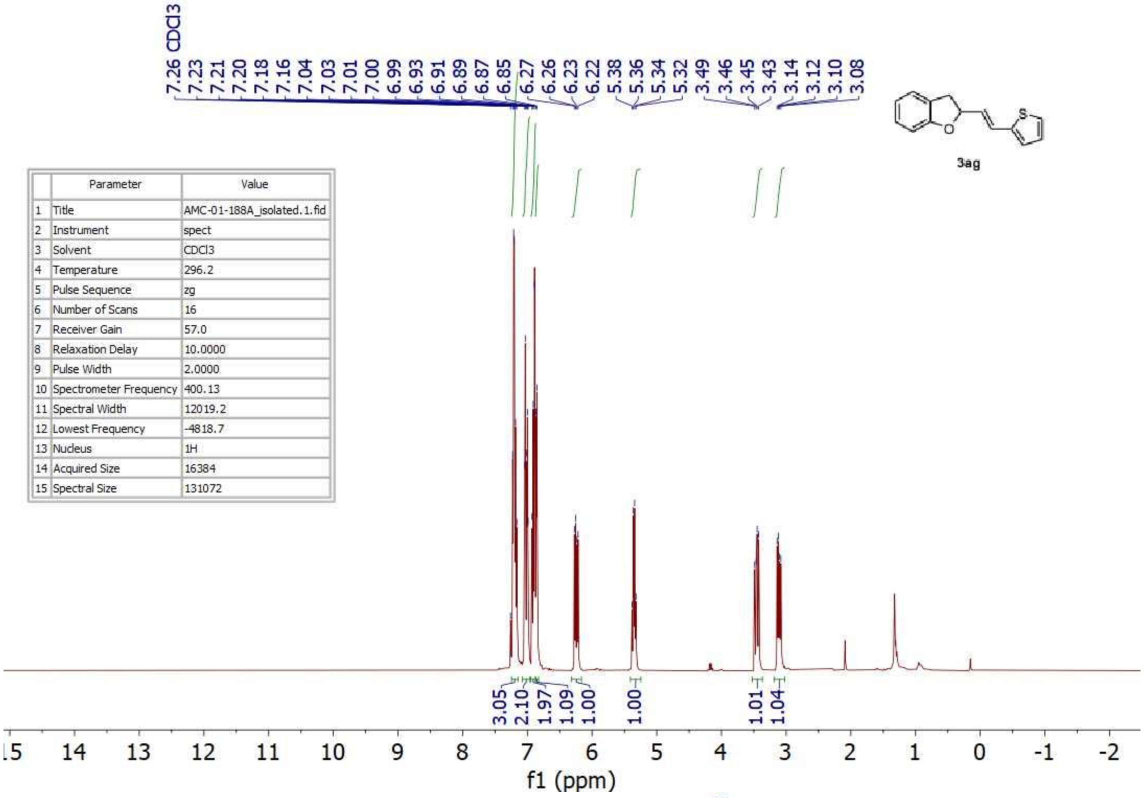

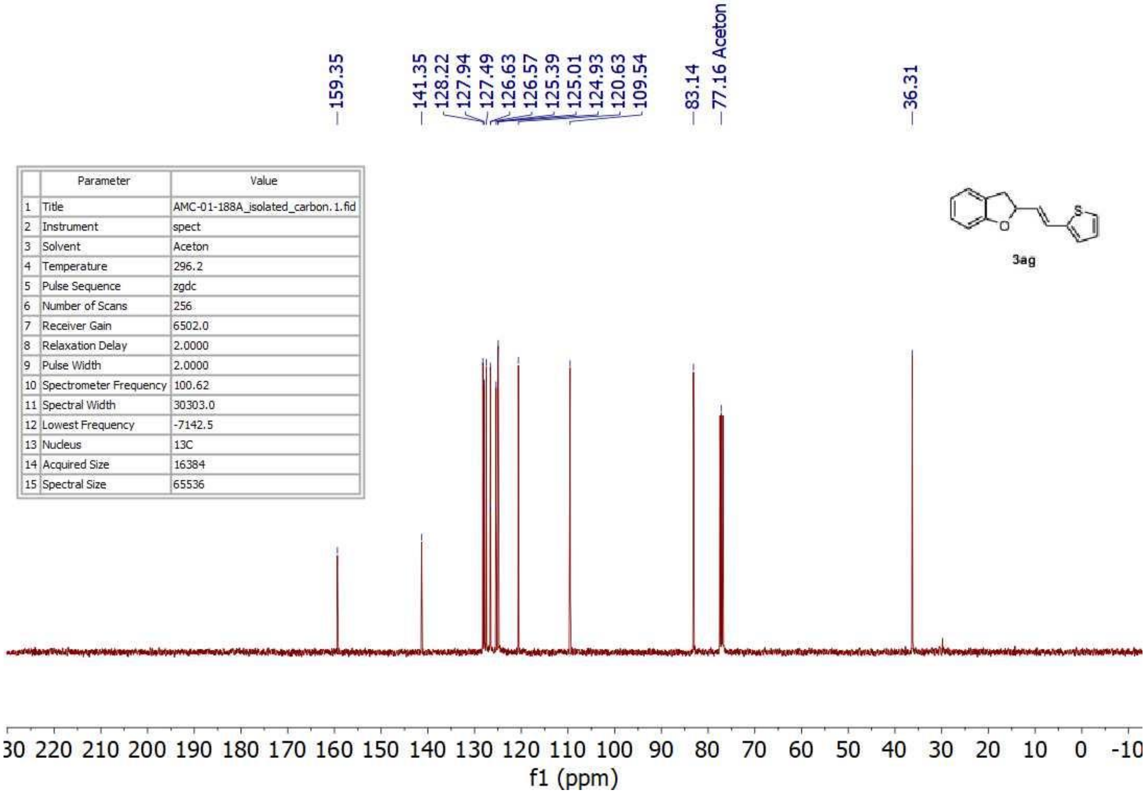

(*E*)-2-(2-(5-methylfuran-2-yl)vinyl)-2,3-dihydrobenzofuran (3ah)

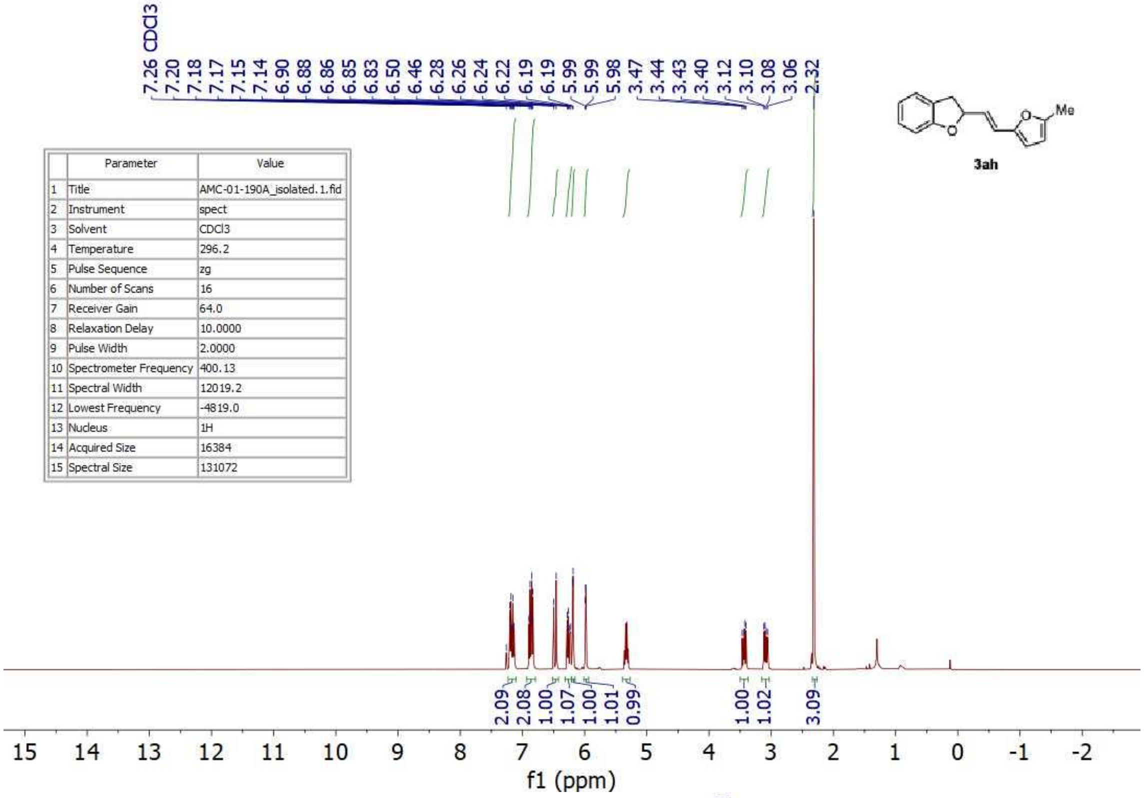

	Parameter	Value
1	Title	AMC-01-190A_isolated.1.fid
2	Instrument	spect
3	Solvent	CDCl3
4	Temperature	296.2
5	Pulse Sequence	zg
6	Number of Scans	16
7	Receiver Gain	64.0
8	Relaxation Delay	10.0000
9	Pulse Width	2.0000
10	Spectrometer Frequency	400.13
11	Spectral Width	12019.2
12	Lowest Frequency	-4819.0
13	Nucleus	1H
14	Acquired Size	16384
15	Spectral Size	131072

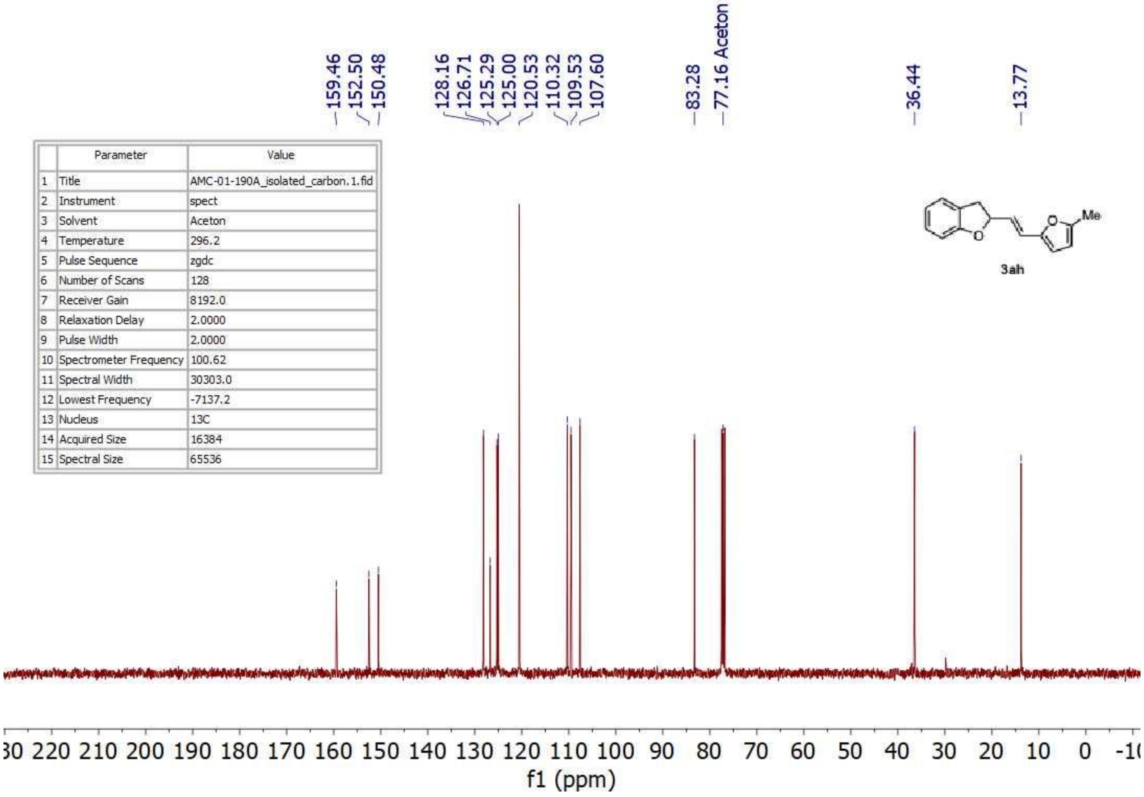

	Parameter	Value
1	Title	AMC-01-190A_isolated_carbon.1.fid
2	Instrument	spect
3	Solvent	Aceton
4	Temperature	296.2
5	Pulse Sequence	zgdc
6	Number of Scans	128
7	Receiver Gain	8192.0
8	Relaxation Delay	2.0000
9	Pulse Width	2.0000
10	Spectrometer Frequency	100.62
11	Spectral Width	30303.0
12	Lowest Frequency	-7137.2
13	Nucleus	13C
14	Acquired Size	16384
15	Spectral Size	65536

(*E*)-2-(4-(2,3-dihydrobenzofuran-2-yl)but-3-en-1-yl)isoindoline-1,3-dione (3ai)

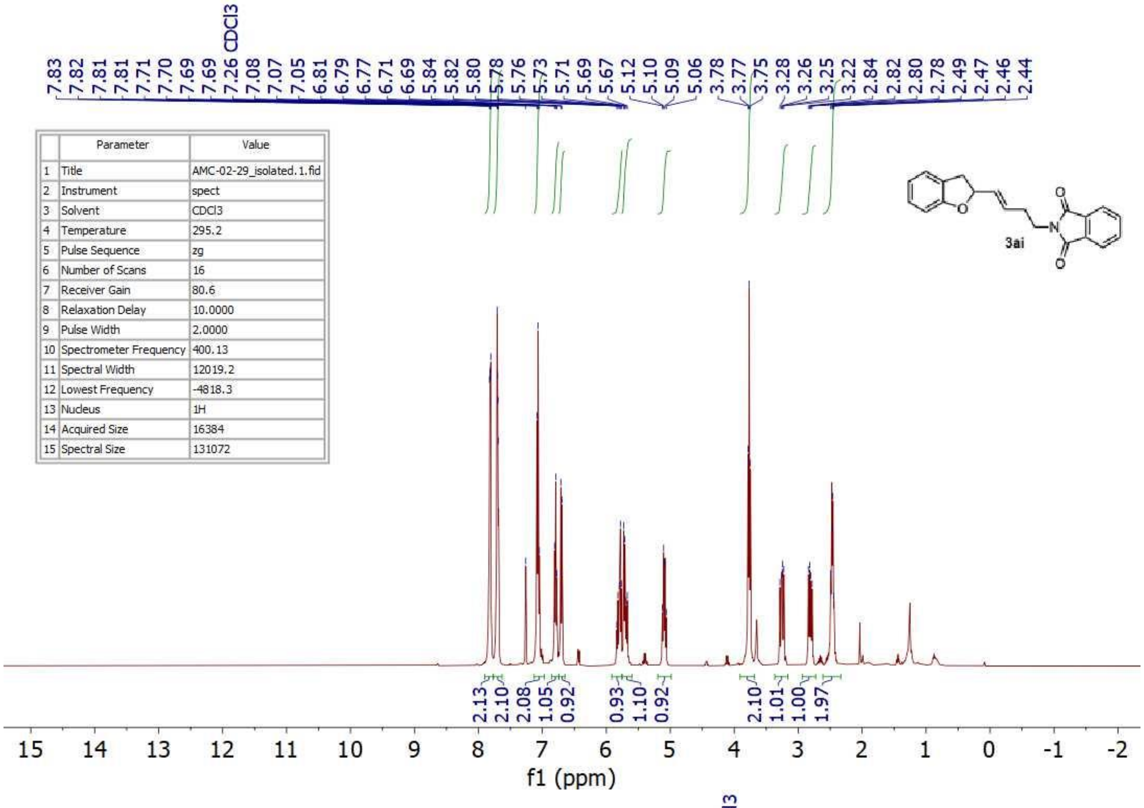

	Parameter	Value
1	Title	AMC-02-29_isolated.1.fid
2	Instrument	spect
3	Solvent	CDCl3
4	Temperature	295.2
5	Pulse Sequence	zg
6	Number of Scans	16
7	Receiver Gain	80.6
8	Relaxation Delay	10.0000
9	Pulse Width	2.0000
10	Spectrometer Frequency	400.13
11	Spectral Width	12019.2
12	Lowest Frequency	-4818.3
13	Nucleus	1H
14	Acquired Size	16384
15	Spectral Size	131072

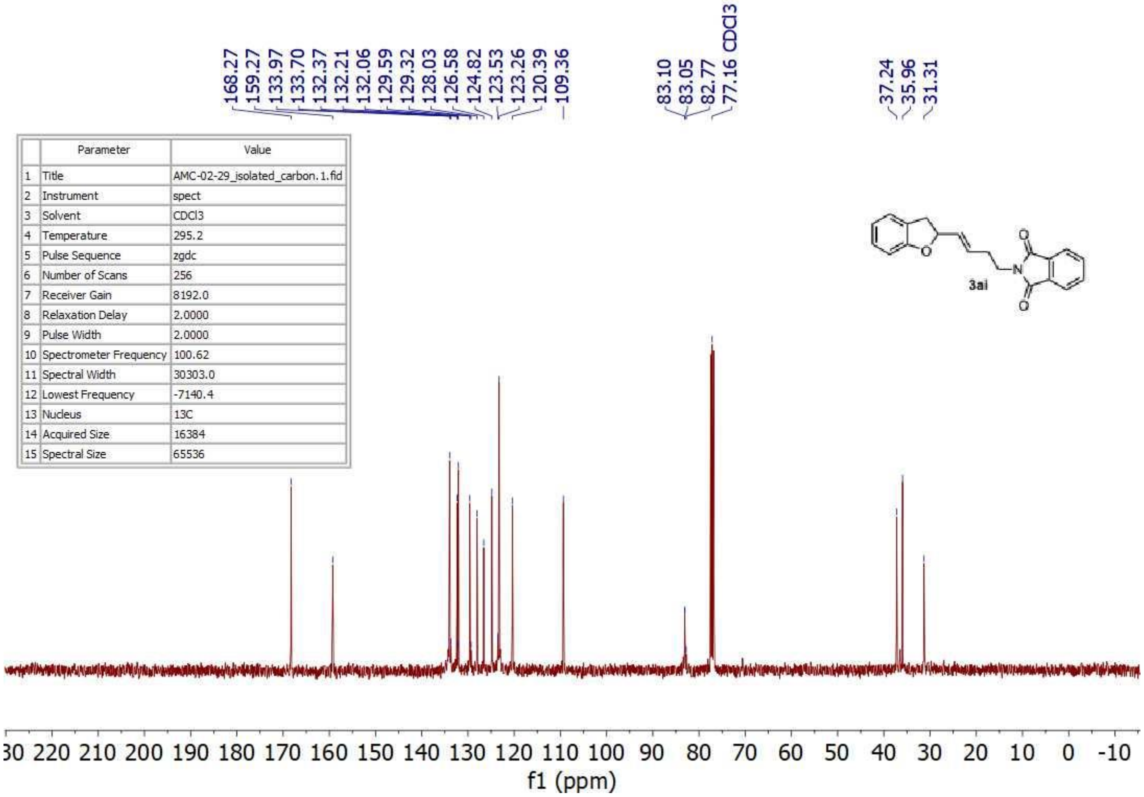

	Parameter	Value
1	Title	AMC-02-29_isolated_carbon.1.fid
2	Instrument	spect
3	Solvent	CDCl3
4	Temperature	295.2
5	Pulse Sequence	zgdc
6	Number of Scans	256
7	Receiver Gain	8192.0
8	Relaxation Delay	2.0000
9	Pulse Width	2.0000
10	Spectrometer Frequency	100.62
11	Spectral Width	30303.0
12	Lowest Frequency	-7140.4
13	Nucleus	13C
14	Acquired Size	16384
15	Spectral Size	65536

2-(6-methylhepta-1,5-dien-2-yl)-2,3-dihydrobenzofuran (3aj)

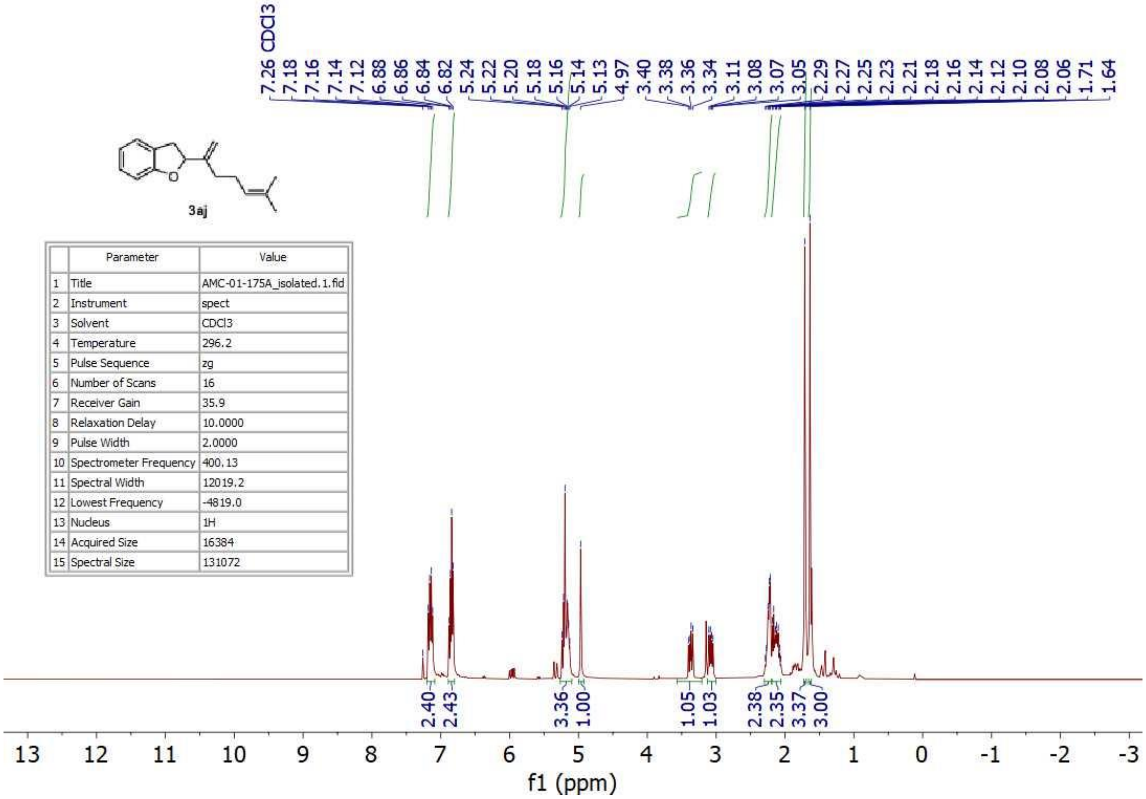

	Parameter	Value
1	Title	AMC-01-175A_isolated.1.fid
2	Instrument	spect
3	Solvent	CDCl3
4	Temperature	296.2
5	Pulse Sequence	zg
6	Number of Scans	16
7	Receiver Gain	35.9
8	Relaxation Delay	10.0000
9	Pulse Width	2.0000
10	Spectrometer Frequency	400.13
11	Spectral Width	12019.2
12	Lowest Frequency	-4819.0
13	Nucleus	1H
14	Acquired Size	16384
15	Spectral Size	131072

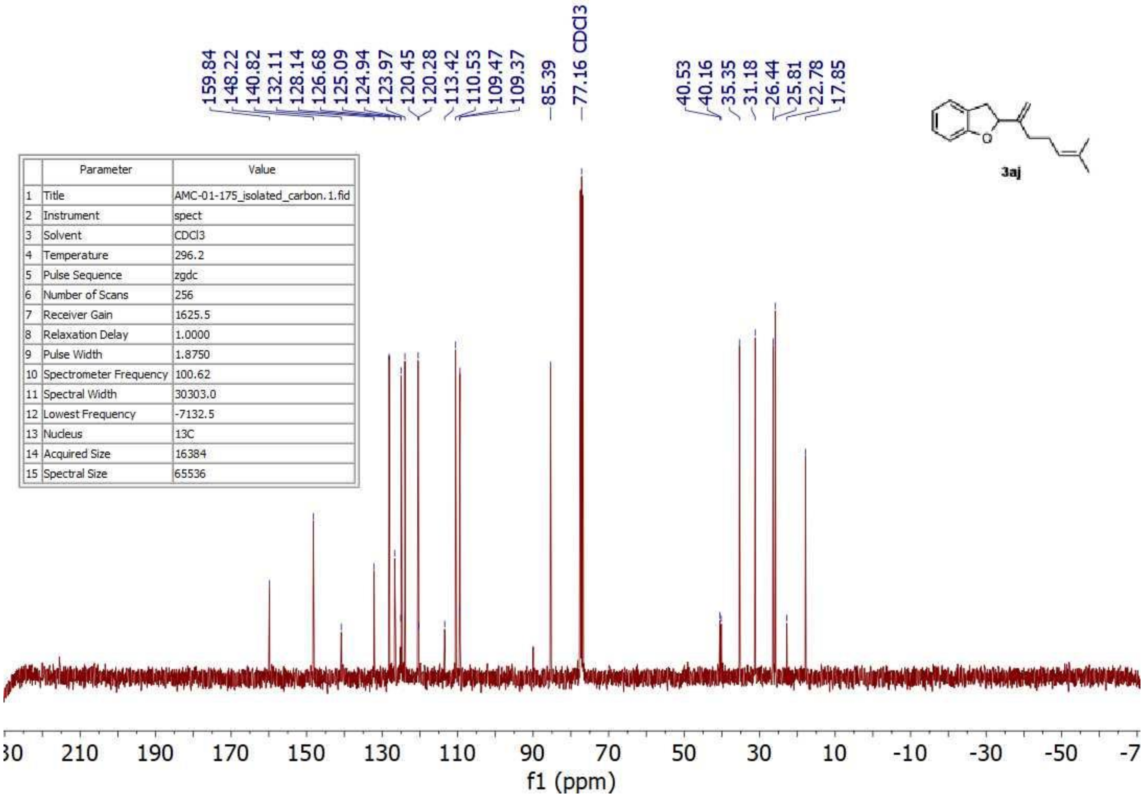

	Parameter	Value
1	Title	AMC-01-175_isolated_carbon.1.fid
2	Instrument	spect
3	Solvent	CDCl3
4	Temperature	296.2
5	Pulse Sequence	zgdc
6	Number of Scans	256
7	Receiver Gain	1625.5
8	Relaxation Delay	1.0000
9	Pulse Width	1.8750
10	Spectrometer Frequency	100.62
11	Spectral Width	30303.0
12	Lowest Frequency	-7132.5
13	Nucleus	13C
14	Acquired Size	16384
15	Spectral Size	65536

2-(4-(3,3-dimethyloxiran-2-yl)but-1-en-2-yl)-2,3-dihydrobenzofuran (3ak)

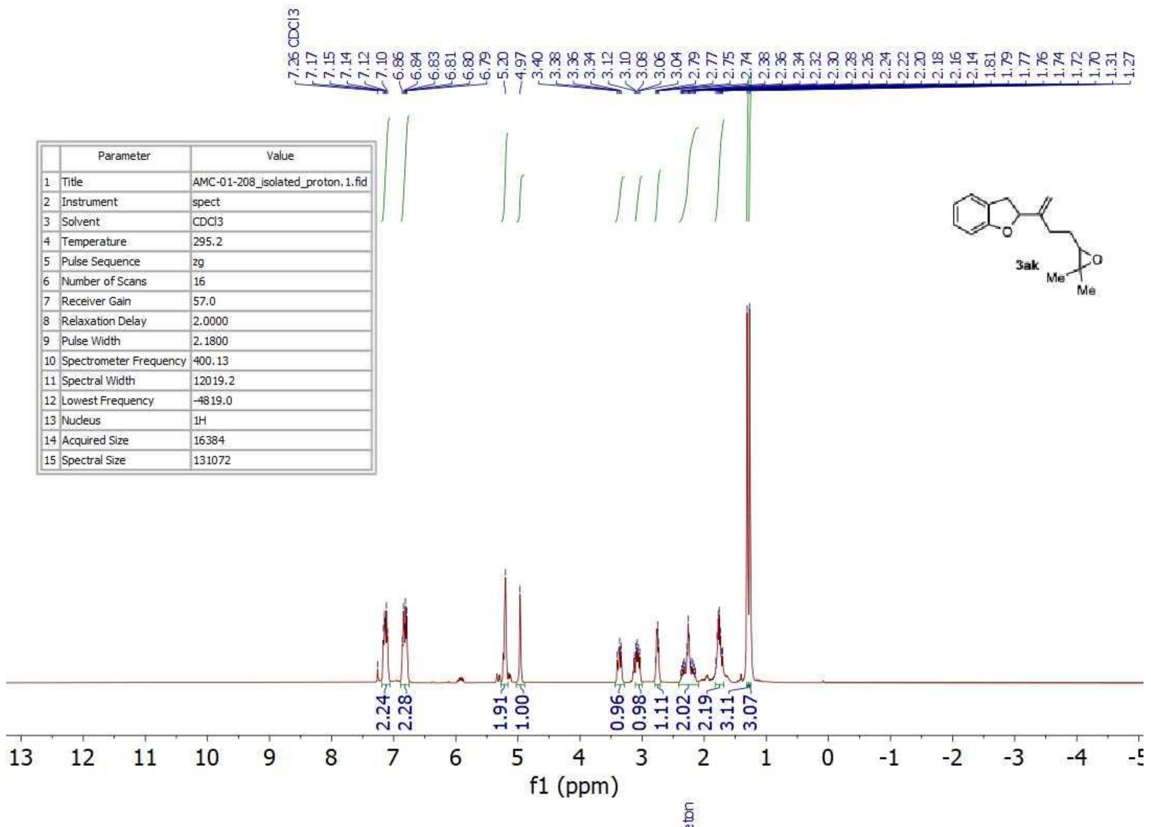

	Parameter	Value
1	Title	AMC-01-208_isolated_proton.1.fid
2	Instrument	spect
3	Solvent	CDCl3
4	Temperature	295.2
5	Pulse Sequence	zg
6	Number of Scans	16
7	Receiver Gain	57.0
8	Relaxation Delay	2.0000
9	Pulse Width	2.1800
10	Spectrometer Frequency	400.13
11	Spectral Width	12019.2
12	Lowest Frequency	-4819.0
13	Nucleus	1H
14	Acquired Size	16384
15	Spectral Size	131072

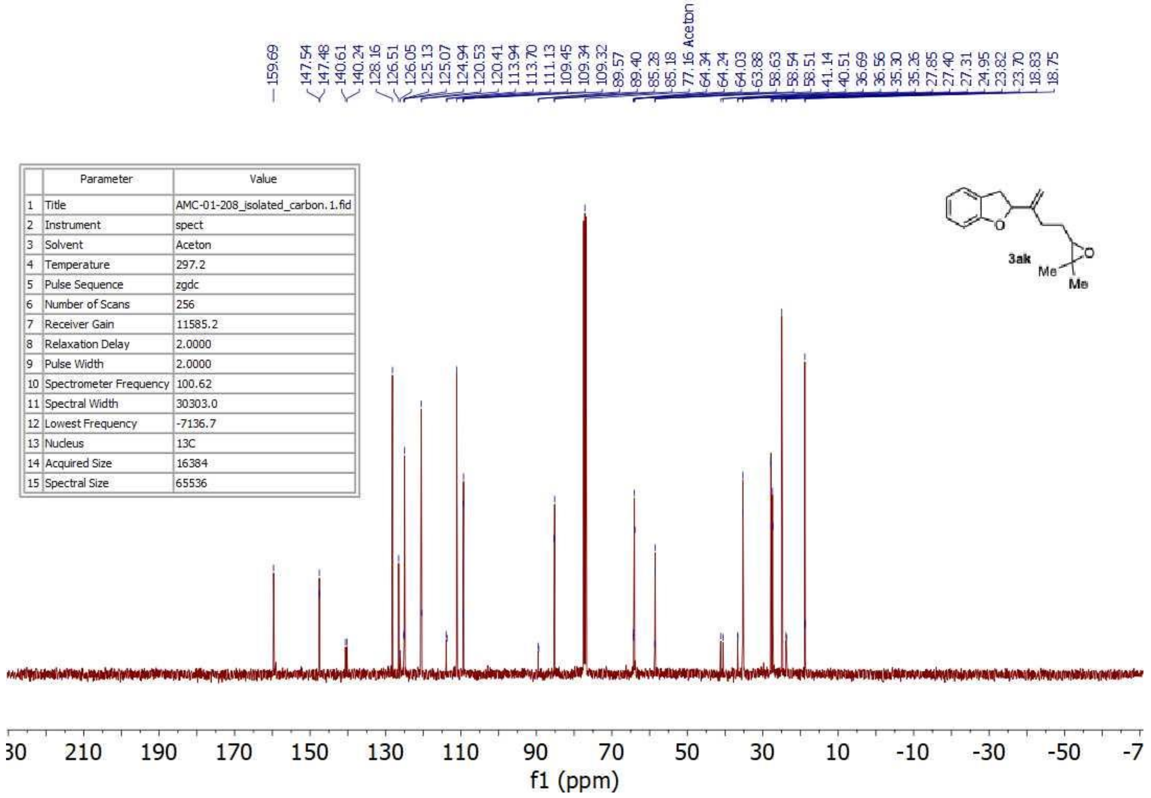

	Parameter	Value
1	Title	AMC-01-208_isolated_carbon.1.fid
2	Instrument	spect
3	Solvent	Aceton
4	Temperature	297.2
5	Pulse Sequence	zgdc
6	Number of Scans	256
7	Receiver Gain	11585.2
8	Relaxation Delay	2.0000
9	Pulse Width	2.0000
10	Spectrometer Frequency	100.62
11	Spectral Width	30303.0
12	Lowest Frequency	-7136.7
13	Nucleus	13C
14	Acquired Size	16384
15	Spectral Size	65536

6-(2,3-dihydrobenzofuran-2-yl)-2-methylhept-6-en-2-ol (3al)

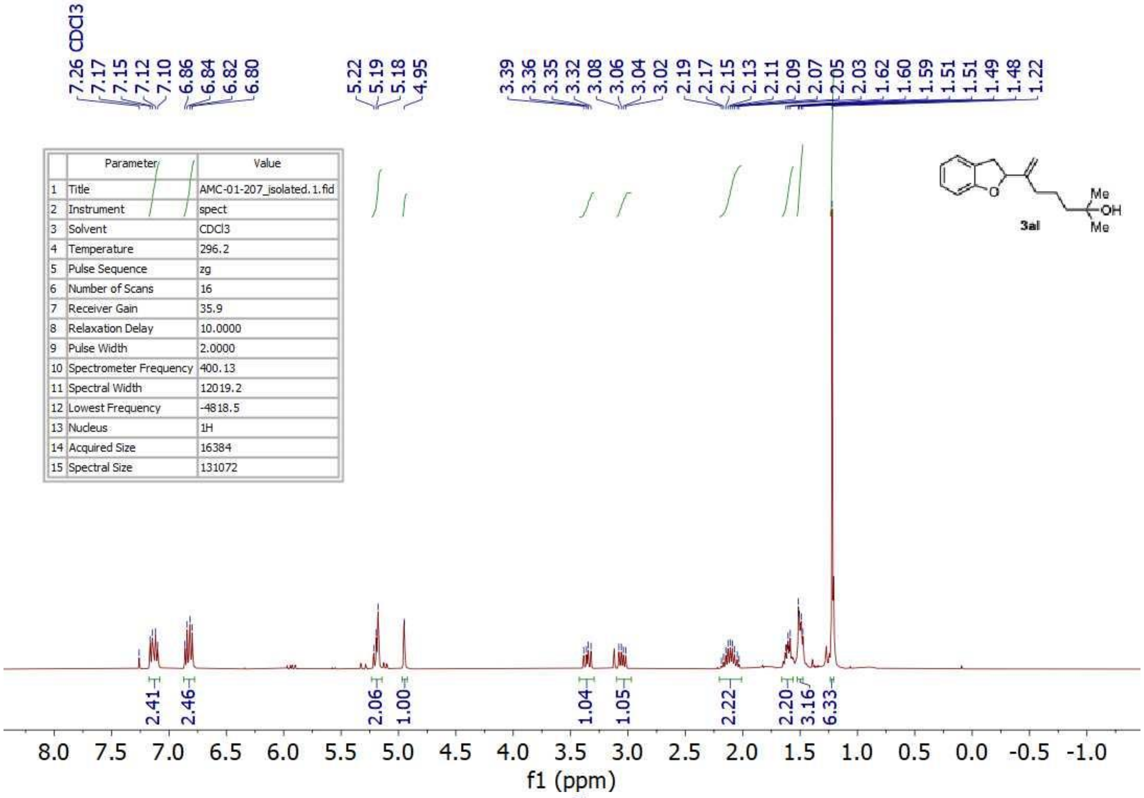

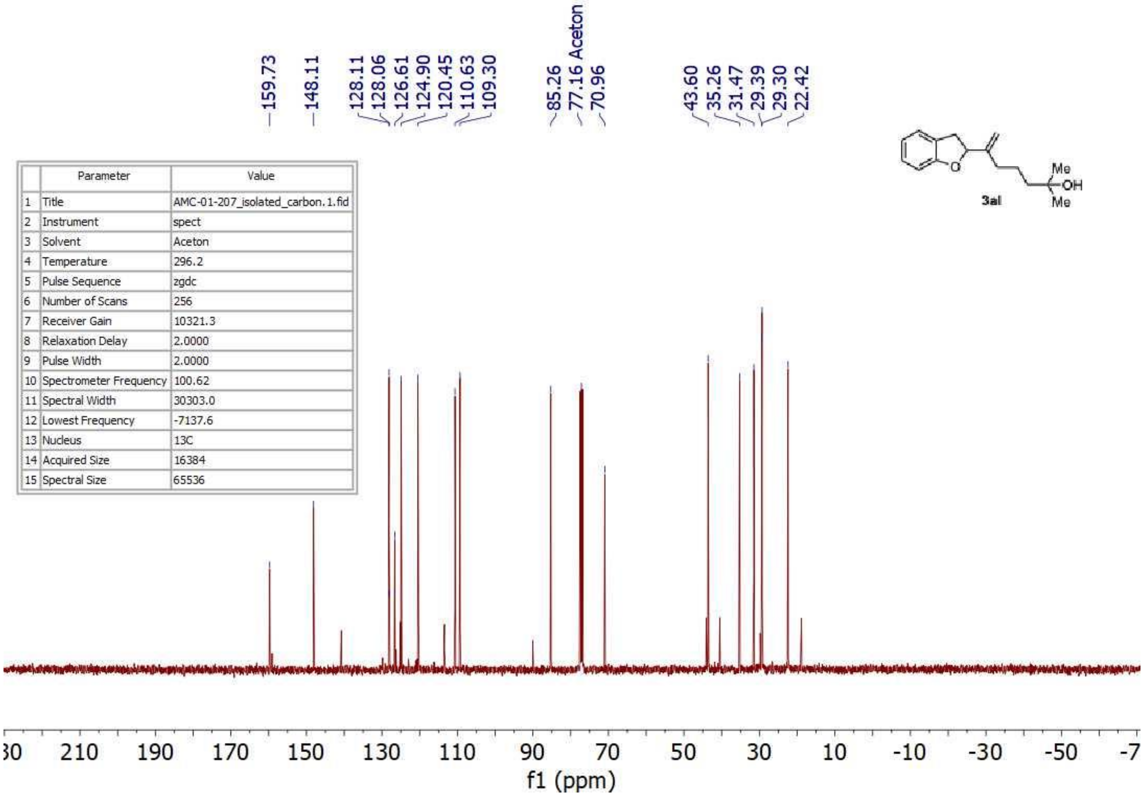

(*E*)-2-(hex-1-en-1-yl)-2-methyl-2,3-dihydrobenzofuran (3am)

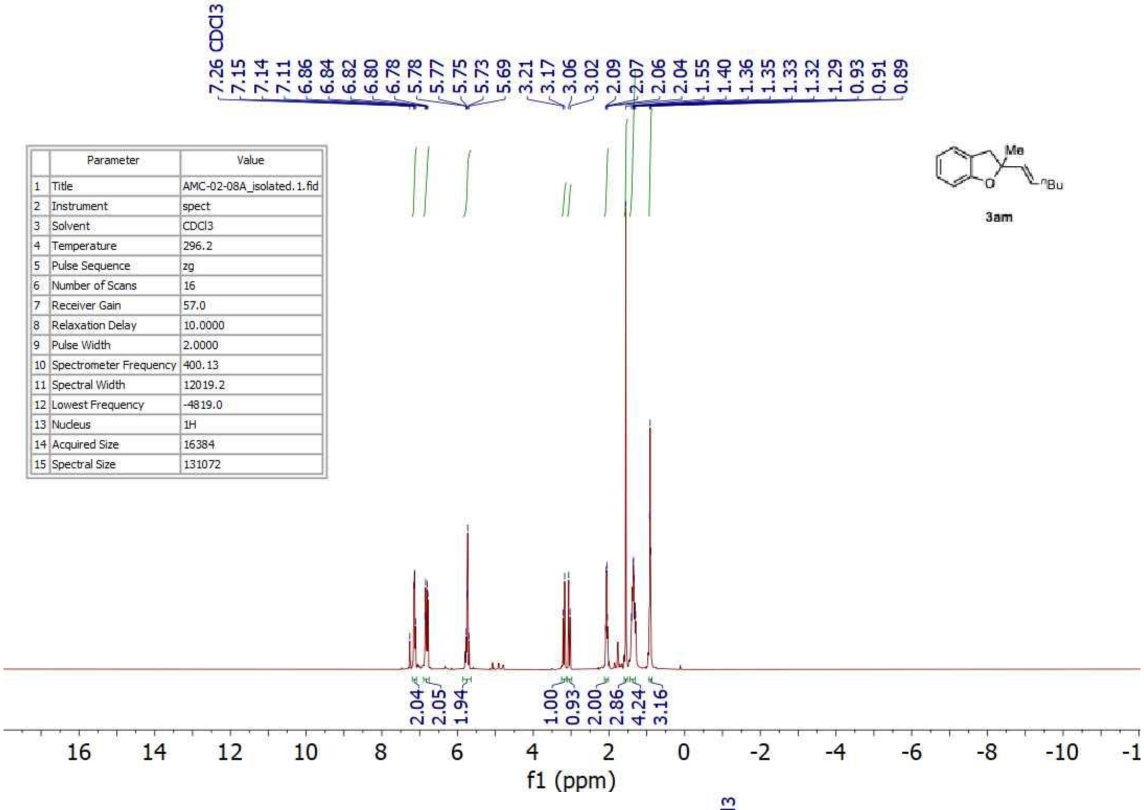

	Parameter	Value
1	Title	AMC-02-08A_isolated.1.fid
2	Instrument	spect
3	Solvent	CDCl3
4	Temperature	296.2
5	Pulse Sequence	zg
6	Number of Scans	16
7	Receiver Gain	57.0
8	Relaxation Delay	10.0000
9	Pulse Width	2.0000
10	Spectrometer Frequency	400.13
11	Spectral Width	12019.2
12	Lowest Frequency	-4819.0
13	Nucleus	1H
14	Acquired Size	16384
15	Spectral Size	131072

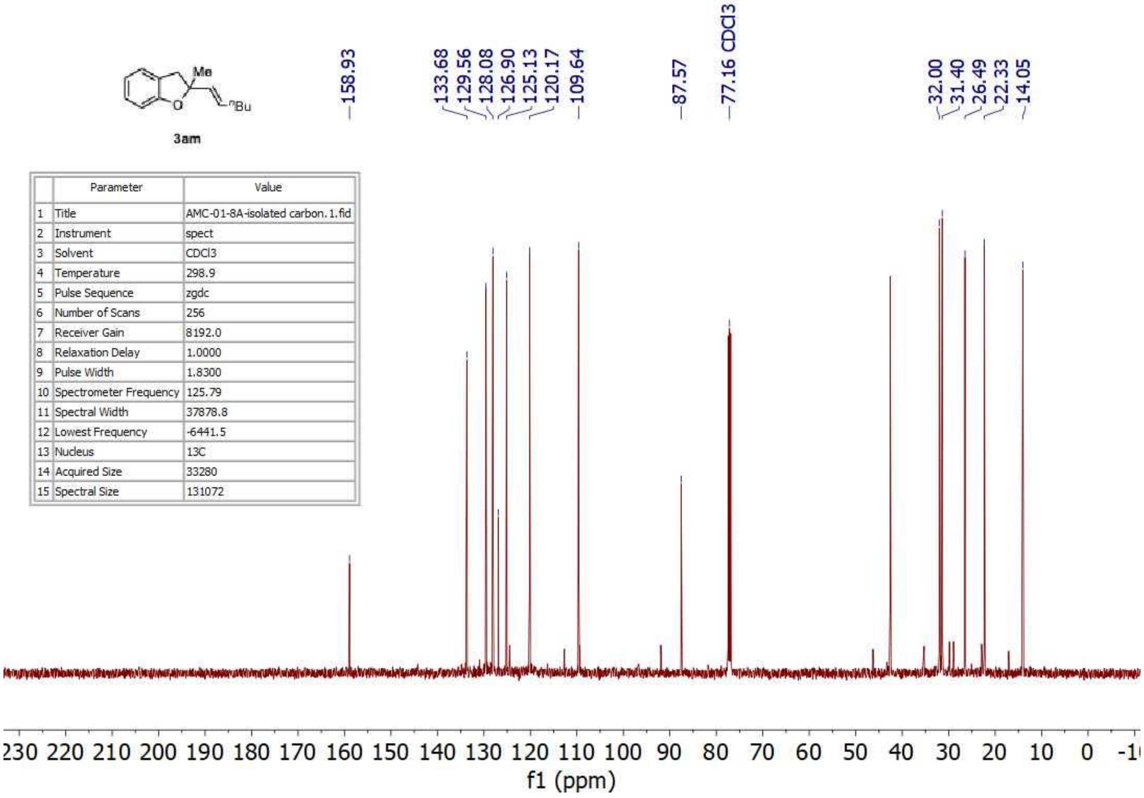

	Parameter	Value
1	Title	AMC-01-8A-isolated carbon.1.fid
2	Instrument	spect
3	Solvent	CDCl3
4	Temperature	298.9
5	Pulse Sequence	zgdc
6	Number of Scans	256
7	Receiver Gain	8192.0
8	Relaxation Delay	1.0000
9	Pulse Width	1.8300
10	Spectrometer Frequency	125.79
11	Spectral Width	37878.8
12	Lowest Frequency	-6441.5
13	Nucleus	13C
14	Acquired Size	33280
15	Spectral Size	131072

(*E*)-2-(1-phenylprop-1-en-2-yl)-2,3-dihydrobenzofuran (3an)

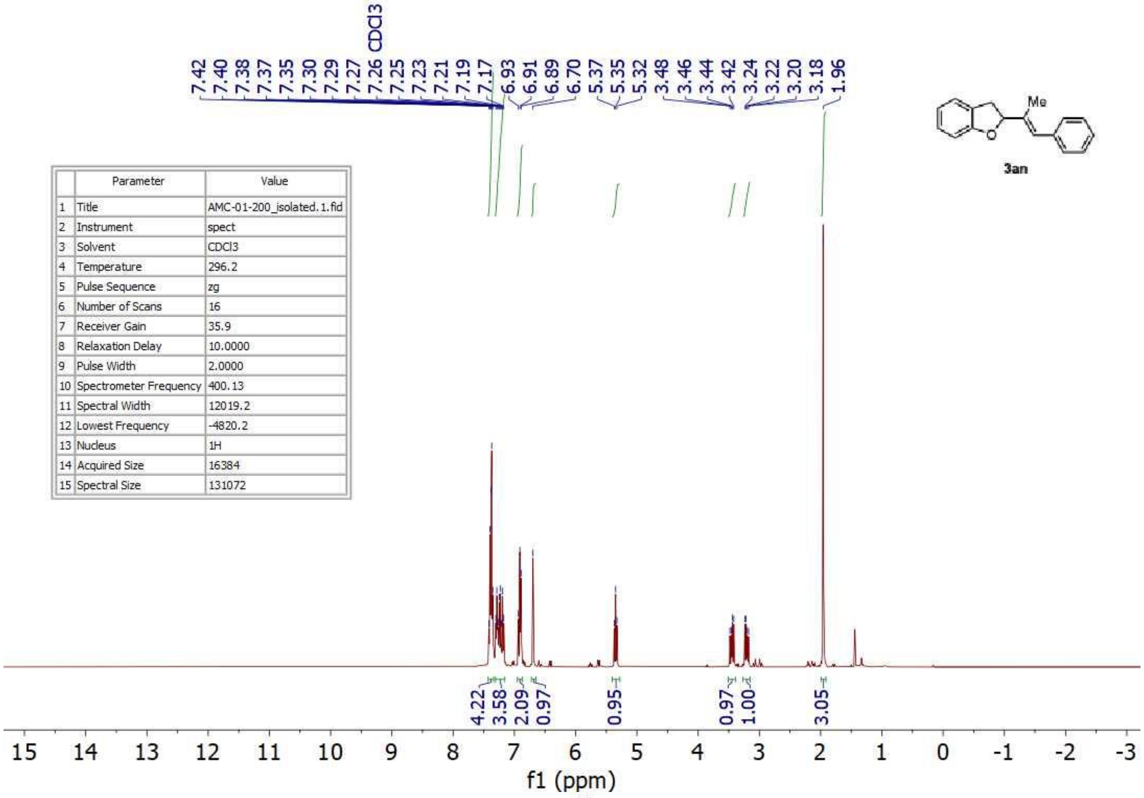

	Parameter	Value
1	Title	AMC-01-200_isolated.1.fid
2	Instrument	spect
3	Solvent	CDCl3
4	Temperature	296.2
5	Pulse Sequence	zg
6	Number of Scans	16
7	Receiver Gain	35.9
8	Relaxation Delay	10.0000
9	Pulse Width	2.0000
10	Spectrometer Frequency	400.13
11	Spectral Width	12019.2
12	Lowest Frequency	-4820.2
13	Nucleus	1H
14	Acquired Size	16384
15	Spectral Size	131072

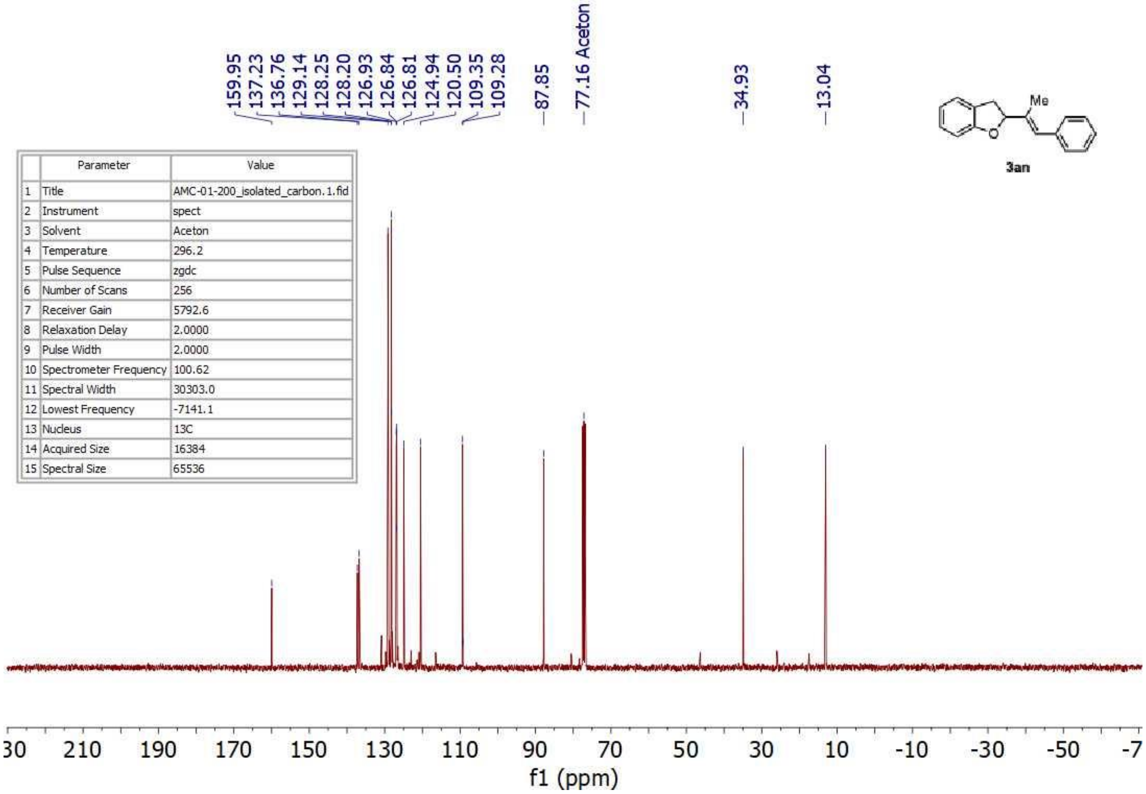

	Parameter	Value
1	Title	AMC-01-200_isolated_carbon.1.fid
2	Instrument	spect
3	Solvent	Aceton
4	Temperature	296.2
5	Pulse Sequence	zgdc
6	Number of Scans	256
7	Receiver Gain	5792.6
8	Relaxation Delay	2.0000
9	Pulse Width	2.0000
10	Spectrometer Frequency	100.62
11	Spectral Width	30303.0
12	Lowest Frequency	-7141.1
13	Nucleus	13C
14	Acquired Size	16384
15	Spectral Size	65536
